# Endocyclic Trisubstituted Hydroxylamine Isosteres of Basic Amines for ADME Modulation and Reduction of hERG Activity

**DOI:** 10.1021/acsmedchemlett.6c00062

**Published:** 2026-03-20

**Authors:** Iftikhar Khan, Asiri A. Hettikankanamalage, Yizhi Cui, David Crich

**Affiliations:** † Innovations in Drug Discovery Program, Department of Pharmaceutical and Biomedical Sciences, 1355 University of Georgia, 250 West Green Street, Athens, Georgia 30602, United States; ‡ Department of Chemistry, University of Georgia, 302 East Campus Road, Athens, Georgia 30602, United States; § Complex Carbohydrate Research Center, University of Georgia, 315 Riverbend Road, Athens, Georgia 30602, United States

**Keywords:** Bioisostere, hydroxylamine, 1,2,5-oxadiazepane, PAK1, hERG

## Abstract

The attachment of solvent-exposed basic amines to kinase inhibitors via an aliphatic chain is a strategy commonly employed to increase aqueous solubility but one that is often complicated by increased human ether-a-go-go (hERG) activity and reduced metabolic stability. Extrapolating from previous work on the replacement of the solubilizing morphilinoalkyl and *N*-methyl piperazinoalkyl chains in the tyrosine kinase inhibitors gefitinib and bosutinib by isosteric hydroxylamines with concomitant reduction in hERG activity without loss of potency against the target kinases, using the PAK1 kinase inhibitor FRAX1036 as model, we describe our exploration of endo- and exocyclic hydroxylamine units as replacements of solubilizing *N*-methyl-4-piperidinylalkyl chains. These studies culminate with the development of the 5*N*-methyl-1,2,5-oxadiazepan-5-yl moiety as an isosteric replacement of the parent *N*-methyl-4-piperidinylalkyl group without loss of potency as a PAK1 inhibitor, a 2-fold increase in selectivity over hERG inhibition, parent-like rates of metabolism by human liver microsomes and MDCKII and Caco-2 efflux ratios.

## Graphical abstract

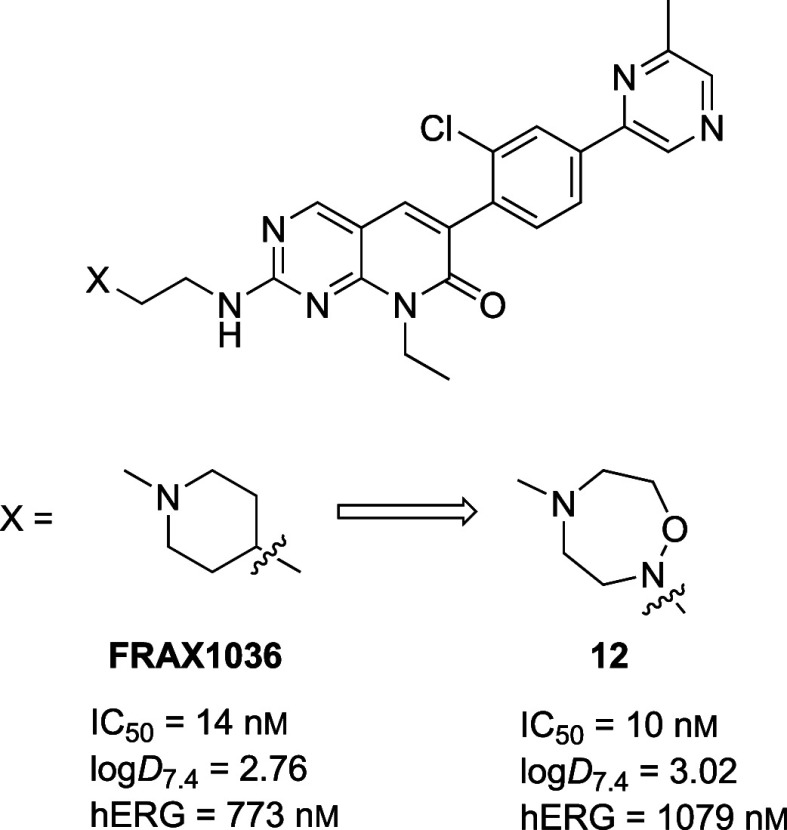

Trisubstituted hydroxylamines, or hydroxalogs, display useful properties when employed as bioisosteres of tertiary amines, ethers, and even hydrocarbon moieties as revealed by a matched molecular pair analysis.^1^ Such properties include the modulation of log*P*, log*D*_7.4_, solubility, protein plasma binding, membrane permeability, metabolism by liver microsomes and hepatocytes, and reduction in hERG inhibition when compared to isosteric tertiary amines. We illustrated these advantageous properties through the replacement of the morpholinoalkyl and *N*-methylpiperazinylalkyl side chains in the mutation-activated EGFR and BCR-ABL tyrosine kinase inhibitors (TKIs) gefitinib (**1**) and bosutinib (**2**), respectively, by the morpholinooxyalkyl residue in **3** and the *N*-methylpiperazinyloxyalkyl units in **4** and **5** (Figure 1)^2–4^ and rationalized them in terms of the modulation of conjugate acid p*K*_a_ on conversion of basic amines to the corresponding hydroxylamines.^3^

**Figure 1:**
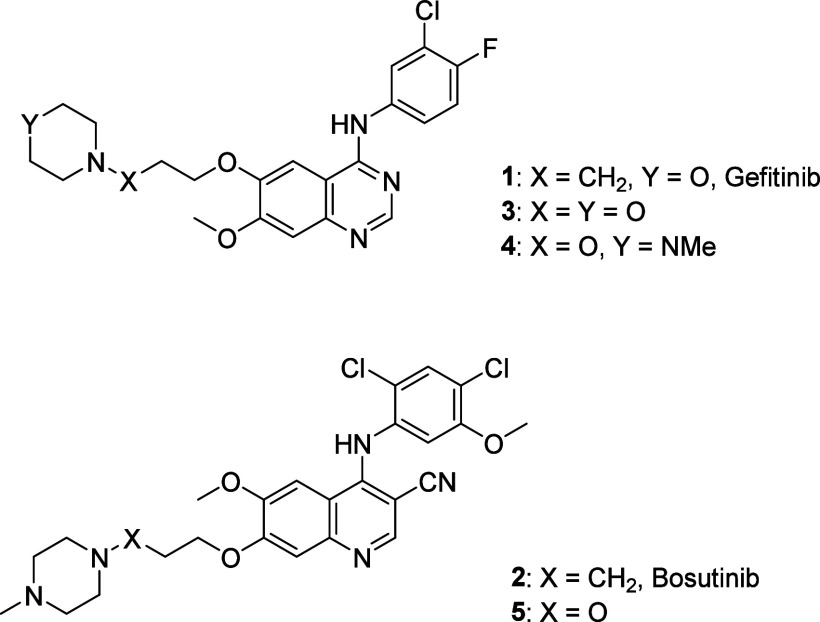
Hydroxalogs **3**, **4**, and **5** of gefitinib and bosutinib, **1** and **2**.

In **1** and **2**, the basic amine serves as a branch point for attachment to the TKI core via a propyloxy chain such that the corresponding hydroxalogs **3**–**5** can be considered to be internal and minimally exposed modifications. Looking to further probe the utility of the tertiary amine to trisubstituted hydroxylamine switch we turned to external tertiary amines in which the tertiary amine unit is at the terminus of the chain through which it is appended to the core of the TKI. We selected as a probe the p21 activated kinase (PAK) inhibitor FRAX1036, **6**, (Figure 2),^5^ with its solubilizing tertiary amine unit attached to the core of the inhibitor via a 4-piperdinylethyl moiety.

**Figure 2:**
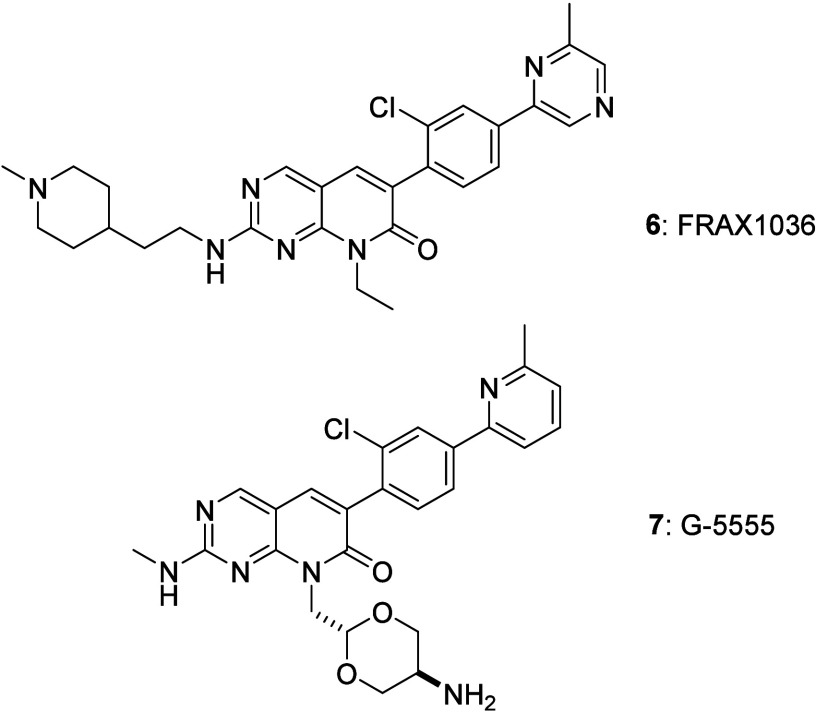
PAK1 inhibitors FRAX1036 **6** and G-5555 **7**.

PAKs are divided into two families, group I (PAKs 1–3) and group II (PAKs 4–6), and PAK1 is a promising target for therapeutic intervention in many cancers as loss of PAK1 function is well-tolerated in normal cells and whole organisms but is lethal to many tumors and metastatic lesions that overexpress it.^6–10^ As such much effort has been focused on the development of PAK1 inhibitors, both orthosteric and allosteric, the latter with occupation of sites more or less remote from the ATP binding site.^8,9,11,12^ FRAX1036, **6**, is a potent inhibitor of PAK1 with moderate selectivity over group II PAKs. However, it suffers from relatively high levels of human ether-a-go-go (hERG) voltage-gated potassium channel inhibition likely arising from the presence of the piperidinyl moiety.^5^ Indeed, redesign of **6** gave G-5555 (**7**) with a corresponding reduction in hERG activity, as well as increased potency and stability.^5,13^ G-5555 (**7**) was ultimately shown to suffer from acute cardiovascular toxicity in mice that was traced to its poor PAK1/PAK2 selectivity, which is a feature of the entire series of pyrido­[2,3-*d*]­pyrimidin-7-one class of PAK1 inhibitors.^14^ Notwithstanding this setback, interest in PAK1 inhibitors continues, as evidenced by the recent publication of PAK1 degraders including one based on the pyrido­[2,3-*d*]­pyrimidin-7-one core.^15,16^

The terminal position of the piperidine moiety in **6** led us to design the directly isosteric tetrahydro-1,2-oxazine **8**, as well as what might be considered the more conventional hydroxalogs **9**–**11** based on the alkoxypiperazine moiety featured in our earlier compounds **4** and **5**. The 1,2-isoxazolidines are frequent motifs in medicinal chemistry,^17^ and their much less common homologues, the tetrahydro-1,2-oxazines, have also been noted to have useful properties.^18^ Seeking to retain the intermediate basicity of the alkoxypiperazine unit^3^ without the need for chain extension (as in **9**) or the addition of a protruding terminal methoxy group (as in **10** and **11**), we designed the oxadiazepanes **12** and **13** in which the hydroxylamine moiety is endocyclic (Figure 3). The *N*-methoxyamine function displayed in **10**, albeit rare, is a feature of the narrow spectrum tetracycline class antibiotic sarecycline^19^ and of the insecticide spiropidion among others.^20,21^ We report here on the synthesis of **8**–**13** and their evaluation for PAK activity and selectivity, hERG activity, and various ADME parameters.

**Figure 3:**
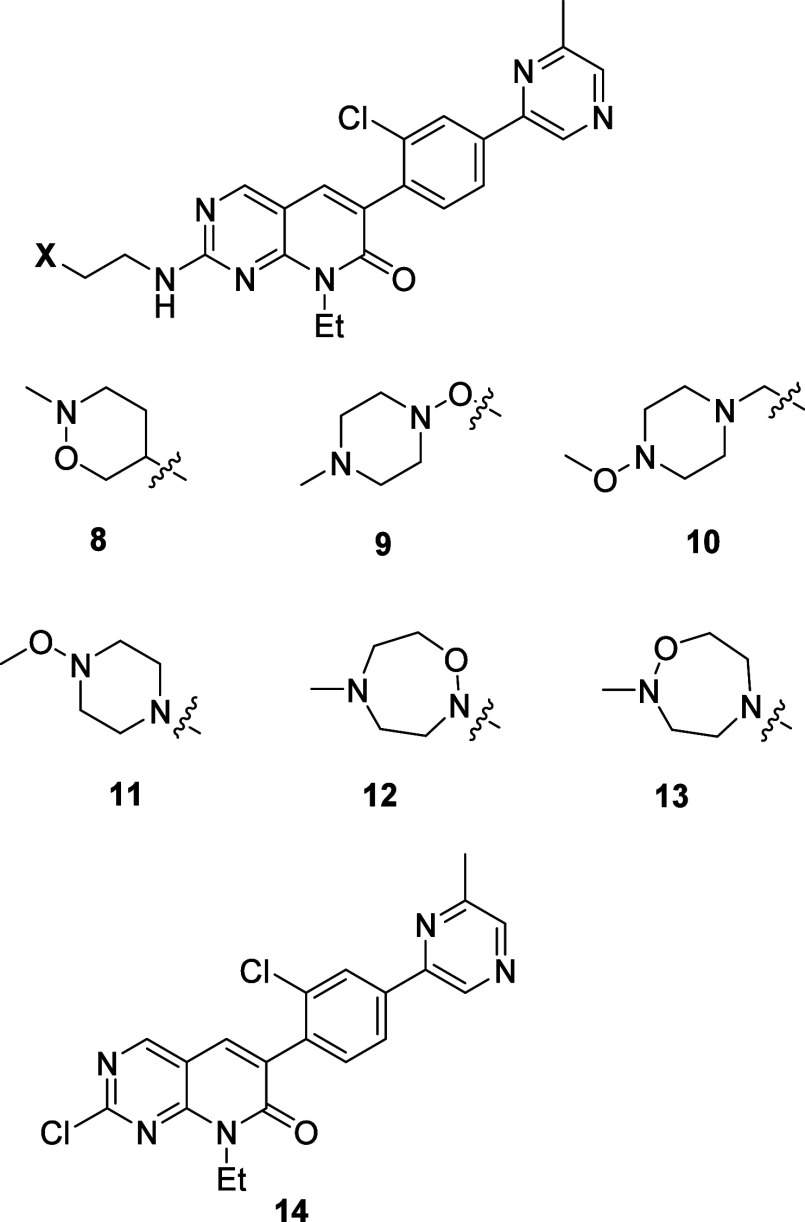
Target hydroxalogs **8**–**13** and core **14**.

Authentic FRAX1036, **6**, was prepared by nucleophilic aromatic substitution of chloride **14** with 2-(1-methylpiperidinyl)­ethylamine essentially according to a literature protocol,^22^ and it was envisaged that hydroxalogs **8**–**13** would be similarly accessible by nucleophilic aromatic substitution of **14** with the corresponding amines. Turning first to the tetrahydro-1,2-oxazine **8**, alcohol **15** ^23^ was converted under Appel conditions to the bromide **16** followed by displacement with the sodium salt of *N*-Boc hydroxylamine in THF to give **17** in high overall yield. Deprotonation with sodium hydride and exposure to allyl bromide then gave **18**, which was heated to reflux in dichloromethane with 5 mol % of the second generation Grubbs ring-closing metathesis catalyst,^24^ when the dihydrooxazine **19** was obtained in 98% yield. Hydrogenation over palladium on carbon with concomitant hydrogenolysis of the benzyl ether gave a 76% yield of **20**, which was converted to azide **21** in 74% yield via the corresponding bromide. Boc removal with TFA was followed by reductive amination with formaldehyde to give **22**, which was exposed to trimethylphosphine in the presence of sodium hydroxide to give the requisite amine **23** (Scheme 1).

**Scheme 1:**
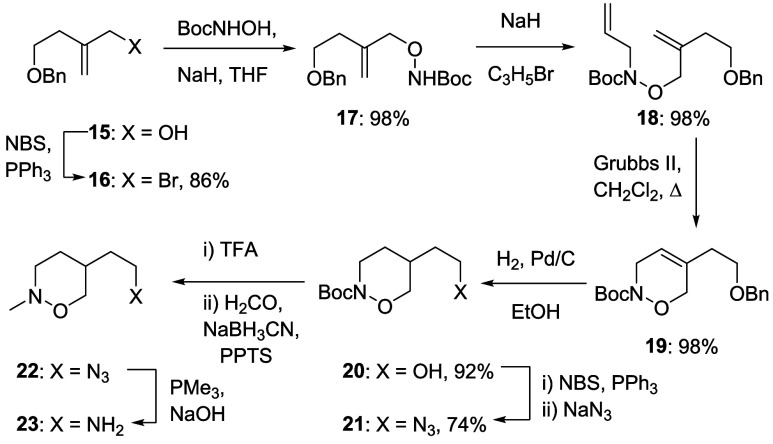
Synthesis of the Tetrahydro-1,2-oxazinylamine **23**

The chlorination of alcohol **24** ^3^ was followed by treatment with sodium azide to afford **25** in 31% over 2 steps. Subsequent azide reduction with trimethylphosphine accessed the corresponding amine **26** in 66% yield. Ozonolysis of commercial *N*-Boc pyrroline **27** with dimethyl sulfide workup, followed by reaction with methoxyamine hydrochloride and sodium cyanoborohydride,^25^ afforded the reductive amination product **28** in 65% over 2 steps. Boc removal with TFA followed by alkylation with 3-bromopropanol furnished compound **30** in 83% yield over 2 steps. Alcohol **30** then was converted to the corresponding chloride and subsequently to azide **31** by standard means in 67% yield over 2 steps. Azide reduction with trimethylphosphine afforded the corresponding amine **32** in 81% yield. Alkylation of **29** with 2-(2-bromoethyl)­isoindoline-1,3-dione **33** and subsequent hydrazinolysis provided primary amine **35** (Scheme 2).

**Scheme 2:**
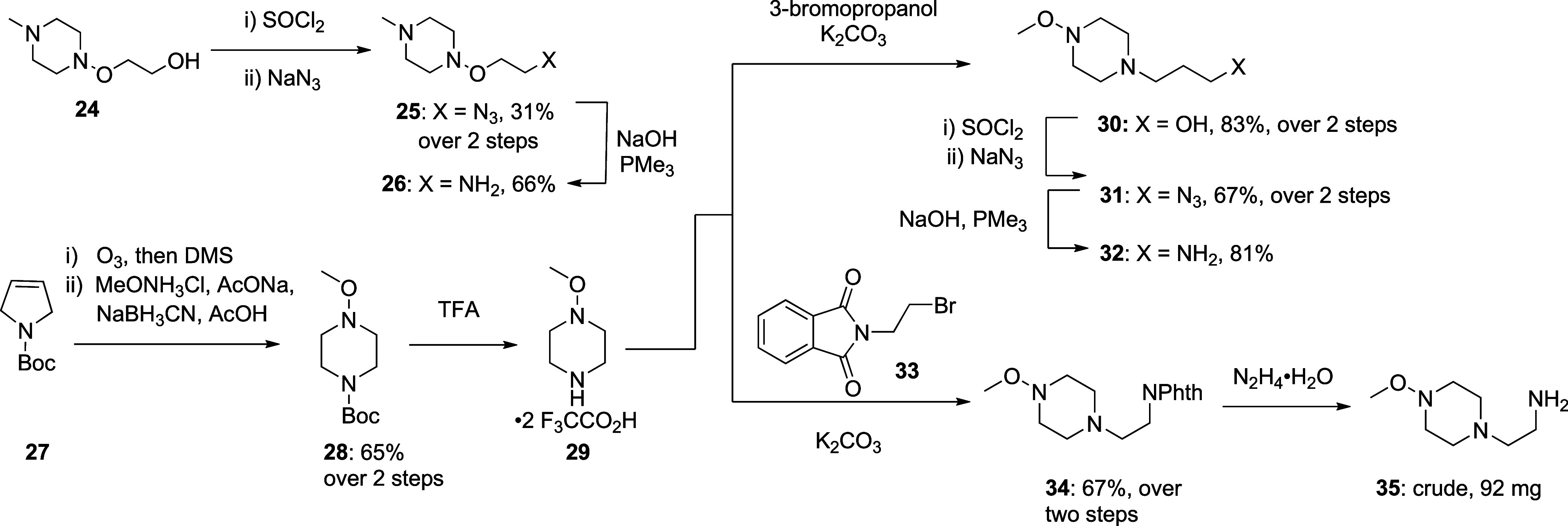
Synthesis of the Alkoxypiperidinyl Amines **26**, **32** and **35**

Treatment of *N*-Boc-bis­(2-chloroethyl)­amine **36** with *N*-alloc-hydroxylamine in the presence of NaH gave oxadiazepane **37** in 37% yield.^26,27^ Exposure of **37** to catalytic tetrakis­(triphenylphosphine)­palladium then gave *N*-allyl-oxadiazepane **38**,^28^ which underwent ozonolysis to form alcohol **39** in 88% yield. Alcohol **39** was converted to the corresponding mesylate **40** in the standard manner and then, by treatment with sodium azide, to azide **41** in 74% yield. Boc group removal with TFA and reductive amination then afforded *N*-methyl compound **42** from which amine **43** was obtained in 53% yield with PMe_3_ and sodium hydroxide. Oxadiazepine **44** was obtained in 42% yield by reaction of *N*-Boc bis­(2-chloroethyl)­amine **36** and benzyl *N*-hydroxycarbamate in the presence of sodium hydride. Careful removal of the Cbz group by hydrogenolysis over palladium on carbon at atmospheric pressure was followed by reductive amination with formaldehyde to afford compound **46** in 82% yield. TFA removal of the Boc group and subsequent alkylation with **33** gave **48**, which upon hydrazinolysis with hydrazine monohydrate provided the corresponding amine **49** in 68% yield (Scheme 3).

**Scheme 3:**
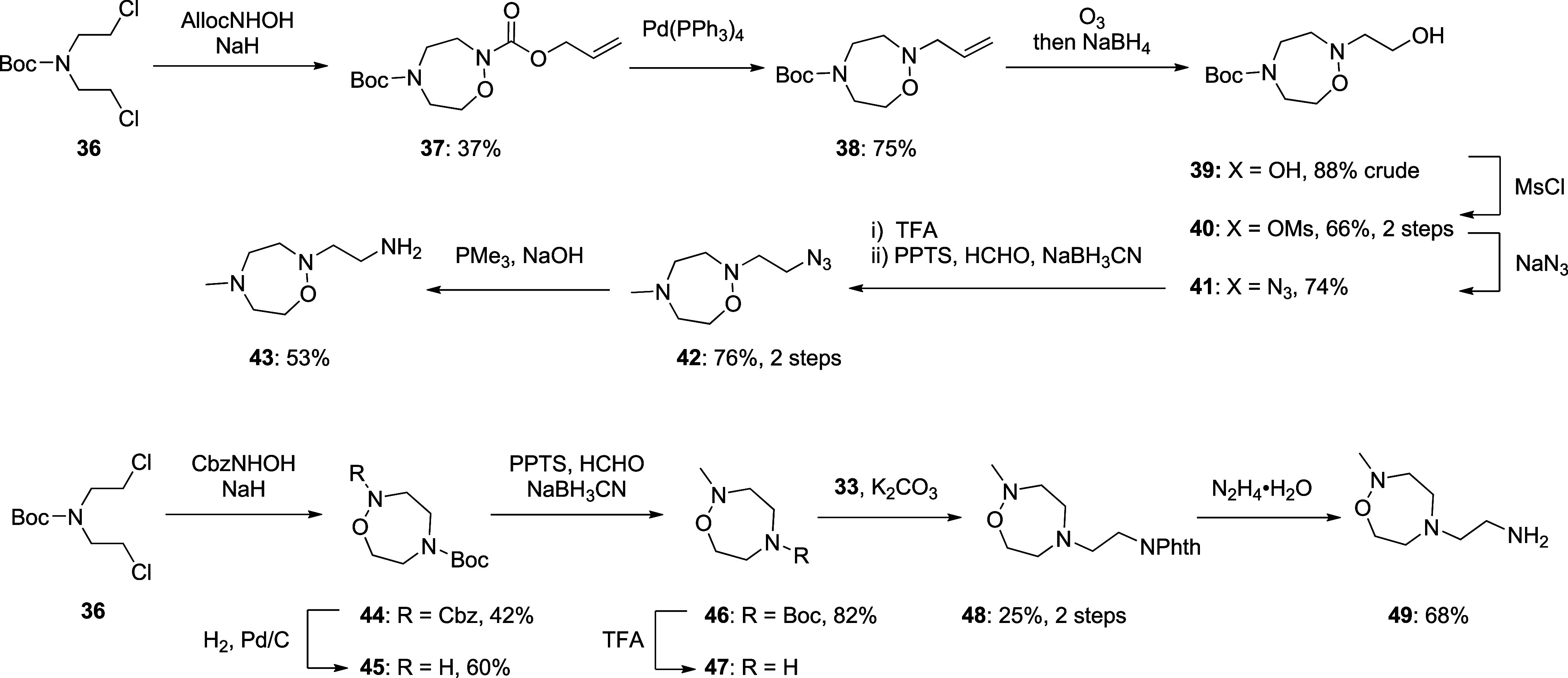
Synthesis of the Oxadiazepanyl Amines **43** and **49**

Finally, as anticipated, all analogs of FRAX1036, **6**, were obtained by nucleophilic aromatic substitution of **14** with the above synthesized amines in DMF at 85 °C with the yields reported in Table 1.

**Table 1:**
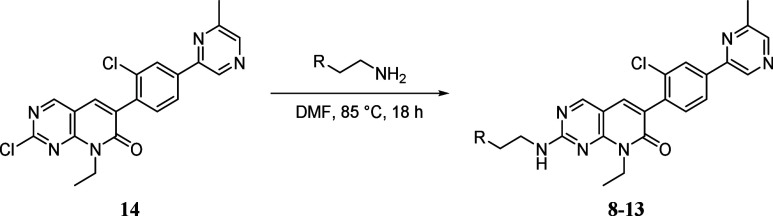

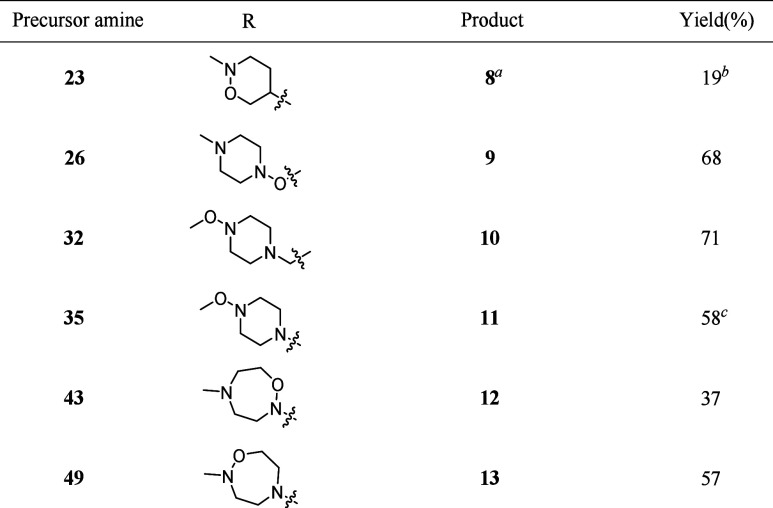
Synthesis of **8**–**13** by S_N_Ar of **14**

a 6 h reaction.

b Yield over three steps from **21**.

c Yield over two steps from **34**.

Log*D*_7.4_ was determined for all compounds (Table 2). The closest isostere to the parent **6**, the tetrahydro-1,2-oxazine **8**, shows the largest increase (Δ1.7) in log*D*_7.4_ consistent with the reduction in basicity from a typical tertiary amine to a hydroxylamine. The smallest increase in log*D*_7.4_ over the parent **6** is presented by the oxadiazepane **12** (Δ0.3) consistent with it having the greatest structural homology with **6** in terms of the placement of the tertiary amine unit. The solubility of all compounds was determined in PBS buffer at pH_7.4_ (Table 2) and correlates with log*D*_7.4_, showing the dramatic effect of the basicity of the side chain amine on the solubility at physiological pH in this series of compounds. On the other hand, although not determined for all compounds, the data reveal that solubility is fully recovered in PBS at p*H* 1.2 even for the least basic tetrahydro-1,2-oxazine **8**. All compounds assayed also showed excellent solubility in the SGF buffer (Table 2). Little variation in PPB, whether human or rat, was observed across the entire series of compounds (Table 2) indicating that this is driven by the core of the molecule rather than by the side chain.

**Table 2:** Log*D*_7.4_, Solubility (μM), and Human (H) and Rat (R) Protein Plasma Binding (%)

	**6**	**8**	**9**	**10**	**11**	**12**	**13**
Log*D*_7.4_	2.76	4.46	3.37	3.75	3.66	3.02	3.64
sol PBS pH 7.4	229.3	0.36	23.6	4.67	1.23	46.2	9.8
sol PBS pH 1.2	292.9	277.8	nd	nd	nd	279.9	nd
sol SGF	280.5	290.5	298.6	301.7	nd	277.2	nd
PPB, H/R	99.4/95.9	99.9/99.9	98.2/98.2	99.0/99.2	98.4/98.1	98.6/96.5	98.8/98.8

IC_50_ values were determined by Eurofins using a Human STE Kinase Enzymatic Radiometric [10 μM ATP] assay for all compounds against the target PAK1, the close homologue PAK2, whose inhibition was determined to be the main source of toxicity of the pyrido­[2,3-*d*]­pyrimidin-7-one class of PAK1 inhibitors,^14^ and PAK4 as a representative of the group II PAKs (Table 3). Considerable variability in terms of PAK1 inhibition was observed across the series with the oxadiazepane **12** being equipotent to the parent **6**, but with compounds **10**, **11**, and **13** displaying a 10–20-fold loss of activity at the other extreme and compounds **8** and **9** showing a more modest 5- or 3-fold drop in activity. The reduction in activity seen with compounds **10**, **11**, and **13** and to a lesser extent with **8** and **9** can be ascribed to the absence of a sufficiently basic terminal amine correctly placed to form a salt bridge with Asp106 that is featured in the PAK1/**6** complex (PDB: 5DFP).^5^ The subtle nature of this interaction is apparent from the only 3-fold reduction in activity exhibited by **9**, whose tertiary amine is only one bond further from the core of the molecule than that of **6**, and the only 5-fold reduction in activity of **8** with its correctly placed but weakly basic hydroxylamine, compared to **10**, **11**, and **13** with their approximately correctly placed but even less basic hydroxylamines.^29^ Compound **12** on the other hand has a correctly located tertiary amine that retains sufficient basicity to take full advantage of the salt bridge to Asp106 and thus retains the full activity of the parent. None of the modifications introduced resulted in a significant change in selectivity for PAK1 over PAK2, while, for all compounds for which PAK4 activity was determined, very high selectivity for PAK1 over PAK4 was retained (Table 3).

**Table 3:** PAK (IC_50_, nM)^*a*^ and hERG (IC_50_, nM)^a^ Inhibition and Selectivity

	**6**	**8**	**9**	**10**	**11**	**12**	**13**
PAK1	14 ± 0.6	75 ± 9.4	43 ± 3.3	274 ± 44	137 ± 7.5	10 ± 2.5	208 ± 2.0
PAK2	55 ± 1.3	344 ± 14	120 ± 1.0	487 ± 4.5	236 ± 44	20 ± 0.3	373 ± 0.5
PAK2/PAK1	3.9	4.6	2.8	1.8	1.7	2.0	1.8
PAK4	>10,000	>10,000	>10,000	>10,000	nd	nd	nd
hERG	773 ± 134	7963 ± 942	5175 ± 191	2159 ± 250	3281 ± 214	1079 ± 114	1379 ± 111
hERG/PAK1	55	106	120	7.9	24	108	6.7

a IC_50_ values are given as the mean ± standard error of means (SEM) from two duplicate experiments.

hERG IC_50_ values (Table 3) were determined by Pharmaron using the manual patch clamp method and vary over an almost 10-fold range with the least active compound being the least basic **8** and the most active compound being **12** with the terminal basic amine. The pattern of hERG activity observed across the series of compounds is consistent with standard hypotheses of the influence of lipophilicity and basicity on binding to the hERG channel.^5,30–33^ Notwithstanding the largely parallel trends for the influence of amine basicity on PAK1 and hERG activity, compound **12** retained high PAK1 inhibition comparable to the parent compound **6** but displayed a 2-fold increase in selectivity over hERG inhibition. Compounds **8** and **9** also showed an approximately 2-fold increase in selectivity for PAK1 over hERG inhibition at the expense of only a moderate reduction in PAK1 activity. These results are consistent with the pattern observed earlier for inhibition of the BCR-ABL kinase in relation to hERG activity by compound **5** in relation to bosutinib **2**.^3^

Finally, with the assistance of Pharmaron, we returned to the influence of hydroxalogs on ADMET properties and more specifically to metabolism by human (H) and rat (R) hepatocytes and on bidirectional permeability across Caco-2 and MDCKII wild-type cell membranes (Table 4). In human hepatocytes all hydroxalogs but two, **8** and **13**, were equally or in most cases slightly more resistant to metabolism than the parent **6** confirming our earlier observations of the relative stability of the trisubstituted hydroxylamine moiety.^1–4^ The two exceptions, **8** and **13**, carry an exposed endocyclic hydroxylamine at the terminus of the side chain and, while still moderately stable with *t*_1/2_ values of 57 and 80 min, respectively, are noticeably less so than all other compounds. Presumably, this reduction in stability arises because of the unprotonated nature of the hydroxylamine function at physiological pH and the exposed nature of the hydroxylamine α-C–H bonds, which together facilitate oxidation by cytochromes. The parent piperidine has equally exposed C–H bonds adjacent to the basic nitrogen atom, but the protonated nature of the compound under physiological conditions considerably strengthens them^34^ and so blocks oxidation. Of considerable interest is the relative stability of the highly exposed *N*-methoxypiperidines **10** and **11** whose methyl C–H bonds might have been considered highly susceptible to oxidation by cytochromes; evidently the few kcal/mol increase in C–H bond strength expected on going from the secondary C–H bonds adjacent to the hydroxylamine oxygen in **8** and **13** to the methyl C–H bonds in **10** and **11** is sufficient to suppress their oxidative metabolism. All compounds were significantly less stable when exposed to the more aggressive rat hepatocytes (Table 4).

**Table 4:** Metabolic Stability in Human (H) and Rat (R) Hepatocytes and Bidirectional Permeability across Caco-2 and MDCKII Wild-Type Cell Membranes

	**6**	**8**	**9**	**10**	**11**	**12**	**13**
*t*_1/2_ (min) H/R	102.4/87.5	56.9/29.8	111.1/6.5	96.6/12.6	117.2/28.3	112.9/17.7	79.9/11.9
Cl_int_ H/R (μL/min/10^6^ cells)	13.5/15.9	24.4/46.6	12.5/214.3	14.4/109.6	11.9/49.1	12.3/78.2	17.4/116.5
MDCKII Perm. (a–b)/(b–a) (10^6^ cm/s)	4.4/8.1	4.0/3.1	12.7/10.0	11.7/11.0	12.0/9.1	10.7/7.0	6.8/8.6
MDCKII efflux (a–b)/(b–a) ratio	1.8	0.8	0.8	0.9	0.8	0.7	1.3
Caco-2 Perm. (a–b)/(b–a) (10^6^ cm/s)	3.7/10.1	nd	4.0/6.5	4.9/5.5	5.8/6.1	3.4/9.8	5.5/6.9
Caco-2 efflux (a–b)/(b–a) ratio	2.7	nd	1.6	1.1	1.1	2.9	1.3

To assess permeability, as in the original report on **6** and its optimization to **7**,^5^ we employed wild-type Madin–Darby canine kidney (MDCKII) epithelial cells^35,36^ and found the apparent permeability *P*_app_(a–b) to be approximately tripled for hydroxalogs **9**–**12** over the parent amine **6** suggestive of improved cellular activity and oral bioavailability. A more modest increase in *P*_app_ was observed for **13**, while no significant change in *P*_app_ was seen for the simple tetrahydro-1,2-oxazine **8** (Table 4). We also investigated permeability in the widely employed colon carcinoma (Caco-2) cells^37,38^ for comparison purposes with earlier hydroxalogs of EGFR and BCR-ABL TKIs.^2–4^ We find, in agreement with the earlier work, that hydroxalogs generally present smaller Caco-2 efflux ratios than the corresponding amines (Table 4), which is consistent with current hypotheses linking lipophilicity (log*D*_7.4_) and permeability,^39^ blood–brain barrier penetration,^40,41^ and even intracellular diffusion.^42^ The one exception to the general trend of reduced Caco-2 efflux is compound **12** whose efflux ratio of 2.9 is comparable to that of parent compound **6** (2.7), which presumably arises from the comparable lipophilicity (log*D*_7.4_) of the two compounds.

Overall, six hydroxylamine-based isosteric replacements of the pendant, water-solubilizing *N*-methylpiperidin-4-yl moiety in the allosteric PAK1 inhibitor FRAX1036 **6** were designed and prepared. The closest isostere, the *N*-methyl-tetrahydro-1,2-oxazin-5-yl derivative **8**, showed a increase in log*D*_7.4_ that was coupled with 5-fold reduction in potency against PAK1 and a 10-fold reduction in hERG inhibition. Other isosteres in which the basic amine was directly replaced by a hydroxylamine moiety also displayed reductions in activity against PAK1 indicating the importance of retaining a basic amine at the terminus of the pendant chain. Ultimately, a novel 5-*N*-methyl-1,2,5-oxadiazepan-2-yl ring was developed as a bioisosteric replacement for the *N*-methylpiperidin-4-yl moiety in the parent compound leading to compound **12** that exhibited a similar log*D*_7.4_, good solubility in PBS buffer at pH_7.4_, parent-like potency against PAK1, a 2-fold increase in PAK1/hERG selectivity, and parent-like rates of metabolism by human liver microsomes and efflux by the MDCKII and Caco-2 cell membranes. As such, the 1,2,5-oxadiazepanyl system merits consideration as a bioisosteric replacement for 5-, 6-, and 7-membered basic amines in other compound optimization campaigns.

## Supplementary Material



## Notes

Biological materials and methods, synthetic procedures, characterization data, Supplementary Tables S1–S25, Supplementary Figures S1–S14, NMR spectra, and references (PDF)

## Abbreviations Used

ABL1: Abelson murine leukemia viral oncogene homologue 1
ATP: adenosine triphosphate
BCRP: breast cancer resistance protein
Caco-2: colon carcinoma cell line
EGFR: epidermal growth factor receptor
hERG: human ether-a-go-go-related gene
H: human
log*D*: logarithm of distribution at pH_7.4_
log*P*: logarithm of partition coefficient
MDCK: Madin–Darby canine kidney cell line
nd: not determined
PAK: p21-activated kinase
PDB: Protein Data Bank
Perm: permeability
PMe_3_: trimethylphosphine
PPB: plasma protein binding
PPTS: pyridinium *p*-toluenesulfonate
SGF: simulated gastric fluid
Sol: solubility
R: rat
TFA: trifluoroacetic acid
