# Supplementary material for: Endocyclic Trisubstituted Hydroxylamine Isosteres of Basic Amines for ADME Modulation and Reduction of hERG Activity
Source: ACS Med Chem Lett. 2026 Mar 20;17(4):925–31. doi: 10.1021/acsmedchemlett.6c00062 (PMC13071656; doi:10.1021/acsmedchemlett.6c00062)
Supplement: Supplementary file 1 [file ml6c00062_si_001.pdf]

Supporting Information

For

Endocyclic Trisubstituted Hydroxylamine Isosteres of Basic Amines for ADME Modulation and Reduction of hERG Activity

Iftikhar Khan,[1] Asiri A. Hettikankanamalage,[1,2] Yizhi Cui,[1,2,] and David Crich[1,2,3,*]

[1]Innovations in Drug Discovery Program, Department of Pharmaceutical and Biomedical Sciences, University of Georgia, 250 West Green Street, Athens, GA 30602, USA.

[2]Department of Chemistry, University of Georgia, 302 East Campus Road, Athens, GA 30602, USA.

[3]Complex Carbohydrate Research Center, University of Georgia, 315 Riverbend Road, Athens, GA 30602, USA.

[*]Corresponding author. Email: David.crich@uga.edu

Table of Contents

1. Biological Methods and Data……………………………..……………………………………..S2
2. Biological Materials and Data……………………………………………………………………S3
3. General Procedures for Chemical Synthesis, Materials and Instrumentation…………S10
4. Synthetic Procedures……………………………………………………………………………S11
5. Catalog of Spectra………………………………………………………………………………S36

1. Biological Methods and Data

Kinase Activity

In vitro kinase activity (biochemical inhibition (IC_{50}'s) were performed by Eurofins Cerep. Each compound was run duplicative trials in an assay using a 9-point, half-log dilution series starting from a compound concentration of 10 μM and an ATP concentration of 10 μM.

Cell lines

The Caco-2 cell line was obtained from ATCC. MCKII-Wild Type cells were obtained from the Netherlands Cancer Institute. HEK293 cells were obtained from Invitrogen. HEK293 cells were cultured in DMEM (Gibco) with 10% FBS, 0.1 mM NEAA, 25 mM HEPES, 100 U/mL penicillin-streptomycin, 5 μg/mL blasticidin and 400 μg/mL geneticin. All cells were cultured in a humidified incubator with 5% CO_2 at 37 °C.

***In-vitro* ADMET**

Lipophilicity, solubility, plasma protein binding, metabolic stability in hepatocytes, permeability studies, and hERG channel inhibition was determined by Pharmaron Inc. using previously described methods.

2. Biological Materials and Data

Table S1 | Inhibitory Activity Against PAKs 1, 2 and 4.

**Compound**	**IC_{50} (nM)**[a]		
	**PAK1**	**PAK2**	**PAK4**
**6**	14 ± 0.6	55 ± 1.3	>10,000
**8**	75 ± 9.4	344 ± 14.1	>10,000
**9**	43 ± 3.3	120 ± 1	>10,000
**10**	274 ± 43.5	487 ± 4.5	>10,000
**11**	137 ± 7.5	236 ± 43.5	-
**12**	10 ± 2.45	20 ± 0.3	-
**13**	208 ± 2	373 ± 0.5	-

[a]IC_{50} values (μM) are reported beside the dose-response curves with ± SEM.

Plasma protein binding by equilibrium dialysis

Table S2 | Plasma details used in the plasma protein binding assay.

Item	Supplier
Human Plasma, Mixed Gender	The second People's Hospital of Liaocheng (Batch No. KBXJ595-(01097-01146)-20240708)
SD Rat Plasma, Mixed Gender	BioIVT (Batch No. RAT544194)

Table S3 | Results for plasma protein binding in human and rat plasma.

Compound	Species	% Bound	% Unbound	% Recovery	% Remaining at 6 h
Ketoconazole	Human	99.10	0.90	97.41	107.73
	Rat	99.46	0.54	96.90	104.16
**6**	Human	99.38	0.62	97.68	99.21
	Rat	95.94	4.06	102.83	102.88
**8**	Human	99.87	0.13	90.54	95.04
	Rat	99.89	0.11	94.84	99.70
**9**	Human	98.19	1.81	93.40	100.75
	Rat	98.15	1.85	100.19	103.29
**10**	Human	99.04	0.96	95.63	103.17
	Rat	99.22	0.78	100.51	102.80
**11**	Human	98.35	1.65	98.92	99.73
	Rat	98.11	1.89	107.67	94.85
**12**	Human	98.56	1.44	96.59	99.48
	Rat	96.51	3.49	101.97	104.82
**13**	Human	98.80	1.20	95.05	100.29
	Rat	98.81	1.19	97.88	103.00

Metabolic stability in human and rat hepatocytes

Table S4 | Hepatocyte details used in the hepatocyte stability assay.

Item	Supplier
Human Hepatocytes, Mixed Gender	BioIVT (Product No. X008000, Lot No. UNQ)
SD Rat Hepatocytes, Male	BioIVT (Product No. M00005, Lot No. CLZ)

Table S5 | Results for hepatocyte stability data in human and rat hepatocytes.

Compound	Species	Remaining Percentage (%)					
		0.5 min	15 min	30 min	60 min	90 min	120 min
Verapamil	Human	100.00	61.44	42.57	20.69	11.54	7.45
	Rat	100.00	9.01	1.84	BLOD	BLOD	BLOD
**6**	Human	100.00	102.12	99.58	81.94	56.70	48.66
	Rat	100.00	88.10	80.10	58.50	46.73	40.09
**8**	Human	100.00	99.18	85.66	59.96	36.19	25.74
	Rat	100.00	66.17	47.80	24.74	11.95	6.20
**9**	Human	100.00	107.95	86.34	78.44	54.52	53.46
	Rat	100.00	21.16	3.67	0.59	0.54	0.54
**10**	Human	100.00	94.41	75.30	66.53	47.72	44.76
	Rat	100.00	50.72	19.87	3.01	1.03	0.46
**11**	Human	100.00	91.62	81.30	68.84	56.98	49.58
	Rat	100.00	71.99	47.64	22.55	11.39	6.15
**12**	Human	100.00	99.19	85.66	74.96	62.04	48.18
	Rat	100.00	58.53	31.56	8.60	4.99	2.57
**13**	Human	100.00	90.98	73.84	58.37	42.13	37.38
	Rat	100.00	45.23	17.96	4.06	1.74	1.16

Abbreviations: BLOD, below level of detection.

Caco-2 permeability

Table S6 | Caco-2 cellular details used in the Caco-2 cellular permeability assay.

Item	Supplier
Caco-2 cells	ATCC (ATCC® No. HTB-37)
HTS Transwell 96 Well	Corning Corp. (Cat No. 3391)

Table S7 | Results for Caco-2 cellular permeability.

Compound	$P_{app\ (A\text{-}B)}$ (10^{-6}, cm/s)	$P_{app\ (B\text{-}A)}$ (10^{-6}, cm/s)	Efflux Ratio	Recovery (%) AP-BL	Recovery (%) BL-AP
Metoprolol	28.92	23.06	0.80	111.35	103.49
Digoxin	0.37	18.20	49.70	91.20	105.80
**6**	3.68	10.07	2.74	47.79	75.26
**9**	4.02	6.50	1.64	53.85	73.57
**10**	4.88	5.54	1.14	53.34	61.03
**11**	5.82	6.13	1.05	55.82	57.90
**12**	3.37	9.77	2.91	55.29	72.98
**13**	5.54	6.94	1.25	59.71	60.59

Abbreviations: Papp, apparent permeability; AP, apical; BL, basolateral.

MDCKII-Wild type Permeability

Table S8 | MDCKII-Wild type cellular details used in the MDCKII-Wild type permeability assay.

Item	Supplier
MDCKII cells (wild type)	The Netherlands Cancer Institute (Amsterdam)
HTS Transwell 96 Well	Corning Corp. (Cat No. 3391)

Table S9 | Results for MDCKII-Wild type Permeability.

Compound	$P_{app\ (A\text{-}B)}$ (10^{-6}, cm/s)	$P_{app\ (B\text{-}A)}$ (10^{-6}, cm/s)	Efflux Ratio	Recovery (%) AP-BL	Recovery (%) BL-AP
Metoprolol	25.47	26.19	1.03	105.66	103.72
Digoxin	1.10	8.59	7.84	111.18	95.05
**6**	4.41	8.10	1.84	41.48	63.06
**8**	4.00	3.08	0.78	43.47	23.63
**9**	12.71	10.02	0.79	67.39	60.85
**10**	11.74	10.98	0.94	66.89	76.93
**11**	11.99	9.10	0.76	65.75	60.51
**12**	10.65	6.95	0.65	56.59	65.46
**13**	6.84	8.61	1.28	59.56	52.19

Abbreviations: Papp, apparent permeability; AP, apical; BL, basolateral.

hERG Safety evaluation by manual patch-clamp system

Table S10 | HEK293 cellular details used in hERG safety evaluation.

Item	Supplier
HEK 293 Cell Line	Invitrogen (Cat No. K1236)
TrypLE™ Express	Invitrogen (Cat No. 12604039)
Dofetilide	TRC (Cat No. D525700)

Table S11 | hERG safety evaluation results.

Test article	hERG IC_{50} [μM][a]
Dofetilide	0.013 ± 0.00035
**6**	0.773 ± 0.1337
**8**	7.963 ± 0.9423
**9**	5.175 ± 0.1907
**10**	2.159 ± 0.2496
**11**	3.281 ± 0.2138
**12**	1.079 ± 0.1144
**13**	1.379 ± 0.1114

[a] IC_{50} values (μM) are reported beside the dose-response curves with ± SEM.

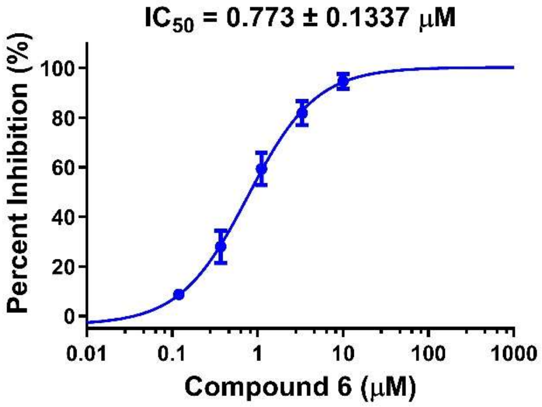

Figure S1 | hERG percent inhibition of compound **6**.

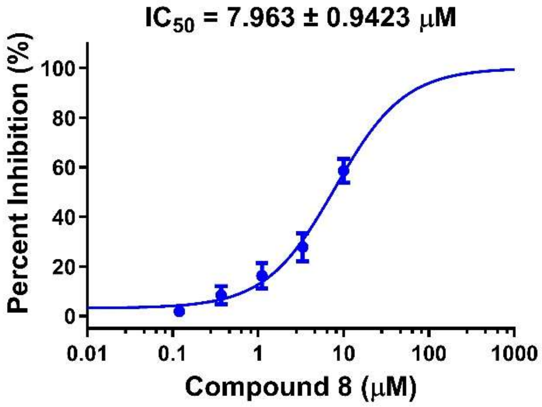

Figure S2 | hERG percent inhibition of compound **8**.

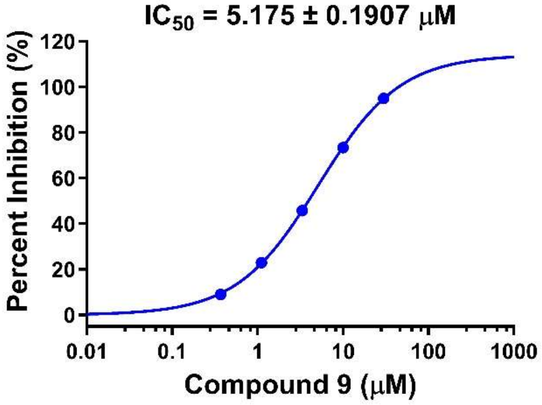

Figure S3 | hERG percent inhibition of compound **9**.

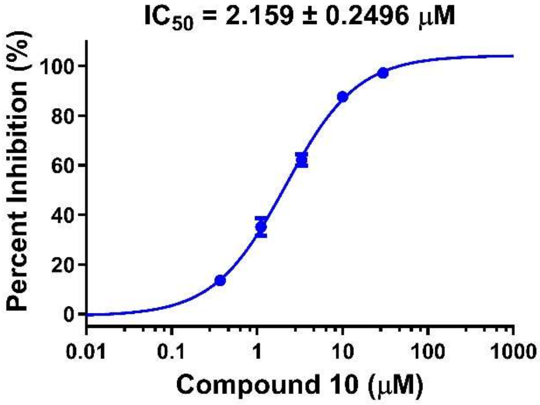

Figure S4 | hERG percent inhibition of compound **10**.

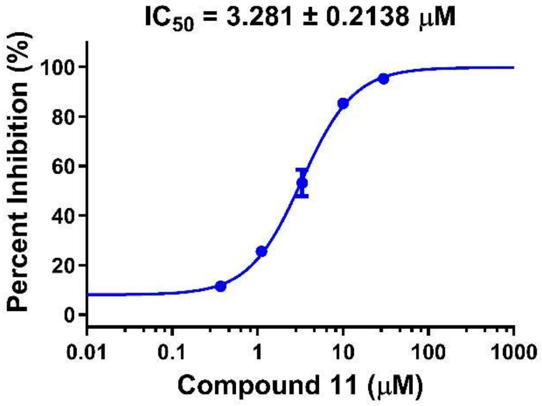

Figure S5 | hERG percent inhibition of compound **11**.

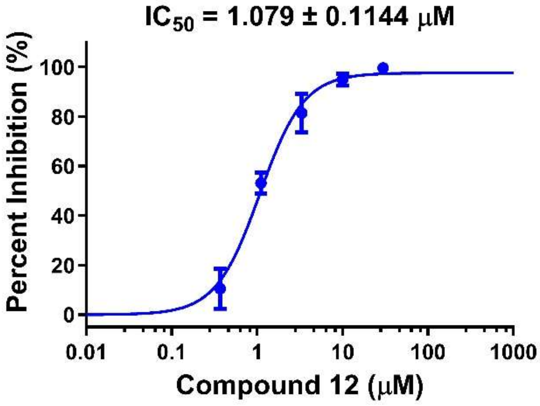

Figure S6 | hERG percent inhibition of compound **12**.

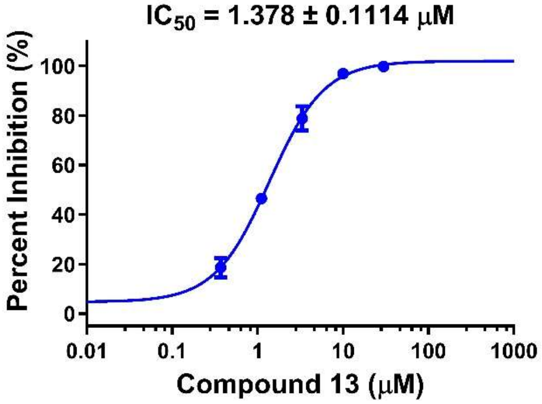

Figure S7 | hERG percent inhibition of compound **13**.

Figure S1-S7 indicates hERG inhibition curves; points indicate mean, and error bars indicate the SD; 2 independent replicates; IC_{50} values (μM) are reported beside the dose-response curves with ± SEM.

3. General Procedures for Chemical Synthesis, Materials and Instrumentation

All reactions were performed in oven dried glassware capped with a rubber septum under argon atmosphere unless otherwise stated. All organic solutions were concentrated under reduced pressure on a rotary evaporator with water bath. Flash column chromatographies were performed using COMBIFLASH® NEXTGEN system with indicated solvent. All thin-layer chromatography (TLC) was carried out with 250 μM glass back silica (XHL) plates. TLC plates were visualized by ultraviolet lamp (254 nm) and/or submersion in ceric ammonium molybdate (CAM) ethanol solution followed by heating on a hot plate (120 °C, 10-15 s). Ozonolysis was conducted with an ozone generator purchased from Ozone Water Systems Inc in well-ventilated fume hood.

Solvents were purchased from Sigma-Aldrich and used without further purification. Commercial reagents were purchased from Sigma-Aldrich, Fisher Scientific, Ambeed Inc, and Oakwood Products Inc and used without purification.

NMR spectra were obtained by dissolving samples in $CDCl_3$ (δH 7.26 and δC 77.16 ppm, respectively), benzene-D_6 (δH 7.16 and δC 128.06), or toluene-D_8 (δH 7.09, 6.98, 7.00, 2.09 and δC 137.86, 129.24, 128.33, 125.49, 20.40 ppm, respectively). All NMR spectra were conducted with JEOL, Varian and Bruker spectrometers (500 MHz, 600 MHz and 900 MHz). Chemical shifts (δ) are given in ppm with respect to solvent peaks, and multiplicities are abbreviated as follows: s (singlet), m (multiplet), br (broad), d (doublet), t (triplet), q (quartet), br s (broad singlet), dd (doublet of doublet), dt (doublet of triplet), dq (doublet of quartet), ddd (doublet of doublet of doublet), dddt (doublet of doublet of doublet of triplet), td (triplet of doublet), tdq (triplet of doublet of quartet), ttd (triplet of triplet of doublet). HRMS were obtained on a ThermoFisher Orbitrap analyzer using electrospray ionization (ESI). Ultra high-performance liquid chromatography (UHPLC) traces were obtained using a ThermoFisher Vanquish UHPLC with PDA detector and an Acclaim 120 18C 4.6 × 50 mm column and the %purity determined by Avalon peak area algorithm.

Safety statement: Although no safety issues have been encountered, the following hazards should be noted: Compound **24** is prepared by chemistry involving the use of peroxides and standard caution (i.e., avoidance of exposure to light, reducing agents, excessive heat, and working behind a protective shield) for such reactions should be exercised. Reactions using palladium on carbon in a hydrogen atmosphere should be performed with avoidance of heat.

4. Synthetic Procedures

(((3-(Bromomethyl)but-3-en-1-yl)oxy)methyl)benzene (16)

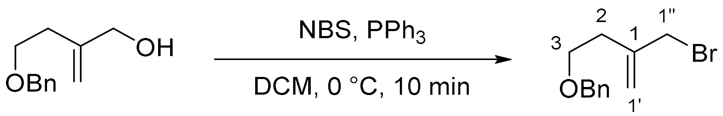

In an oven-dried flask under an argon atmosphere, to a solution of 4-(benzyloxy)-2-methylenebutan-1-ol (800 mg, 4.17 mmol, 1.0 equiv.) in DCM (42.0 mL) was added triphenylphosphine (2.19 g, 8.34 mmol, 2 equiv.) and *N*-bromosuccinimide (1.48 g, 8.34 mmol, 1.3 equiv.) at 0 °C stirred for 10 min. Upon completion, the reaction mixture was diluted with of CH_2Cl_2 (50 mL) and quenched with sat. $NaHCO_3$ (50 mL), and the aqueous layer was extracted with CH_2Cl_2 (5 × 30 mL). The combined organic layers were dried over Na_2SO_4 and concentrated under reduced pressure. The green residue was purified by flash column chromatography (eluent: hexanes → 10% EtOAc in hexane) to provide the title compound (**16**) as a colorless liquid (911 mg, 3.57 mmol, 86%). The experimental data matched with the literature. TLC (silica) R_f = 0.75 (20% EtOAc/hexane, UV, CAM). ^{1}H NMR (600 MHz, $CDCl_3$) δ 7.41 – 7.33 (m, 4H, ArH), 7.34 – 7.28 (m, 1H, ArH), 5.27 (q, *J* = 0.9 Hz, 1H, H1'), 5.07 (q, *J* = 1.3 Hz, 1H, H1'), 4.56 (s, 2H, benzyl CH_2), 4.03 (d, *J* = 0.8 Hz, 2H, H1''), 3.66 (t, *J* = 6.5 Hz, 2H, H3), 2.57 (t, *J* = 6.5 Hz, 2H, H2). ^{13}C NMR (151 MHz, $CDCl_3$) δ 142.9 (C1), 138.2 (Ar), 128.4 (2C, Ar), 127.7(2C, Ar), 127.6 (Ar), 116.6 (C1'), 73.0 (benzyl CH_2), 68.4 (C3), 37.0 (C1''), 33.5 (C2).

***tert*-Butyl (4-(benzyloxy)-2-methylenebutoxy) carbamate (17)**

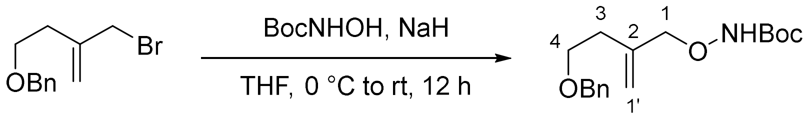

In an oven dried flask under an argon atmosphere, NaH (60 w% dispersion in mineral oil) (57 mg, 1.44 mmol, 1.1 equiv.) was added to a stirred solution of *tert*-butyl *N*-hydroxycarbamate (435 mg, 3.26 mmol, 2.5 equiv.) in anhydrous THF (5.25 ml) at 0 °C. The mixture was then stirred for 5 min and alkyl bromide (**16**) (333 mg, 1.31 mmol, 1.0 equiv.) was engaged dropwise while dissolved in (1.25 + 1 mL) of anhydrous THF at 0 °C. The reaction mixture was bought to room temperature and stirred for 12 h. The reaction mixture was diluted with EtOAc (20 mL) and quenched with ice, extracted with EtOAc (10 mL × 4), dried over Na_2SO_4, and concentrated. The residue was purified by silica flash column chromatography (eluent: hexane → 15% EtOAc in hexane) to give title

compound (**17**) as a colorless syrup (393 mg, 1.28 mmol, 98%). R_f = 0.63 (1:5 hexane/EtOAc; UV, CAM) ^{1}H NMR (600 MHz, $CDCl_3$) δ 7.39 – 7.33 (m, 3H, ArH), 7.33 – 7.29 (m, 2H, ArH), 5.17 (s, 1H, H1'), 5.12 (d, *J* = 2.2 Hz, 1H, H1'), 4.57 – 4.53 (m, 2H, benzyl CH_2), 4.34 (d, *J* = 2.0 Hz, 2H, H1), 3.68 (td, *J* = 6.8, 1.7 Hz, 2H, H4), 2.47 (t, *J* = 6.5 Hz, 2H, H3), 1.51 – 1.47 (m, 9H, ^{t}Bu). ^{13}C NMR (151 MHz, $CDCl_3$) δ 156.5 (carbamate), 141.5 (C2), 138.3 (Ar), 128.4 (Ar), 127.7 (Ar), 127.6 (Ar), 116.9 (C1'), 81.5 (^{t}Bu quaternary C), 79.5 (C1), 73.0 (benzyl CH_2), 68.9 (C4), 33.5 (C3), 28.2 (^{t}Bu CH_3). HRMS-ESI (*m/z*): $[M + Na]^+$ calculated for $[C_{17}H_{25}NO_4Na]^+$: 330.1681, found: 330.1684.

***tert*-Butyl allyl(4-(benzyloxy)-2-methylenebutoxy)carbamate (18)**

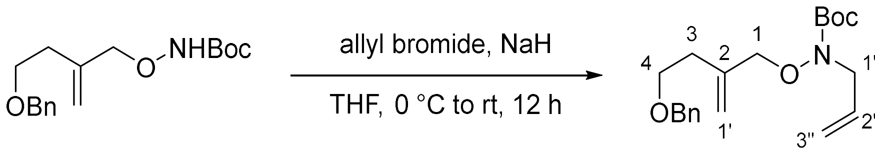

In an oven dried flask under an argon atmosphere, NaH (60 w% dispersion in mineral oil) (51 mg, 1.28 mmol, 1.0 equiv.) was added to a stirred solution of (**17**) (393 mg, 1.28 mmol, 1.0 equiv.) in anhydrous THF (5.25 mL) at 0 °C. The mixture was then stirred for 5 min and allyl bromide (110 µL, 1.28 mmol, 1.0 equiv.) was engaged dropwise while dissolved in (1.25 + 1 mL) of anhydrous THF at 0 °C. the reaction mixture bought to room temperature and stirred for 12 h. The reaction mixture was diluted with DCM (20 mL) and quenched with ice, extracted with DCM (10 mL × 4), dried over Na_2SO_4, and concentrated. The residue was purified by silica flash column chromatography (eluent: hexane → 15% EtOAc in hexane) to give titled compound (**18**) as a colorless syrup (435 mg, 1.24 mmol, 98%). R_f = 0.75 (3:2 hexane/EtOAc; UV, CAM). ^{1}H NMR (600 MHz, $CDCl_3$) δ 7.36 (dd, *J* = 6.2, 1.8 Hz, 4H, ArH), 7.33 – 7.29 (m, 1H, ArH), 5.89 (ttd, *J* = 11.8, 6.1, 1.6 Hz, 1H, H2''), 5.24 (dt, *J* = 17.1, 1.5 Hz, 1H, H3''), 5.21 – 5.15 (m, 2H, H1' and H3''), 5.07 (d, *J* = 2.3 Hz, 1H, H1'), 4.54 (d, *J* = 1.8 Hz, 2H, benzyl CH_2), 4.32 (s, 2H, H1), 4.05 (dd, *J* = 6.0, 1.8 Hz, 2H, H1''), 3.64 (td, *J* = 6.8, 1.8 Hz, 2H, H4), 2.49 (t, *J* = 6.8 Hz, 2H, H3), 1.51 (d, *J* = 1.8 Hz, 8H, ^{t}Bu). ^{13}C NMR (151 MHz, $CDCl_3$) δ 156.5 (carbamate), 141.4 (C2), 138.5 (Ar), 132.8 (C2''), 128.4 (2C, Ar), 127.6 (2C, Ar), 127.6 (Ar), 117.9 (C3''), 116.3 (C1'), 81.4 (^{t}Bu quaternary C), 78.3 (C1), 72.9 (benzyl CH_2), 68.7 (C4), 52.7 (C1''), 33.8 (C3), 28.3 (3C, ^{t}Bu CH_3). HRMS-ESI (*m/z*): $[M + Na]^+$ calculated for $[C_{20}H_{29}NO_4Na]^+$: 370.1994, found: 370.2004.

***tert*-Butyl 5-(2-(benzyloxy)ethyl)-3,6-dihydro-2*H*-1,2-oxazine-2-carboxylate (19)**

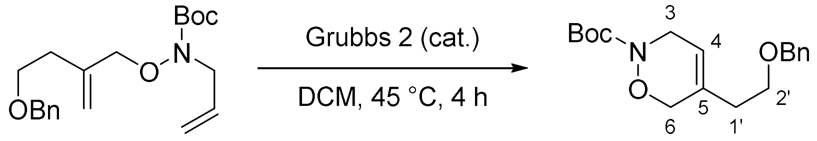

Hydroxylamine (**18**) (435 mg, 1.24 mmol, 1.0 equiv.) was dissolved in dry DCM (10 mL) and 2nd generation Grubbs catalyst (53 mg, 0.062 mmol, 0.05 equiv.) was engaged. The reaction mixture was stirred at 45 °C for 4 hours. Upon completion, the reaction mixture was cooled and filtered through a celite pad and concentrated under reduced pressure. The residue was purified by flash silica column chromatography (eluent: hexane → 20% EtOAc in hexane) to provide cyclized 1,2-oxacene product (**19**) as a colorless oil (388 mg, 1.216 mmol, 98%). R_f = 0.51 (20% EtOAc/hexane, UV, CAM) ^{1}H NMR (600 MHz, $CDCl_3$) δ 7.40 – 7.27 (m, 5H, ArH), 5.63 – 5.59 (m, 1H, H4), 4.53 (d, *J* = 2.0 Hz, 2H, benzyl CH_2), 4.37 (s, 2H, H6), 4.08 (br s, 2H, H3), 3.57 (td, *J* = 6.5, 1.8 Hz, 2H, H2'), 2.31 (t, *J* = 6.7 Hz, 2H, H1'), 1.52 (d, *J* = 1.9 Hz, 9H, ^{t}Bu). ^{13}C NMR (151 MHz, $CDCl_3$) δ 155.1 (carbamate), 138.1 (Ar), 133.6 (C5), 128.4 (2C, Ar), 127.7 (2C, Ar), 127.6 (Ar), 117.6 (C4), 81.5 (^{t}Bu quaternary C), 73.1 (benzyl CH_2), 70.7 (C6), 68.67 (C2'), 44.9 (C3), 33.3 (C1'), 28.3 (3C, ^{t}Bu CH_3). HRMS-ESI (*m/z*): [M + Na]$^+$ calculated for $[C_{18}H_{25}NO_4Na]^+$: 342.1681, found: 342.1665.

***tert*-Butyl 5-(2-hydroxyethyl)-1,2-oxazinane-2-carboxylate (20)**

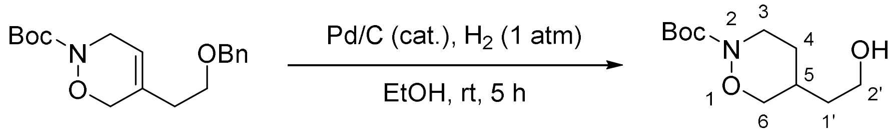

Palladium on carbon (50.8 mg, 0.48 mmol, 0.11 equiv.) was engaged at room temperature with a solution of 1,2-oxazine (**19**) (1.39 g, 4.34 mmol, 1.0 equiv.) in EtOH (96 mL). reaction mixture stirred at room temperature for 5 hours, and upon completion was filtered through a celite pad and concentrated under reduced pressure, The residue was purified by flash silica column chromatography (eluent: hexane → 40% EtOAc in hexane) to provide the reduced product (**20**) as a colorless oil (927 mg, 4.01 mmol, 92%). R_f = 0.12 (20% EtOAc/hexane; $KMnO_4$) ^{1}H NMR (600 MHz, $CDCl_3$) δ 4.08 – 3.98 (m, 2H, H3 and H6), 3.78 – 3.68 (m, 2H, H2'), 3.58 (dd, *J* = 11.4, 10.2 Hz, 1H, H6), 3.33 (ddd, *J* = 13.4, 11.4, 3.2 Hz, 1H, H3), 2.11 – 2.00 (m, 1H, H5), 1.88 – 1.80

(m, 1H, H1'), 1.52 (s, 11H, ^{t}Bu and H4), 1.39 (dtd, *J* = 13.3, 11.1, 4.3 Hz, 1H, H1'). ^{13}C NMR (151 MHz, $CDCl_3$) δ 155.1 (carbamate), 81.4 (^{t}Bu quaternary C), 75.7 (C6), 60.6 (C2'), 45.8 (C3), 34.6 (C4), 31.5 (C5), 29.1 (C1'), 28.3 (3C, ^{t}Bu CH_3). HRMS ESI (*m/z*): $[M + Na]^+$ calculated for $[C_{11}H_{21}NO_4Na]^+$: 254.1362, found:254.1354.

***tert*-Butyl 5-(2-azidoethyl)-1,2-oxazinane-2-carboxylate (21)**

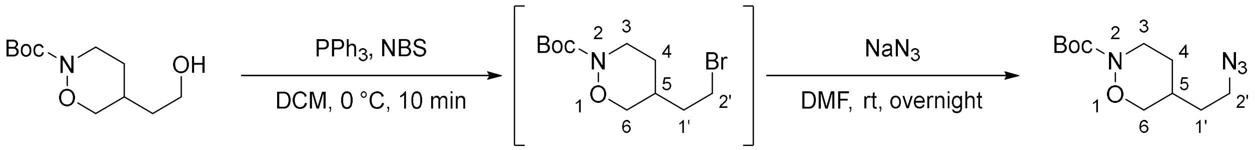

In an oven-dried flask under an argon atmosphere, to a solution of (**20**) (80 mg, 0.34 mmol, 1.0 equiv.) in DCM (3.4 mL) was added triphenylphosphine (181 mg, 0.69 mmol, 2 equiv.) and *N*-bromosuccinimide (123 mg, 0.69 mmol, 2 equiv.) at 0 °C stirred for 10 min. Upon completion, the reaction mixture was diluted with CH_2Cl_2 (20 mL) and quenched with sat. $NaHCO_3$ (20 mL), and the aqueous layer was extracted with CH_2Cl_2 (5 × 10 mL). The combined organic layers were dried over Na_2SO_4 and concentrated under reduced pressure. The green residue was purified by flash column chromatography (eluent: hexane → 20% EtOAc/hexane) to provide to provide *tert*-butyl 5-(2-bromoethyl)-1,2-oxazinane-2-carboxylate (102 mg) as a light-yellow liquid. R_f = 0.7 (20% EtOAc/Hexane; CAM). ^{1}H NMR (600 MHz, $CDCl_3$) δ 4.00 (ddd, *J* = 11.5, 4.5, 1.5 Hz, 1H, H6), 3.96 (dt, *J* = 13.6, 4.1 Hz, 1H, H3), 3.55 (dd, *J* = 11.4, 9.8 Hz, 1H, H6), 3.46 – 3.37 (m, 2H, H2'), 3.33 (ddd, *J* = 13.9, 11.0, 3.2 Hz, 1H, H3), 2.10 (dddt, *J* = 14.5, 10.6, 6.5, 3.7 Hz, 1H, H5), 1.87 – 1.79 (m, 2H, H4 and H1'), 1.76 (dq, *J* = 14.1, 6.9 Hz, 1H, H4), 1.49 (s, 9H, ^{t}Bu), 1.34 (dtd, *J* = 13.2, 10.7, 4.4 Hz, 1H, H1'). ^{13}C NMR (151 MHz, $CDCl_3$) δ 155.0 (carbamate), 81.5 (^{t}Bu quaternary C), 74.8 (C6), 45.5 (C3), 34.4 (C4), 32.9 (C5), 30.1 (C2'), 28.31 (C1'), 28.29(3C, ^{t}Bu CH_3).

Under an argon atmosphere, a stirred solution of *tert*-butyl 5-(2-bromoethyl)-1,2-oxazinane-2-carboxylate (102 mg, 0.34 mmol, 1 equiv.) in DMF (3.4 mL) was engaged with sodium azide (44 mg, 0.68 mmol, 2 equiv.) at room temperature and stirred overnight. Upon completion reaction mixture diluted with DCM and quenched with 10 mL of ice water, aqueous layer extracted into DCM (10 mL × 4) and combined organic phases washed with 0.1 M $KHSO_4$ and dried over Na_2SO_4 and concentrated under reduced pressure. Crude residue separated over silica flash chromatography (eluent: hexane to 20% EtOAc/hexane) to obtain the title compound (**21**) as a

colorless oil (65 mg, 0.25 mmol, 74% over 2 steps). R_f = 0.35 (20% EtOAc/hexane; CAM). ^{1}H NMR (600 MHz, $CDCl_3$) δ 4.02 – 3.93 (m, 2H, H3 and H6), 3.53 (dd, *J* = 11.4, 10.0 Hz, 1H, H6), 3.38 – 3.27 (m, 3H, H3 and H2'), 1.96 (tdq, *J* = 10.8, 6.9, 3.6 Hz, 1H, H5), 1.84 – 1.77 (m, 1H, H1'), 1.55 (dq, *J* = 13.9, 6.9 Hz, 1H, H4), 1.49 (s, 9H, ^{t}Bu), 1.48 – 1.43 (m, 1H, H4), 1.39 – 1.30 (m, 1H, H1'). ^{13}C NMR (151 MHz, $CDCl_3$) δ 155.0 (carbamate), 81.4 (^{t}Bu quaternary C), 75.1 (C6), 48.6 (C2'), 45.6 (C3), 32.0 (C5), 30.7 (C1'), 28.7 (C4), 28.3 (3C, ^{t}Bu CH_3). HRMS-ESI (*m/z*): [M + Na]$^+$ calculated for $[C_{11}H_{20}N_4O_3Na]^+$: 279.1433, found: 279.1445.

5-(2-Azidoethyl)-2-methyl-1,2-oxazinane (22)

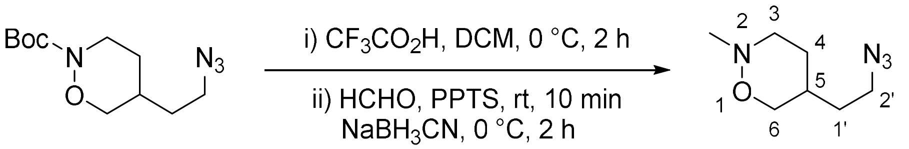

Under an argon atmosphere, a stirred solution of (**21**) (100 mg, 0.39 mmol, 1 equiv.) in DCM (2 mL) was engaged with trifluoracetic acid (150 mL, 1.95 mmol, 5 equiv.) at 0 °C and stirred for 2 h, at 0 °C. Upon completion reaction mixture diluted with DCM and quenched with 10 mL of saturated Na_2CO_3 solution, aqueous layer extracted into DCM (10 mL × 4) and combined organic phases dried over Na_2SO_4 and concentrated under reduced pressure. Crude residue used in next reaction. A 37% solution of formaldehyde (32 mL, 0.43 mmol, 1.1 equiv.) was engaged into a stirred solution of crude product and pyridinium *p*-toluenesulfonate (980 mg, 3.9 mmol, 10 equiv.) in dry methanol (3.9 mL) at room temperature and the mixture was stirred for 10 min. The mixture was then cooled to 0 °C and sodium cyanoborohydride (49 mg, 0.78 mmol, 2 equiv.) was added to the reaction mixture and stirring at 0 °C for 2 h. Upon completion the reaction mixture was diluted with EtOAc, washed periodically with water, saturated $NaHCO_3$ solution and brine and dried over Na_2SO_4 and concentrated under reduced pressure. The product was filtered through a silica plug with 30% EtOAc/hexane to give the active filtrate of crude compound (**22**) as a colorless oil (51 mg). R_f = 0.33 (20% EtOAc/hexane; CAM). HRMS-ESI (*m/z*): [M + H]$^+$ calculated for $[C_7H_{15}N_4O]^+$:171.1246, found: 171.1229.

2-(2-Methyl-1,2-oxazinan-5-yl)ethan-1-amine (23)

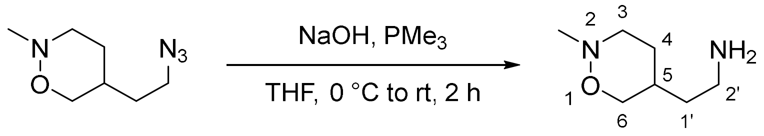

Under an argon atmosphere, a stirred solution of (**22**) (51 mg, 0.30 mmol, 1 equiv.) in dry THF (1.2 mL) was added NaOH (1 M) (0.93 mL, 0.93 mmol, 3.10 equiv.) and then was added trimethyl phosphine (610 mL, 0.61 mmol, 2 equiv.) at 0 °C and stirred for 1 h, at 0 °C and bought to room temperature over 1 h. Upon completion reaction mixture diluted with DCM and added 20 mL DI water, aqueous layer extracted into DCM (20 mL × 4) and combined organic phases dried over Na_2SO_4 and concentrated under reduced pressure at 15 °C. The residue was filtered through a neutral alumina plug with 10% MeOH/DCM to give the active filtrate of crude (**23**) (32 mg) as a colorless oil. R_f = 0.1 (10% MeOH/DCM; ninhydrin). HRMS-ESI (*m/z*): $[M + H]^+$ calculated for $[C_7H_{15}N_2O]^+$: 145.1351, found: 145.1341.

6-(2-Chloro-4-(6-methylpyrazin-2-yl)phenyl)-8-ethyl-2-((2-(2-methyl-1,2-oxazinan-5-yl)ethyl)amino)pyrido[2,3-*d*]pyrimidin-7(8*H*)-one (8)

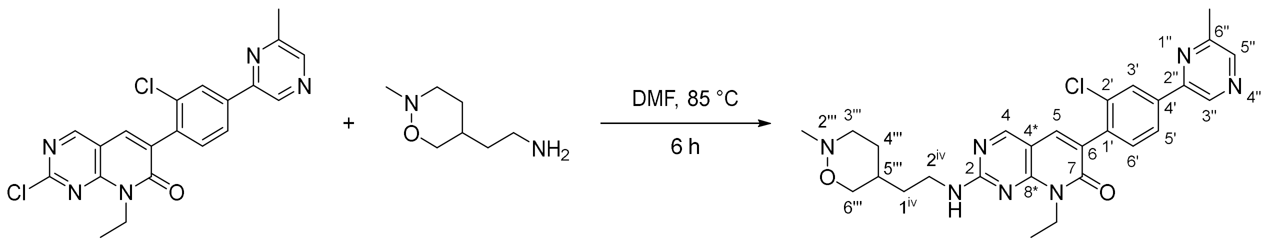

In a flame-dried flask under an argon atmosphere, to a solution of (**14**) (45 mg, 0.11 mmol, 1.0 equiv.) dissolved in anhydrous DMF (0.5 mL) was added (**23**) (32 mg, 0.22 mmol, 2.0 equiv., in 0.5 mL anhydrous DMF). The reaction mixture stirred at 85 °C for 6 h, until all starting material was shown to be consumed by TLC and MS. The organics were washed with 5% NaOH (10 mL × 3), brine (10 mL × 2), dried over Na_2SO_4, concentrated under reduced pressure, and separated on a neutral alumina flash column (eluent: DCM to 60% DCM/MeOH) to yield (**8**) as pale yellow crystalline solid (19 mg, 0.036 mmol, 19%, 3 steps). R_f = 0.55 (10% MeOH/DCM, UV, CAM), ^{1}H NMR (600 MHz, $CDCl_3$) δ 8.85 (s, 1H, H5), 8.48 (s, 1H, H4), 8.45 (s, 1H, H3''), 8.19 (d, *J* = 1.8 Hz, 1H, H3'), 7.96 (dd, *J* = 8.0, 1.8 Hz, 1H, H6'), 7.59 (s, 1H, H5''), 7.54 (d, *J* = 7.9 Hz, 1H, H5'),

5.62 (br s, 1H, NH), 4.51 (s, 2H, CH_2), 4.05 – 4.00 (m, 1H, H6’’’), 3.67 – 3.52 (m, 3H, H2^{iv}, and H6’’’), 3.00 (br s, 1H, H3’’’), 2.66 (s, 3H, CH_3), 2.63 (s, 3H, CH_3), 2.60 – 2.52 (m, 1H, H3’’’), 1.94 (br s, 1H, H4’’’), 1.78 – 1.74 (m, 1H, H5’’’), 1.68 – 1.50 (m, 3H, H1^{iv} and H4’’’), 1.38 (s, 3H, CH_3). ^{13}C NMR (151 MHz, $CDCl_3$) δ 161.7 (C=O), 161.6, 158.8, 155.4, 153.5, 150.0, 143.3, 138.9, 137.8, 136.6, 135.8, 134.7, 134.1, 132.3, 128.2, 126.2, 124.9 (aromatic), 74.5 (C6’’’), 57.7 (C3’’’), 46.6 (CH_3), 39.2 (C1^{iv}), 36.5 (CH_2), 32.4 (C5’’’), 30.6 (C2^{iv}), 29.7 (C4’’’), 21.8 (CH_3), 13.0 (CH_3). HRMS-ESI (*m/z*): $[M + H]^+$ calculated for $[C_{27}H_{31}ClN_7O_2]^+$: 520.2228, found: 520.2230.

1-(2-Azidoethoxy)-4-methylpiperazine (25)

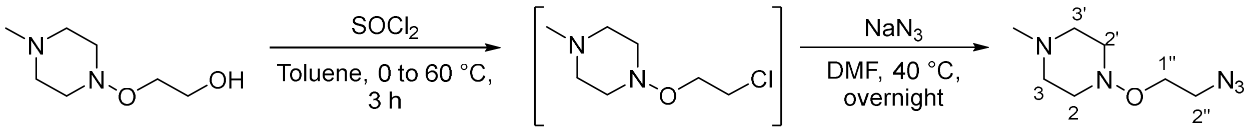

Under an argon atmosphere, to a stirred solution of (**24**) (850 mg, 5.31 mmol, 1.0 equiv.) in anhydrous toluene (22 mL) at 0 °C was added $SOCl_2$ (0.96 mL, 13.26 mmol, 2.5 equiv.) dropwise. The reaction mixture was stirred until it was no longer exothermic and then warmed to 60 °C and stirred for 3 h. After this time, the reaction mixture was concentrated under reduced pressure. The crude reaction mixture was diluted in EtOAc (100 mL), washed with sat. aqueous K_2CO_3 (50 mL × 2), dried over Na_2SO_4, filtered, and concentrated under reduced pressure. The resulting crude (390mg) product was used directly in the next step without further purification.

To a stirred solution of the above crude (230 mg, 1.29 mmol, 1 equiv.) in DMF (11 mL) was added sodium azide (209 mg, 3.21 mmol, 2.5 equiv.) at room temperature and then the reaction mixture was warmed up to 40 °C and stirred overnight. After such time, the reaction mixture was diluted with EtOAc (50 mL), washed with aq. $NaHCO_3$ (25 mL), brine (25 mL), dried over Na_2SO_4, filtered, and concentrated under reduced pressure. The resulting residue was purified by silica gel flash chromatography (eluent: 5% MeOH in DCM) to afford the title compound (**25**) (178 mg, 31% over 2 steps) as a light brown oil. TLC R_f = 0.40 (7.5:92.5 MeOH/DCM; CAM). ^{1}H NMR (600 MHz, Toluene-D_8) δ 3.48 (t, J = 5.1 Hz, 2H, H1’’), 2.99 (d, J = 10.7 Hz, 2H, H2), 2.84 (t, J = 5.1 Hz, 2H, H2’’), 2.71 (t, J = 10.6 Hz, 2H, H3), 2.43 – 2.40 (m, 2H, H2’), 2.02 – 1.99 (m, 2H, H3’), 1.98 (s, 3H, CH_3); ^{13}C NMR (151 MHz, Toluene-D_8) δ 69.8 (C1’’), 55.3 (C2), 54.2 (C3), 49.7 (C2’’), 45.3 (CH_3). HRMS-ESI (*m/z*): $[M + H]^+$ calculated for $[C_7H_{16}N_5O]^+$: 186.1349, found: 186.1349.

2-((4-Methylpiperazin-1-yl)oxy)ethan-1-amine (26)

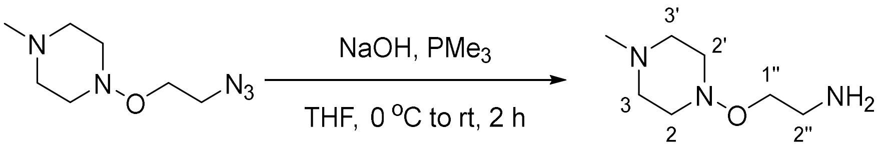

Under an argon atmosphere, to a stirred solution of (**25**) (80 mg, 0.432 mmol, 1 equiv.) in anhydrous THF (1.7 mL) was added NaOH (1 M) (1.34 mL, 1.34 mmol, 3.10 equiv.) at 0 ºC. The reaction mixture was stirred for 5 min, then was treated at 0 ºC dropwise with trimethyl phosphine (1 M in THF) (0.86 mL, 0.86 mmol, 2.0 equiv.), and then allowed to warm to room temperature and stirred for 2 h. Upon complete conversion, the reaction mixture was cooled to 0 ºC, acidified to pH ≈ 2 by adding 4 M HCl dropwise, and washed with Et_2O (20 mL × 3), the aqueous phase was basified to pH ≈ 10 by dropwise addition of 4 M KOH, and extracted with 3:1 mixture of chloroform: isopropanol (30 mL × 4). The extracts were dried over Na_2SO_4, filtered, and concentrated under reduced pressure at 25 ºC. The crude residue was purified by neutral alumina flash column chromatography (eluent: 10 % MeOH in DCM) to afford the title compound (**26**) (45 mg, 66%) as a yellow oil. TLC (N-Alumina) R_f = 0.20 (10:90 MeOH/DCM; Ninhydrin). ^{1}H NMR (900 MHz, Toluene-D_8) δ 3.57 (t, *J* = 5.3 Hz, 2H, H1'), 3.01 (d, *J* = 11.2 Hz, 2H, H2), 2.71 (t, *J* = 11.0 Hz, 2H, H3), 2.67 (t, *J* = 5.6 Hz, 2H, H2''), 2.45 (d, *J* = 11.2 Hz, 2H, H2'), 2.05 (t, *J* = 11.0 Hz, 2H, H3'), 2.02 (s, 3H, CH_3), 1.00 (s, 2H, NH_2); ^{13}C NMR (226 MHz, Toluene-D_8) δ 74.2 (C1'), 55.8 (C2), 54.5 (C3), 45.5 (C2''), 41.8 (CH_3). HRMS-ESI (*m/z*): $[M + H]^+$ calculated for $[C_7H_{18}N_3O]^+$: 160.1444, found: 160.1444.

6-(2-Chloro-4-(6-methylpyrazin-2-yl)phenyl)-8-ethyl-2-((2-((4-methylpiperazin-1-yl)oxy)ethyl)amino)pyrido[2,3-*d*]pyrimidin-7(8*H*)-one) (9)

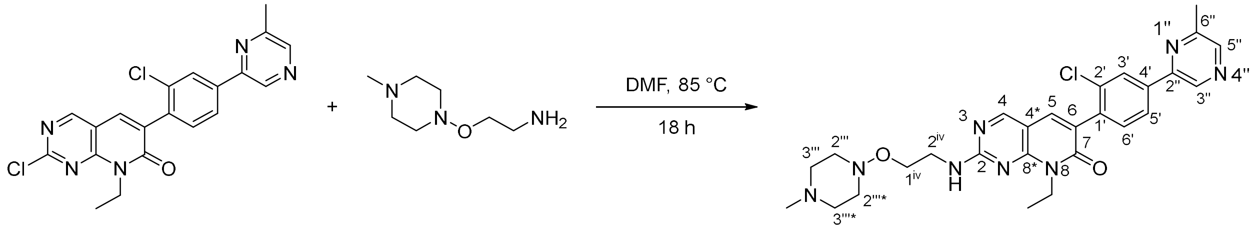

In an oven dried flask, under argon atmosphere, a solution of (**14**) (55 mg, 0.133 mmol, 1.0 equiv.) in DMF (1.65 mL) was treated with (**26**) (42 mg, 0.267 mmol, 2.0 equiv.) at room temperature and then brought to 85 ºC and stirred for 18 h. After such time, the reaction mixture was diluted with EtOAc (50 mL) and washed with 5% aqueous NaOH (20 mL × 2), brine (20 mL), dried over

Na_2SO_4, filtered, and concentrated under reduced pressure. The resulting crude residue was purified by neutral alumina flash column (eluent: 3 % MeOH in DCM) to afford the title compound (**9**) as light-yellow solid (53 mg, 68%). TLC (N-Alumina) R_f = 0.50 (10:90 MeOH/DCM; UV). ^1H NMR (500 MHz, $CDCl_3$) δ 8.83 (s, 1H, H5), 8.48 (s, 1H, H4), 8.42 (s, 1H, H3'), 8.16 (s, 1H, H5''), 7.93 (d, J = 8.0 Hz, 1H, H5'), 7.57 (s, 1H, H3''), 7.51 (d, J = 8.0 Hz, 1H, H6'), 6.00 (br s, 1H, NH), 4.48 (s, 2H, CH_2), 3.93 (t, J = 5.1 Hz, 2H, $H1^{iv}$), 3.74 (t, J = 5.3 Hz, 2H, $H2^{iv}$), 3.34 (s, 2H, H2'''*), 2.97 (br s, 4H, H2''', H3'''*), 2.64 (s, 3H, CH_3), 2.57 – 2.32 (m, 5H, H3''', CH_3), 1.35 (t, J = 7.0 Hz, 3H, CH_3); ^{13}C NMR (151 MHz, $CDCl_3$) δ 161.9 (C=O), 161.7, 158.9, 155.7, 153.6, 150.2, 143.4, 139.0, 138.1, 136.7, 135.9, 134.9 132.4, 128.4, 125.0 (aromatic), 70.1 ($C1^{iv}$), 53.6 (CH_3), 44.7 ($C2^{iv}$), 41.7 (C2''', C3'''), 36.6 (CH_2), 21.8 (CH_3), 13.1 (CH_3). HRMS-ESI (m/z): $[M + H]^+$ calculated for $[C_{27}H_{32}N_8O_2{}^{35}Cl]^+$: 535.2331, found: 535.2332.

***tert*-Butyl 4-methoxypiperazine-1-carboxylate (28)**

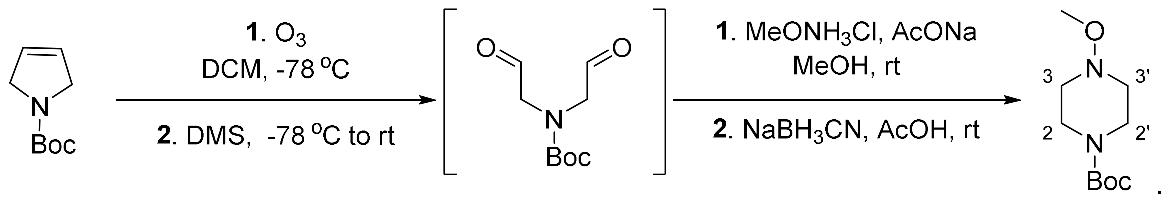

Ozone was bubbled at -78 °C into a solution of *tert*-butyl 2,5-dihydro-1*H*-pyrrole-1-carboxylate (**27**) (3.39 g, 20.0 mmol, 1.0 equiv.) in DCM (60 mL) in an oven dried flask. for 0.5 h until the reaction turned blue. The excess ozone was purged off with a flow of nitrogen until the reaction mixture turned colorless and dimethyl sulfide (3.67 mL, 50 mmol, 2.5 equiv.) was added at -78 °C, and the reaction was allowed to stir and warm to rt over 1 h. The reaction mixture was concentrated under reduced pressure to give the crude dialdehyde (3.31 g), which was used in the next step without further purification.

Methoxylamine hydrochloride (4.18 g, 50.0 mmol) and sodium acetate (4.10 g, 50.0 mmol) were dissolved in methanol (70 mL) and stirred at rt for 0.5 h before the above crude dialdehyde in methanol (30 mL) was added to the reaction mixture. After stirring for 4 h, sodium cyanoborohydride (6.28 g, 100 mmol, 5.0 equiv.) and glacial acetic acid (11.4 mL, 200 mmol, 10.0 equiv.) were slowly added, and the reaction mixture was stirred at rt for another 4 h. The mixture was then concentrated by rotary evaporator, diluted with 50 mL ethyl acetate, and washed with saturated aqueous $NaHCO_3$ (100 mL × 2) and brine. The organic layer was dried over Na_2SO_4, filtered, and concentrated under reduced pressure. The resulting residue was purified by silica flash chromatography (eluent: 10-30% EtOAc in hexane) to afford the title compound (**28**) (2.80 g, 65% over 2 steps) as colorless oil. TLC R_f= 0.58 (25:75 EtOAc/Hexane; CAM). ^1H NMR (500

MHz, $CDCl_3$) δ 4.09 - 3.80 (m, 2H, H3), 3.54 (s, 3H, CH_3), 3.28 - 3.12 (m, 2H, H2'), 3.10 - 2.90 (m, 2H, H3'), 2.55 - 2.39 (m, 2H, H2), 1.45 (s, 9H, ($C(CH_3)_3$); ^{13}C NMR (126 MHz, Benzene-D_6) δ 154.4 (C=O), 79.3 (quat), 58.8 (CH_3), 54.9 (C2), 42.9 (C3), 28.4 ($C(CH_3)_3$). HRMS-ESI (*m/z*): [M + Na]$^+$ calculated for $[C_{10}H_{20}N_2O_3Na]^+$: 239.1366, found: 239.1363.

1-Methoxypiperazine bis(trifluoroacetic acid) salt (29)

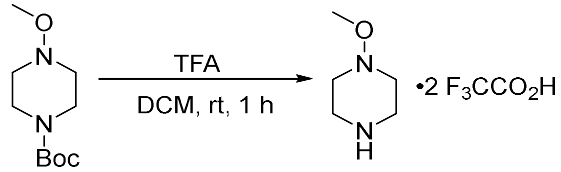

tert-Butyl 4-methoxypiperizine-1-carboxylate (**28**) (2.60 g, 12.0 mmol, 1.0 equiv.) was dissolved in DCM (10 mL) and trifluoroacetic acid (10 mL, 131 mmol, 10.9 equiv.) was added at rt. The reaction mixture was allowed to stir for 1 h and then was concentrated under reduced pressure. The residue was triturated with 5 mL diethyl ether, then evaporated under vacuum to afford the title compound (**29**) (3.85 g) as pale-white solid, which was used in the next step without further purification.

3-(4-Methoxypiperazin-1-yl)propan-1-ol (30)

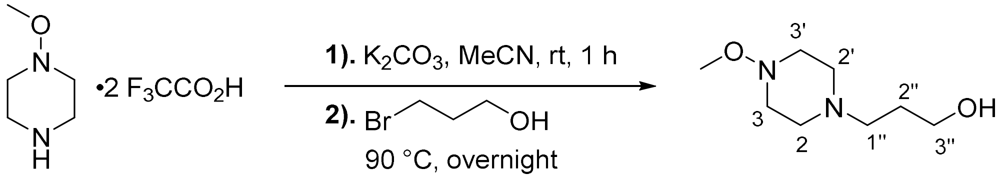

1-Methoxypiperazine bis(trifluoroacetic acid) salt (**29**) (3.85 g, 11.2 mmol, 1.0 equiv.) and potassium carbonate (10.0 g, 72.6 mmol, 6.5 equiv.) were dissolved in acetonitrile (100 mL). 3-Bromopropan-1-ol (1.5 mL, 16.8 mmol, 1.5 equiv.) was added and the mixture was stirred at rt for 1 h, then heated to 90 °C overnight. After such time, the reaction mixture was concentrated under reduced pressure, then diluted with water (90 mL) and extracted with EtOAc (60 mL × 3), and the combined organic layer was washed with brine (100 mL), dried over Na_2SO_4, filtered, and concentrated under reduced pressure. The resulting residue was purified by silica gel flash chromatography (eluent: 25% MeOH in DCM) to afford the title compound (**30**) (1.61 g, 77% over two steps) as a colorless oil. TLC R_f = 0.43 (40:60 MeOH/DCM; CAM). ^1H NMR (500 MHz, $CDCl_3$) δ 3.77 (t, *J* = 5.3 Hz, 2H, H3''), 3.51 (s, 3H, CH_3), 3.27 - 3.18 (m, 2H, H3), 3.02 – 2.90 (m, 2H, H2), 2.67 – 2.60 (m, 2H, H3'), 2.59 (t, *J* = 5.8 Hz, 2H, H1') 2.28 – 2.17 (m, 2H, H2'), 1.69 (p, *J* =

5.5 Hz, 2H, H2''); ^{13}C NMR (126 MHz, $CDCl_3$) δ 64.6 (C3''), 59.2 (CH_3), 58.3 (C2), 54.8 (C1'), 52.0 (C3), 27.5 (C2''). HRMS-ESI (*m/z*): $[M + H]^+$ calculated for $[C_8H_{19}N_2O_2]^+$: 175.1441, found: 175.1438.

1-(3-Azidopropyl)-4-methoxypiperazine (31)

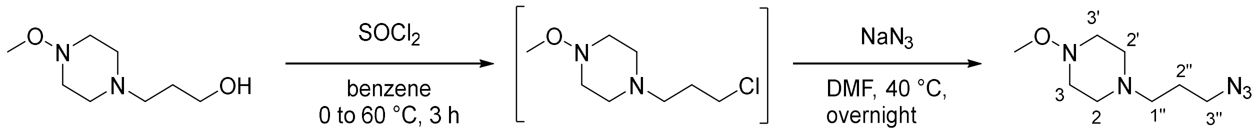

In an oven dried flask, under argon atmosphere, to a stirred solution of (**30)** (790 mg, 4.53 mmol, 1.0 equiv.) in benzene (18.8 mL) was added $SOCl_2$ (0.986 mL, 13.6 mmol, 2.5 equiv.) dropwise at 0 °C. The reaction mixture was stirred until no longer exothermic and then warmed to 60 °C and stirred for 3 h. After such time, the reaction mixture was concentrated under reduced pressure and the residue dissolved in EtOAc (80 mL), washed with sat. aqueous Na_2CO_3 (50 mL × 2), dried over Na_2SO_4, filtered, and concentrated under reduced pressure. The resulting crude residue (770 mg) was used directly in the next step without further purification.

Under an argon atmosphere, the above crude (770 mg, 4.0 mmol, 1 equiv.) was diluted in dry DMF (20 mL), sodium azide (520 mg, 7.99 mmol, 2.0 equiv.) was added at room temperature, and the reaction mixture was warmed up to 40 °C and stirred overnight. After such time, the reaction mixture was diluted with Et_2O (100 mL) and washed with aq. $NaHCO_3$ (50 mL), brine (50 mL), dried over Na_2SO_4, filtered, and concentrated under reduced pressure. The resulting crude residue was separated over silica flash chromatography (eluent: 5% MeOH in DCM) to afford the title compound (**31**) (605 mg, 67% over 2 steps) as colorless oil. TLC R_f = 0.30 (5:95 MeOH/DCM; CAM). ^1H NMR (500 MHz, Toluene-D_8) ^1H NMR (500 MHz, Toluene-d8) δ 3.43 (s, 3H, CH_3), 3.07 (d, *J* = 10.0 Hz, 2H, H3), 2.81 (t, *J* = 6.8 Hz, 2H, H1'), 2.61 (t, *J* = 10.8 Hz, 2H, H2), 2.41 (d, *J* = 11.0 Hz, 2H, H3'), 1.98 (t, *J* = 6.8 Hz, 2H, H3''), 1.93 (t, *J* = 10.4 Hz, 2H, H2'), 1.27 (p, *J* = 6.8 Hz, 2H, H2'').; ^{13}C NMR (126 MHz, Toluene-D_8) δ 58.7 (CH_3), 55.2 (C2), 54.5 (C3), 52.1 (C3''), 49.3 (C1'), 26.6 (C2''). HRMS-ESI (*m/z*): $[M + H]^+$ calculated for $[C_8H_{18}N_5O]^+$: 200.1505, found: 200.1503.

3-(4-Methoxypiperazin-1-yl)propan-1-amine (32)

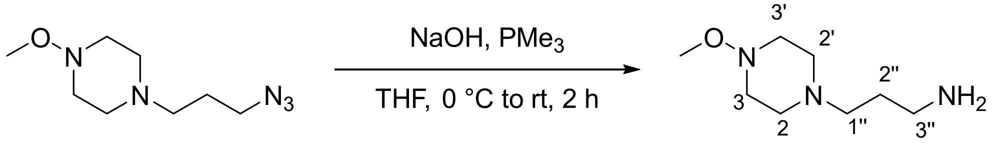

NaOH (1 M) (8.25 mL, 8.25 mmol, 3.10 equiv.) at 0 ºC under an argon atmosphere was added to a stirred solution of (**31**) (530 mg, 2.66 mmol, 1 equiv.) in THF (10.6 mL). After 5 min trimethyl phosphine (1 M in THF) (5.32 mL, 5.32 mmol, 2.0 equiv.) was added dropwise, and then the reaction mixture was brought to room temperature and stirred for 2 h. After such time, the reaction mixture was cooled to 0 ºC and acidified to pH ≈ 2 by dropwise addition of 4 M HCl and washed with Et_2O (50 mL × 3). The aqueous phase was basified to pH ≈ 10 by dropwise addition of 4 M KOH and was extracted with 3:1 mixed solvent of chloroform and isopropanol (50 mL × 4), dried over Na_2SO_4, filtered, and concentrated under reduced pressure at 25 ºC. The crude residue was purified by neutral alumina flash column chromatography (10 % MeOH in DCM) to access the title compound (**32**) (374 mg, 81%) as a colorless oil TLC (N-Alumina) R_f = 0.30 (10:90 MeOH/DCM; Ninhydrin). ^{1}H NMR (500 MHz, $CDCl_3$) δ 3.48 (s, 3H, CH_3), 3.19 (d, *J* = 10.0 Hz, 2H, H3), 2.81 (d, *J* = 11.4 Hz, 2H, H3'), 2.71 (t, *J* = 6.9 Hz, 2H, H1'), 2.59 (t, *J* = 11.1 Hz, 2H, H2), 2.35 (t, *J* = 7.3 Hz, 2H, H3''), 2.16 (t, *J* = 11.2 Hz, 2H, H2'), 1.98 (s, 2H, NH_2), 1.58 (p, *J* = 7.0 Hz, 2H, H2''); ^{13}C NMR (126 MHz $CDCl_3$) δ 59.0 (CH_3), 55.9 (C3''), 54.9 (C2), 52.1 (C3), 40.8(C1'), 30.5 (C2''). HRMS-ESI (*m/z*): $[M + H]^+$ calculated for $[C_8H_{20}N_3O]^+$: 174.1600, found: 174.1599.

6-(2-Chloro-4-(6-methylpyrazin-2-yl)phenyl)-8-ethyl-2-((3-(4-methoxypiperazin-1-yl)propyl)amino)pyrido[2,3-*d*]pyrimidin-7(8*H*)-one (10)

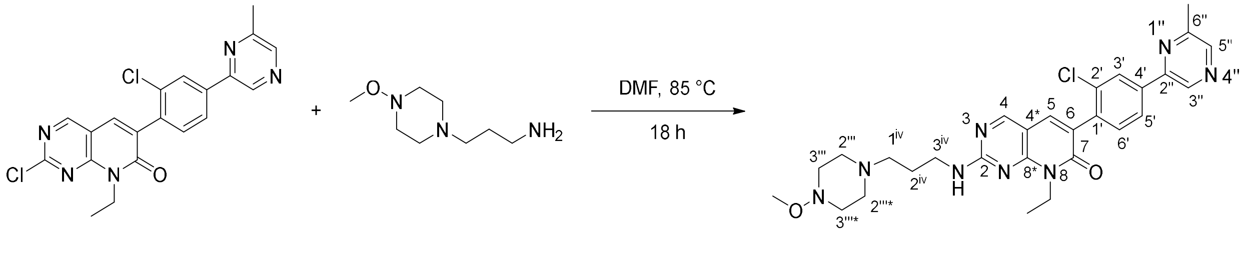

Under an argon atmosphere, (**14**) (250 mg, 0.606 mmol, 1.0 equiv.) was dissolved in dry DMF (7.5 mL), stirred and treated with (**32**) (120 mg, 1.21 mmol, 2.0 equiv.) at room temperature. The reaction mixture was then heated to 85 ºC with stirring for 18 h, then cooled to room temperature,

diluted with Et_2O (50 mL), and washed with 5% aqueous NaOH (30 mL × 2), and brine (30 mL). The combined organic layer was dried over Na_2SO_4, filtered, and concentrated under reduced pressure. The resulting residue was purified by neutral alumina flash column (eluent: 3 % MeOH in DCM) to afford the desired compound (**10**) as a yellow solid (237 mg, 71%). TLC (N-Alumina) R_f = 0.45 (10:90 MeOH/DCM; UV). ^{1}H NMR (500 MHz, $CDCl_3$) δ 8.82 (s, 1H, H5), 8.44 (s, 1H, H4), 8.41 (s, 1H, H3'), 8.16 (d, *J* = 1.5 Hz, 1H, H5''), 7.95 – 7.88 (m, 1H, H5'), 7.56 (s, 1H, H3''), 7.51 (d, *J* = 8.0 Hz, 1H, H6'), 4.48 (s, 2H, CH_2), 3.60 (q, *J* = 6.2 Hz, 2H, $H3^{iv}$), 3.53 (s, 3H, CH_3), 3.28 (s, 2H, H2'''*), 2.92 (s, 2H, H3'''*), 2.75 (s, 2H, H2'''), 2.64 (s, 3H, CH_3), 2.54 (s, 2H, $H1^{iv}$), 2.29 (s, 2H, H3'''), 1.88 – 1.82 (m, 2H, $H2^{iv}$), 1.34 (t, *J* = 7.5 Hz 3H, CH_3); ^{13}C NMR (126 MHz, $CDCl_3$) δ 161.8, 161.8, 158.9, 155.5, 153.6, 150.1, 143.4, 139.0, 137.8, 136.8, 136.0, 134.9, 132.4, 128.3, 125.0 (aromatic), 70.7 (C3'''), 59.2 (CH_3), 56.2 ($C1^{iv}$), 54.4 (C2'''), 40.6 ($C3^{iv}$), 36.5 (CH_2), 25.8 ($C2^{iv}$), 21.9 (CH_3), 13.1(CH_3). HRMS-ESI (*m/z*): $[M + H]^+$ calculated for $[C_{28}H_{34}N_8O_2{}^{35}Cl]^+$: 549.2487, found: 549.2488.

2-(2-(4-Methoxypiperazin-1-yl)ethyl)isoindoline-1,3-dione (34)

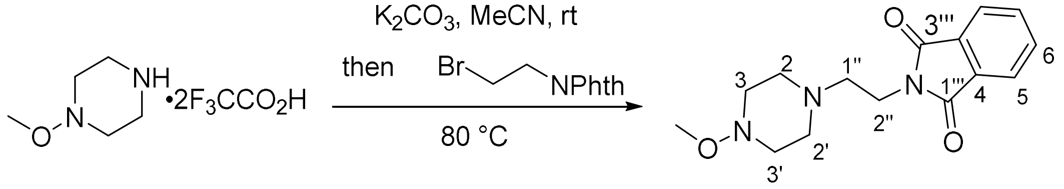

1-Methoxypiperazine di-trifluoroacetic acid salt (**29**) (784 mg, 2.28 mmol, 1.0 equiv.) and potassium carbonate (2.07 g, 15.0 mmol, 6.5 equiv.) was dissolved in acetonitrile (20 mL). The mixture was stirred at rt for 1 h following addition of 2-(2-bromoethyl)isoindoline-1,3-dione (**33**) (608 mg, 2.40 mmol, 1.05 equiv.). The reaction was heated to 80 °C overnight. The solvent was removed by evaporation, then diluted by water (50 mL). The mixture was extracted by ethyl acetate (30 mL × 3), and the combined organic layer was washed with brine (500 mL), dried by anhydrous Na_2SO_4, and evaporated by using rotary evaporator. The crude product was purified by column chromatography (silica, hexane ethyl acetate 70:30) to give titled product (**34**) as white solid (440 mg, 62% over 2 steps). TLC (N-Alumina) R_f = 0.35 (50:50 EtOAc/hexane; UV) ^{1}H NMR (500 MHz, $CDCl_3$) δ 7.81 (dd, *J* = 5.3, 3.2 Hz, 2H, H6), 7.69 (dd, *J* = 5.2, 3.0 Hz, 2H, H5), 3.78 (t, *J* = 6.4 Hz, 2H, H2''), 3.48 (s, 3H, CH_3), 3.20 – 3.12 (m, 2H, H3), 2.94 – 2.85 (m, 2H, H2), 2.61 (t, *J* = 6.1 Hz, 2H, H1''), 2.58 – 2.47 (m ,2H, H2'), 2.35 – 2.23 (m, 2H, H3'). ^{13}C NMR (126 MHz, $CDCl_3$) δ 168.3 (C1'''), 133.8 (C6), 132.4 (C4), 123.2 (C5), 58.9 (CH_3), 55.0 (C3), 54.7 (C1''), 51.7

(C2), 35.5 (C2''). HRMS-ESI (*m/z*): [M + H^+] calculated for $[C_{15}H_{20}N_3O_3]^+$: 290.1499, found: 290.1495.

2-(4-Methoxypiperazin-1-yl)ethan-1-amine (35)

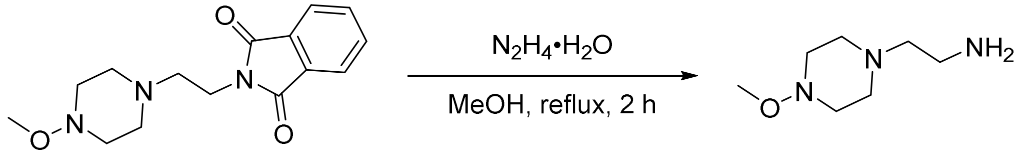

To a mixture of 2-(2-(4-methoxypiperazin-1-yl)ethyl)isoindoline-1,3-dione (**34**) (440 mg, 1.52 mmol, 1 equiv.) in methanol (20 mL) was added hydrazine monohydrate (55 w%, 278 mg, 3.05 mmol, 2.0 equiv.). The reaction was heated to reflux for 2 h. After this time, the reaction was concentrated, 2 M HCl (15 mL) was added to the pale grey remnant and the mixture was heated for 15 min, followed by stirring at rt for 30 min. NaOH aqueous solution (25 w%) was added to alter the pH to 10. The mixture was extracted by dichloromethane (20 mL × 3). The combined organic layers were dried over $MgSO_4$, filtered, and concentrated. The crude product (**35**) was processed into the next step without further purification as yellowish oil. HRMS-ESI (*m/z*): [M + H^+] calculated for $[C_7H_{18}N_3O]^+$: 160.1444, found: 160.1439.

6-(2-Chloro-4-(6-methylpyrazin-2-yl)phenyl)-8-ethyl-2-((2-(4-methoxypiperazin-1-yl)ethyl)amino)pyrido[2,3-*d*]pyrimidin-7(8*H*)-one (11)

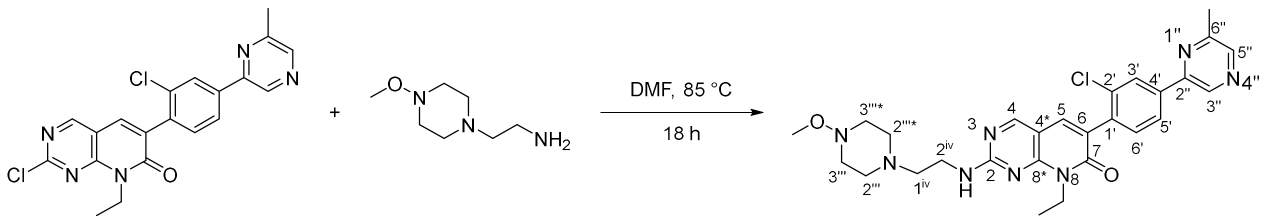

In an oven dried flask, under argon atmosphere, a solution of (**14**) (120 mg, 0.291 mmol, 1.0 equiv.) in DMF (3.6 mL) was treated with (**35**) (92 mg, 0.587 mmol, 2.0 equiv.) at room temperature and then brought to 85 °C and stirred for 18 h. After such time, the reaction mixture was diluted with EtOAc (50 mL), and washed with 5% aqueous NaOH (25 mL × 2), brine (25 mL) dried over Na_2SO_4, filtered, and concentrated under reduced pressure. The resulting crude residue was purified by neutral alumina flash column (eluent: 5 % MeOH in DCM) to afford the title compound (**11**) as yellow solid (90 mg, 58%). TLC (N-Alumina) R_f = 0.45 (5:95 MeOH/DCM;

UV). ^{1}H NMR (500 MHz, $CDCl_3$) δ 8.81 (s, 1H, H5), 8.45 (s, 1H, H4), 8.40 (s, 1H, H3'), 8.15 (d, *J* = 1.8 Hz, 1H, H5''), 7.91 (dd, *J* = 8.0, 1.8 Hz, 1H, H5'), 7.55 (s, 1H, H3''), 7.50 (d, *J* = 7.9 Hz, 1H, H6'), 4.56 – 4.38 (m, 2H, CH_2), 3.59 (q, *J* = 5.8 Hz, 2H, $H2^{iv}$), 3.53 (s, 3H, CH_3), 3.25 (d, *J* = 10.1 Hz, 2H, H3'''*), 2.90 (d, *J* = 11.1 Hz, 2H, H3'''), 2.73 – 2.63 (m, 4H, H2'''*, $H1^{iv}$), 2.62 (s, 3H, CH_3), 2.36 (d, *J* = 11.3 Hz, 2H, H2'''), 1.34 (t, *J* = 7.0 Hz, 3H, CH3); ^{13}C NMR (126 MHz, $CDCl_3$) δ 161.7 (C=O), 161.7, 158.9, 155.5, 153.6, 150.1, 143.4, 139.0, 137.8, 136.7, 136.0, 134.2, 133.0, 128.2, 125.0 (aromatic), 59.2 (CH_3), 56.0 ($C1^{iv}$), 54.8 (C2), 51.7 (C3), 38.5 ($C2^{iv}$), 36.5 (CH_2), 21.8 (CH_3), 13.1 (CH_3). HRMS-ESI (*m/z*): $[M + H]^+$ calculated for $[C_{28}H_{27}N_{10}O_2]^+$: 535.2313, found 535.2317.

Allyl hydroxycarbamate

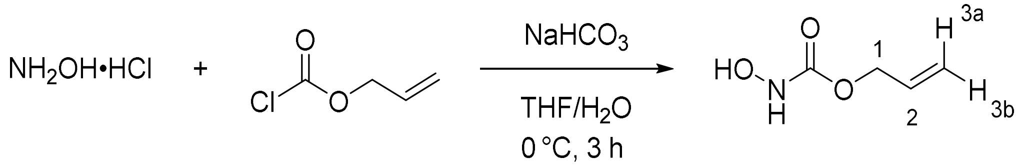

In an oven dried flask, under argon atmosphere, to a stirred solution of hydroxylamine hydrochloride (5.50 g, 79.15 mmol, 1.10 equiv.) in anhydrous THF (47 mL) and H_2O (87 mL) was added $NaHCO_3$ (9.07 g, 107.9 mmol, 1.5 equiv.) The reaction mixture was cooled to 0 °C and treated with allyloxycarbonyl chloride (7.68 mL, 72 mmol, 1.0 equiv.) dissolved in 40 mL THF dropwise over a period of 20 min. The reaction mixture was stirred for 3 h at 0 °C then was diluted with 100 mL H_2O. The organic layer was decanted and the aqueous phase was extracted with EtOAc (70 mL × 3), and the combined organic layer was washed with aq. $NaHCO_3$ (100 mL), dried over Na_2SO_4, filtered and concentrated under reduced pressure. The resulting crude residue was purified by silica flash chromatography (eluent: 5% MeOH in DCM) to afford the title compound (5.34 g, 64 %) as a colorless oil. TLC R_f = 0.50 (10:90 MeOH/DCM; $KMnO_4$). ^{1}H NMR (500 MHz, $CDCl_3$) δ 7.58 (s, 1H, NH), 5.95 – 5.85 (m, 1H, H2), 5.32 (dd, *J* = 17.2, 1.5 Hz, 1H, H3a), 5.24 (dd, *J* = 10.4, 1.3 Hz, 1H, H3b), 4.63 (d, *J* = 5.7 Hz, 2H, H1); ^{13}C NMR (126 MHz, $CDCl_3$) δ 159.3, (C=O), 131.9 (C2), 118.9 (C3), 66.8 (C1). HRMS-ESI (*m/z*): $[M + H]^+$ calculated for $[C_4H_8NO_3]^+$: 118.0504, found: 118.9787.

2-Allyl 5-(*tert*-butyl) 1,2,5-oxadiazepane-2,5-dicarboxylate (37) (rotamers ratio 1:1)

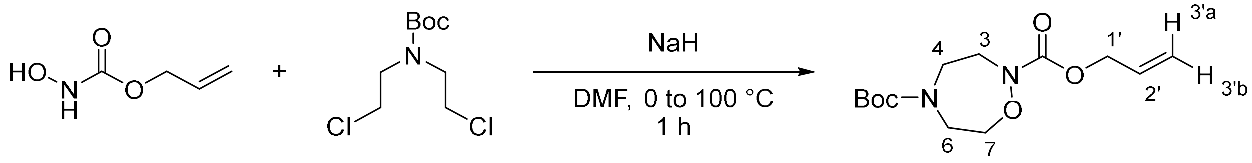

In an oven dried flask, NaH (60 w% dispersion in mineral oil) (3.01 g, 75.23 mmol, 2.2 equiv.) was added to a stirred solution of allyl hydroxycarbamate (4.0 g, 34.16 mmol, 1.0 equiv.) in anhydrous DMF (68 mL) at 0 °C and the mixture was stirred for 20 min. Compound (**36**) (8.28 g, 34.16 mmol, 1.0 equiv.) dissolved in 68 mL DMF was then added at 0 °C dropwise over 20-25 min. The reaction mixture was stirred for 20 min at 0 °C, and then brought to 100 °C and stirred for 1 h. After such time, the reaction mixture was diluted with 150 mL Et_2O, and washed with sat. aqueous NH_4Cl (150 mL). The aqueous phase was washed with Et_2O (100 mL × 2), the combined organic phases were washed with brine (100 mL), dried over Na_2SO_4, filtered and concentrated under reduced pressure. The resulting crude residue was separated by silica flash chromatography (eluent: 20% EtOAc in hexane) to access the title compound (**37**) (3.62 g, 37 %) as a colorless oil. TLC R_f = 0.35 (20:80 EtOAc/hexane; $KMnO_4$). ^{1}H NMR (500 MHz, $CDCl_3$) δ 5.97 – 5.88 (m, 1H, H2'), 5.32 (dd, J = 17.2, 1.5 Hz, 1H, H3'a), 5.24 (dd, J = 10.4, 1.3 Hz, 1H, H3'b), 4.64 (d, J = 5.7 Hz, 2H, H1'), 4.02 (m, 2H, H7), 3.76 (m, 2H, H4), 3.60 – 3.54 (m, 4H, H3, H6), 1.45 (s, 9H, $C(CH_3)_3$); ^{13}C NMR (126 MHz, $CDCl_3$) δ 156.2, 156.1 (C=O), 155.1, 155.1 (C=O), 132.4 (C2'), 118.4 (C3'), 80.4, 80.3 (quat), 73.7, 72.9 (C7), 66.8 (C1'), 49.4, 49.3 (C4), 48.0, 47.9 (C3), 46.8, 46.7 (C6), 28.5 ($C(CH_3)_3$). HRMS-ESI (m/z): $[M + Na]^+$ calculated for $[C_{13}H_{22}N_2O_5Na]^+$: 309.1420, found: 309.1421.

***tert*-Butyl 2-allyl-1,2,5-oxadiazepane-5-carboxylate (38) (rotamers ratio 1:1.3)**

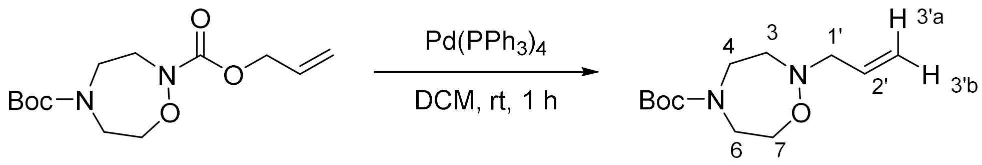

In an oven dried flask, under an argon atmosphere, (**37**) (5.29 g, 18.47 mmol, 1.0 equiv.) was dissolved in anhydrous DCM (32 mL), tetrakis(triphenylphosphine)palladium(0) (427 mg, 0.369 mmol, 0.02 equiv.) was added and the reaction mixture was stirred for 1 h at room temperature. After such time, the reaction mixture was passed through a celite plug and concentrated under reduced pressure. The residue obtained was separated by silica flash chromatography (eluent: 10% EtOAc in hexane) to afford the desired compound (**38**) (3.37 g, 75 %) as a colorless oil. TLC R_f = 0.35 (10:90 EtOAc/hexane; $KMnO_4$). ^{1}H NMR (500 MHz, $CDCl_3$) δ 5.95 – 5.85 (m, 1H, H2'), 5.20 (d, J = 17.0 Hz, 1H, H3'a), 5.14 (d, J = 10.3 Hz, 1H, H3b), 3.86 (t, J = 5.8 Hz, 1H, H7), 3.80

(t, J = 5.9 Hz, 1H, H7), 3.55 – 3.46 (m, 4H, H3, H4), 3.35 (d, J = 3.5 Hz, 2H, H1'), 2.84 (m, 2H, H6), 1.45 (s, 9H, $C(CH_3)_3$); ^{13}C NMR (126 MHz, $CDCl_3$) δ 155.7, 155.6 (C=O), 134.1 (C2'), 118.1 (C3'), 79.6, 79.6 (quat), 69.6, 69.5 (C7), 62.8, 62.7 (C1'), 57.5, 57.2 (C3), 48.7 (C6), 48.4, 48.0 (C4), 28.6, 28.6 ($C(CH_3)_3$). HRMS-ESI (*m/z*): $[M + Na]^+$ calculated for $[C_{12}H_{22}N_2O_3Na]^+$: 265.1522, found: 265.1516.

***tert*-Butyl 2-(2-hydroxyethyl)-1,2,5-oxadiazepane-5-carboxylate (39) (rotamers ratio 1:1.2)**

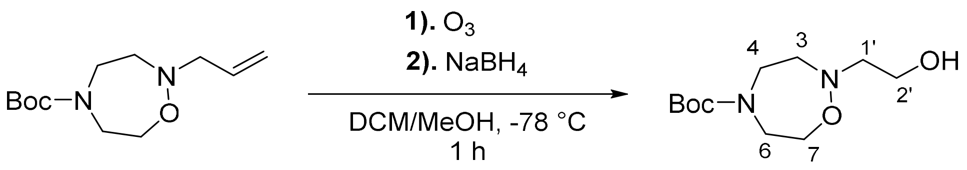

Compound (**38**) (2.97 g, 12.26 mmol, 1.0 equiv.) was dissolved in a stirred mixture of DCM (123 mL) and methanol (30.6 mL) and cooled to −78 °C and then was sparged with ozone for 0.5 h until full consumption of the starting material observed by HRMS-ESI. Argon was then passed through the reaction to disperse residual ozone before $NaBH_4$ (928 mg, 24.51 mmol, 2.0 equiv.) was added in 4 portions at 5 min interval at −78 °C, and the solution gradually warmed to rt and stirred for 1 h. After such time, the reaction was quenched with aq. $NaHCO_3$ (150 mL) and the aqueous layer was extracted with DCM (100 mL × 2). The organic layers were combined and washed with brine (100 mL × 2), dried over Na_2SO_4, filtered, and concentrated under reduced pressure to afford the alcohol (**39**) (2.67 g, 88% crude yield) as a clear oil. TLC R_f = 0.40 (10:90 MeOH/DCM; CAM). ^1H NMR (500 MHz, $CDCl_3$) δ 3.88 (t, J = 5.8 Hz, 1H, H7), 3.83 (t, J = 5.8 Hz, 1H, H7), 3.72 (t, J = 5.0 Hz, 2H, H2'), 3.54 – 3.47 (m, 4H, H4, H6), 2.90 (dt, J = 14.6, 5.7 Hz, 2H, H3), 2.84 (m, 2H, H1'), 2.33 (br s, 1H, OH), 1.44 (s, 9H, $C(CH_3)_3$); ^{13}C NMR (126 MHz, $CDCl_3$) δ 155.7, 155.6 (C=O), 79.8 (quat), 70.4, 70.4 (C7), 61.4, 61.4 (C2'), 59.7, 59.7 (C3), 58.5, 58.0 (C1'), 48.9, 48.9 (C6), 48.1, 47.7 (C4), 28.5, 28.5 $C(CH_3)_3$). HRMS-ESI (*m/z*): $[M + Na]^+$ calculated for $[C_{11}H_{22}N_2O_4Na]^+$: 269.1471, found: 269.1468.

***tert*-Butyl 2-(2-((methylsulfonyl)oxy)ethyl)-1,2,5-oxadiazepane-5-carboxylate (40) (rotamers ratio 1:1.1)**

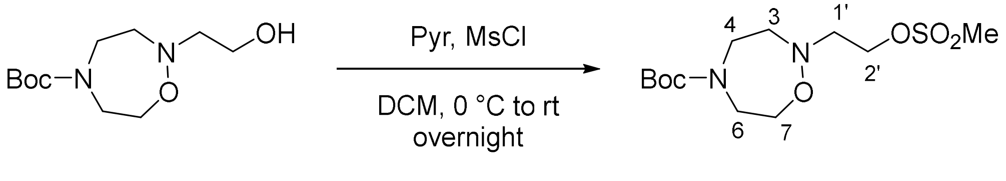

The above crude (**39**) (2.60 g, 10.56 mmol, 1 equiv.) was dissolved in dry DCM (22 mL), and pyridine (2.13 mL, 26.39 mmol, 2.5 equiv.) was added. The reaction mixture was brought to 0 °C and was treated with methanesulfonyl chloride (1.23 mL, 15.83 mmol, 1.5 equiv.) and stirred overnight at room temperature. After such time, the reaction mixture was diluted with DCM (50 mL) and washed with H_2O (50 mL), sat. aqueous $CuSO_4$ (50 mL), and brine (50 mL), dried over Na_2SO_4, filtered, and concentrated under reduced pressure. The resulting residue was separated over silica flash chromatography (eluent: 3% MeOH in DCM) to access compound (**40**) (2.54 g, 66% over 2 steps) as a light-yellow oil. TLC R_f = 0.60 (5:95 MeOH/DCM; CAM). ^{1}H NMR (500 MHz, $CDCl_3$) δ 4.36 (t, J = 5.4 Hz, 2H, H2'), 3.87 (t, J = 5.8 Hz, 1H, H7), 3.82 (t, J = 5.9 Hz, 1H, H7), 3.53 – 3.45 (m, 4H, H4, H6), 3.01 (s, 3H, CH_3), 2.97 (m, 2H, H1'), 2.86 (dt, J = 12.0, 5.6 Hz, 2H, H3), 1.42 (s, 9H, $C(CH_3)_3$); ^{13}C NMR (126 MHz, $CDCl_3$) δ 155.5, 155.5 (C=O), 79.7, 79.7 (quat), 70.2, 70.1 (C7), 66.5 (C2'), 58.5, 58.3 (C1'), 58.2, 58.1 (C3), 48.7, 48.7 (C6), 48.0, 47.6 (C4), 37.8, 37.7 (CH_3), 28.5 ($C(CH_3)_3$). HRMS-ESI (*m/z*): $[M + Na]^+$ calculated for $[C_{12}H_{24}N_2O_6{}^{32}SNa]^+$: 347.1247, found: 347.1245.

***tert*-Butyl 2-(2-azidoethyl)-1,2,5-oxadiazepane-5-carboxylate (41) (rotamers ratio 1:1.3)**

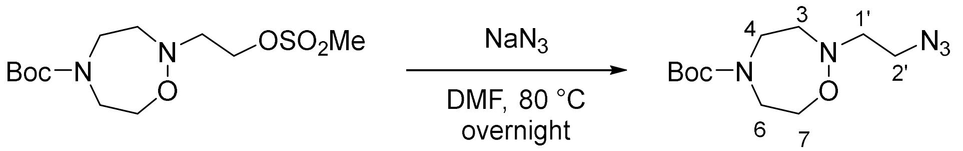

Under an argon atmosphere, to a solution of (**40**) (2.40 g, 7.40 mmol, 1 equiv.) in dry DMF (35.2 mL) was added sodium azide (1.20 g, 18.5 mmol, 2.5 equiv.) at room temperature, and then the reaction mixture was warmed up to 80 °C and stirred for 8 h. After such time, the reaction mixture diluted with Et_2O (100 mL) and washed with aq. $NaHCO_3$ (100 mL), and brine (100 mL). The organic layer was dried over Na_2SO_4, filtered, and concentrated under reduced pressure. The resulting crude residue was separated by silica flash chromatography (eluent: 20% EtOAc in Hexane) to afford the title compound (**41**) (1.49 g, 74%) as a yellow oil. TLC R_f = 0.45 (20:80 EtOAc/hexane; CAM). ^{1}H NMR (500 MHz, $CDCl_3$) δ 3.91 (t, J = 5.9 Hz, 1H, H7), 3.85 (t, J = 5.9 Hz, 1H, H7), 3.58 – 3.49 (m, 4H, H4, H6), 3.40 (m, 2H, H1'), 2.92 – 2.84 (m, 4H, H3, H2'), 1.46 (s, 9H, $C(CH_3)_3$); ^{13}C NMR (126 MHz, $CDCl_3$) δ 155.6, 155.5 (C=O), 79.8, 79.7 (quat), 70.1, 69.8 (C7), 59.1, 59.0 (C3), 59.0, 58.5 (C2'), 48.6, 48.4 (C6), 48.3, 48.3 (C4), 47.8 (C1'), 28.6 ($C(CH_3)_3$). HRMS-ESI (*m/z*): $[M + Na]^+$ calculated for $[C_{11}H_{21}N_5O_3Na]^+$: 294.1536, found: 294.1538.

2-(2-Azidoethyl)-5-methyl-1,2,5-oxadiazepane (42)

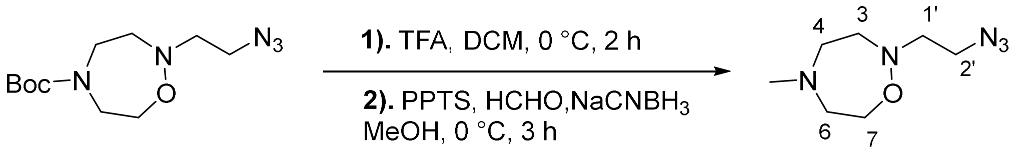

TFA (1.7 mL, 22.11 mmol, 25 equiv.) was added at 0 °C under an argon atmosphere to a stirred solution of (**41**) (240 mg, 0.885 mmol, 1 equiv.) in dry DCM (4.4 mL). The reaction mixture was stirred for 2 h then was quenched with $NaHCO_3$ (25 mL) and diluted with a 3:1 mixture of chloroform and isopropanol (25 mL). The layers were separated, and the aqueous layer was washed with 3:1 mixture of chloroform and isopropanol (30 mL x 3), and the combined organics were dried over Na_2SO_4, filtered, and concentrated under reduced pressure. The resulting crude product was dissolved in dry methanol (7.6 mL) and treated with added pyridinium *p*-toluenesulfonate (1.91 g, 7.59 mmol, 10 equiv.) and then 37% aqueous formaldehyde (0.616 mL, 7.59 mmol, 10 equiv.) at room temperature and the mixture was stirred for 10 min. The mixture was cooled to 0 °C and sodium cyanoborohydride (96 mg, 1.52 mmol, 2 equiv.) was added and stirring continued at 0 °C for 3 h. After such time, the reaction mixture was diluted with EtOAc (50 mL), washed with sat $NaHCO_3$ (50 mL) and brine (25 mL), dried over Na_2SO_4 and concentrated under reduced pressure. The residue was purified by silica flash column chromatography (eluent: 5% MeOH in DCM) to give the title compound (**42**) (91 mg, 76% over 2 steps) as a yellow oil. TLC R_f = 0.30 (5:95 MeOH/DCM; CAM). ^{1}H NMR (500 MHz, $CDCl_3$) δ 3.87 (t, *J* = 6.0 Hz, 2H, H7), 3.38 (t, *J* = 5.7 Hz, 2H, H3), 2.90 (t, *J* = 5.7 Hz, 2H, H1’), 2.84 (t, *J* = 5.7 Hz, 2H, H4), 2.79 (t, *J* = 6.0 Hz, 2H, H6), 2.73 (t, *J* = 5.7 Hz, 2H, H2’), 2.46 (s, 3H, CH_3); ^{13}C NMR (126 MHz, $CDCl_3$) δ 70.8 (C7), 59.2 (C1’), 59.0 (C4), 58.2 (C2’), 57.7 (C6), 48.5 (C3), 45.6 (CH_3). HRMS-ESI (*m/z*): $[M + H]^+$ calculated for $[C_7H_{16}N_5O]^+$: 186.1349, found: 186.1347.

2-(5-Methyl-1,2,5-oxadiazepan-2-yl)ethan-1-amine (43)

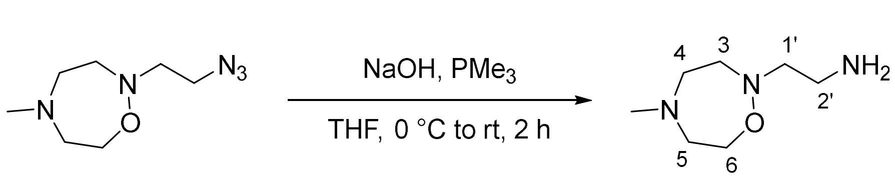

Under an argon atmosphere, to a stirred solution of (**42**) (86 mg, 0.465 mmol, 1 equiv.) in dry THF (1.86 mL) was added NaOH (1 M) (1.44 mL, 1.44 mmol, 3.10 equiv.) at 0 ºC. After 5 min, at 0 ºC

trimethyl phosphine (1 M) (0.929 mL, 0.929 mmol, 2.0 equiv.) was added dropwise, before the reaction mixture was brought to room temperature and stirred for 2 h. The reaction mixture was then cooled to 0 ºC and acidified to pH ≈ 2 by dropwise addition of 4 M HCl, then washed with Et_2O (25 mL x 3). The aqueous phase was basified to pH ≈ 10 by dropwise addition of 4 M NaOH, and extracted with chloroform: isopropanol (3:1) (30 mL x 4), dried over Na_2SO_4, filtered, and concentrated under reduced pressure at 20 ºC. The residue was purified by silica flash column chromatography (eluent: 20 % MeOH in DCM) to give the title compound (**43**) (39 mg, 53%) as a colorless oil. TLC R_f = 0.10 (20:80 MeOH/DCM; Ninhydrin). ^{1}H NMR (500 MHz, $CDCl_3$) δ 3.85 (t, *J* = 5.8 Hz, 2H, H7), 2.88 (m, 4H, H3, H4), 2.81 – 2.68 (m, 6H, H6, H1', H2'), 2.44 (s, 3H, CH_3), 2.17 (s, 2H, NH_2); ^{13}C NMR (126 MHz, $CDCl_3$) δ 71.2 (C7), 62.4 (C3), 59.0 (C6), 58.4 (C1'), 58.2 (C2'), 46.1 (C4), 40.4 (CH_3). HRMS-ESI (*m/z*): $[M + H]^+$ calculated for $[C_7H_{18}N_3O]^+$: 160.1444, found: 160.1440.

6-(2-Chloro-4-(6-methylpyrazin-2-yl)phenyl)-8-ethyl-2-((2-(5-methyl-1,2,5-oxadiazepan-2-yl)ethyl)amino)pyrido[2,3-*d*]pyrimidin-7(8*H*)-one (12)

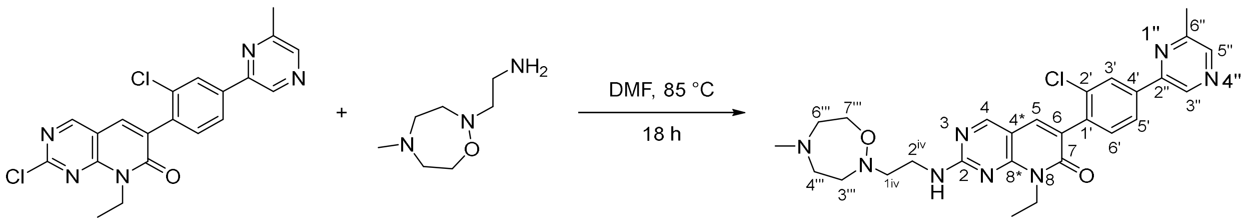

A stirred solution of (**14**) (31 mg, 0.075 mmol, 1.0 equiv.) under argon atmosphere in dry DMF (1.0 mL) was treated (**43**) (24 mg, 0.150 mmol, 2.0 equiv.) at room temperature and then warmed up to 85 ºC and stirred for 18 h. After such time, the reaction mixture was diluted with Et_2O (50 mL), and washed with 5% aqueous NaOH (15 mL × 2), and brine (15 mL), dried over Na_2SO_4, filtered, and concentrated under reduced pressure. The resulting residue was purified by neutral alumina flash column (eluent: 3% MeOH in DCM) to afford (**12**) as white solid (15 mg, 37%). TLC (N-Alumina) R_f = 0.30 (10:90 MeOH/DCM; UV). ^{1}H NMR (500 MHz, $CDCl_3$) δ 8.83 (s, 1H, H5), 8.48 (s, 1H, H4), 8.42 (s, 1H, H3'), 8.16 (d, *J* = 1.8 Hz, 1H, H5''), 7.93 (dd, *J* = 8.0, 1.8 Hz, 1H, H5'), 7.57 (s, 1H, H6'), 7.52 (d, *J* = 8.0 Hz, 1H, H3''), 6.12 (br s, 1H, NH), 4.49 (s, 2H, $H2^{iv}$), 3.97 (t, *J* = 5.8 Hz, 2H, H3'''), 3.73 (q, *J* = 5.0 Hz, 2H, CH_2), 3.03 (t, *J* = 5.8 Hz, 2H, H7'''), 2.98 (t, *J* = 5.5 Hz, 2H, $H1^{iv}$), 2.93 (t, *J* = 5.9 Hz, 2H, H6'''), 2.88 (t, *J* = 5.6 Hz, 2H, H4'''), 2.64 (s, 3H, CH_3), 2.58 (s, 3H, CH_3), 1.35 (t, *J* = 7.0 Hz, 3H, CH_3); ^{13}C NMR (226 MHz, $CDCl_3$) δ 161.8 (C=O), 161.7, 159.0, 155.6, 153.6, 150.1, 143.4, 139.0, 137.9, 136.7, 136.0, 134.9, 132.4, 128.3, 125.0

(aromatic), 70.5 (C3'''), 58.3 (C7'''), 58.1 (C1^{iv}), 57.9 (C6'''), 57.9 (C4'''), 45.3 (CH_3), 39.4 (CH_2), 36.6 (C2^{iv}), 21.9 (CH_3), 13.1 (CH_3). HRMS-ESI (*m/z*): $[M + H]^+$ calculated for $[C_{27}H_{32}N_8O_2{}^{35}Cl]^+$: 535.2331, found: 535.2331.

2-Benzyl 5-(*tert*-butyl) 1,2,5-oxadiazepane-2,5-dicarboxylate (44) (rotamers ratio 1:1.3)

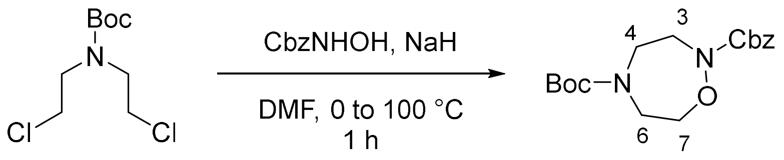

In an oven dried flask NaH (60 w% dispersion in mineral oil) (1.64 g, 40.93 mmol, 2.2 equiv.) was added to a stirred solution of (**36**) (4.5 g, 18.60 mmol, 1.0 equiv.) in anhydrous DMF (38 mL) at 0 °C and the mixture was stirred for 20 min. Benzyl hydroxycarbamate (3.11 g, 18.60. mmol, 1.0 equiv.) dissolved in (38 mL) DMF was then added at 0 °C dropwise over 20-25 min. The reaction mixture was stirred for 20 min at 0 °C, and then brought to 100 °C and stirred for 1 h. After such time, the reaction mixture was diluted with 150 mL Et_2O, and washed with (125 mL) of sat. aqueous NH_4Cl. The aqueous phase was washed with Et_2O (100 mL × 2), the combined organic phases were washed with brine (75 mL), dried over Na_2SO_4, filtered and concentrated under reduced pressure. The resulting crude residue was separated by silica flash chromatography (eluent: 15% EtOAc in hexane) to access the title compound (**44**) (2.64 g, 42%) as a colorless oil. TLC R_f = 0.33 (20:80 EtOAc/hexane). ^{1}H NMR (500 MHz, C_6D_6) δ 7.21 – 7.13 (m, 4H, Ar), 7.05 – 6.94 (m, 6H, Ar), 5.01 (s, 2H, CH_2), 5.00 (s, 2H, CH_2), 3.74 (t, *J* = 5.5 Hz, 2H, H7), 3.69 (t, *J* = 5.5 Hz, 2H, H7), 3.55 (t, *J* = 5.0 Hz, 2H, H3), 3.50 (t, *J* = 5.0 Hz, 2H, H3), 3.16 (t, *J* = 5.5 Hz, 2H, H4), 3.12 (t, *J* = 5.5 Hz, 2H, H4), 3.02 (t, *J* = 5.0 Hz, 2H, H6), 2.94 (t, *J* = 5.0 Hz, 2H, H6), 1.31 (s, 9H, $C(CH_3)_3$), 1.28 (s, 9H, $C(CH_3)_3$); ^{13}C NMR (126 MHz, C_6D_6) δ 156.0, 156.0 (C=O), 154.5, 154.5 (C=O), 136.6, 128.4, 128.1, 128.0, 128.0, 127.9, 127.7 (aromatic), 79.3, 79.2 (quat), 73.0, 72.3 (C7), 67.3 (CH_2), 49.0 (C3), 47.8, 47.5 (C4), 46.6, 46.3 (C6), 28.1, 28.0 ($C(CH_3)_3$); HRMS-ESI (*m/z*): $[M + Na]^+$ calculated for $[C_{17}H_{24}N_2O_5Na]^+$: 359.1583, found: 359.1575.

***tert*-Butyl 1,2,5-oxadiazepane-5-carboxylate (45) (rotamers ratio 1:1.25)**

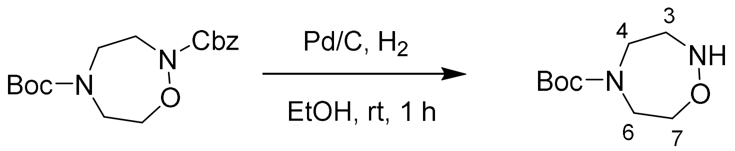

In an oven dried flask, under H_2 atmosphere, (**44**) (1.72 g, 5.11 mmol, 1.0 equiv.) was dissolved in anhydrous ethanol (120 mL), Palladium on carbon Pd/C (10 w%) (109 mg, 0.102 mmol, 0.02 equiv.) was added and the reaction mixture was stirred for 1 h at room temperature. After such time, the reaction mixture was passed through a celite plug and concentrated under reduced pressure. The residue obtained was separated by silica flash chromatography (eluent: 35% EtOAc in hexane) to afford the title compound (**45**) (616 mg, 60%) as a white solid. TLC R_f = 0.35 (30:70 EtOAc/hexane).^{1}H NMR (500 MHz, $CDCl_3$) δ 3.86 (t, J = 5.7 Hz, 2H, H7), 3.81 (t, J = 5.7 Hz, 2H, H7), 3.59 – 3.47 (m, 2×4H, H4, H6), 3.12 – 3.04 (m, 2×2H, H3), 1.45 (s, 2×9H, $C(CH_3)_3$);.^{13}C NMR (126 MHz, $CDCl_3$) δ 155.6, 155.5 (C=O), 79.7 (quat), 70.4, 70.2 (C7), 51.8, 51.5 (C3), 49.2, 48.7 (C6), 48.3, 48.2 (C4), 28.6, 28.5 ($C(CH_3)_3$); HRMS-ESI (m/z): $[M + Na]^+$ calculated for $[C_9H_{18}N_2O_3Na]^+$: 225.1215, found: 225.1211.

tert-Butyl 2-methyl-1,2,5-oxadiazepane-5-carboxylate (46) (rotamers ratio 1:1.25)

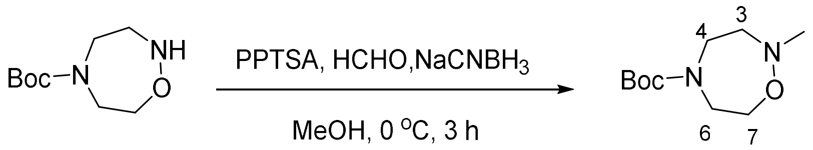

Under an argon atmosphere, compound (**45**) (650 mg, 3.21 mmol, 1.0 equiv.) was dissolved in dry methanol (32 mL) and treated with pyridinium *p*-toluenesulfonate (8.08 g, 32.14 mmol, 10 equiv.) and then 37% aqueous formaldehyde (2.61 mL, 32.14 mmol, 10 equiv.) at room temperature and the mixture was stirred for 10-15 min. Then the reaction mixture was cooled to 0 °C and sodium cyanoborohydride (404 mg, 6.43 mmol, 2 equiv.) was added and stirred it for 3 h at 0 °C. After such time, the reaction mixture was diluted with EtOAc (60 mL), washed with sat $NaHCO_3$ (60 mL) and brine (50 mL), dried over Na_2SO_4 and concentrated under reduced pressure. The residue was purified by silica flash column chromatography (eluent: 15% EtOAc in hexane) to give the title compound (**46**) (573 mg, 82%) as a colorless oil. TLC R_f = 0.37 (20:80 EtOAc/Hexane).^{1}H NMR (500 MHz, $CDCl_3$) δ 3.83 (t, J = 5.7 Hz, 2H, H7), 3.77 (t, J = 5.8 Hz, 2H, H7), 3.51 – 3.40 (m, 2×4H, H4, H6), 2.82 – 2.72 (m, 2×2H, H3), 2.58 (s, 2×3H, CH_3), 1.42 (s, 2×9H, $C(CH_3)_3$); ^{13}C NMR (126 MHz, $CDCl_3$) δ 155.6, 155.5 (C=O), 79.5, 79.5 (quat), 69.0, 68.9

(C7), 59.8, 59.4 (C3), 48.6, 48.6 (C6), 48.3, 47.9 (C4) , 47.4, 47.2 (CH_3), 28.5, 28.5 ($C(CH_3)_3$). HRMS-ESI (*m/z*) $[M + Na]^+$ calculated for $[C_{10}H_{20}N_2O_3Na]$: 239.1372, found: 239.1362.

2-(2-(2-Methyl-1,2,5-oxadiazepan-5-yl)ethyl)isoindoline-1,3-dione (48)

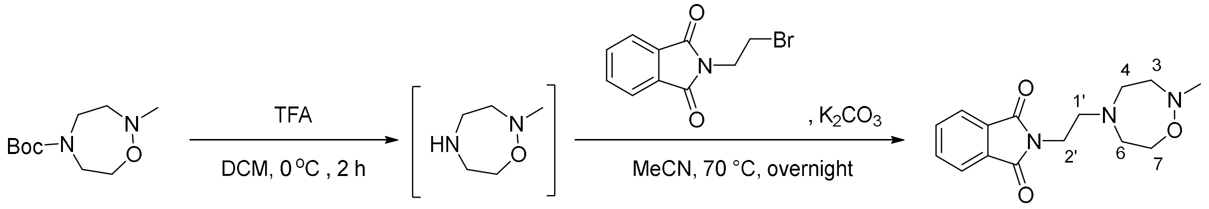

TFA (5.07 mL, 66.23 mmol, 25 equiv.) was added at 0 °C under an argon atmosphere to a stirred solution of (**46**) (573 mg, 2.65 mmol, 1 equiv.) in dry DCM (13.2 mL). The reaction mixture was stirred for 2 h then was quenched with $NaHCO_3$ (30 mL) and diluted with a 3:1 mixture of chloroform and isopropanol (30 mL). The layers were separated, and the aqueous layer was washed with a mixed solvent of chloroform: isopropanol, 3:1 (30 mL × 4), and the combined organics were dried over Na_2SO_4, filtered, and concentrated under reduced pressure. The resulting crude product (**47**) (250 mg, 81%) was subjected to the next step without further purification. Under an argon atmosphere, the crude (**47**) (150 mg, 1.29 mmol, 1.0 equiv.) was dissolved in acetonitrile (3.3 mL) and was added K_2CO_3 (536 mg, 3.87 mmol, 3.0 equiv.) and then *N*-(2-bromoethyl)phthalimide (492 mg, 1.94 mmol, 1.50 equiv.) at room temperature and the mixture was heated to 70 °C and stirred overnight for 12 h. After such time, the reaction mixture was concentrated, taken in dichloromethane (50 mL), washed with water (30 mL) and the aqueous phase was washed with DCM (30 mL × 3), dried over Na_2SO_4 and concentrated under reduced pressure. The residue was purified by silica flash column chromatography (eluent: 70% EtOAc in hexane) to give the title compound (**48**) (115 mg, 25% over 2 steps) as a yellow oil. TLC R_f = 0.30 (80:20 EtOAc/Hexane). ^1H NMR (500 MHz, $CDCl_3$) δ 7.79 (m, 2H, Ar), 7.73 – 7.61 (m, 2H, Ar), 3.81 (t, *J* = 5.8 Hz, 2H, H7), 3.77 (t, *J* = 6.8 Hz, 2H, H1'), 2.95 (t, *J* = 6.2 Hz, 4H, H6, H2'), 2.89 (t, *J* = 5.4 Hz, 2H, H4), 2.78 (t, *J* = 5.5 Hz, 2H, H3), 2.56 (s, 3H, CH_3); ^{13}C NMR (126 MHz, $CDCl_3$) δ 168.4 (C=O), 134.1, 132.3, 123.2 (aromatic), 69.7 (C7), 60.0 (C3), 55.2 (C4), 54.5 (C6),

51.7 (C2’), 47.5 (CH_3), 35.9 (C1’). HRMS-ESI (*m/z*): $[M + H]^+$ calculated for $[C_{15}H_{20}N_2O_3N]^+$: 290.1499, found: 290.1499.

2-(2-Methyl-1,2,5-oxadiazepan-5-yl)ethan-1-amine (49)

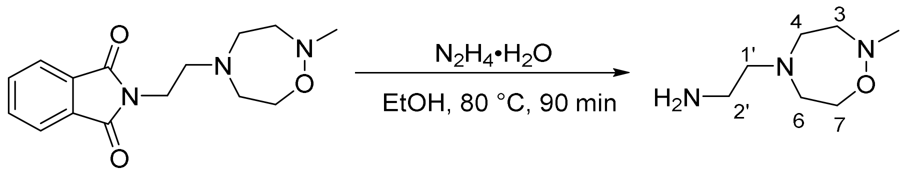

In an oven dried flask, under argon atmosphere, to a solution of (**48**) (110 mg, 0.380 mmol, 1.0 equiv.) in EtOH (1.3 mL) was added hydrazine monohydrate (0.56 mL, 1.14 mmol, 3.0 equiv.) at room temperature, and brought the reaction mixture at 80 ºC and stirred for 1.5 h. After such time, the reaction mixture was filtered, the filter-cake was washed with EtOH (25 mL), and concentrated. The resulting crude residue was purified by neutral alumina flash column on (eluent: 10 % MeOH in DCM) to afford the title compound (**49**) as a colorless oil (41 mg, 68%). TLC (N-Alumina) R_f = 0.3 (10:90 MeOH/DCM; Ninhydrin). ^{1}H NMR (500 MHz, C_6D_6) δ 3.74 (t, *J* = 5.6 Hz, 2H, H7), 2.75 (t, *J* = 6.0 Hz, 2H, H6), 2.67 (t, *J* = 5.2 Hz, 2H, H3), 2.63 – 2.54 (m, 6H, H4, H1’, H2’), 2.49 (s, 3H, CH_3), 2.24 (br s, 2H, NH_2); ^{13}C NMR (126 MHz, C_6D_6) δ 70.0 (C7), 60.5 (C4), 56.6 (C1’), 55.7 (C3), 54.9 (C6), 47.6 (CH_3), 40.0 (C2’). HRMS-ESI (*m/z*): $[M + H]^+$ calculated for $[C_7H_8N_3O]^+$: 160.1444, found 160.1444.

6-(2-Chloro-4-(6-methylpyrazin-2-yl)phenyl)-8-ethyl-2-((2-(2-methyl-1,2,5-oxadiazepan-5-yl)ethyl)amino)pyrido[2,3-*d*]pyrimidin-7(8*H*)-one (13)

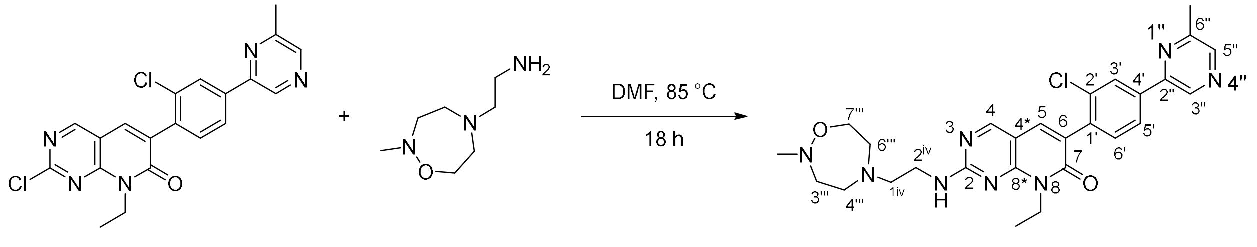

In an oven dried flask, under argon atmosphere, a solution of (**14**) (53 mg, 0.129 mmol, 1.0 equiv.) in DMF (1.6 mL) was treated with (**49**) (41 mg, 0.257 mmol, 2.0 equiv.) at room temperature and then brought to 85 ºC and stirred for 18 h. After such time, the reaction mixture was diluted with EtOAc (50 mL), and washed with 5% aqueous NaOH (25 mL × 2), brine (25 mL) dried over

Na_2SO_4, filtered, and concentrated under reduced pressure. The resulting crude residue was purified by neutral alumina flash column (eluent: 5 % MeOH in DCM) to afford the title compound (**13**) as light-yellow solid (39 mg, 57%). TLC (N-Alumina) R_f = 0.50 (5:95 MeOH/DCM; UV). ^{1}H NMR (500 MHz, $CDCl_3$) δ 8.82 (s, 1H, H5), 8.46 (s, 1H, H4), 8.41 (s, 1H, H3’), 8.16 (s, 1H, H3’’), 7.95 – 7.90 (m, 1H, H5’), 7.55 (s, 1H, H3’’), 7.52 (d, *J* = 8.0 Hz, 1H, H6’), 4.49 (q, *J* = 7.0 Hz, 2H, CH_2), 3.90 (t, *J* = 5.9 Hz, 2H, H3’’’), 3.62 (m, 2H, H7’’’), 3.01 (m, 4H, H5’’’, H6’’’), 2.96 (t, *J* = 5.6 Hz, 2H, $H1^{iv}$), 2.87 (t, *J* = 5.5 Hz, 2H, $H2^{iv}$), 2.63 (s, 6H, CH_3, CH_3) 1.36 (t, *J* = 7.0 Hz, 3H, CH_3); ^{13}C NMR (126 MHz, $CDCl_3$) δ 161.8 (C=O), 158.9, 155.6, 153.6, 150.1, 143.4, 139.0, 137.9, 136.7, 136.0, 134.8, 132.4, 128.3, 125.0 (aromatic), 69.7 (C3’’’), 60.0 ($C2^{iv}$), 55.2 (C4’’’), 54.4 ($C1^{iv}$), 52.5 (C6’’’), 47.6 (CH_3), 39.2 (C7’’’), 36.5 (CH_2), 21.9 (CH_3), 13.1 (CH_3). HRMS-ESI (*m/z*): $[M + H]^+$ calculated for $[C_{28}H_{27}N_{10}O_2]^+$: 535.2313, found 535.2315.

5. Catalog of Spectra

^{1}H NMR (600 MHz, $CDCl_3$) of (((3-(bromomethyl)but-3-en-1-yl)oxy)methyl)benzene (**16**)

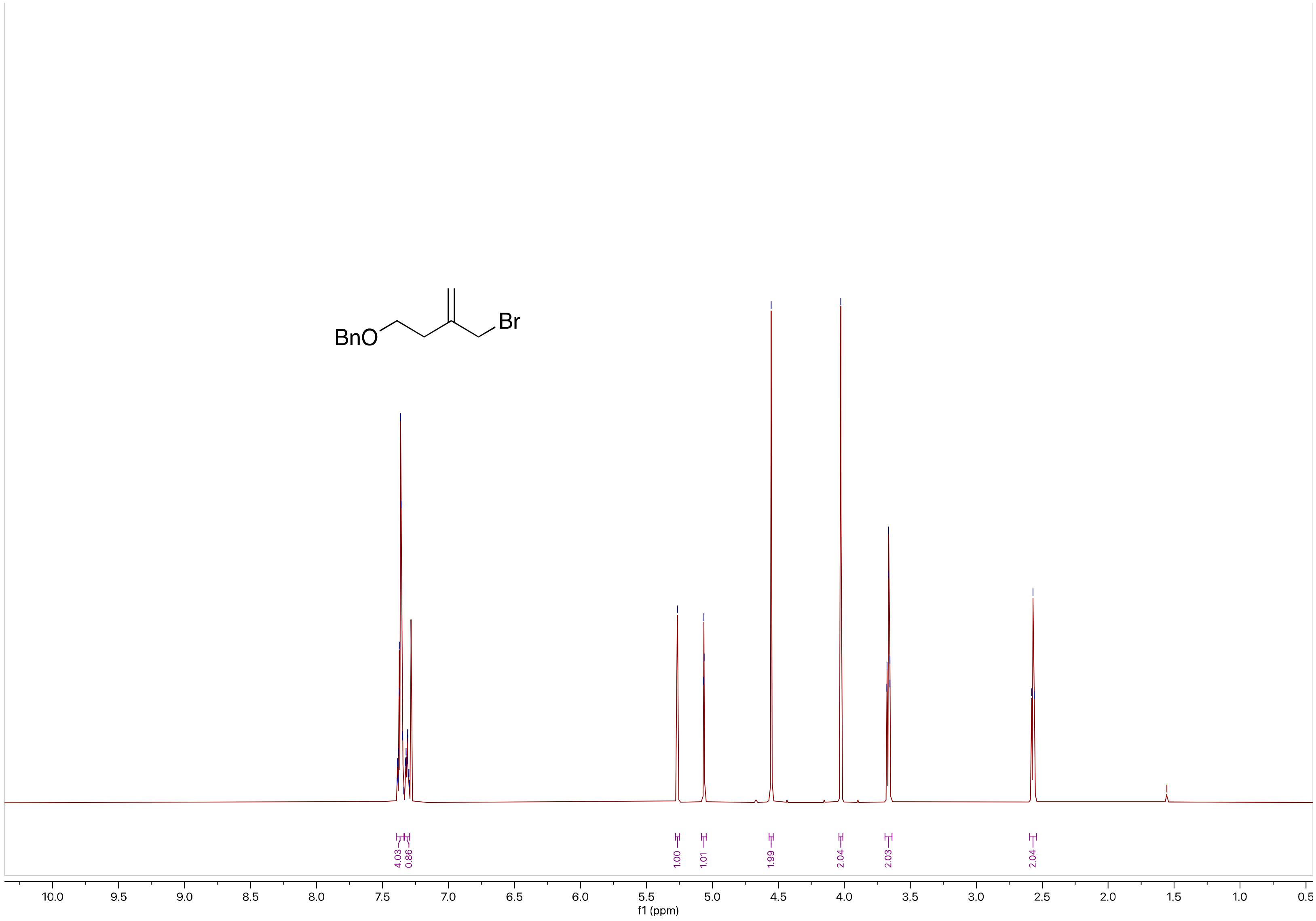

^{13}C NMR (151 MHz, $CDCl_3$) of (((3-(bromomethyl)but-3-en-1-yl)oxy)methyl)benzene (**16**)

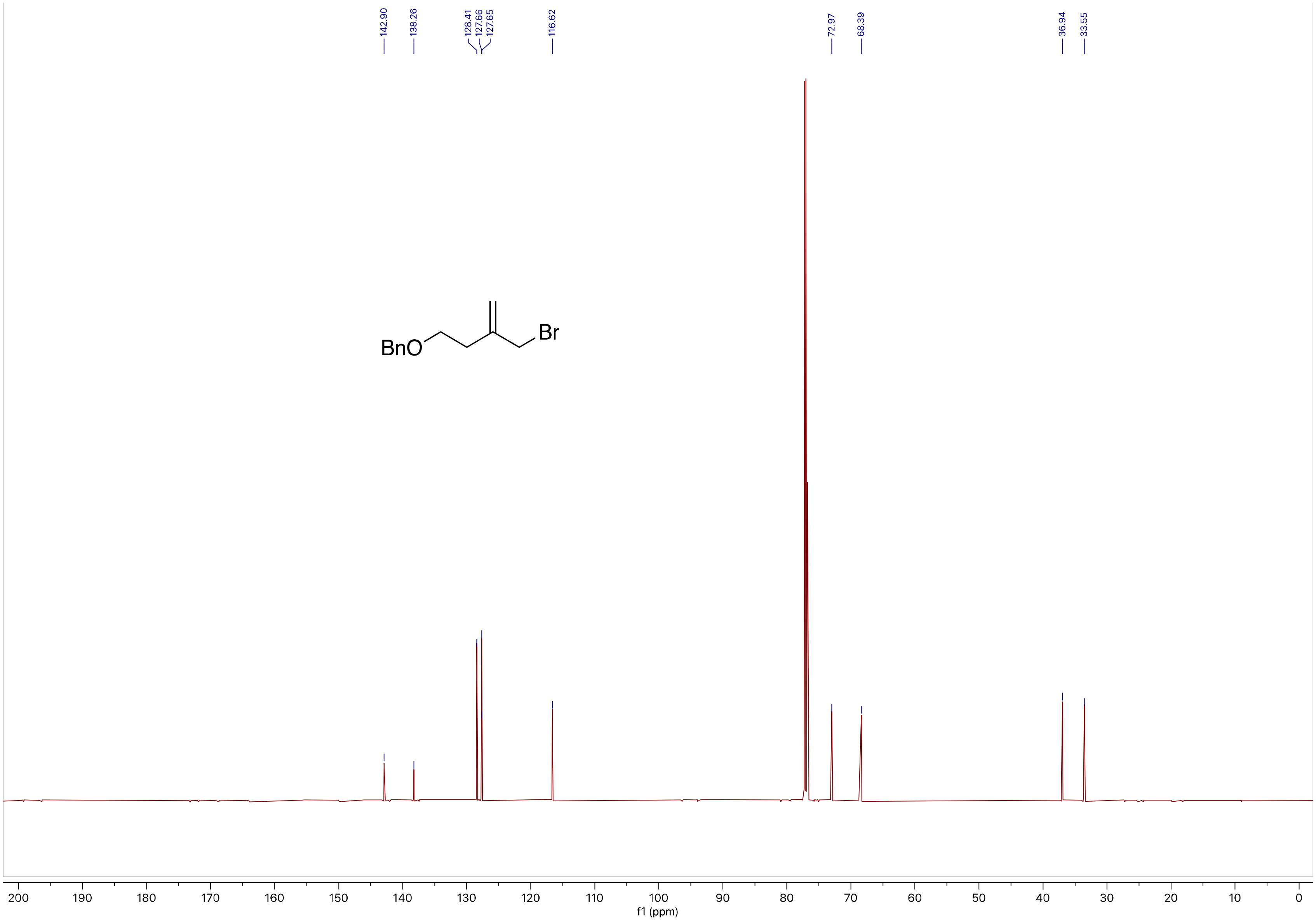

^{1}H NMR (600 MHz, $CDCl_3$) of *tert*-butyl (4-(benzyloxy)-2-methylenebutoxy)carbamate (**17**)

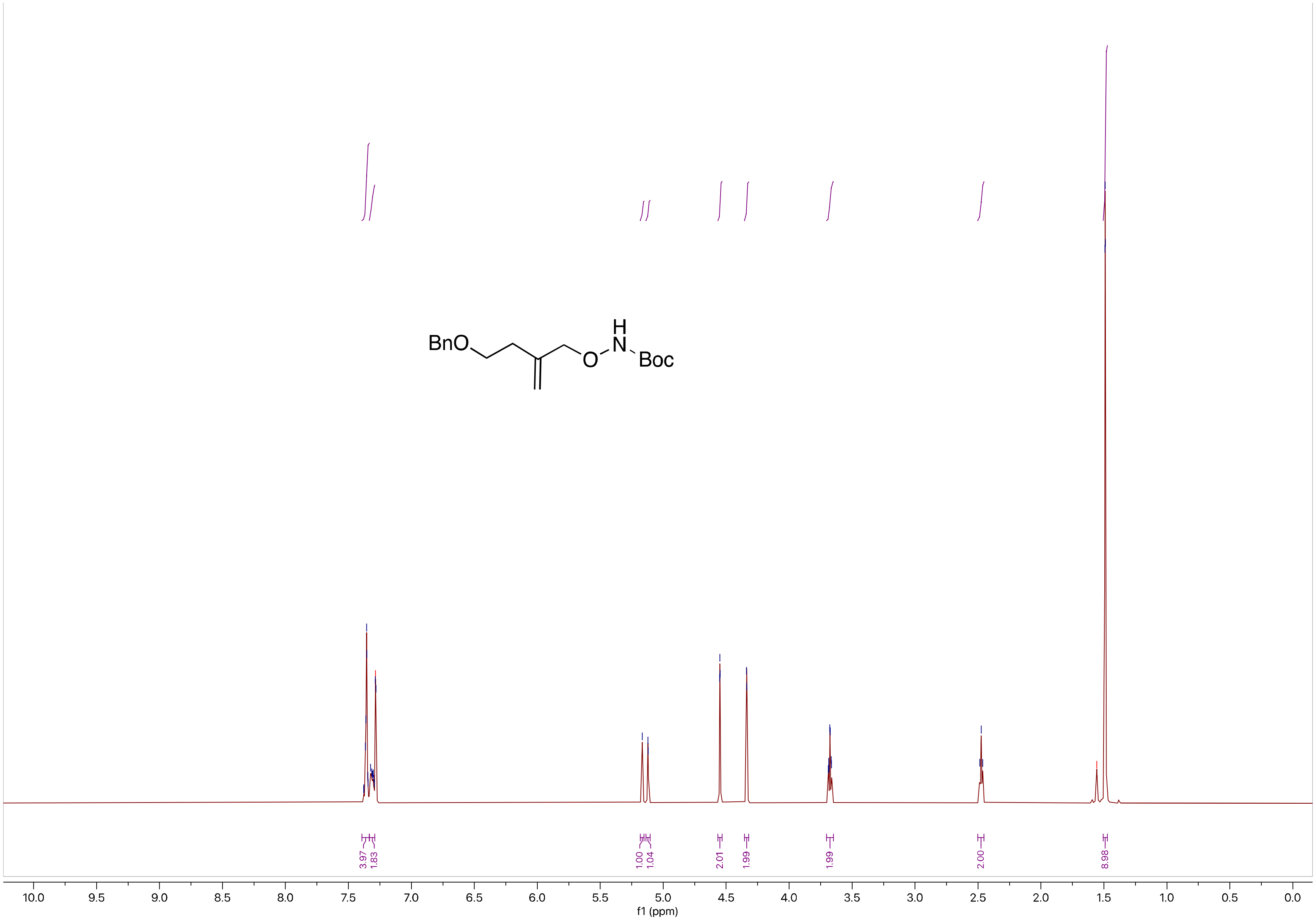

^{13}C NMR (151 MHz, $CDCl_3$) of *tert*-butyl (4-(benzyloxy)-2-methylenebutoxy)carbamate (**17**)

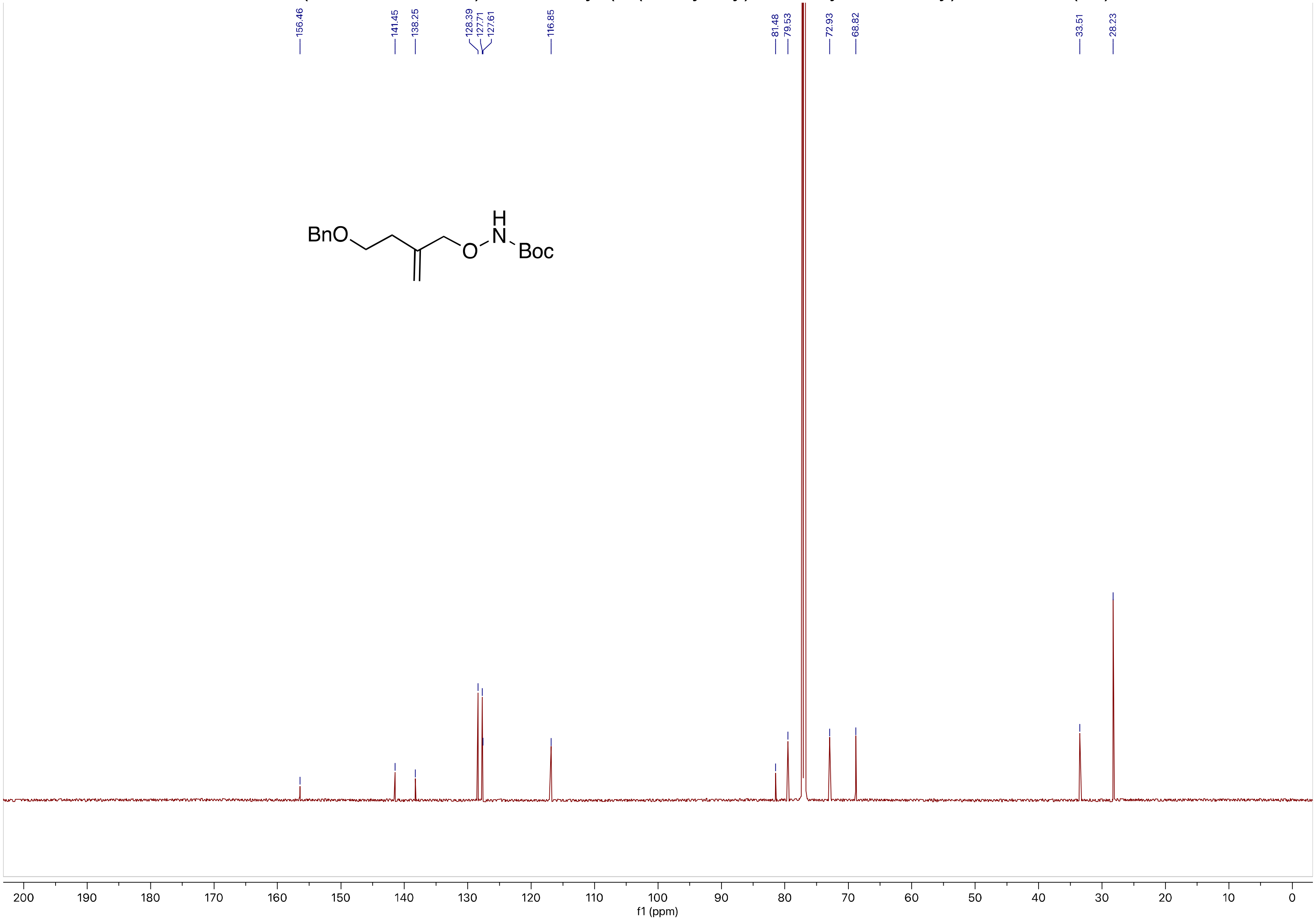

HSQC ($CDCl_3$) spectrum of *tert*-butyl (4-(benzyloxy)-2-methylenebutoxy)carbamate (**17**)

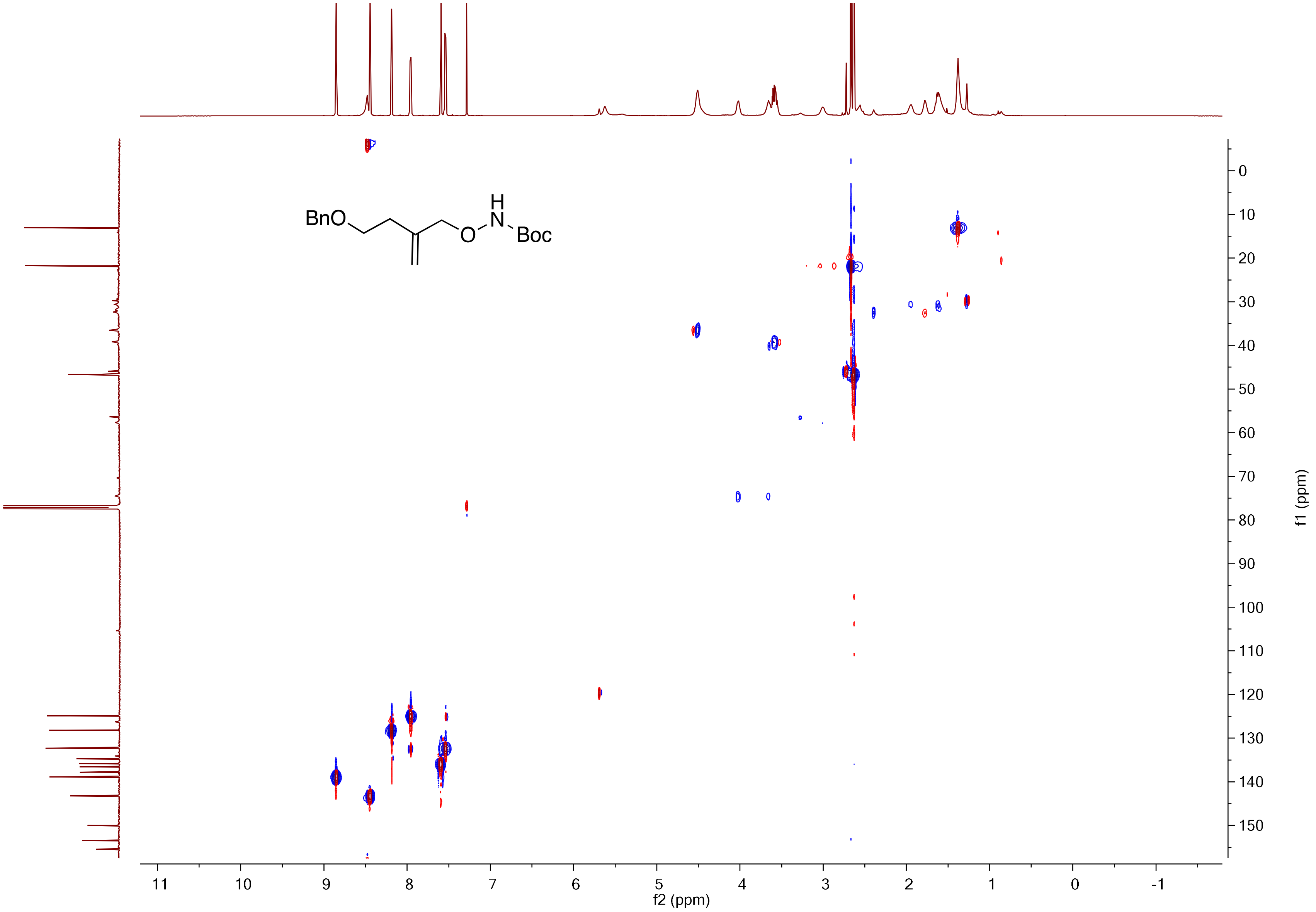

HMBC ($CDCl_3$) spectrum of *tert*-butyl (4-(benzyloxy)-2-methylenebutoxy)carbamate (**17**)

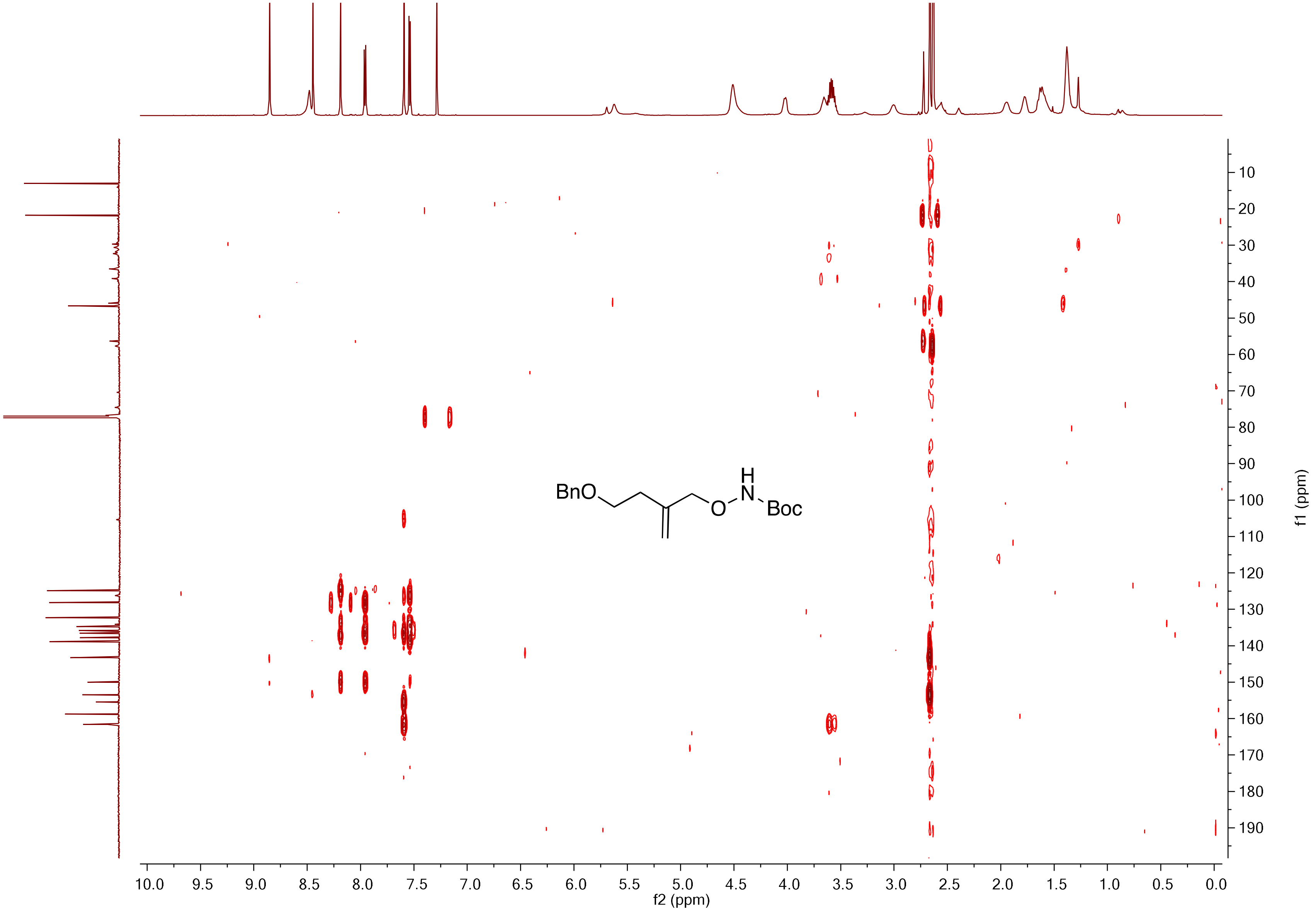

COSY ($CDCl_3$) spectrum of *tert*-butyl (4-(benzyloxy)-2-methylenebutoxy)carbamate (**17**)

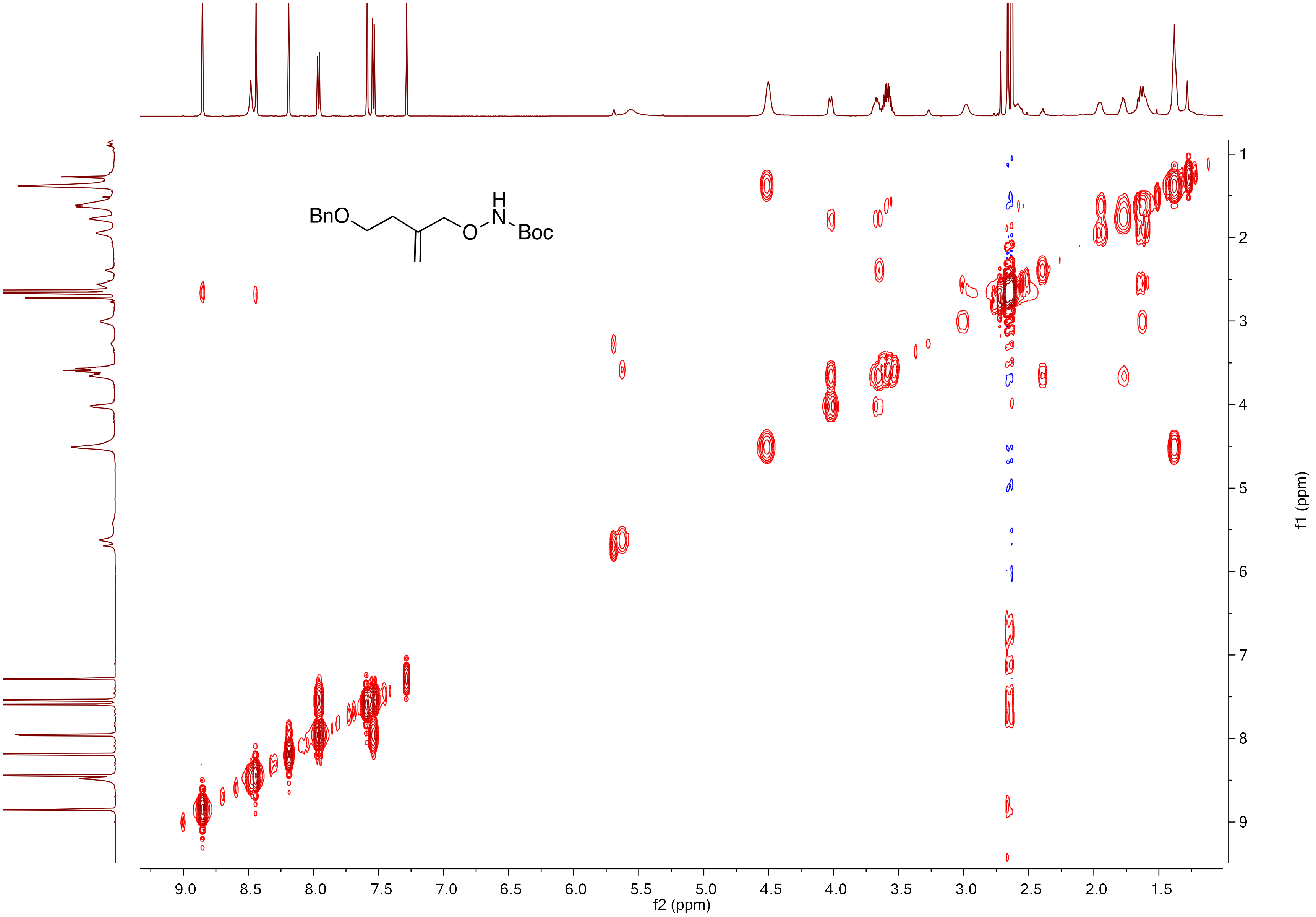

^{1}H NMR (600 MHz, $CDCl_3$) of *tert*-butyl allyl(4-(benzyloxy)-2-methylenebutoxy)carbamate (**18**)

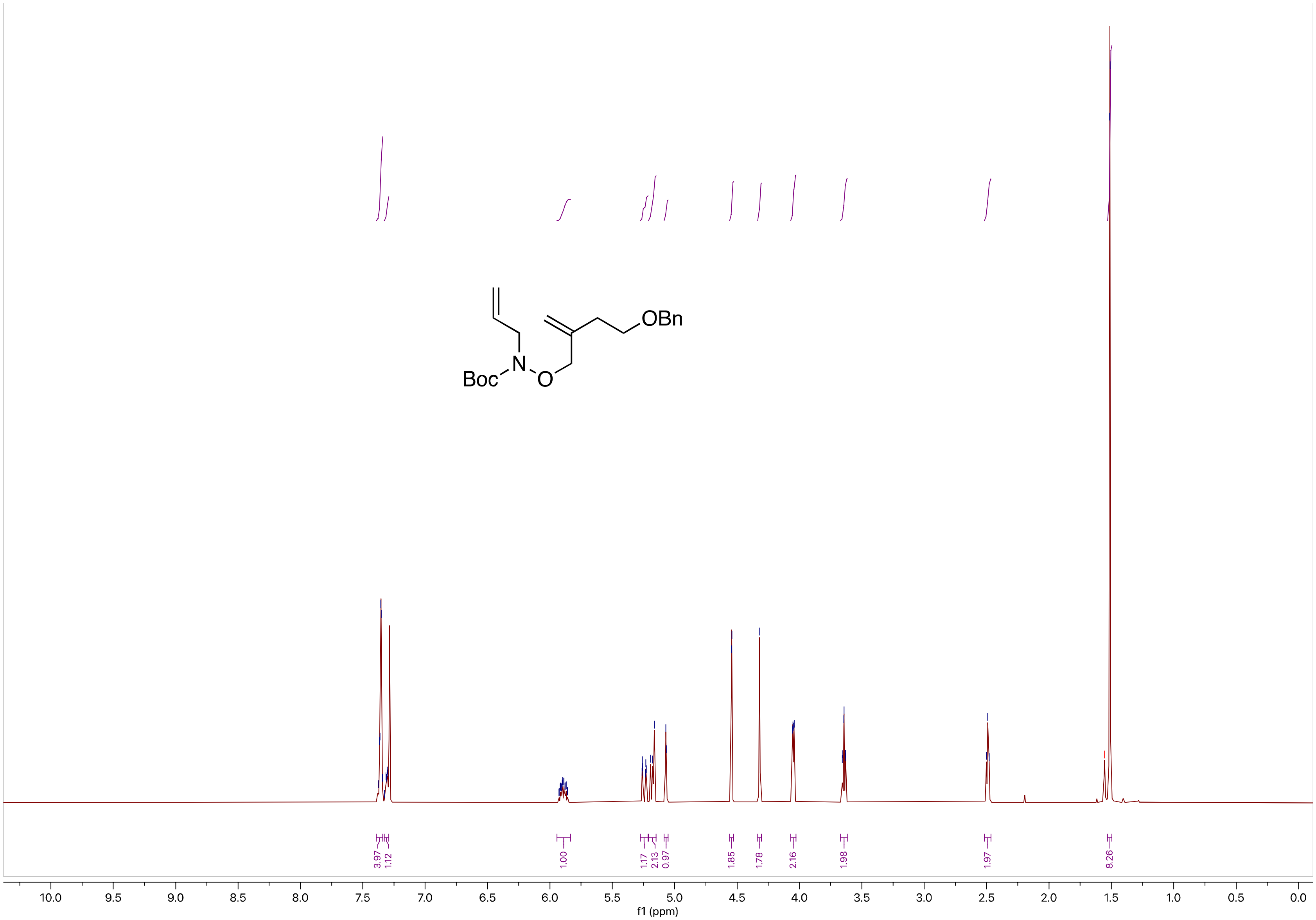

^{13}C NMR (151 MHz, $CDCl_3$) of *tert*-butyl allyl(4-(benzyloxy)-2-methylenebutoxy)carbamate (**18**)

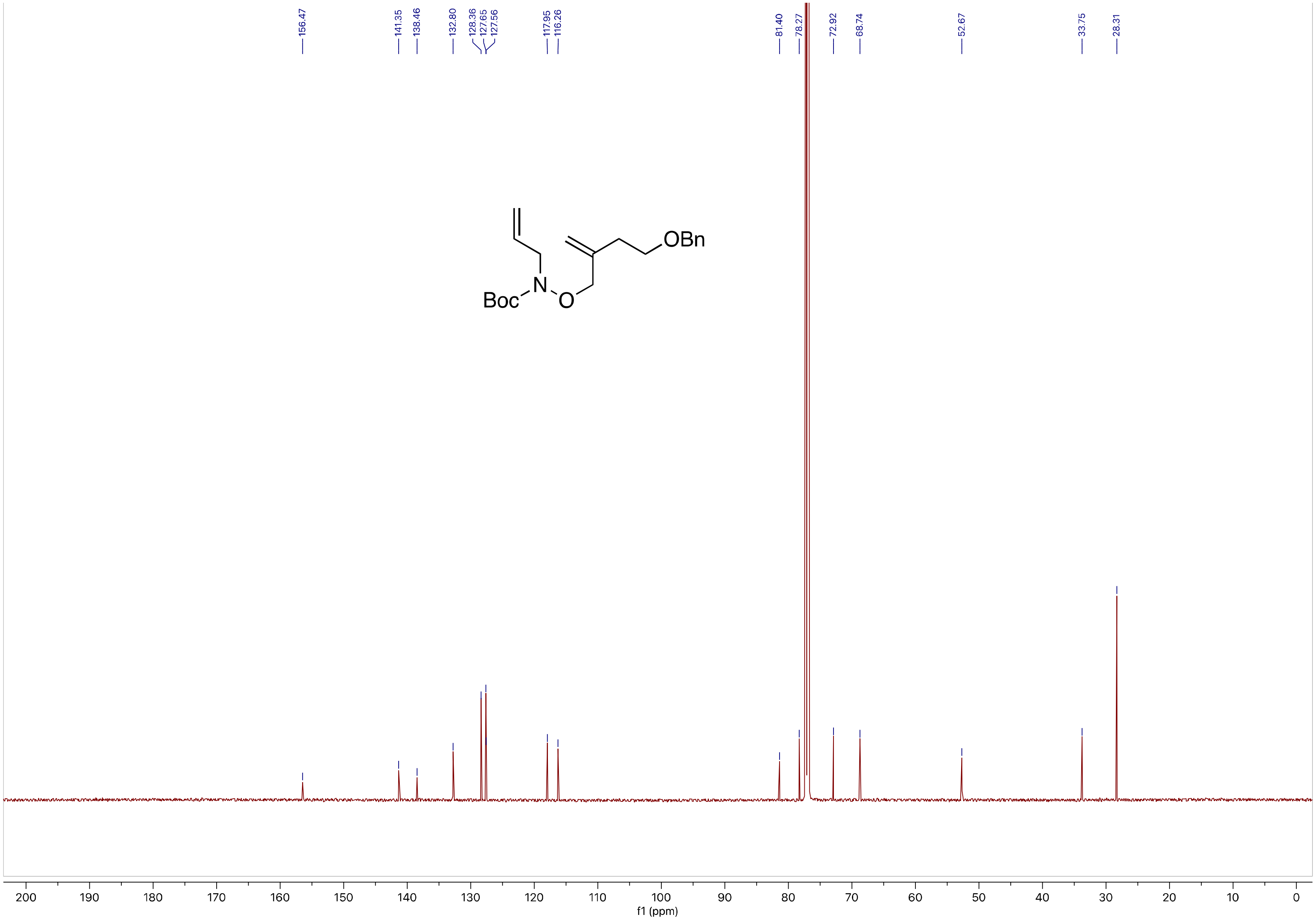

HSQC ($CDCl_3$) spectrum of *tert*-butyl allyl(4-(benzyloxy)-2-methylenebutoxy)carbamate (**18**)

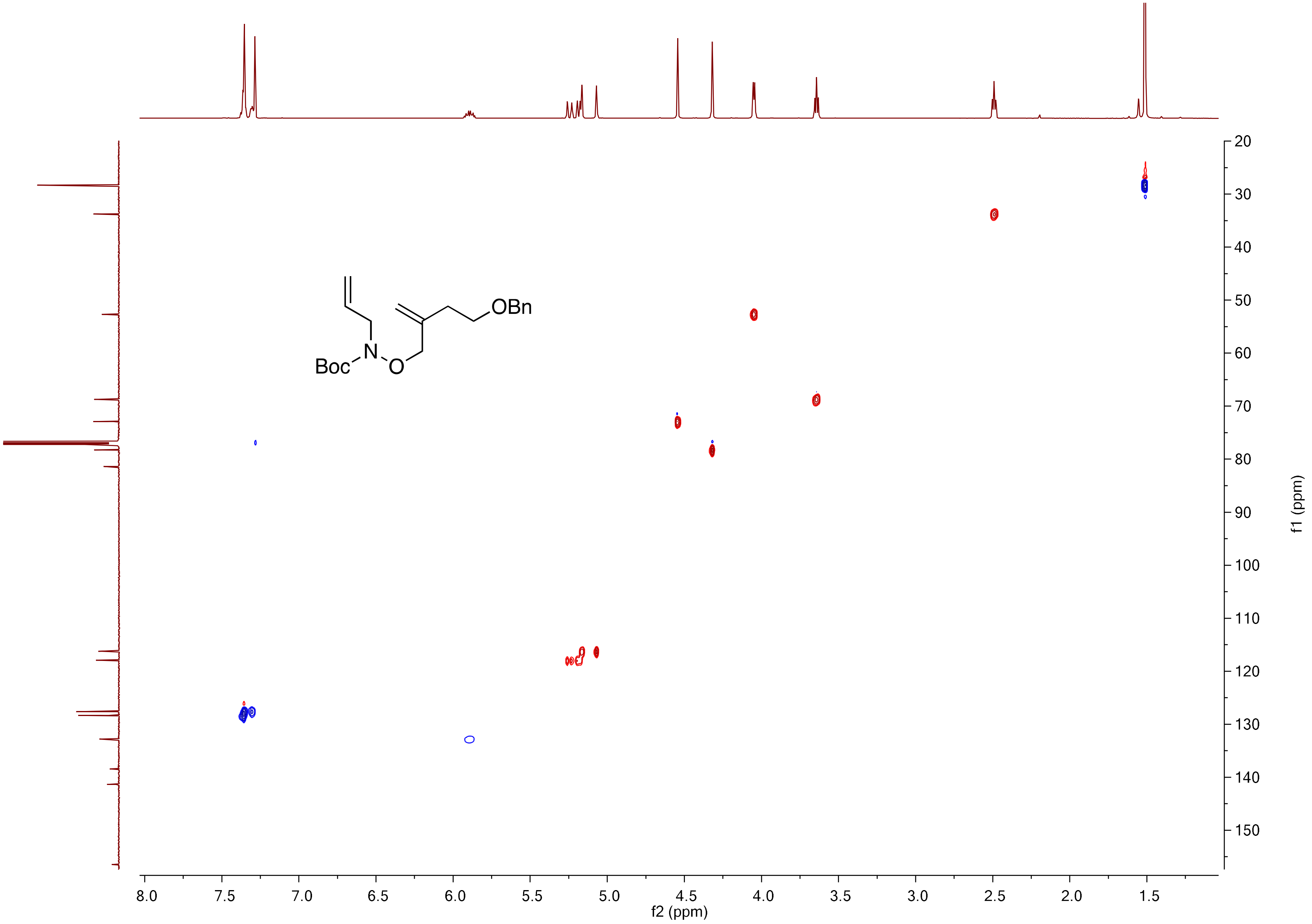

COSY ($CDCl_3$) spectrum of *tert*-butyl allyl(4-(benzyloxy)-2-methylenebutoxy)carbamate (**18**)

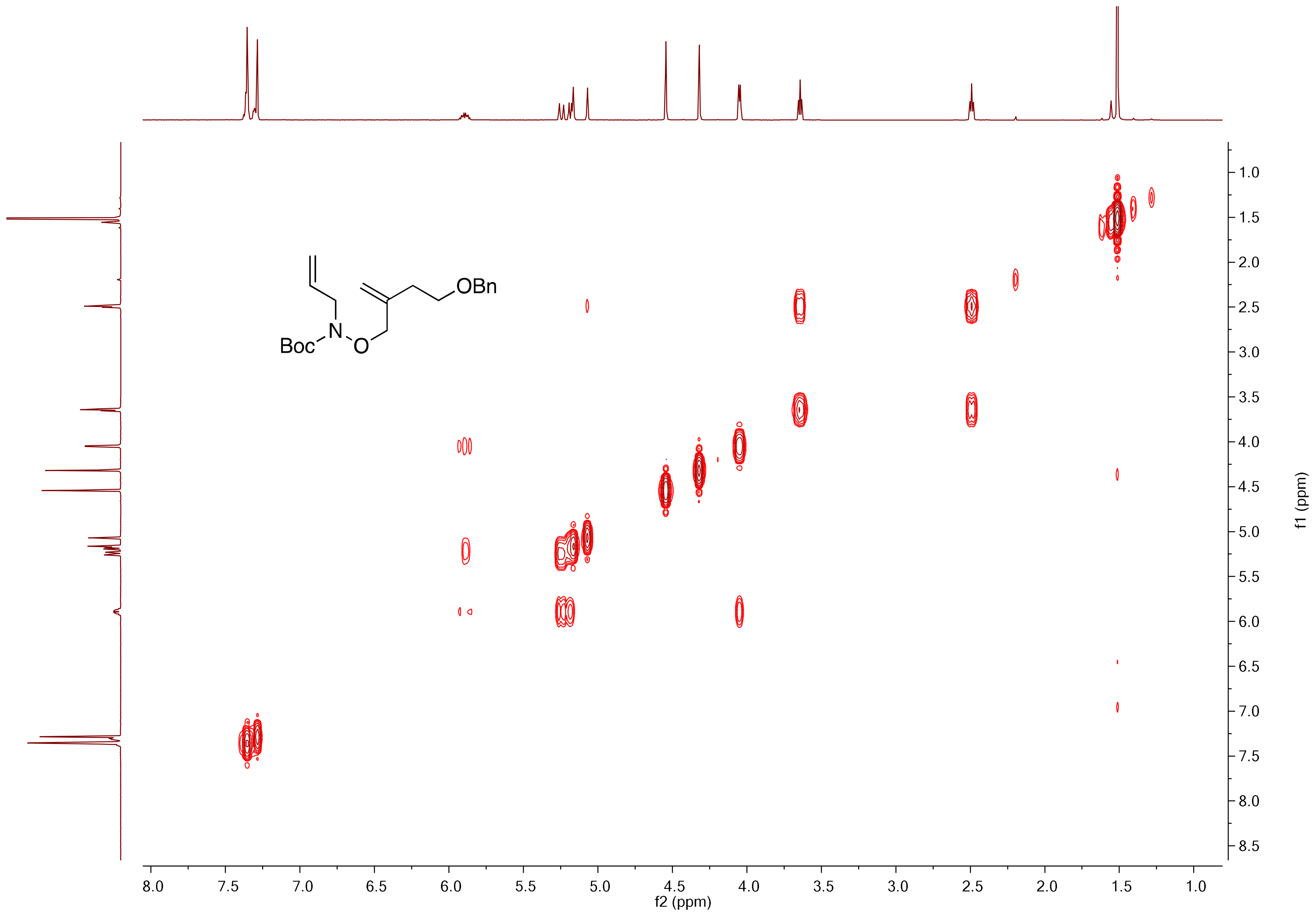

The three-bond ^{1}H-^{15}N HMBC ($CDCl_3$) correlations of *tert*-butyl allyl(4-(benzyloxy)-2-methylenebutoxy)carbamate (**18**)

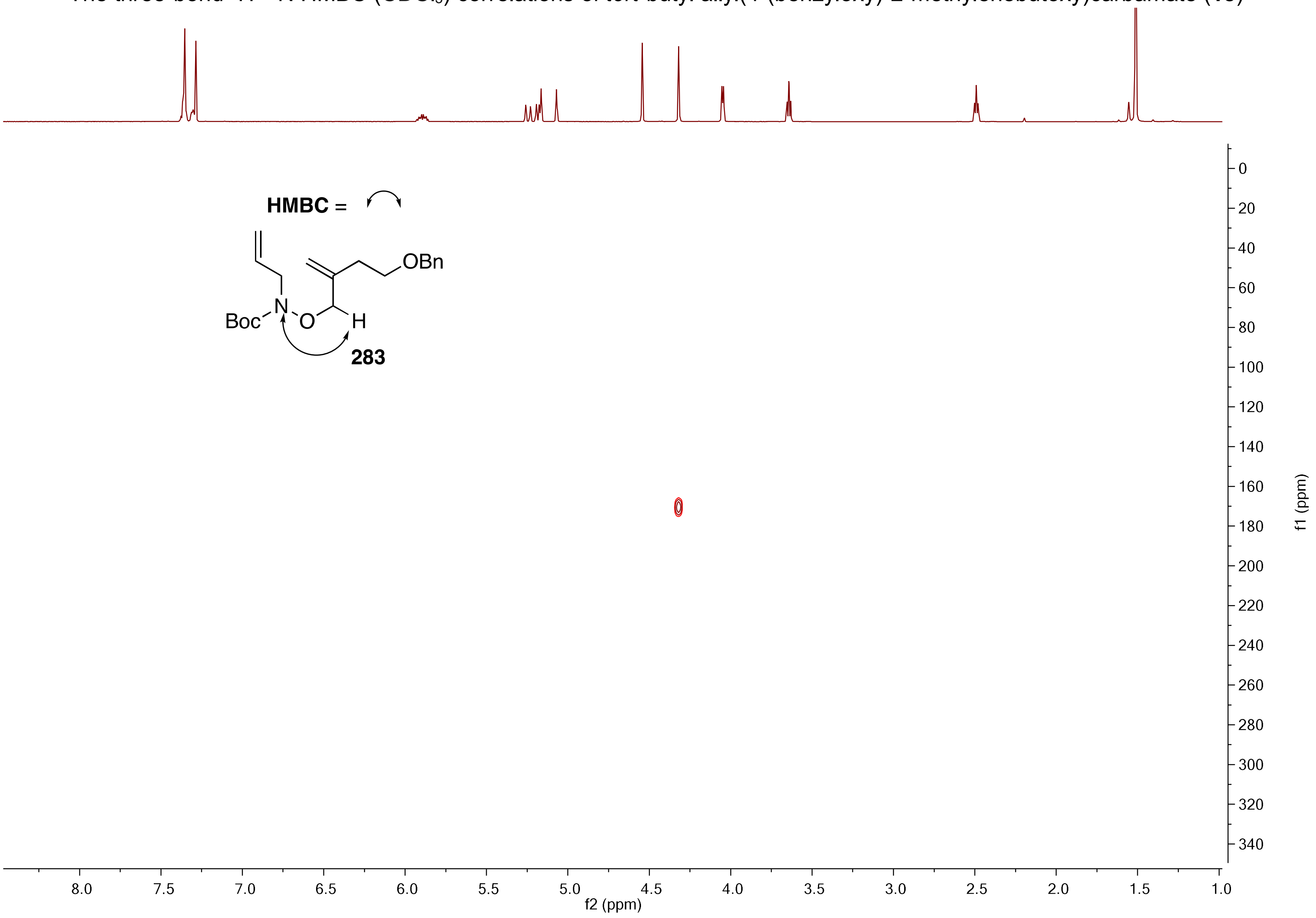

^{1}H NMR (600 MHz, $CDCl_3$) of *tert*-butyl 5-(2-(benzyloxy)ethyl)-3,6-dihydro-2*H*-1,2-oxazine-2-carboxylate (**19**)

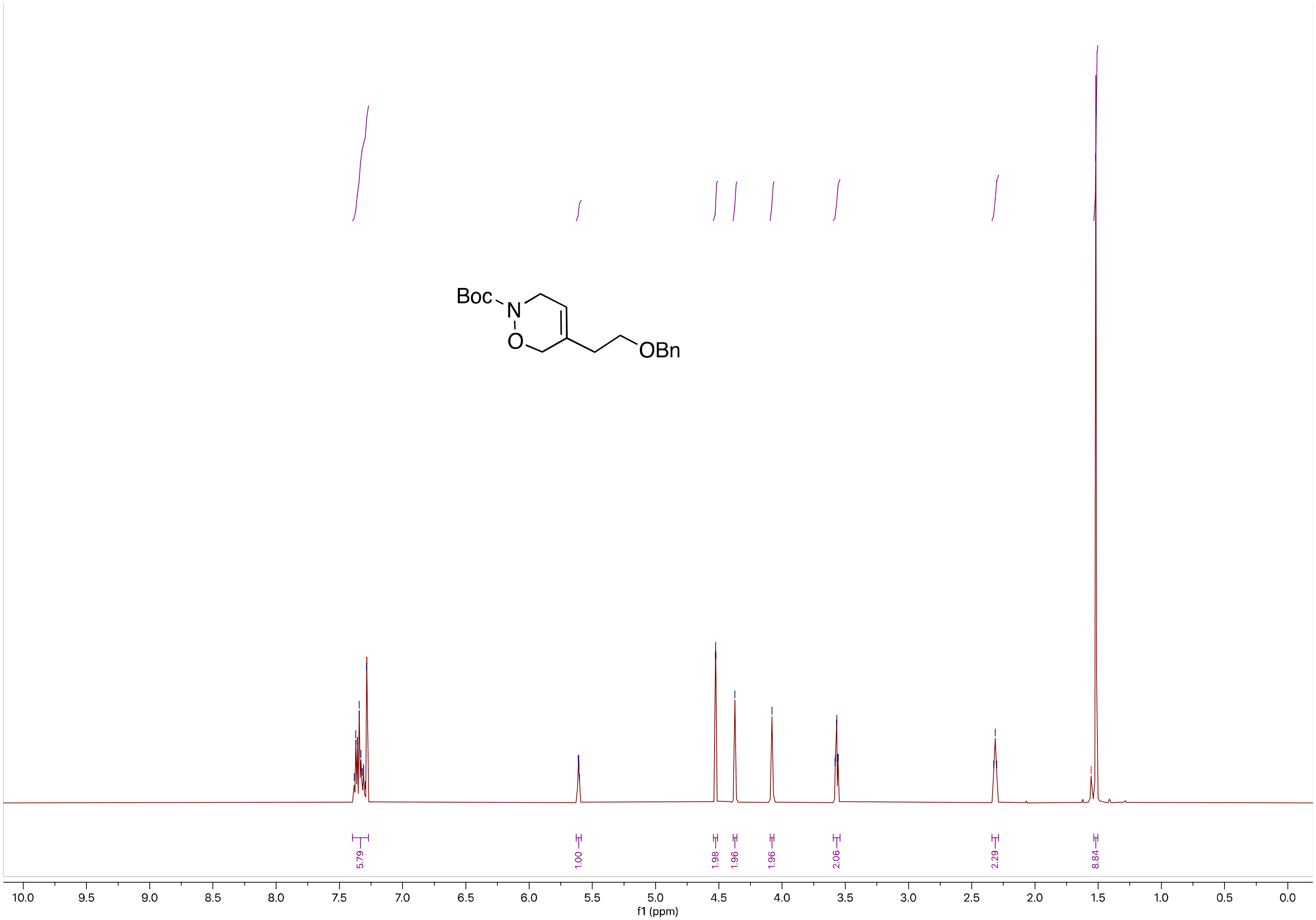

^{13}C NMR (151 MHz, $CDCl_3$) of *tert*-butyl 5-(2-(benzyloxy)ethyl)-3,6-dihydro-2*H*-1,2-oxazine-2-carboxylate (**19**)

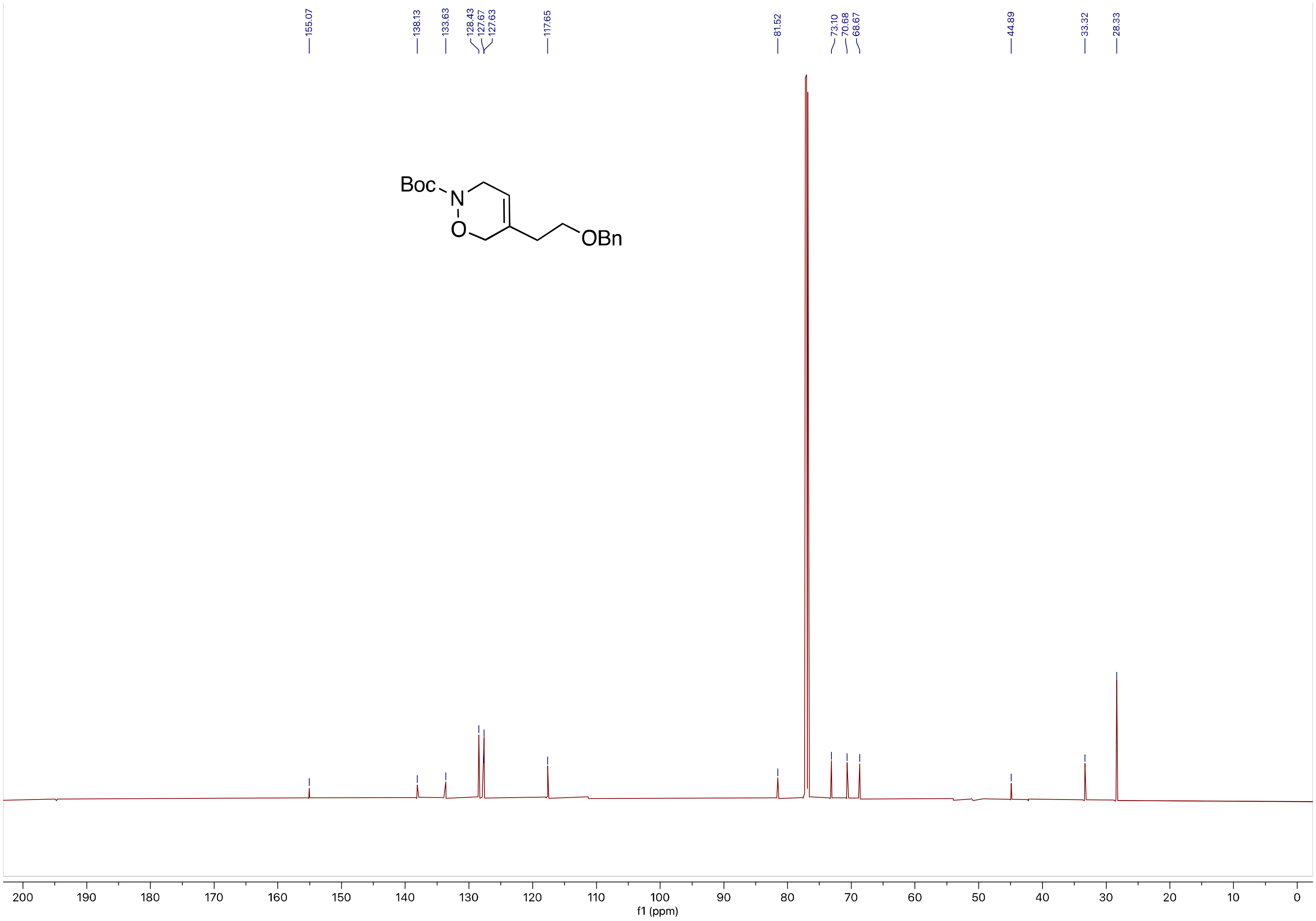

HSQC ($CDCl_3$) spectrum of *tert*-butyl 5-(2-(benzyloxy)ethyl)-3,6-dihydro-2*H*-1,2-oxazine-2-carboxylate (**19**)

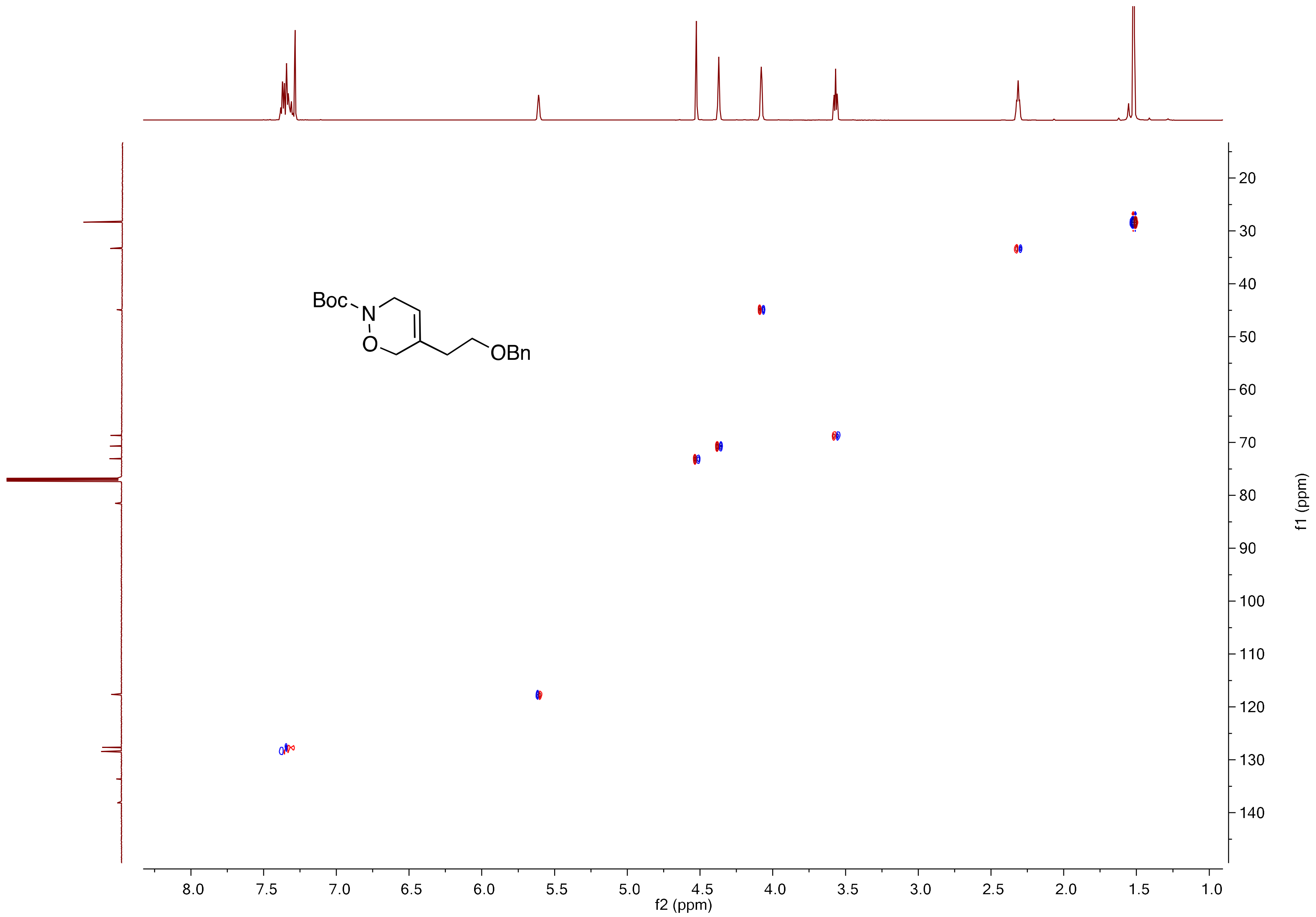

^{1}H NMR (600 MHz, $CDCl_3$) of *tert*-butyl 5-(2-hydroxyethyl)-1,2-oxazinane-2-carboxylate (**20**)

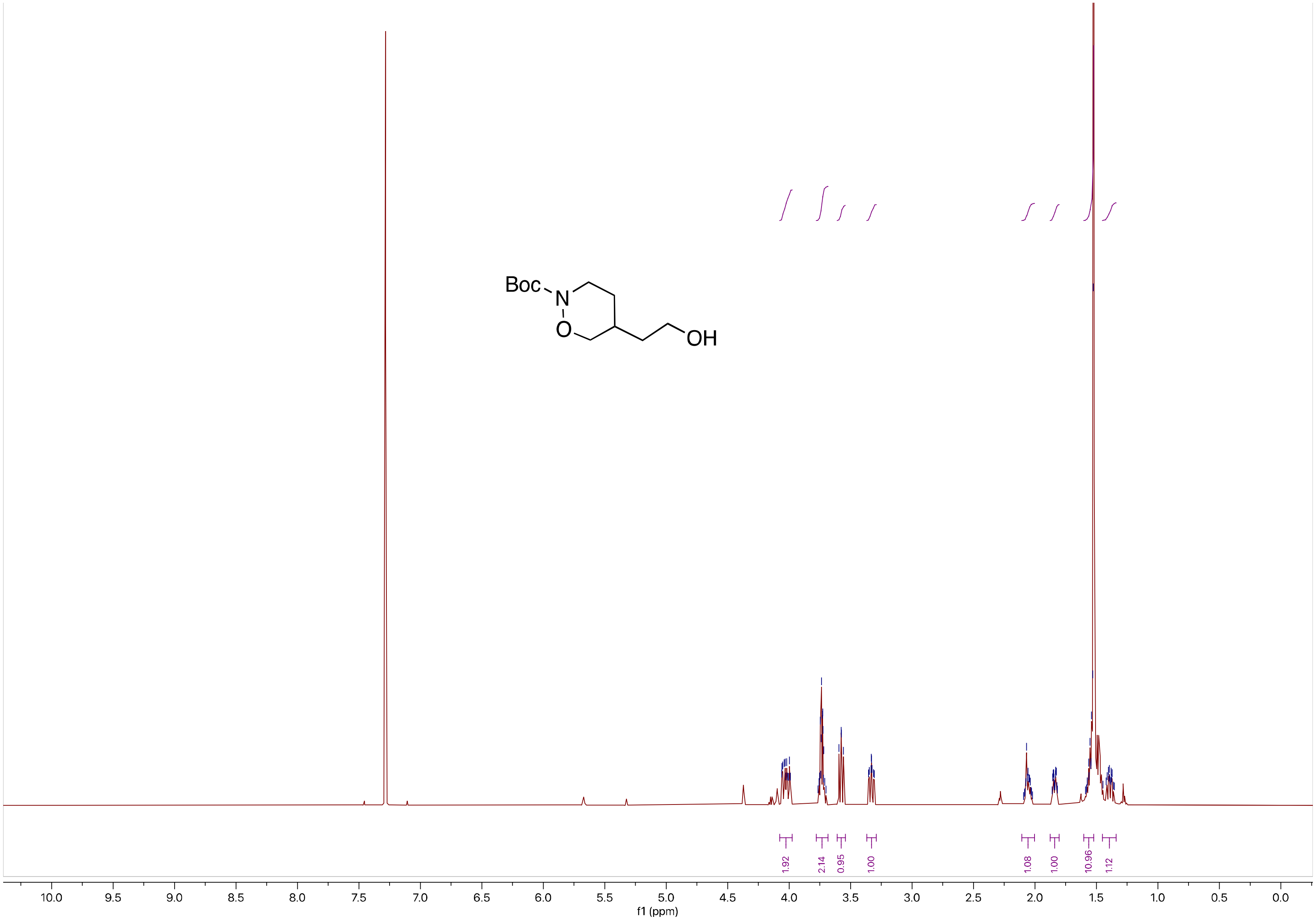

^{13}C NMR (151 MHz, $CDCl_3$) of *tert*-butyl 5-(2-hydroxyethyl)-1,2-oxazinane-2-carboxylate (**20**)

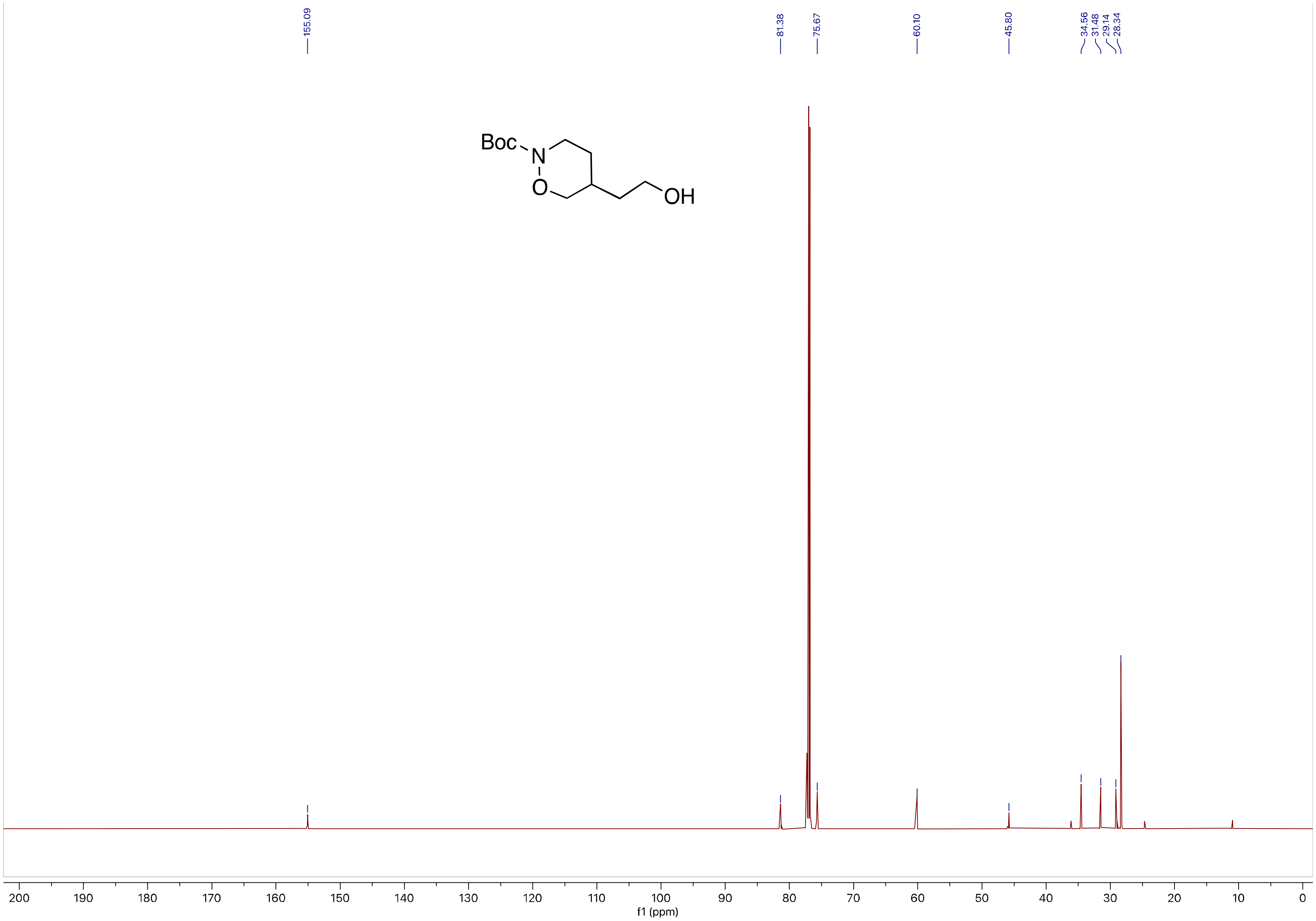

HSQC ($CDCl_3$) spectrum of *tert*-butyl 5-(2-hydroxyethyl)-1,2-oxazinane-2-carboxylate (**20**)

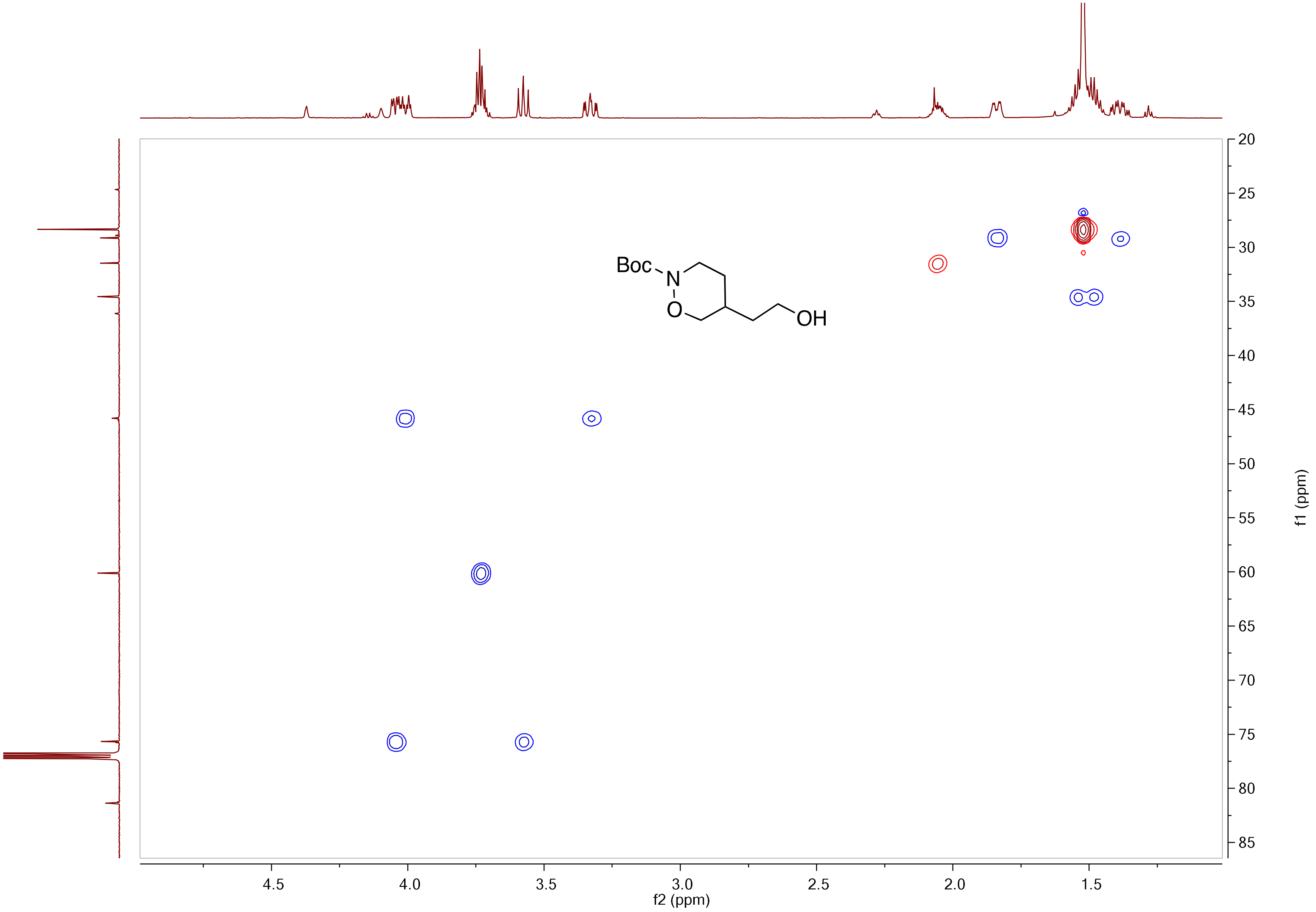

COSY ($CDCl_3$) spectrum of *tert*-butyl 5-(2-hydroxyethyl)-1,2-oxazinane-2-carboxylate (**20**)

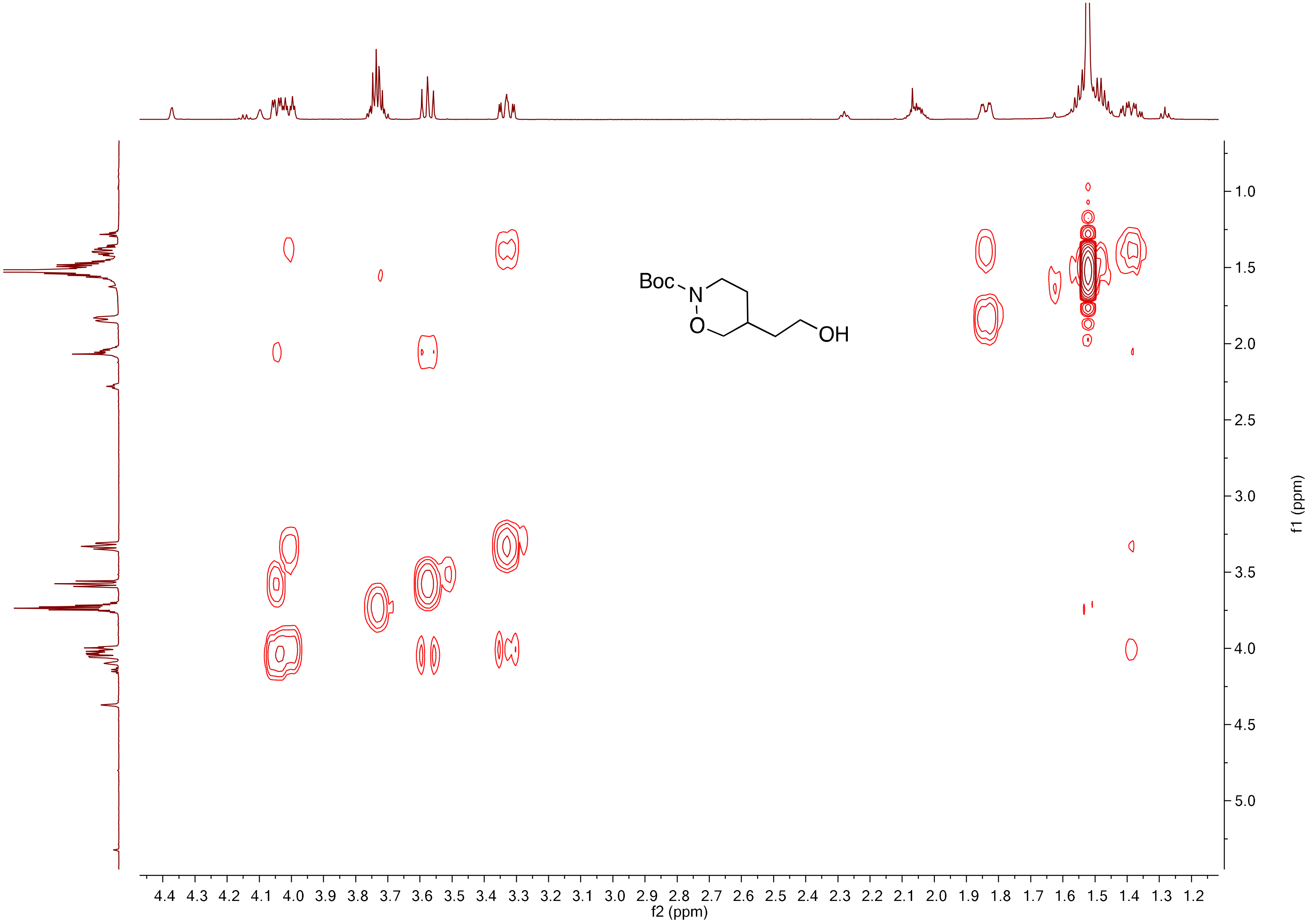

^1H NMR (600 MHz, $CDCl_3$) of *tert*-butyl 5-(2-bromoethyl)-1,2-oxazinane-2-carboxylate

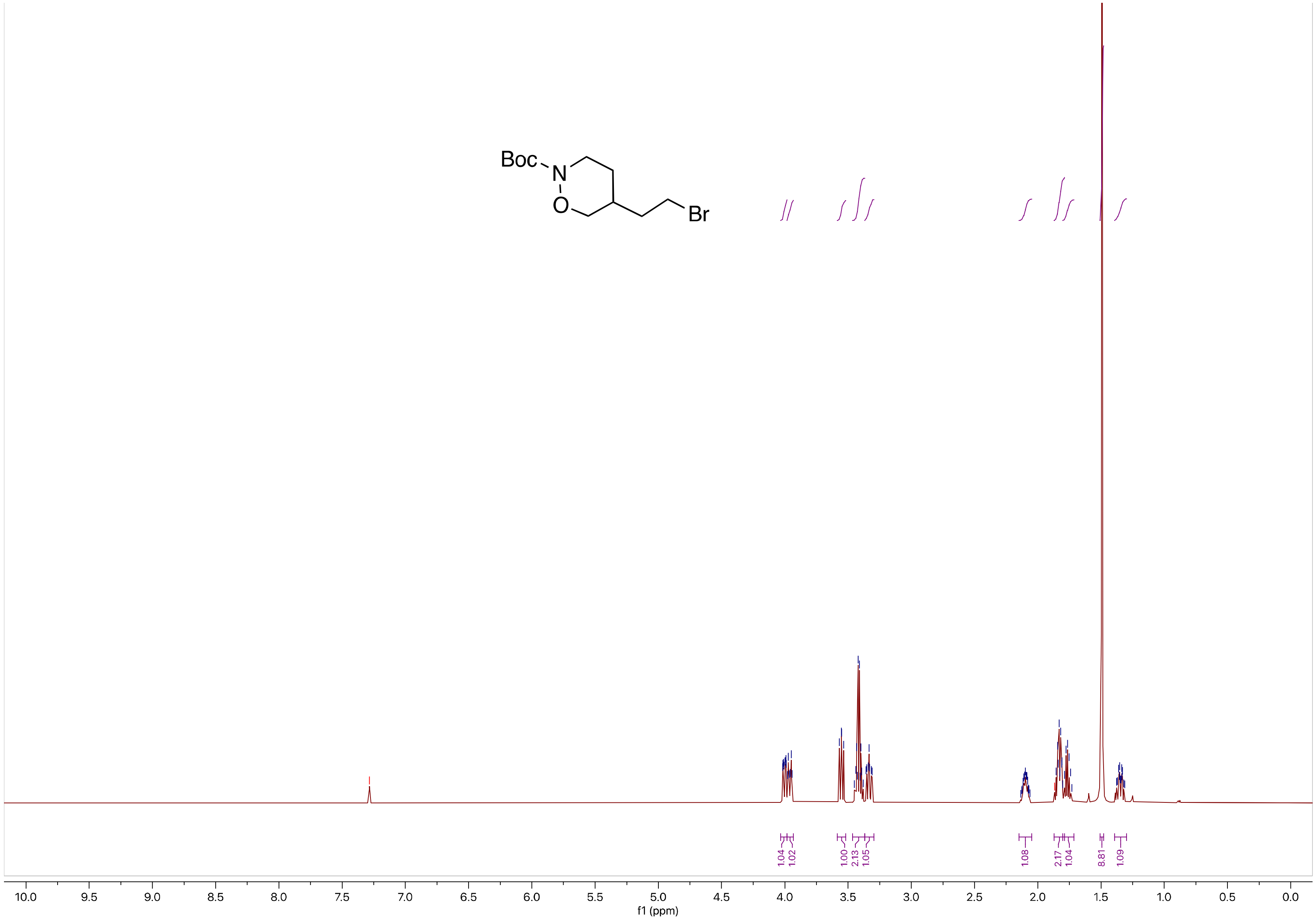

^{13}C NMR (151 MHz, $CDCl_3$) of *tert*-butyl 5-(2-bromoethyl)-1,2-oxazinane-2-carboxylate

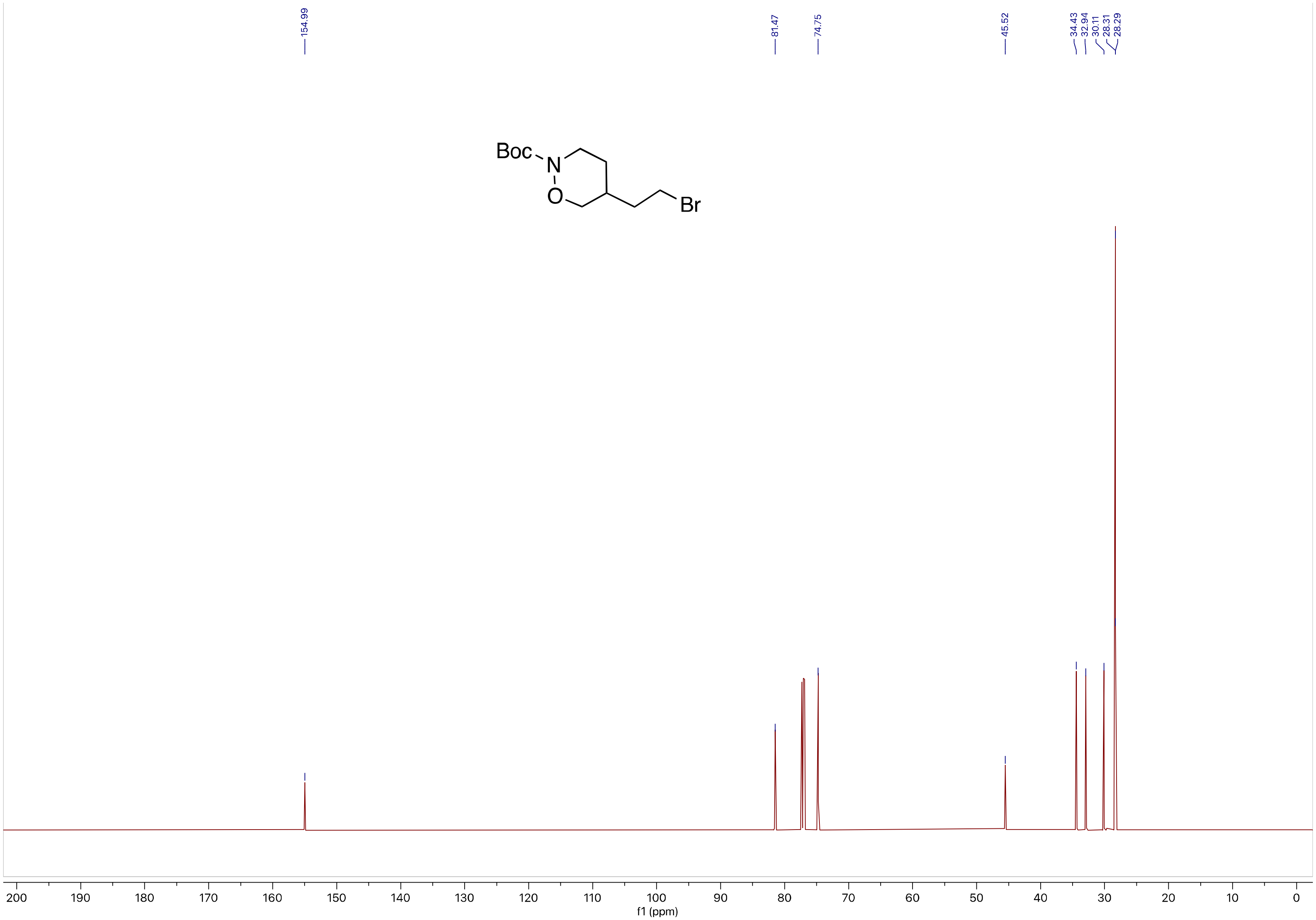

HSQC ($CDCl_3$) spectrum of *tert*-butyl 5-(2-bromoethyl)-1,2-oxazinane-2-carboxylate

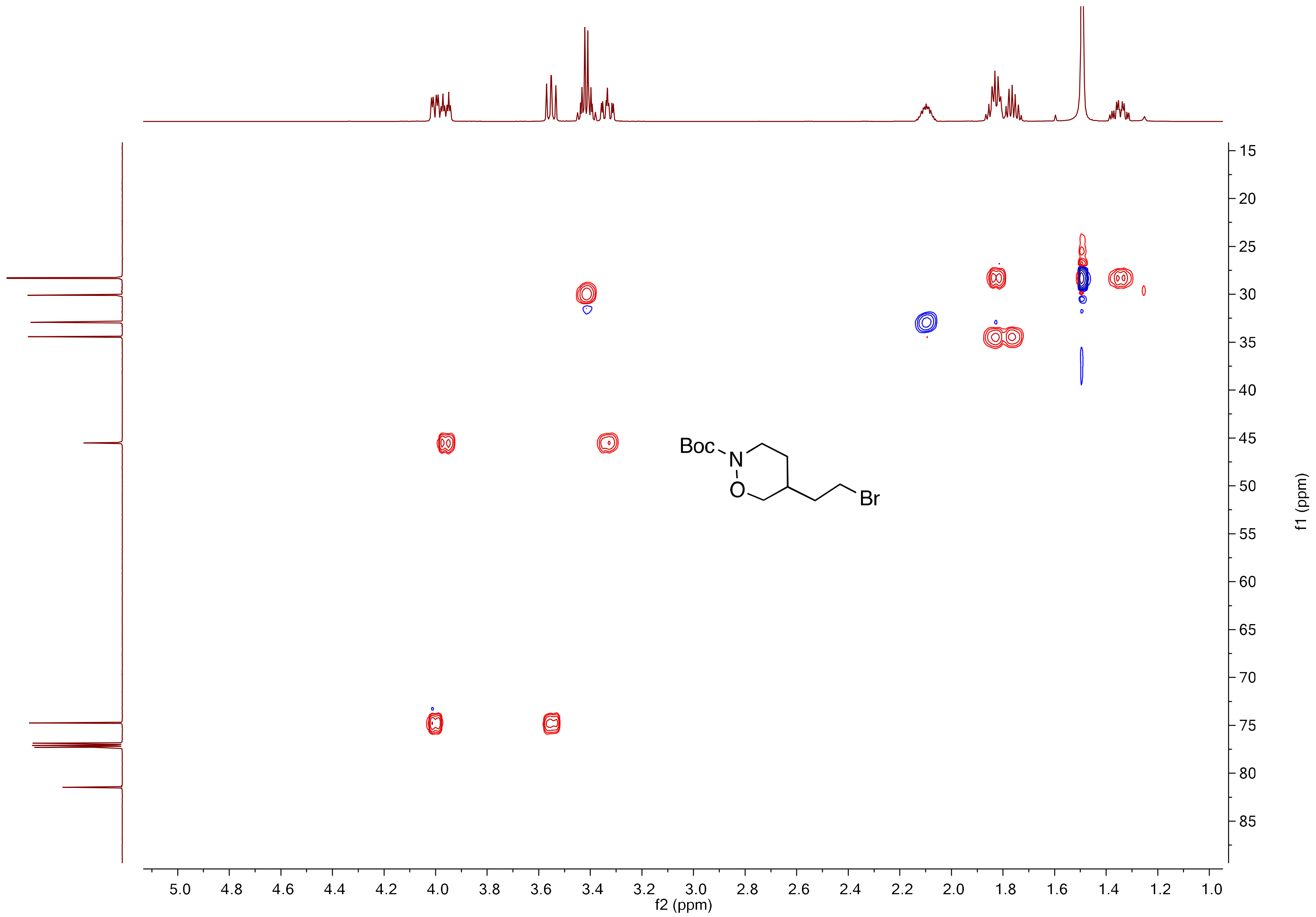

COSY ($CDCl_3$) spectrum of *tert*-butyl 5-(2-bromoethyl)-1,2-oxazinane-2-carboxylate

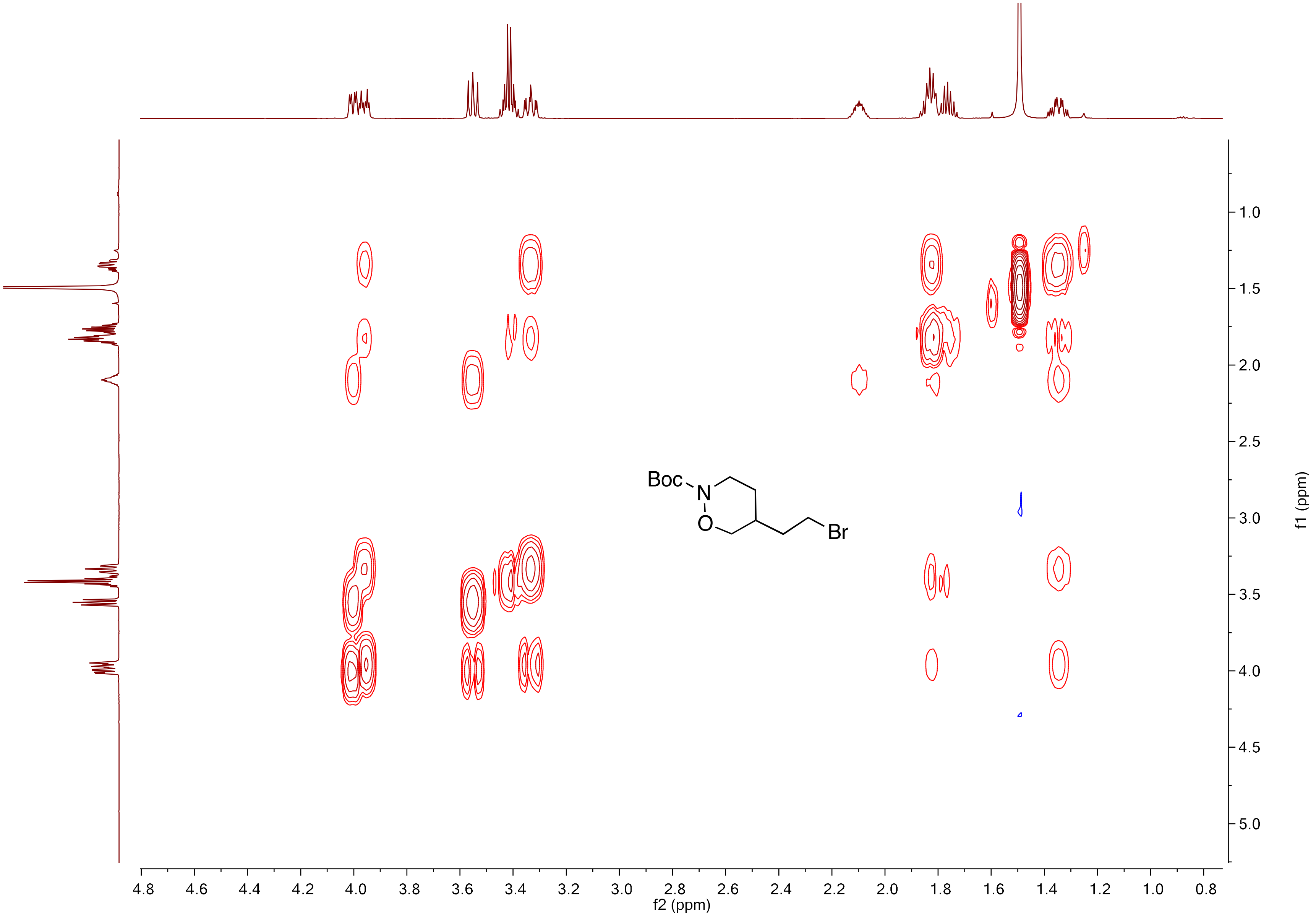

^1H NMR (600 MHz, $CDCl_3$) of *tert*-butyl 5-(2-azidoethyl)-1,2-oxazinane-2-carboxylate (**21**)

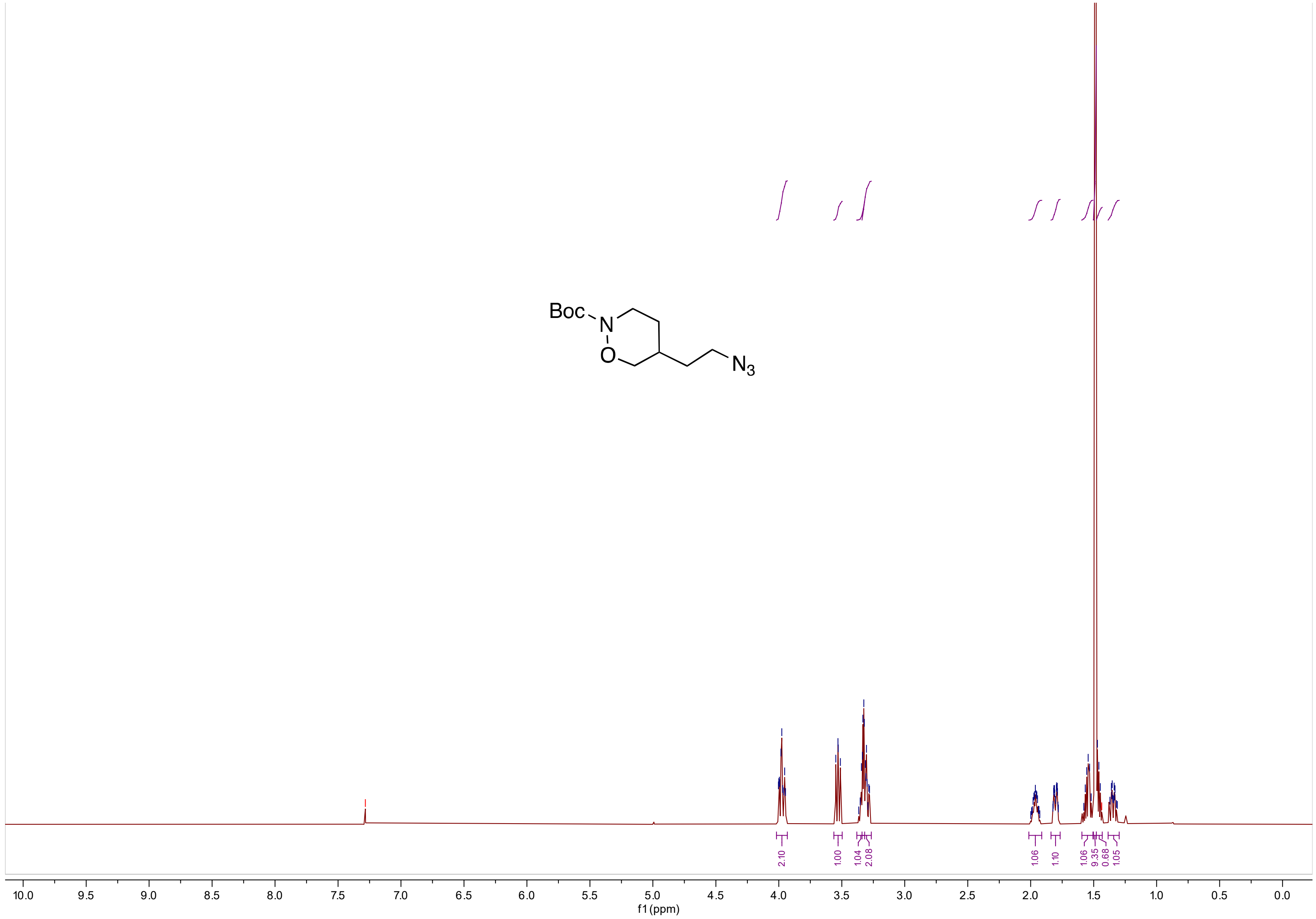

^{13}C NMR (151 MHz, $CDCl_3$) of *tert*-butyl 5-(2-azidoethyl)-1,2-oxazinane-2-carboxylate (**21**)

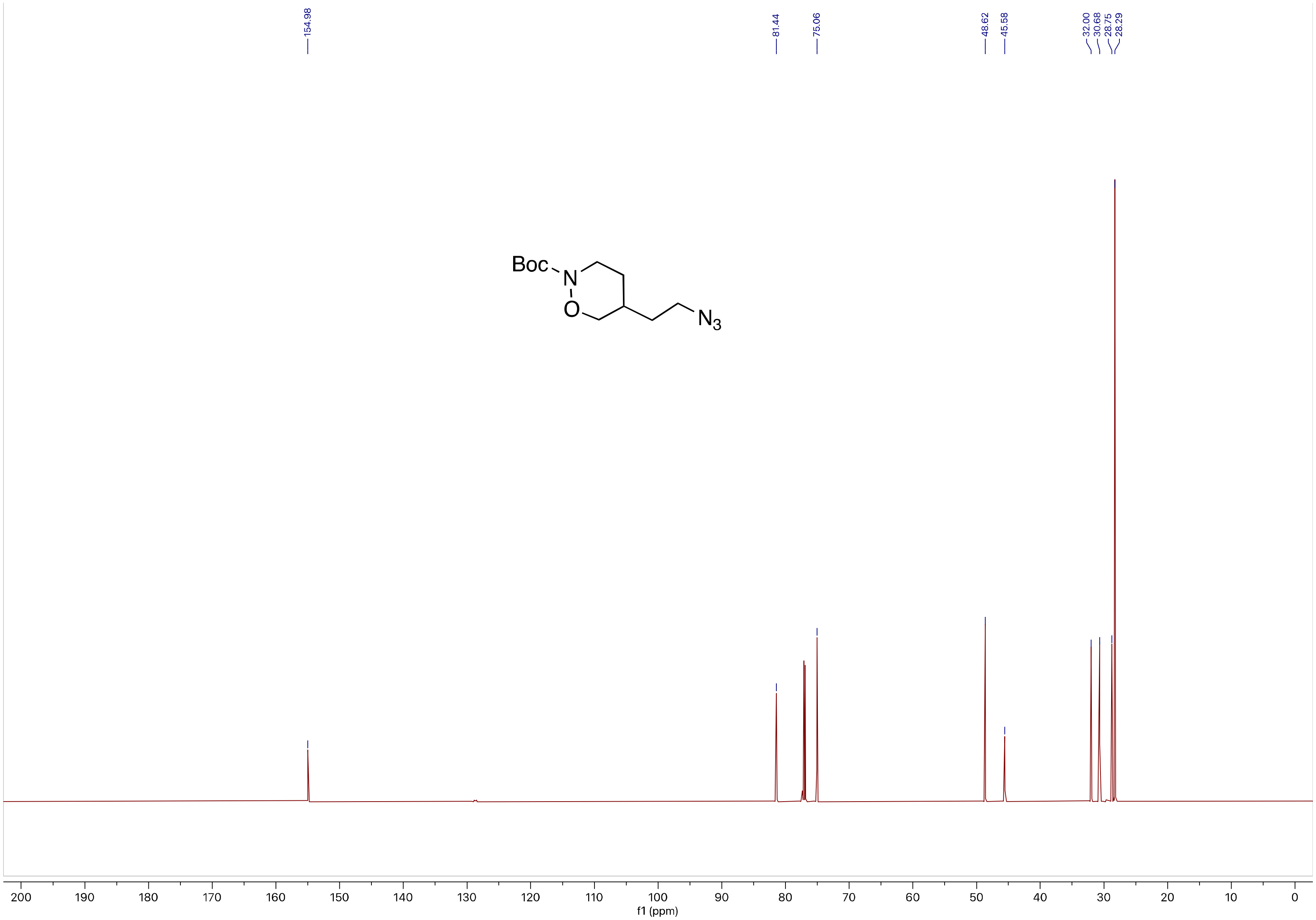

HSQC ($CDCl_3$) spectrum of *tert*-butyl 5-(2-azidoethyl)-1, 2-oxazinane-2-carboxylate (**21**)

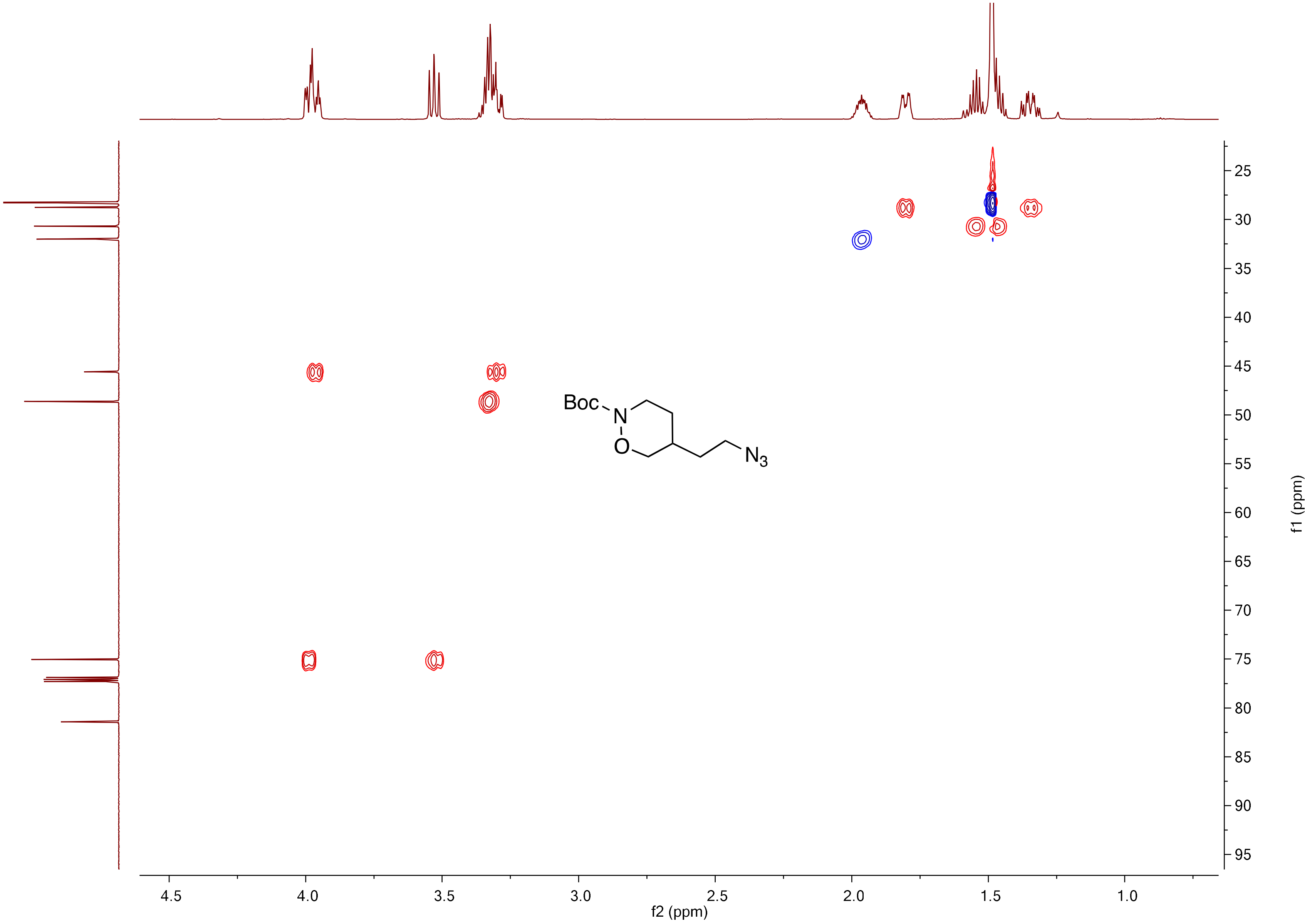

^{1}H NMR (600 MHz, $CDCl_3$) of 6-(2-chloro-4-(6-methylpyrazin-2-yl)phenyl)-8-ethyl-2-((2-(2-methyl-1,2-oxazinan-5-yl)ethyl)amino)pyrido[2,3-*d*]pyrimidin-7(8*H*)-one (**8**)

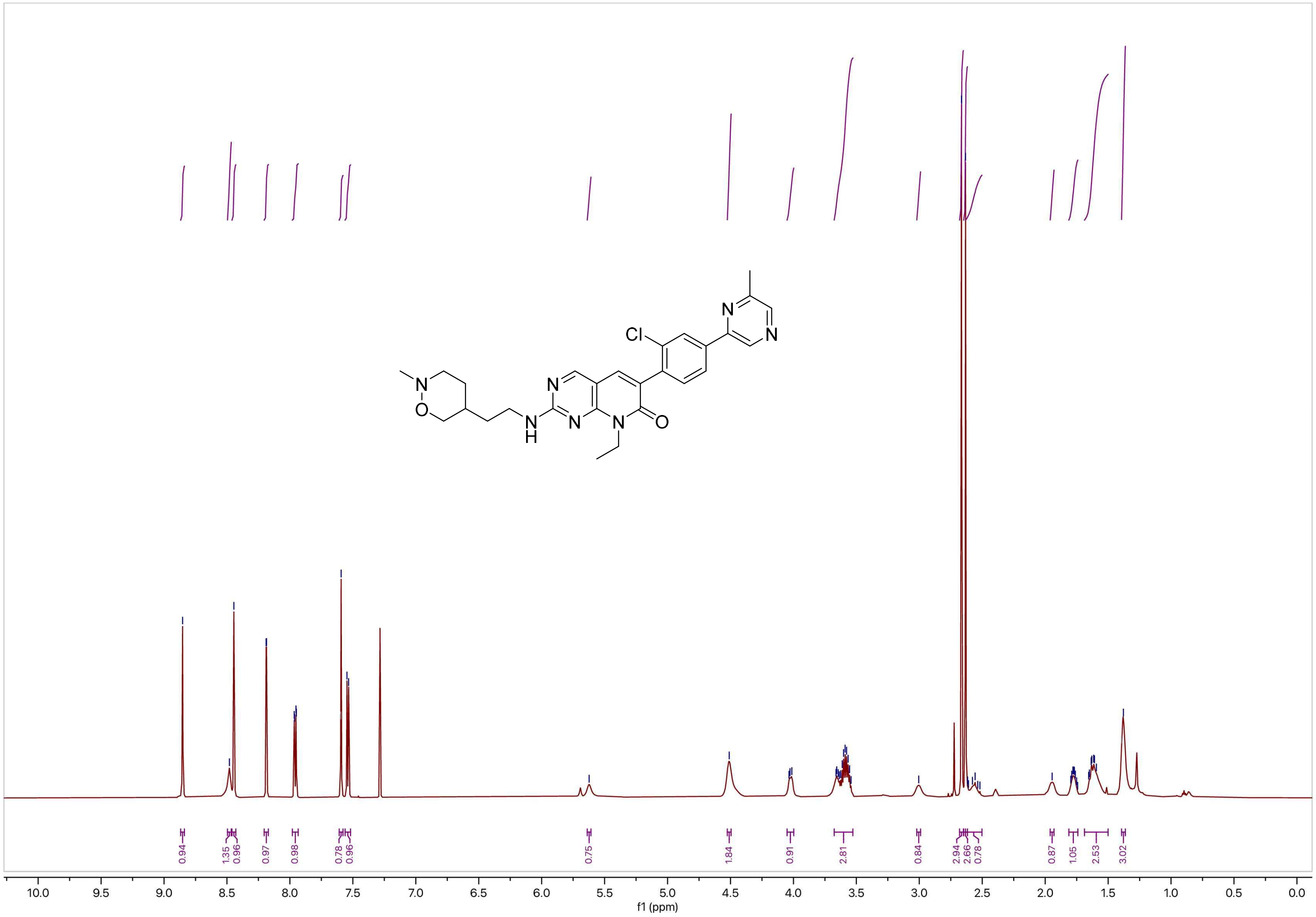

^{13}C NMR (151 MHz, $CDCl_3$) of 6-(2-chloro-4-(6-methylpyrazin-2-yl)phenyl)-8-ethyl-2-((2-(2-methyl-1,2-oxazinan-5-yl)ethyl)amino)pyrido[2,3-*d*]pyrimidin-7(8*H*)-one (**8**)

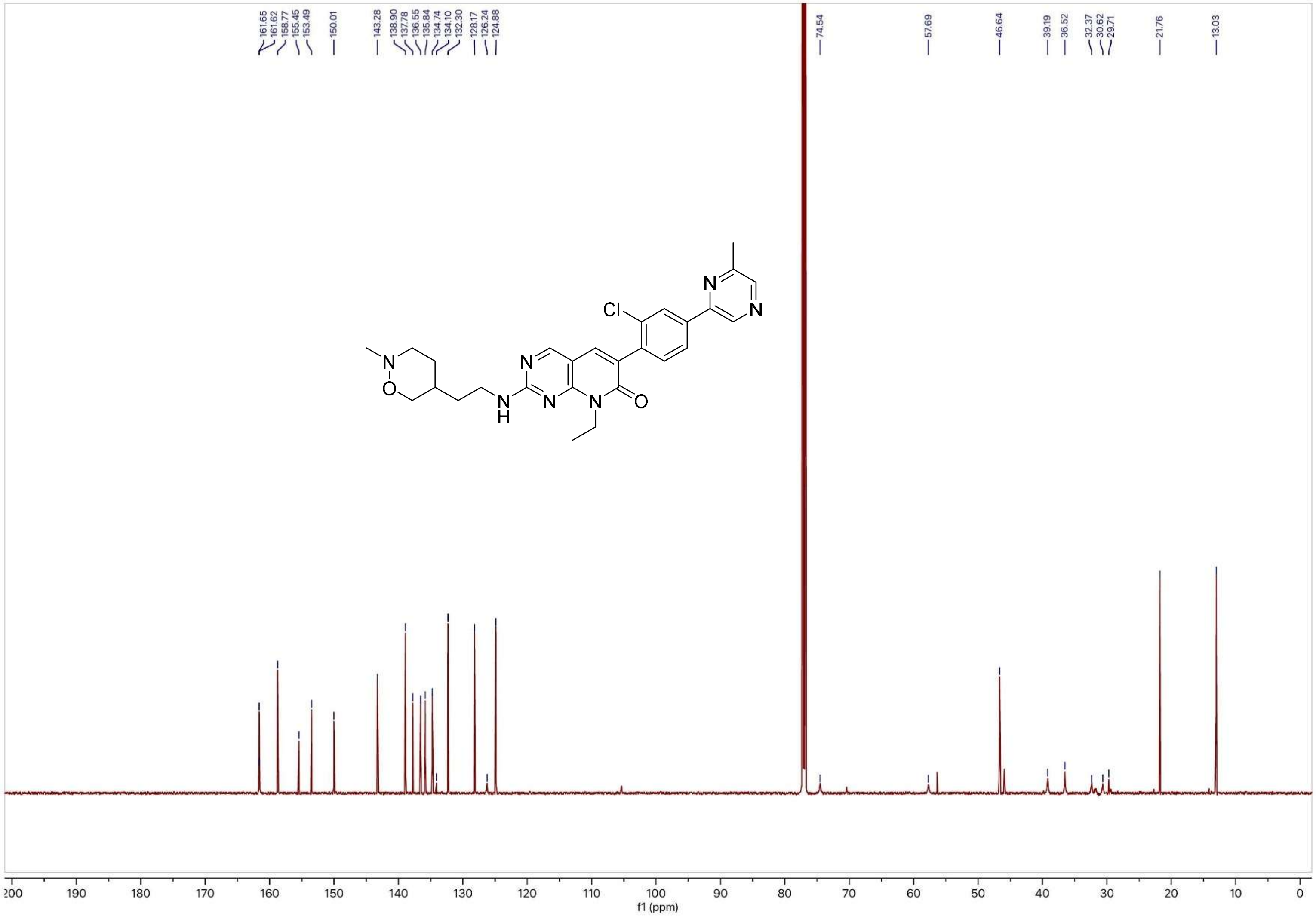

HSQC ($CDCl_3$) spectrum of 6-(2-chloro-4-(6-methylpyrazin-2-yl)phenyl)-8-ethyl-2-((2-(2-methyl-1,2-oxazinan-5-yl)ethyl)amino)pyrido[2,3-*d*]pyrimidin-7(8*H*)-one (**8**)

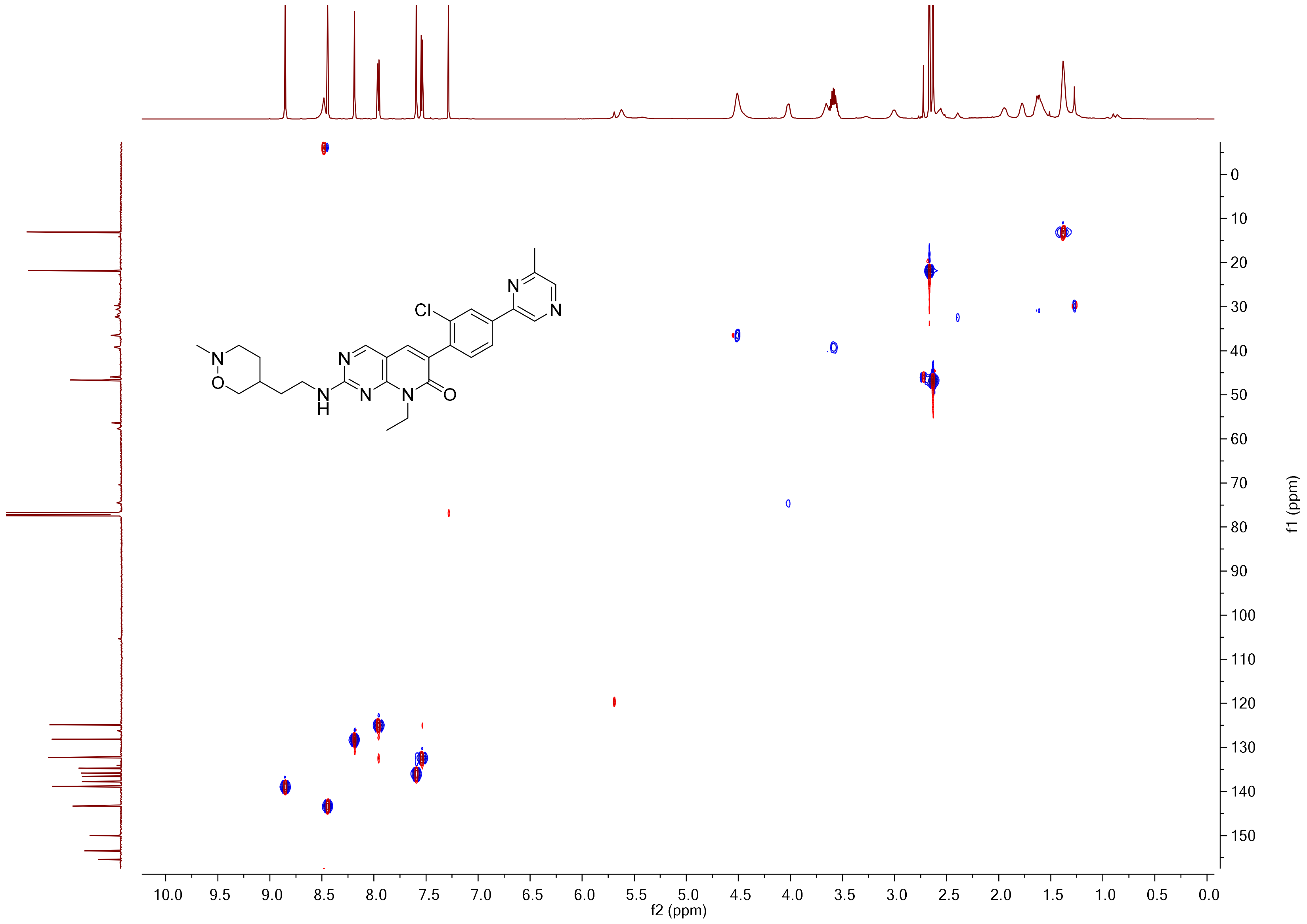

HMBC ($CDCl_3$) spectrum of 6-(2-chloro-4-(6-methylpyrazin-2-yl)phenyl)-8-ethyl-2-((2-(2-methyl-1,2-oxazinan-5-yl)ethyl)amino)pyrido[2,3-*d*]pyrimidin-7(8*H*)-one (**8**)

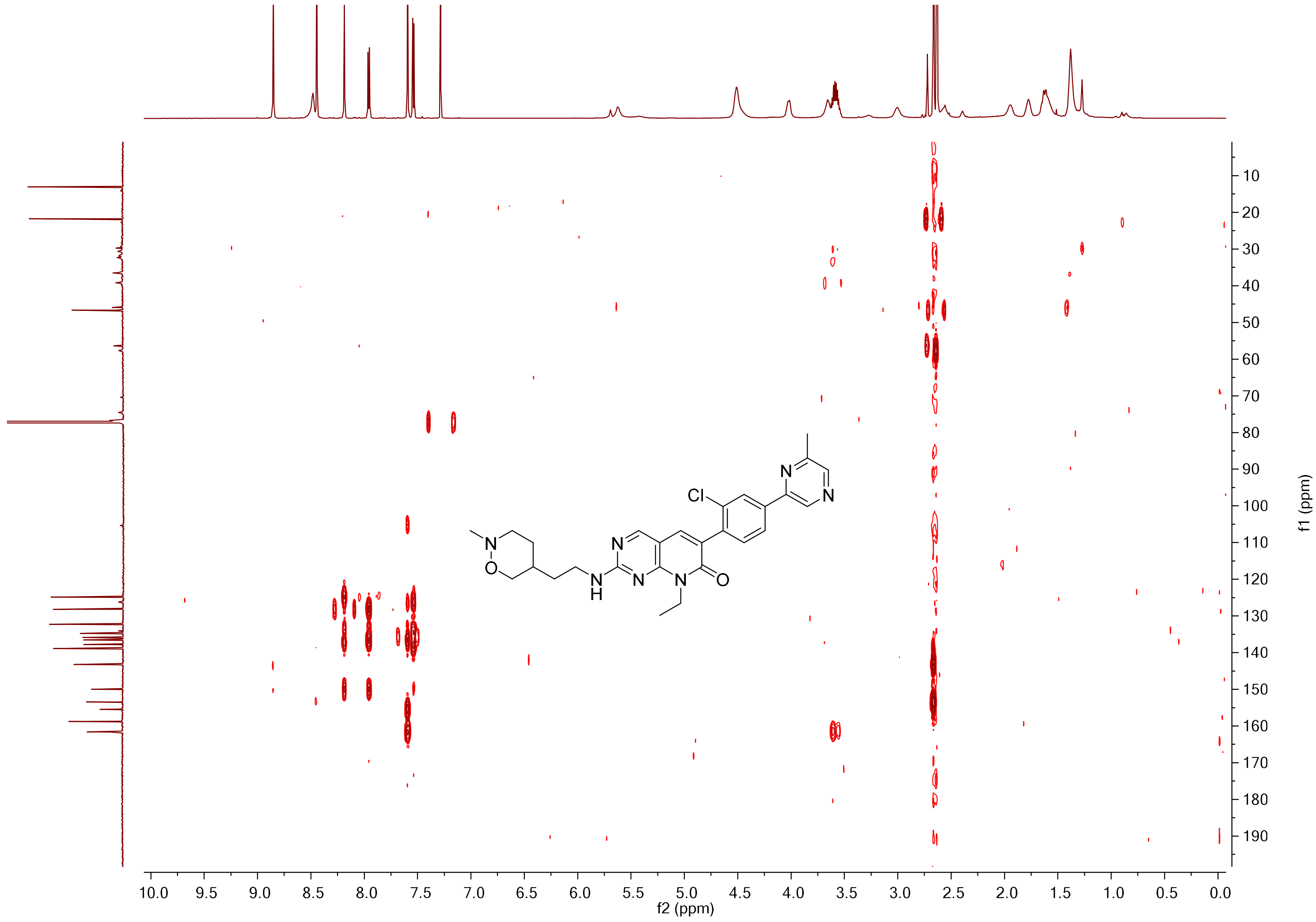

COSY ($CDCl_3$) spectrum of 6-(2-chloro-4-(6-methylpyrazin-2-yl)phenyl)-8-ethyl-2-((2-(2-methyl-1,2-oxazinan-5-yl)ethyl)amino)pyrido[2,3-*d*]pyrimidin-7(8*H*)-one (**8**)

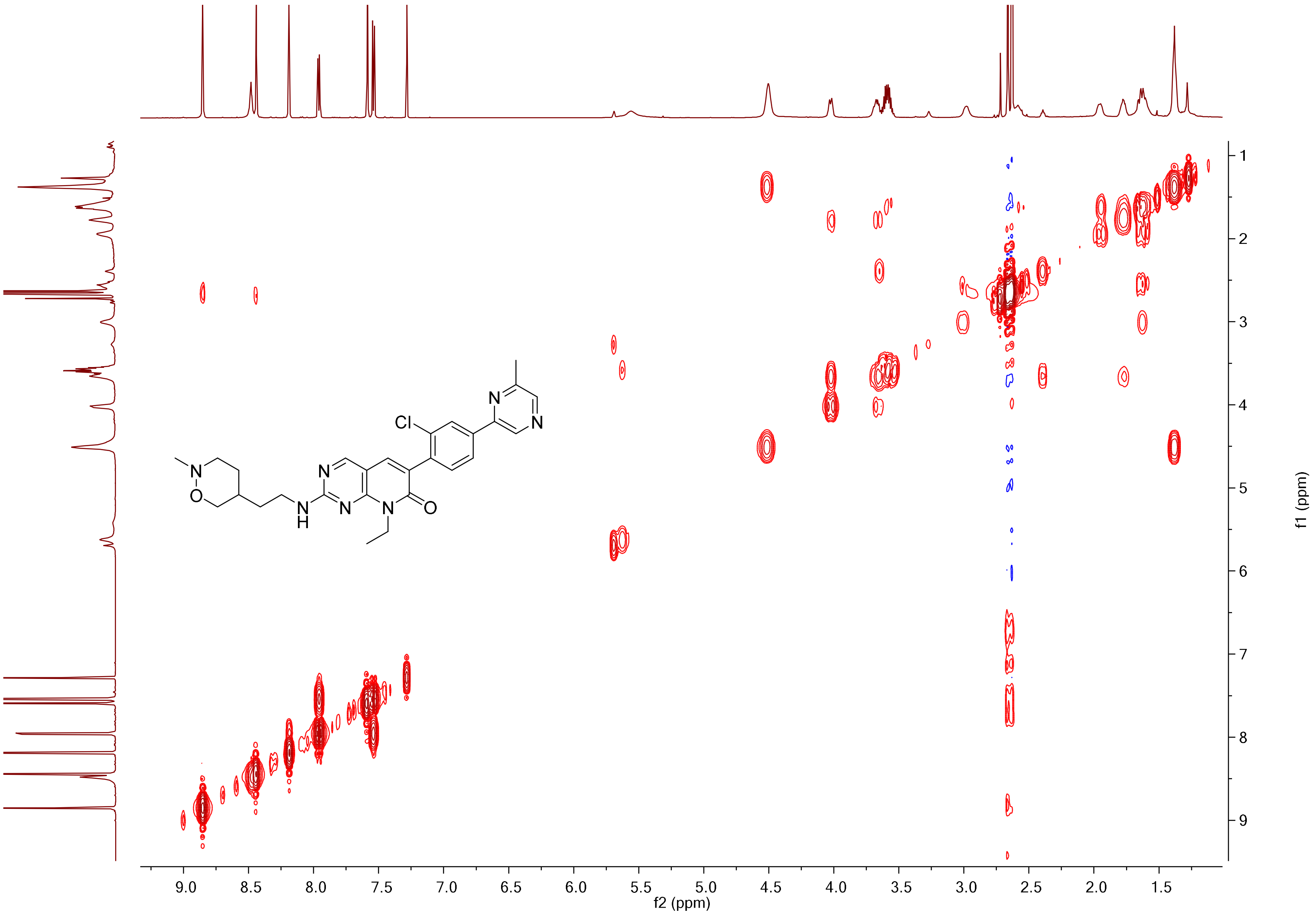

^{1}H NMR Spectra (600 MHz, Toluene-D_8) spectrum of 1-(2-azidoethoxy)-4-methylpiperazine **(25)**

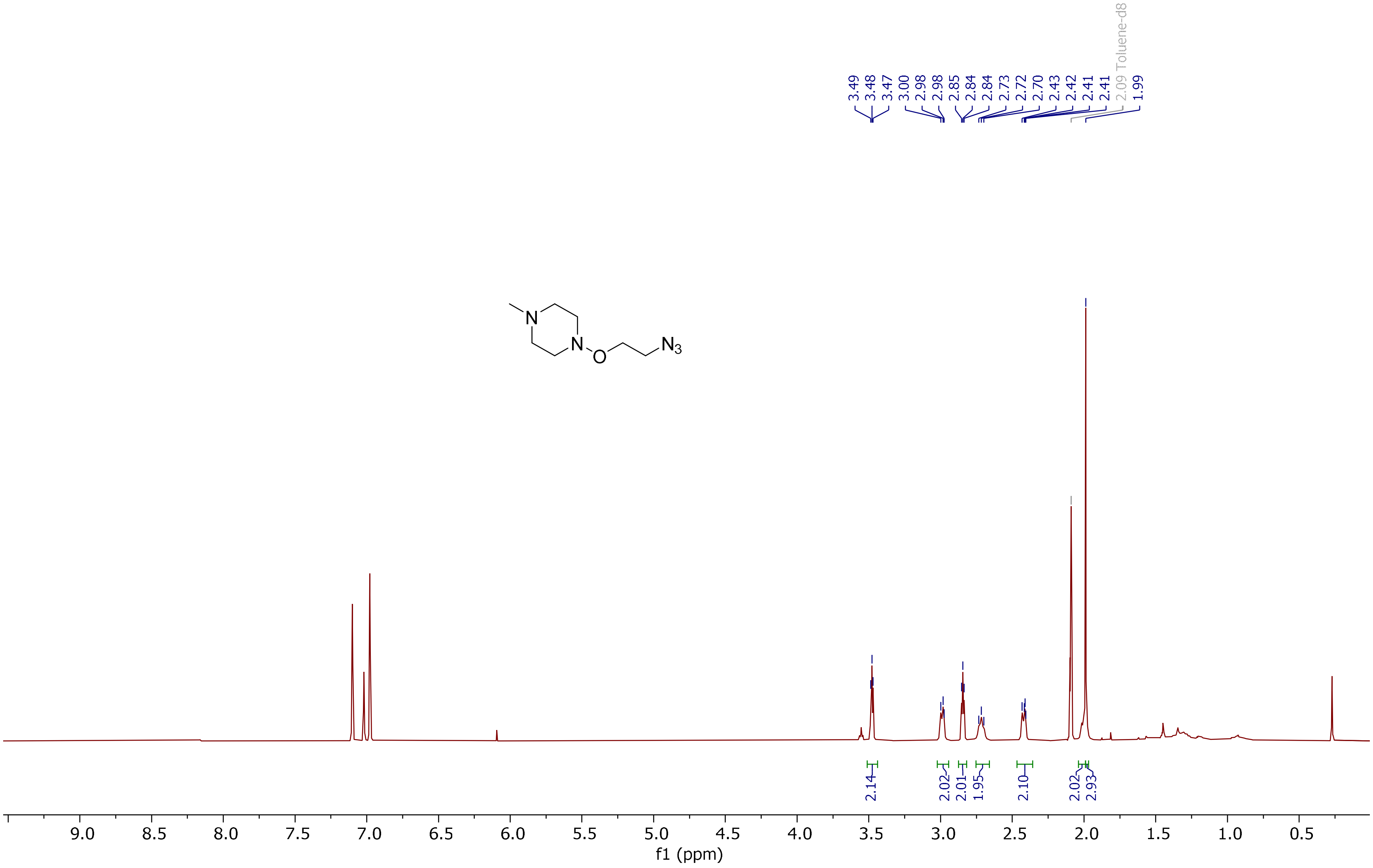

^{13}C NMR (151 MHz, Toluene-D$_8$) spectrum of 1-(2-azidoethoxy)-4-methylpiperazine **(25)**

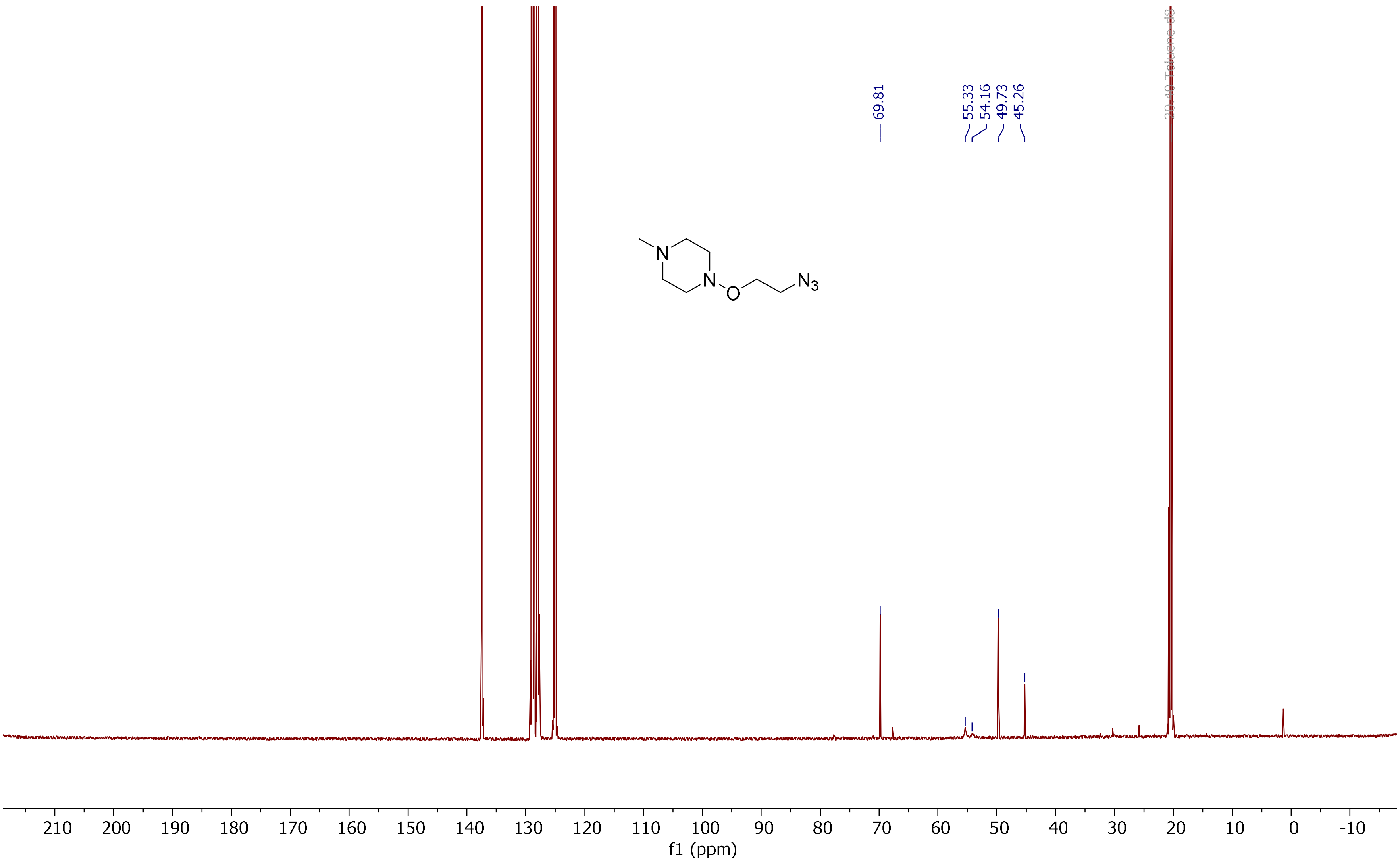

Expanded region of stacked variable temperature ^{13}C NMR (151 MHz, Toluene-D_8) spectrum of 1-(2-azidoethoxy)-4-methylpiperazine **(25)** at a) 348 K and b) 298 K

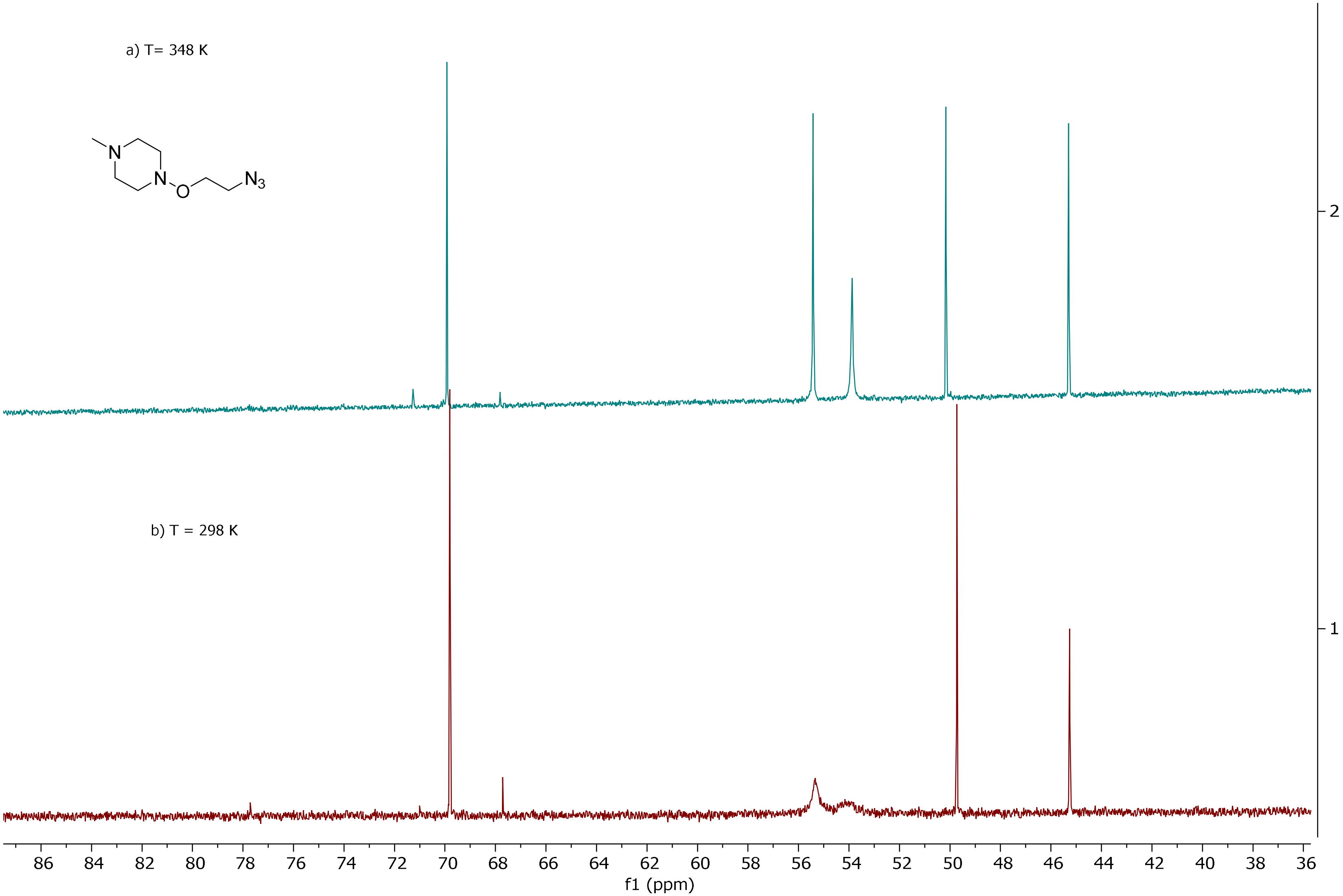

HSQC (Toluene-D_8) spectrum of 1-(2-azidoethoxy)-4-methylpiperazine **(25)**

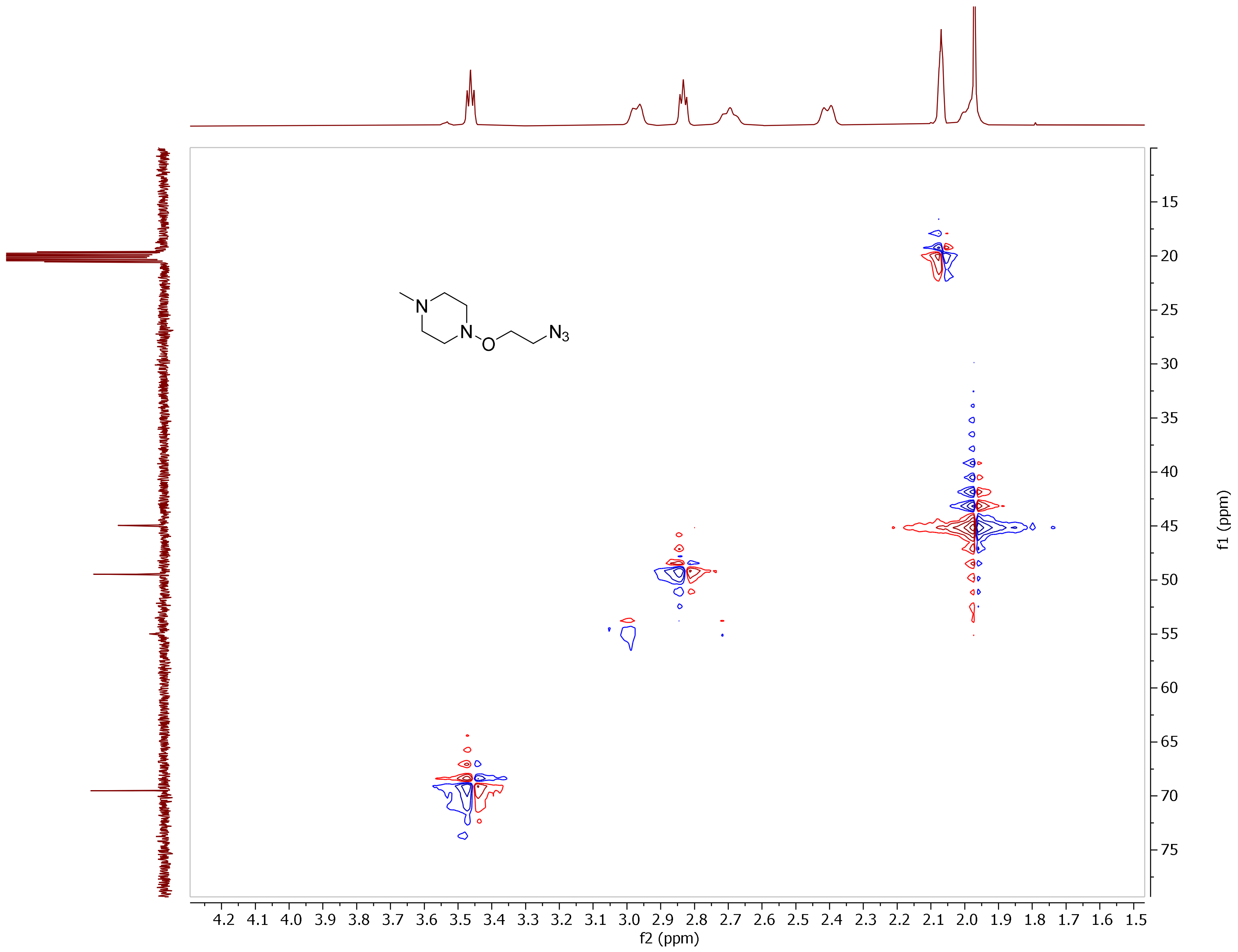

COSY (Toluene-D$_8$) spectrum of 1-(2-azidoethoxy)-4-methylpiperazine **(25)**

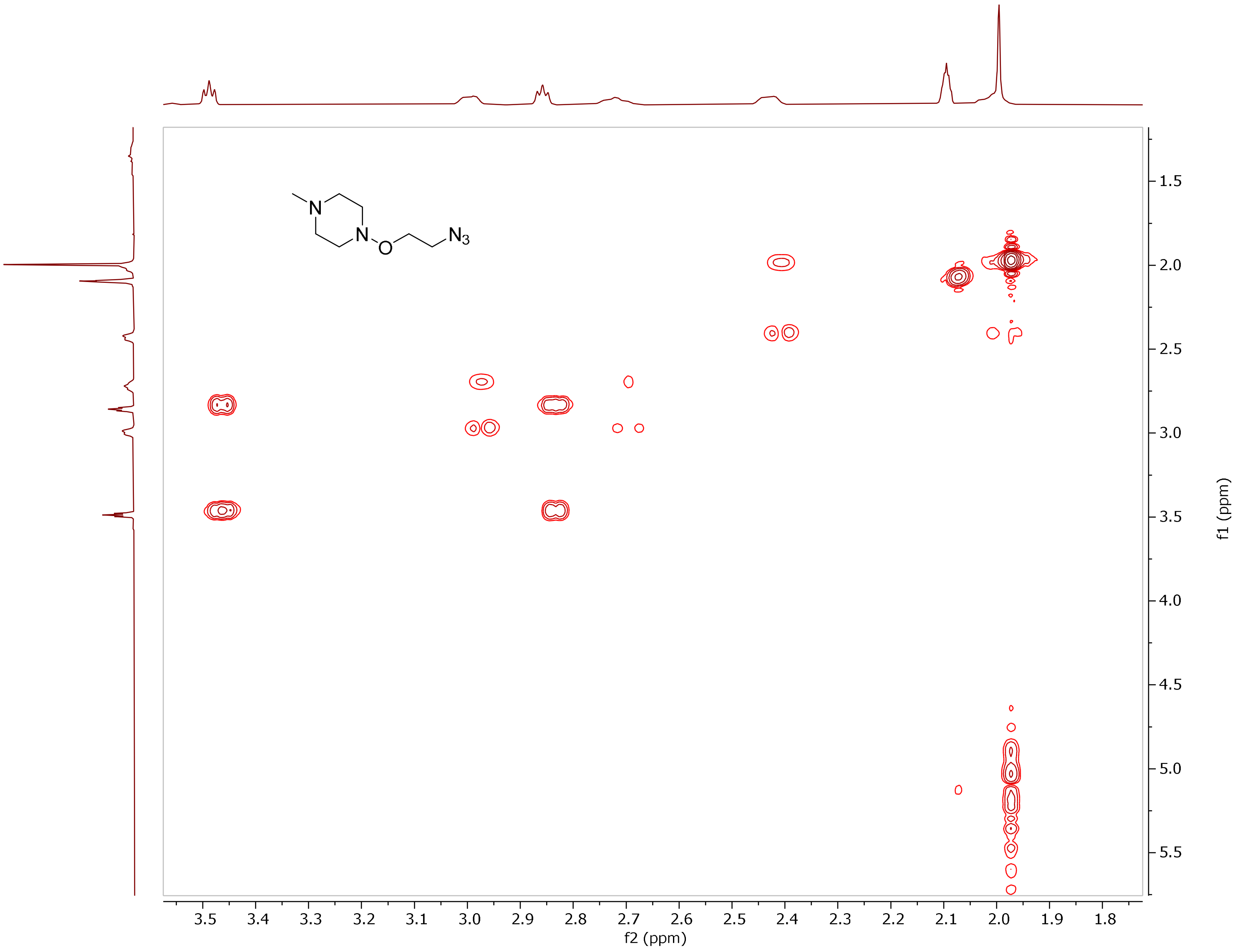

^{1}H NMR (900 MHz, Toluene-D_8) spectrum of 2-((4-methylpiperazin-1-yl)oxy)ethan-1-amine **(26)**

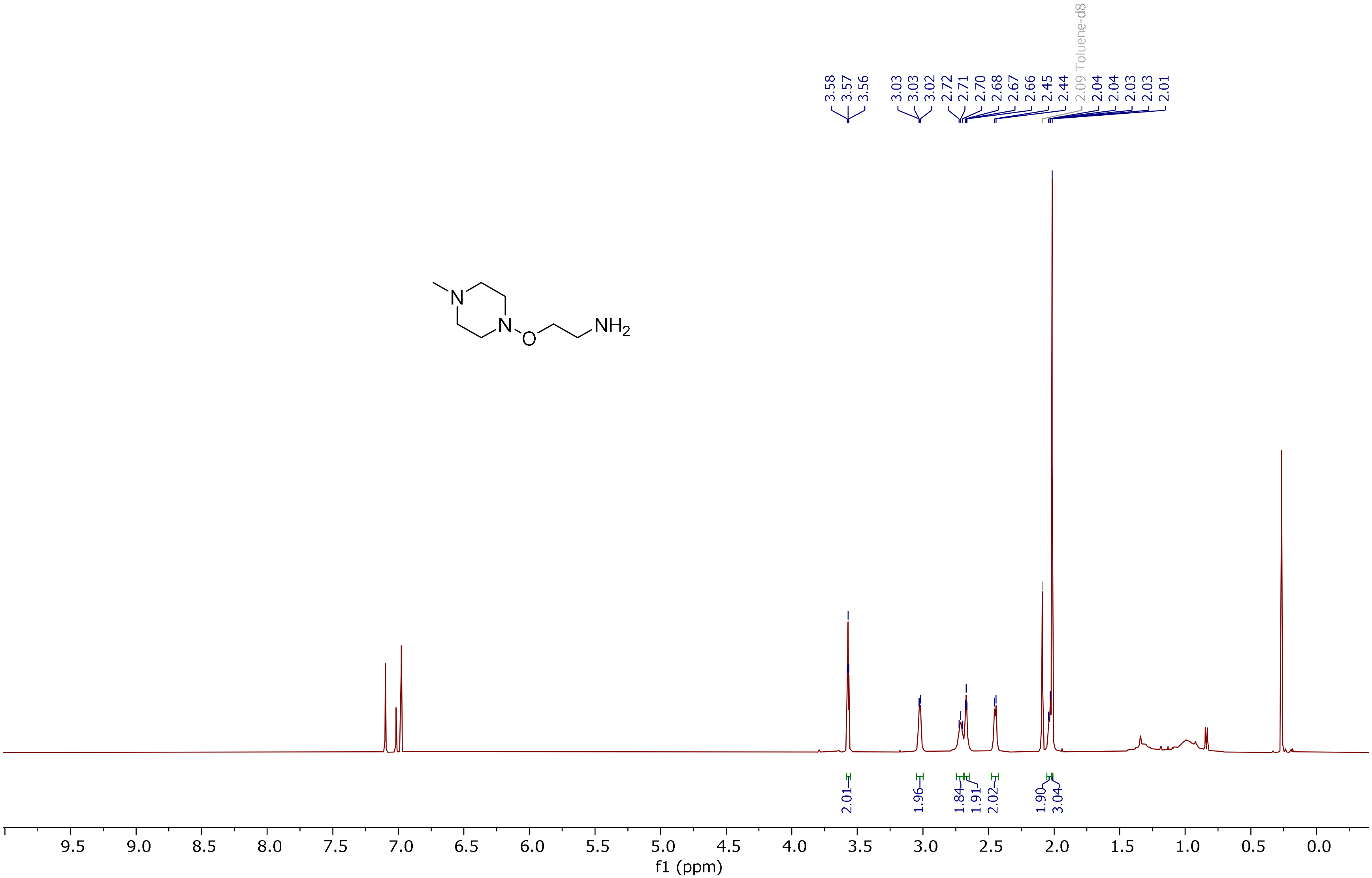

^{13}C NMR (226 MHz, Toluene-D$_8$) spectrum of 2-((4-methylpiperazin-1-yl)oxy)ethan-1-amine **(26)**

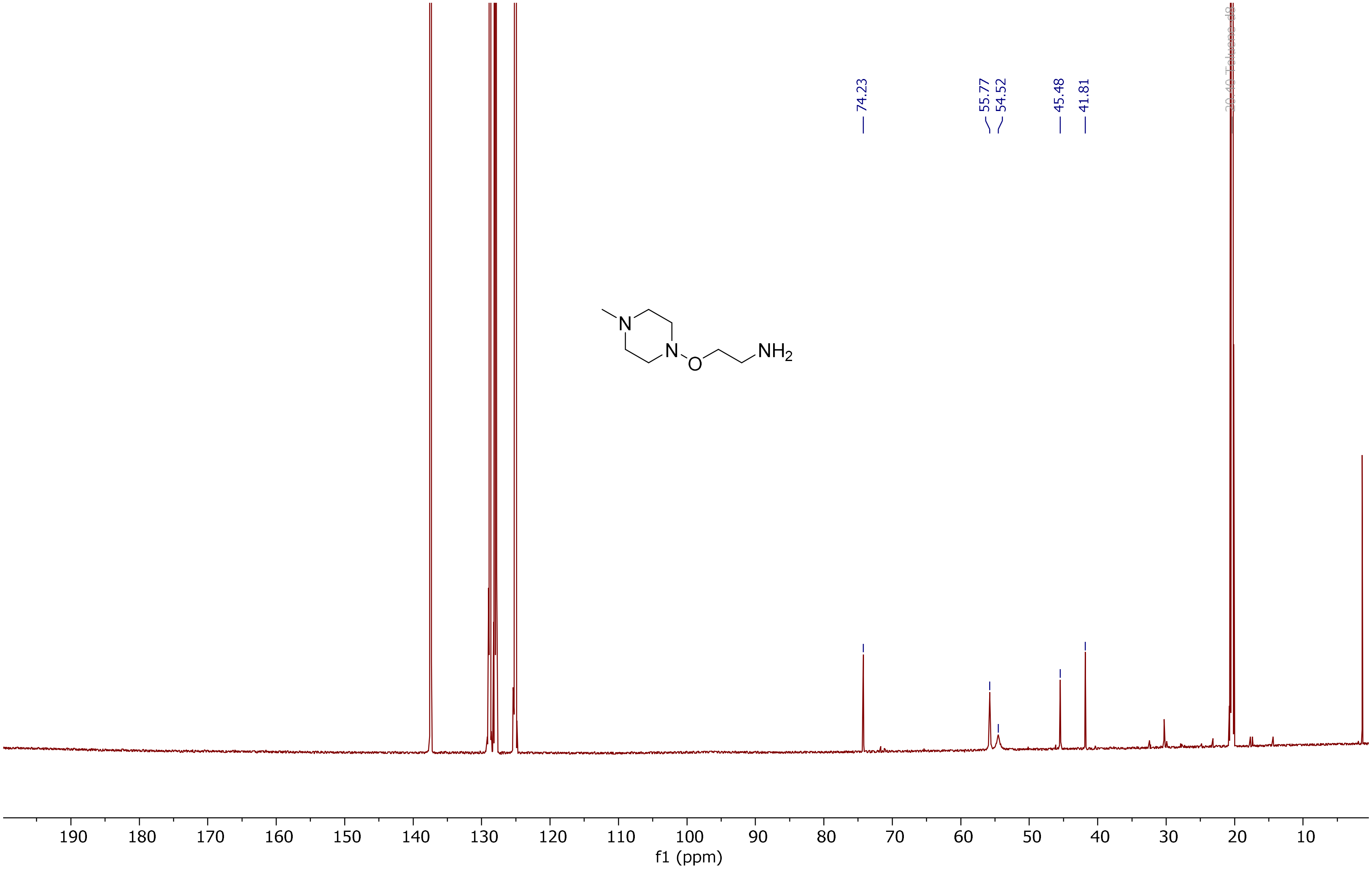

Expanded region of stacked variable temperature ^{13}C NMR (226 MHz, Toluene-D_8) spectrum of 2-((4-methylpiperazin-1-yl)oxy)ethan-1-amine **(26)** at a) 343 K and b) 298 K

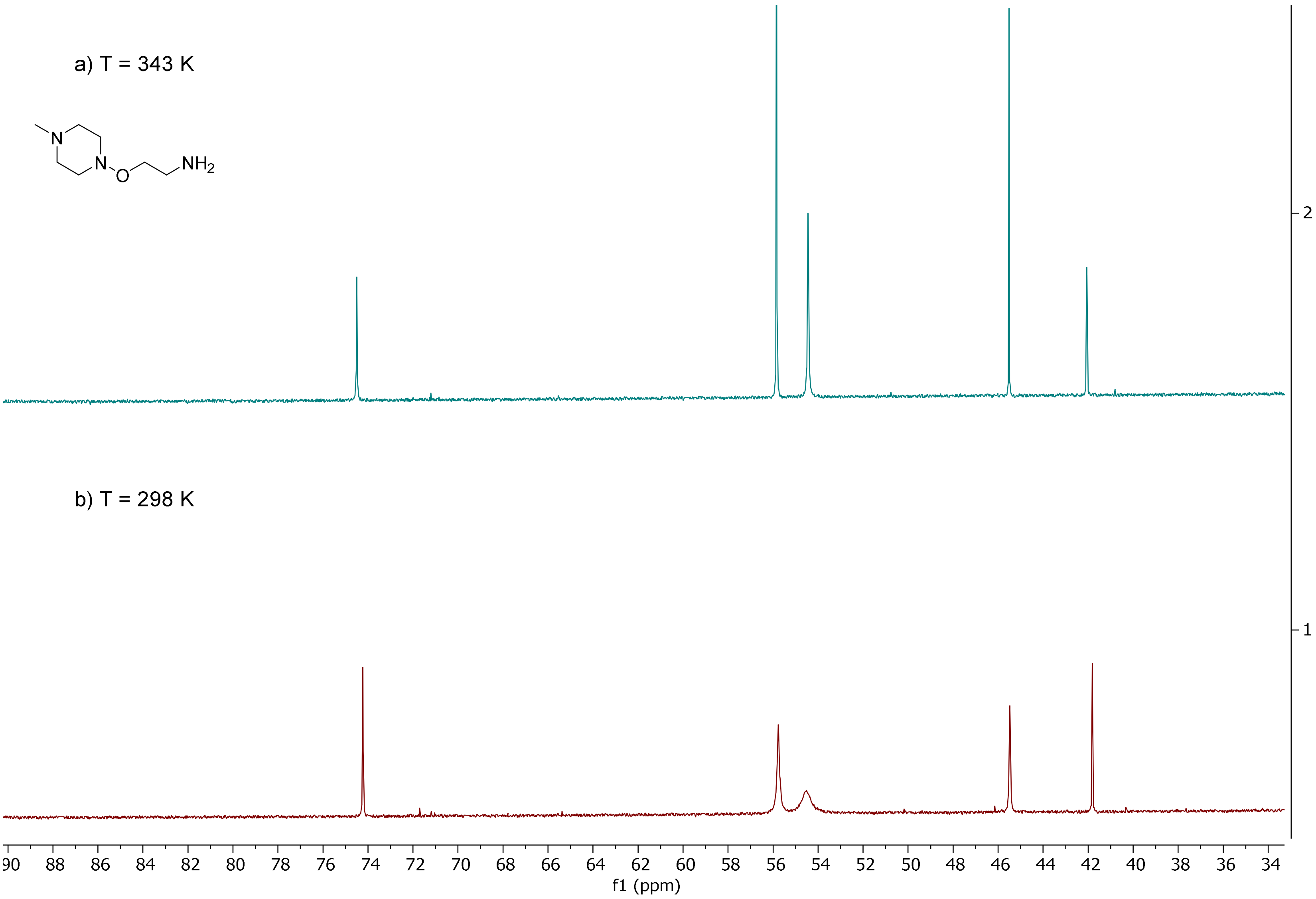

HSQC ($CDCl_3$) spectrum of 2-((4-methylpiperazin-1-yl)oxy)ethan-1-amine **(26)**

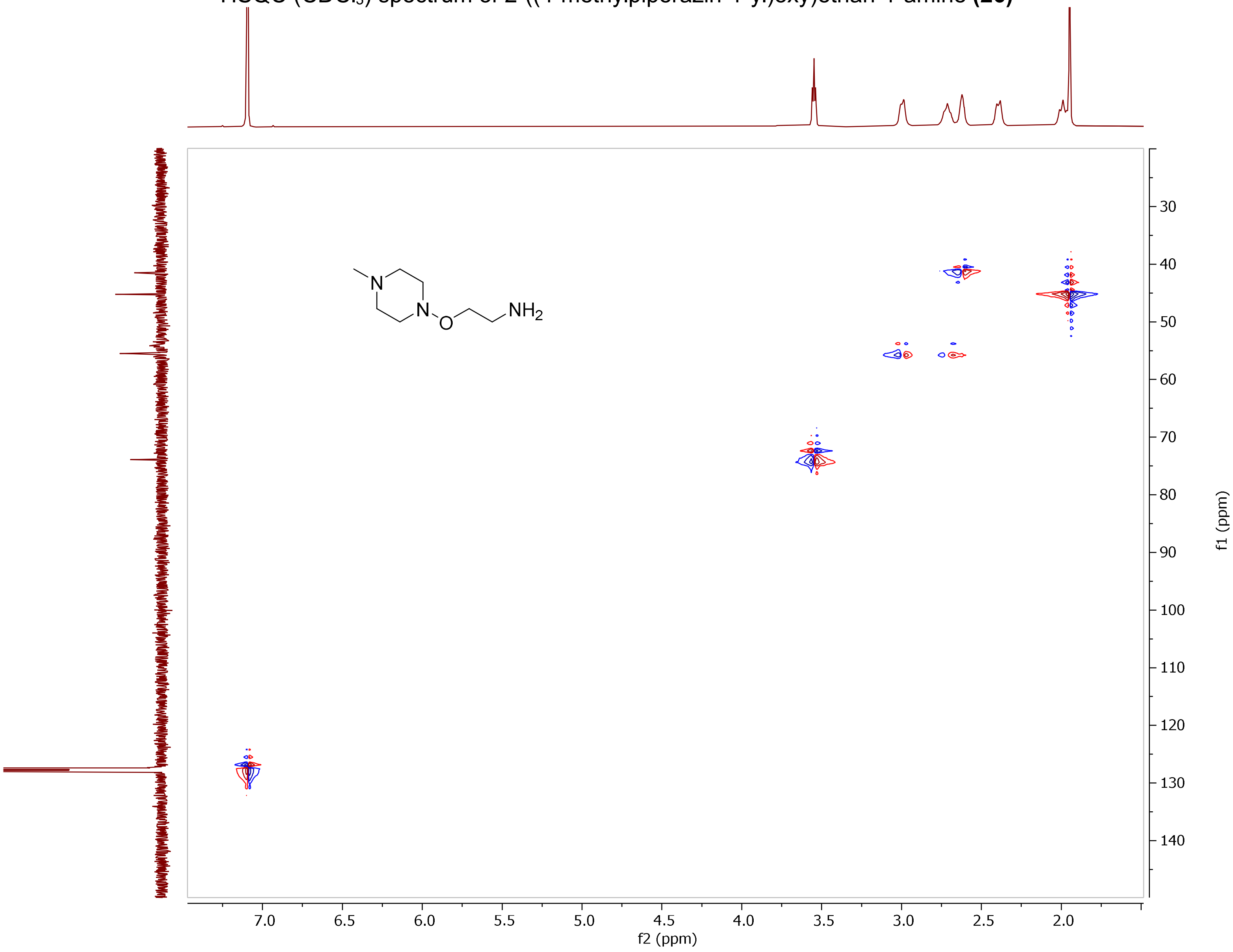

COSY ($CDCl_3$) spectrum of 2-((4-methylpiperazin-1-yl)oxy)ethan-1-amine **(26)**

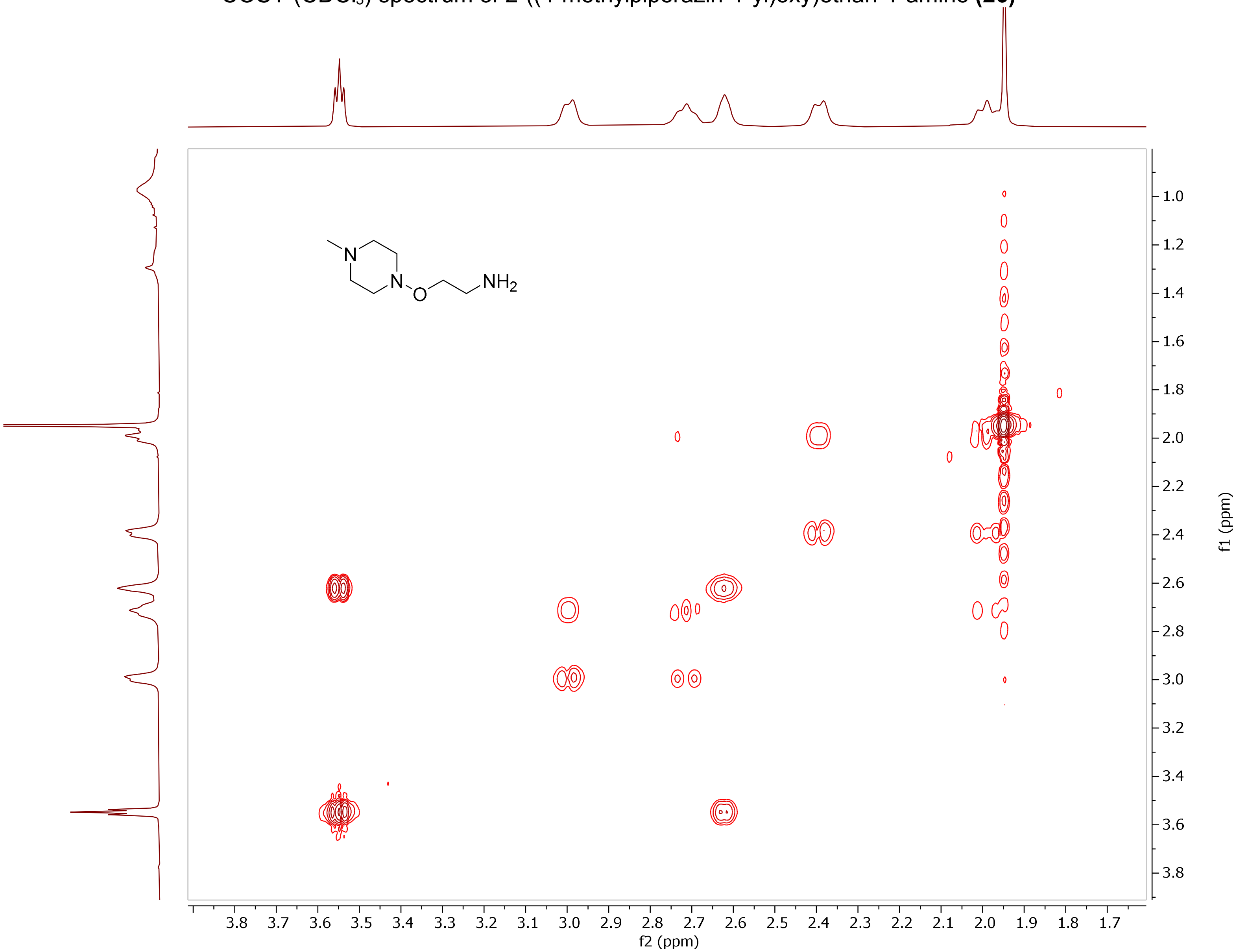

^{1}H NMR (500 MHz, $CDCl_3$) spectrum of 6-(2-chloro-4-(6-methylpyrazin-2-yl)phenyl)-8-ethyl-2-((2-((4-methylpiperazin-1-yl)oxy)ethyl)amino)pyrido[2,3-*d*]pyrimidin-7(8*H*)-one) **(9)**

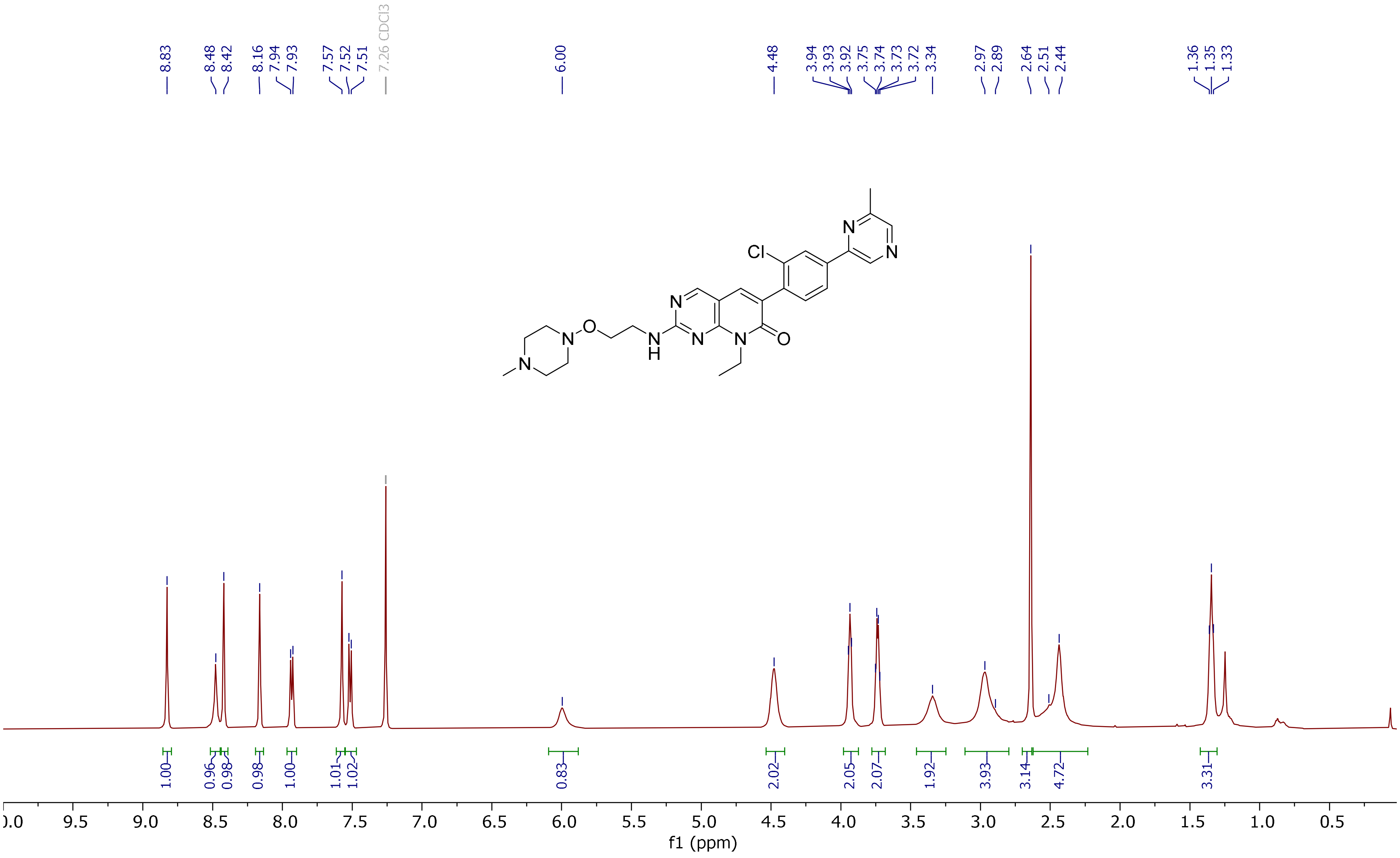

^{13}C NMR (151 MHz, $CDCl_3$) spectrum of 6-(2-chloro-4-(6-methylpyrazin-2-yl)phenyl)-8-ethyl-2-((2-((4-methylpiperazin-1-yl)oxy)ethyl)amino)pyrido[2,3-*d*]pyrimidin-7(8*H*)-one) **(9)** at 343 K

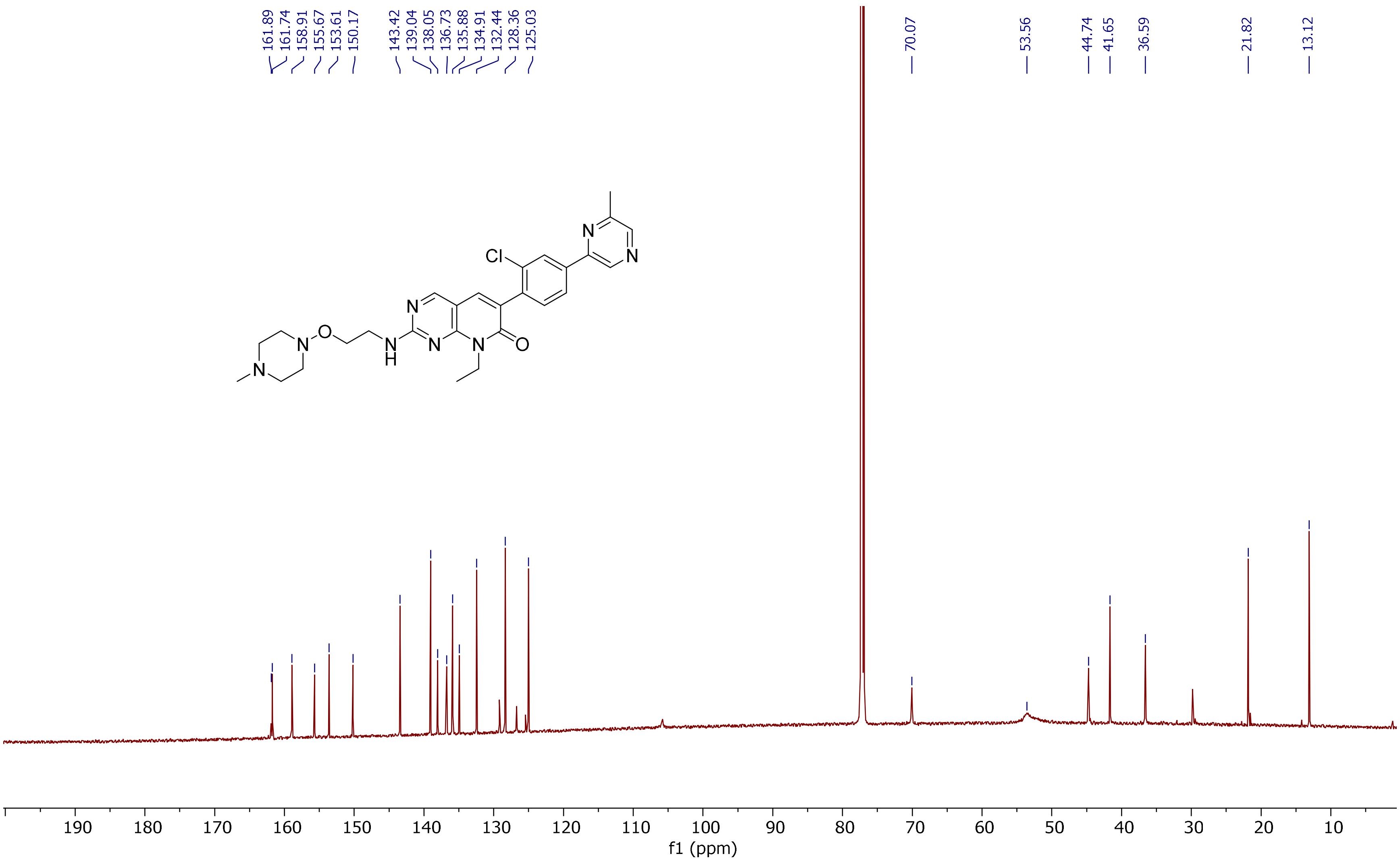

HSQC ($CDCl_3$) spectrum of 6-(2-chloro-4-(6-methylpyrazin-2-yl)phenyl)-8-ethyl-2-((2-((4-methylpiperazin-1-yl)oxy)ethyl)amino)pyrido[2,3-*d*]pyrimidin-7(8*H*)-one) **(9)**

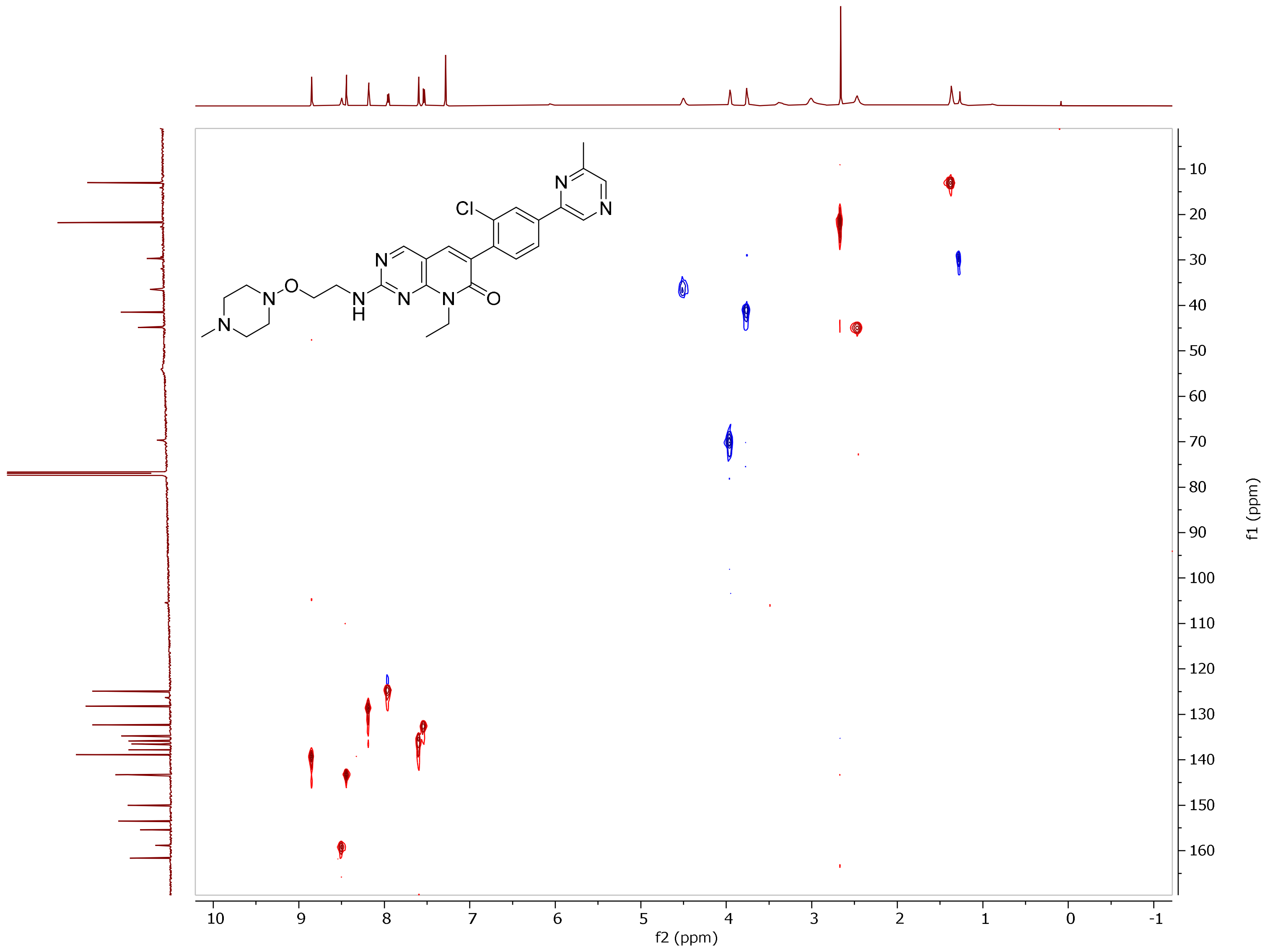

COSY ($CDCl_3$) spectrum of 6-(2-chloro-4-(6-methylpyrazin-2-yl)phenyl)-8-ethyl-2-((2-((4-methylpiperazin-1-yl)oxy)ethyl)amino)pyrido[2,3-*d*]pyrimidin-7(8*H*)-one) **(9)**

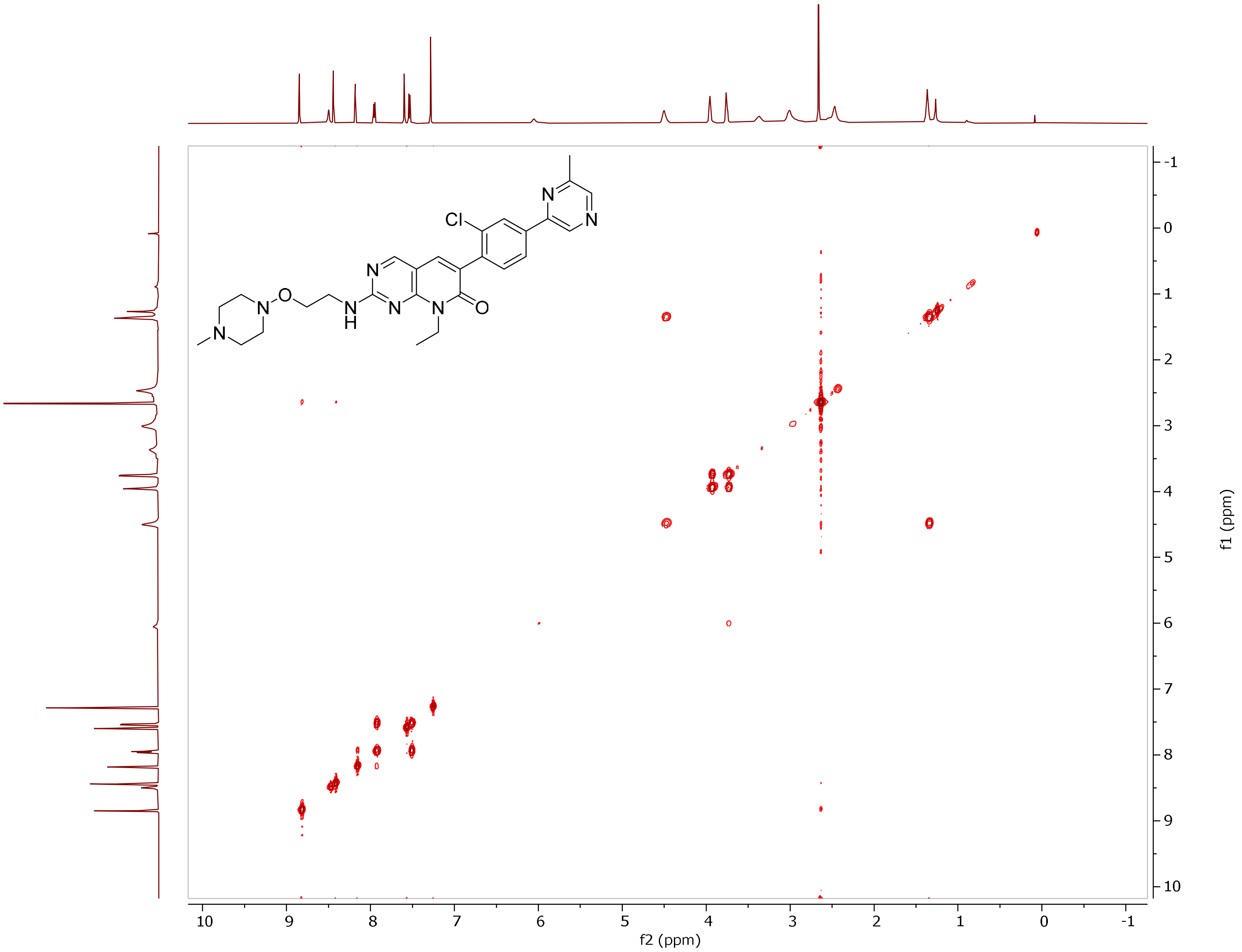

^{1}H NMR (500 MHz, $CDCl_3$) spectrum of *tert*-butyl 4-methoxypiperazine-1-carboxylate **(28)**

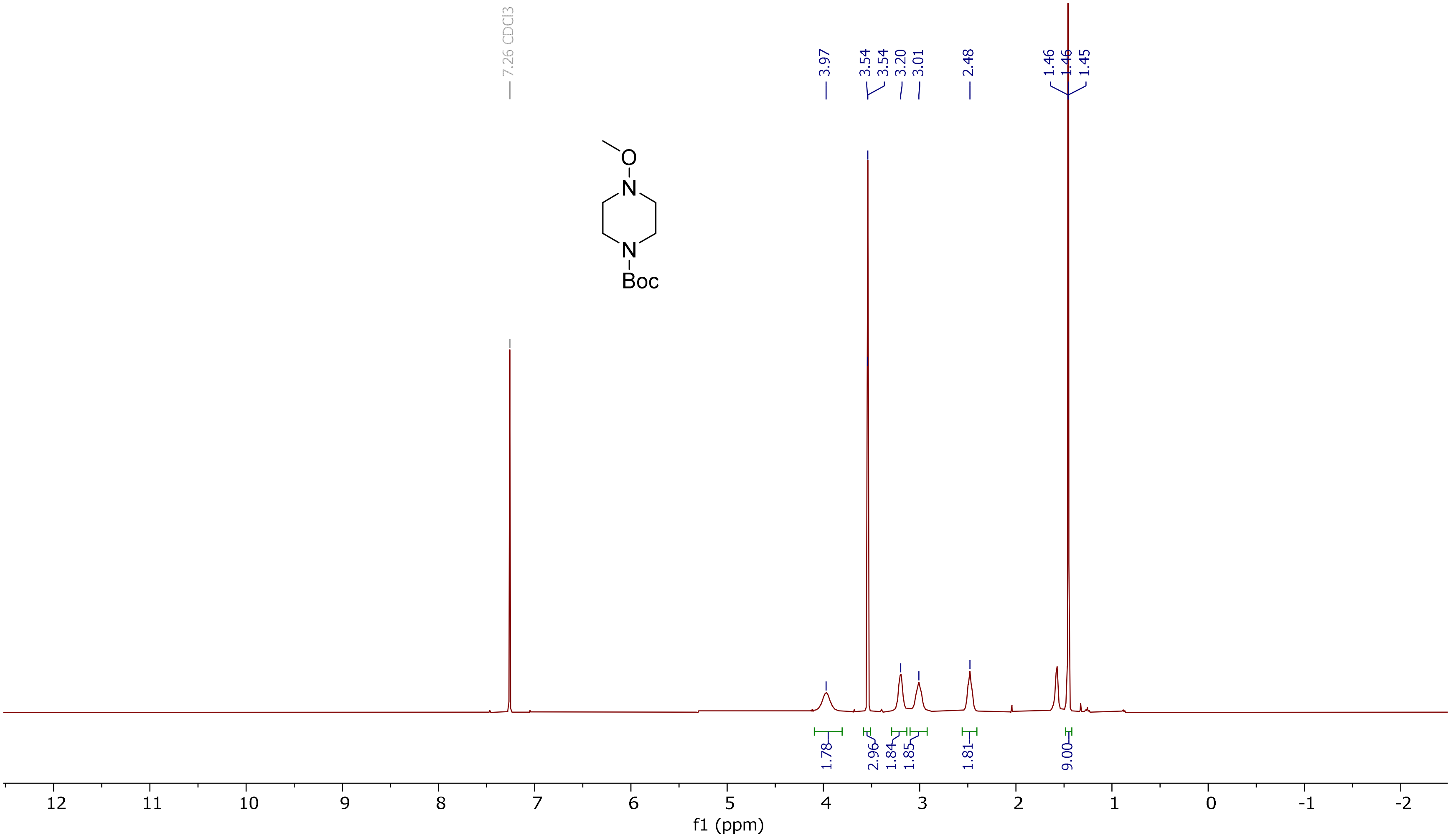

^{13}C NMR (126 MHz, Benzene-D_6) spectrum of *tert*-butyl 4-methoxypiperazine-1-carboxylate **(28)**

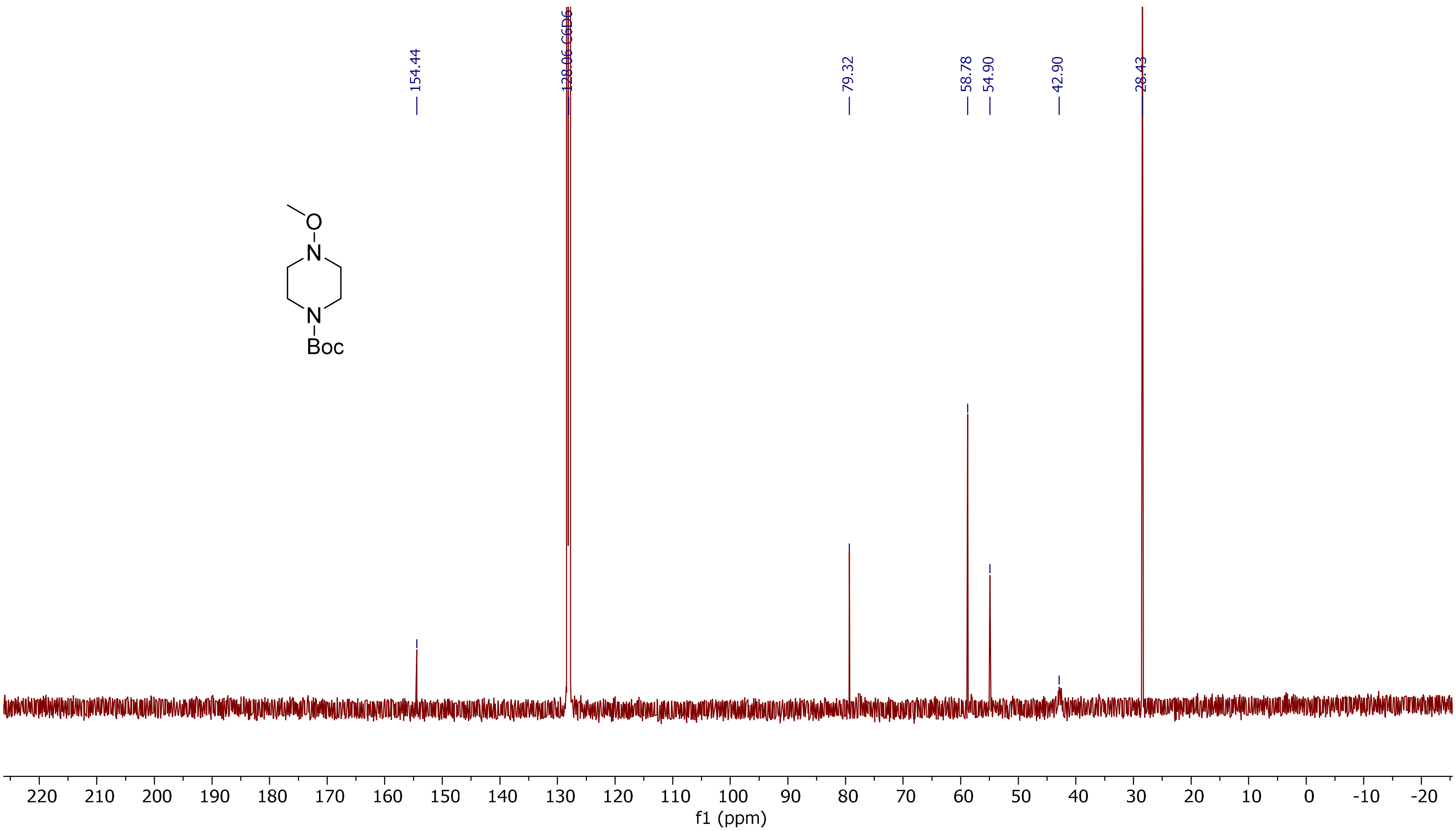

Expanded region of stacked variable temperature ^{13}C NMR (126 MHz, Benzene-D_6) spectrum of 2-((4-methylpiperazin-1-yl)oxy)ethan-1-amine **(28)** at a) 343 K and b) 298 K

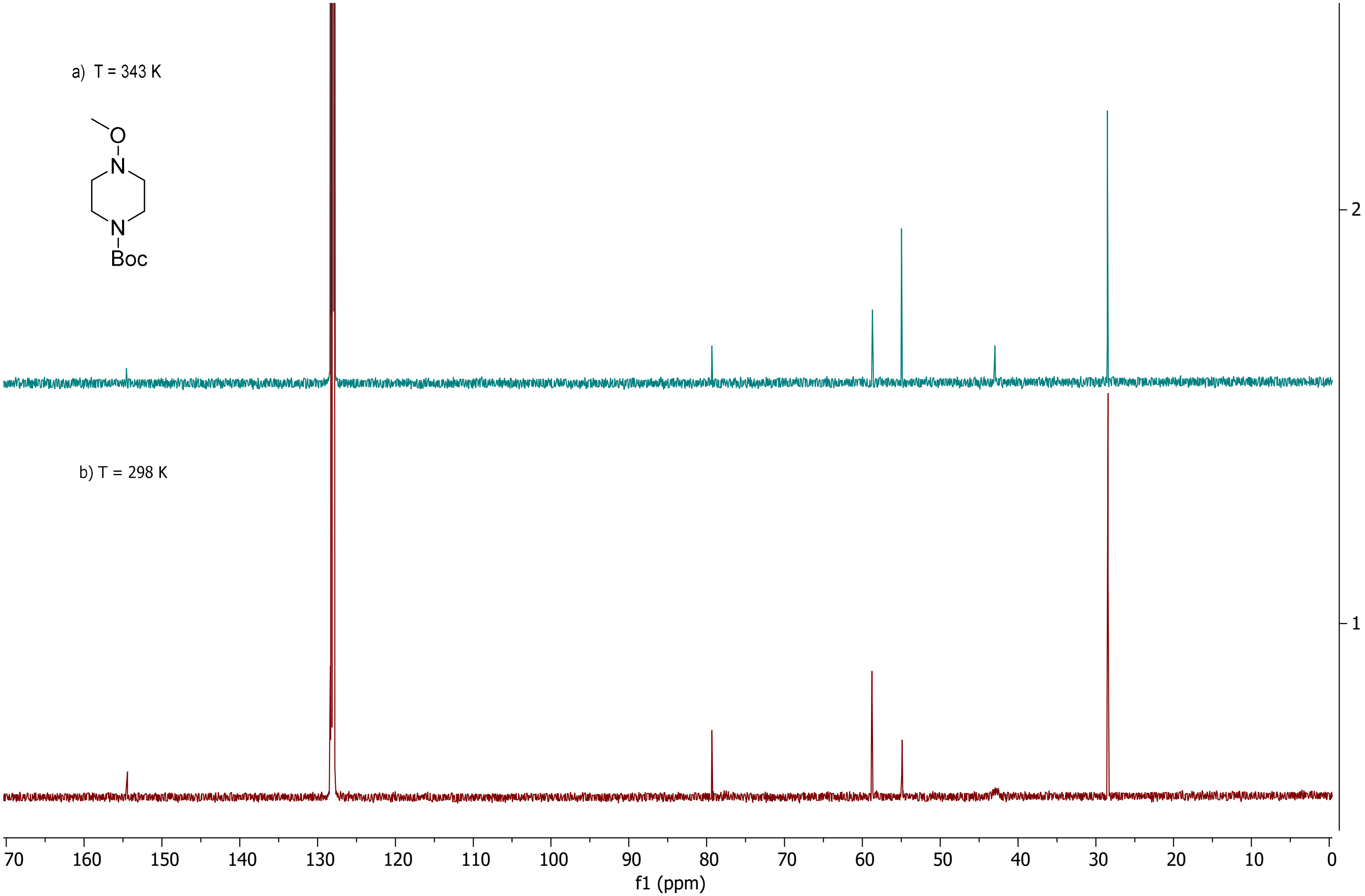

HSQC ($CDCl_3$) spectrum of *tert*-butyl 4-methoxypiperazine-1-carboxylate **(28)**

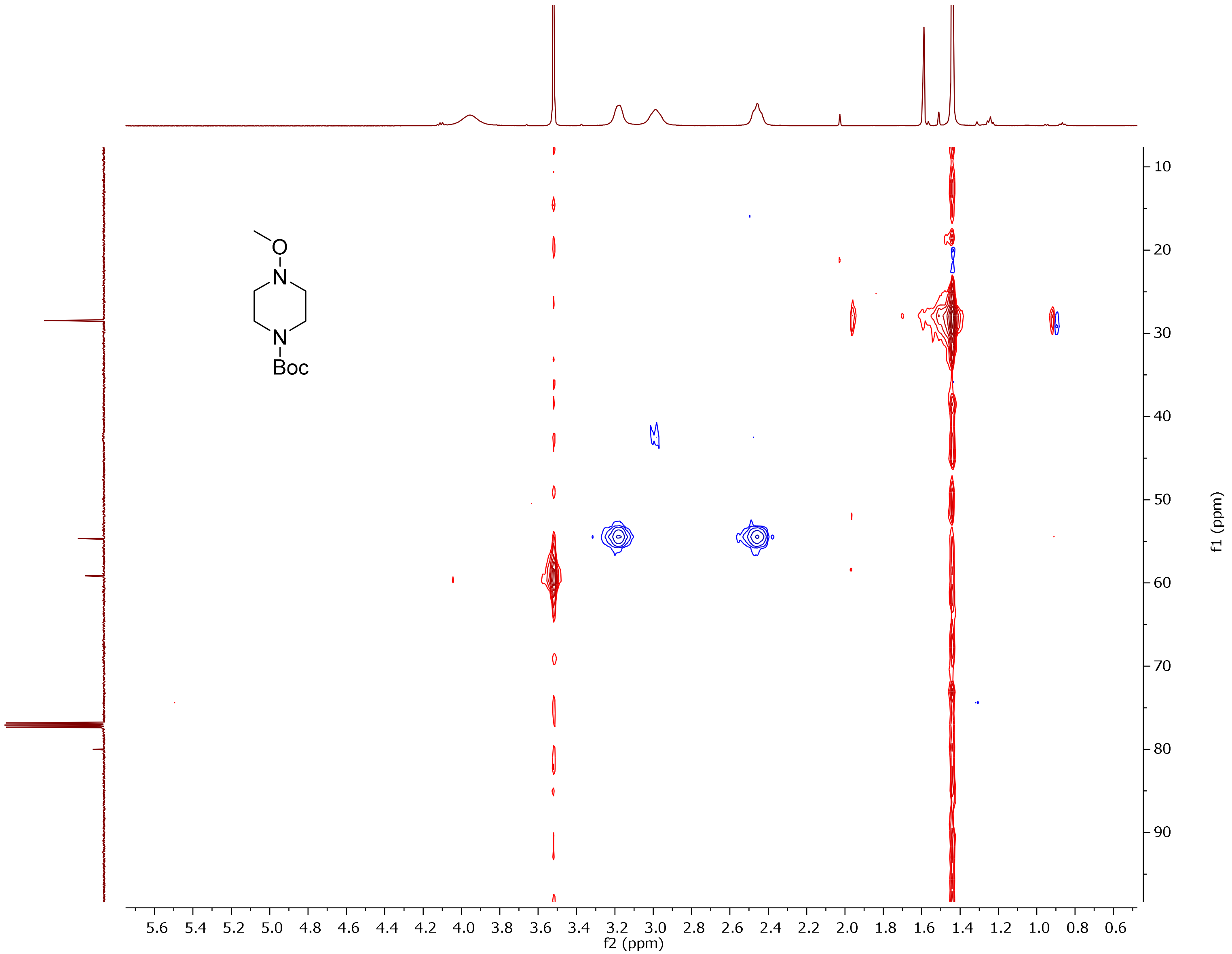

^{1}H NMR (500 MHz, $CDCl_3$) spectrum of 3-(4-methoxypiperazin-1-yl)propan-1-ol **(30)**

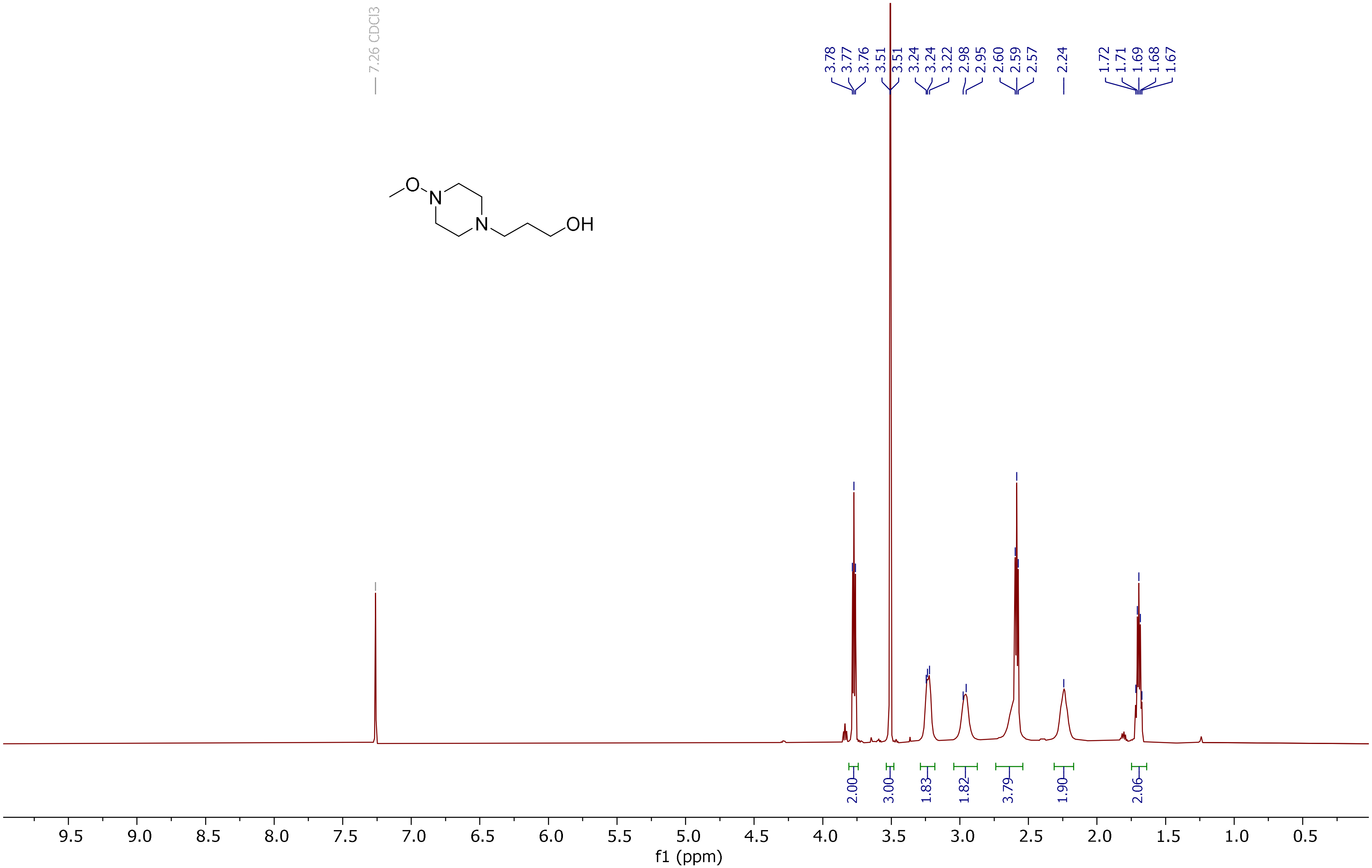

^{13}C NMR (126 MHz, $CDCl_3$) spectrum of 3-(4-methoxypiperazin-1-yl)propan-1-ol **(30)**

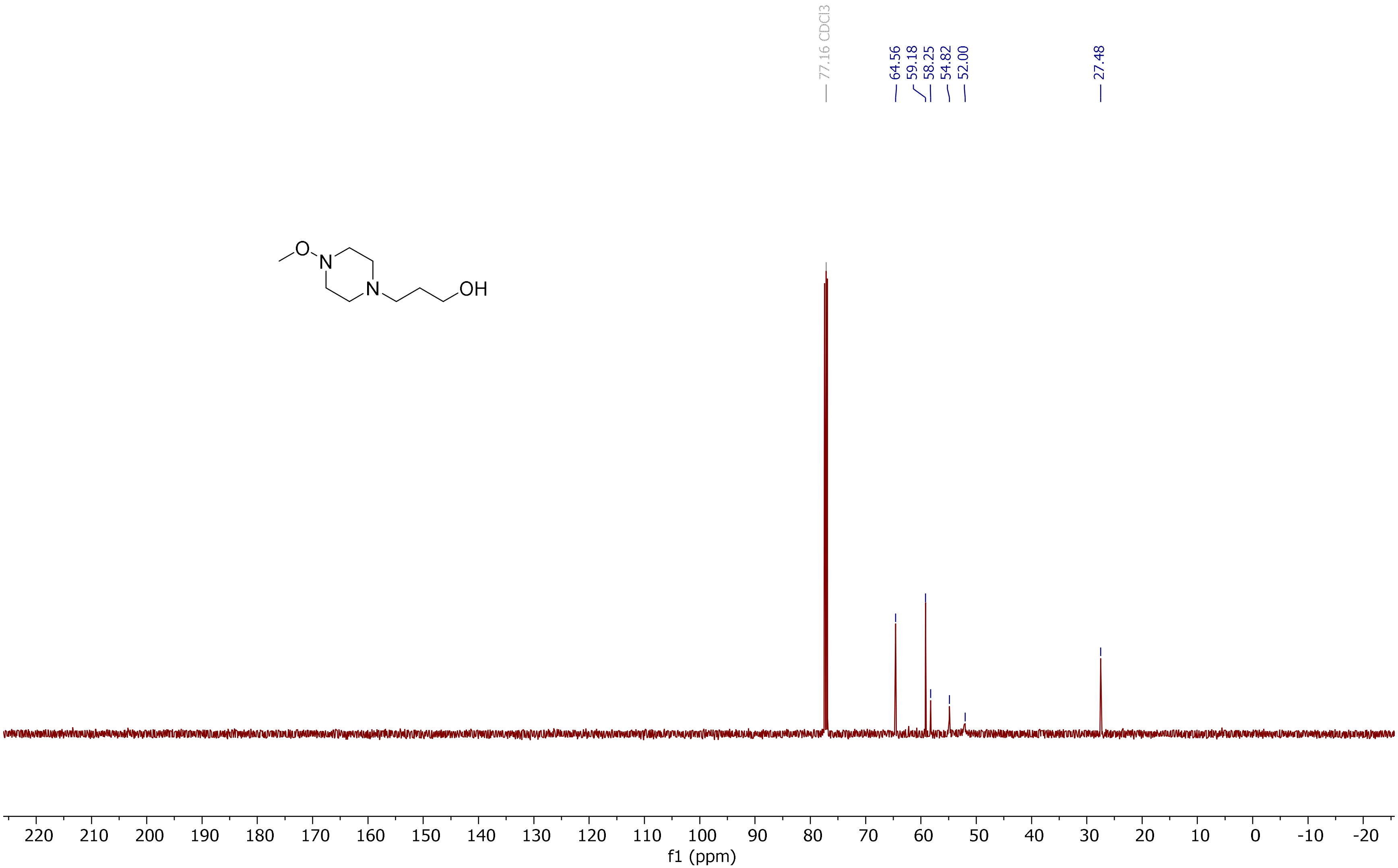

Expanded region of stacked variable temperature ^{13}C NMR (126 MHz, $CDCl_3$) spectrum of 2-((4-methylpiperazin-1-yl)oxy)ethan-1-amine **(30)** at a) 323 K and b) 298 K

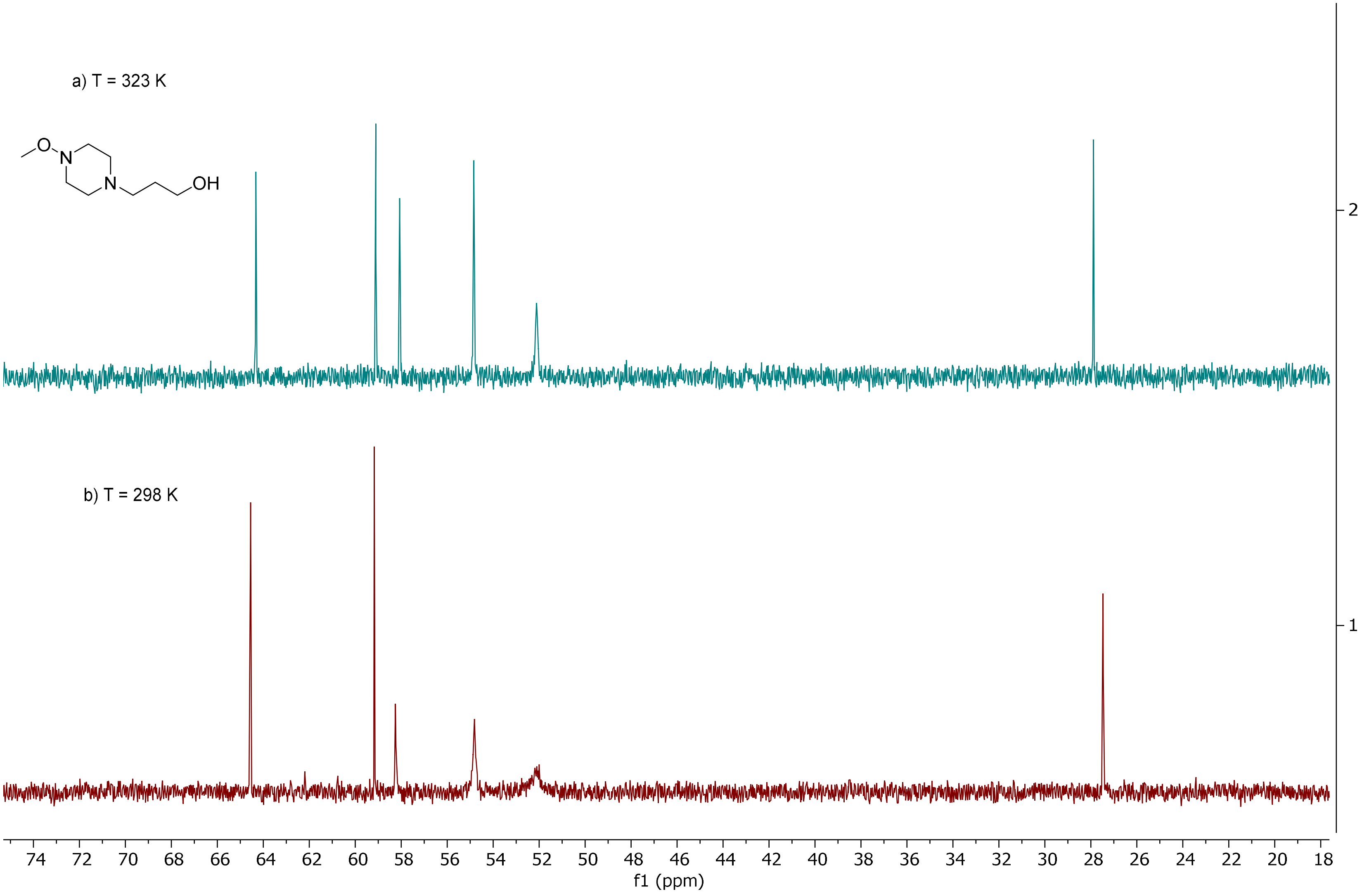

^{1}H NMR (500 MHz, Toluene-D$_8$) spectrum of 1-(3-azidopropyl)-4-methoxypiperazine **(31)**

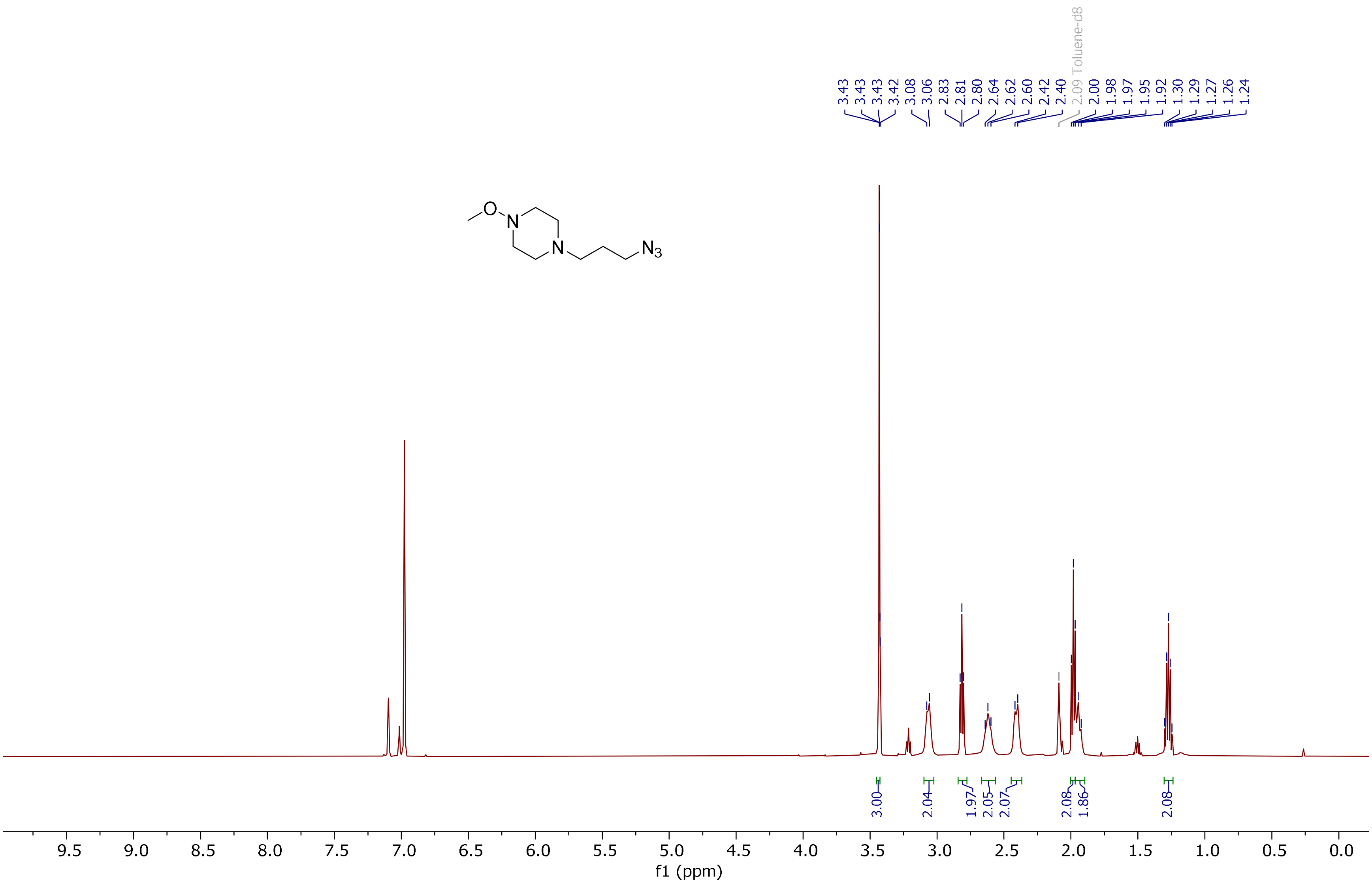

^{13}C NMR (126 MHz, Toluene-D$_8$) spectrum of 1-(3-azidopropyl)-4-methoxypiperazine **(31)** at 298 K

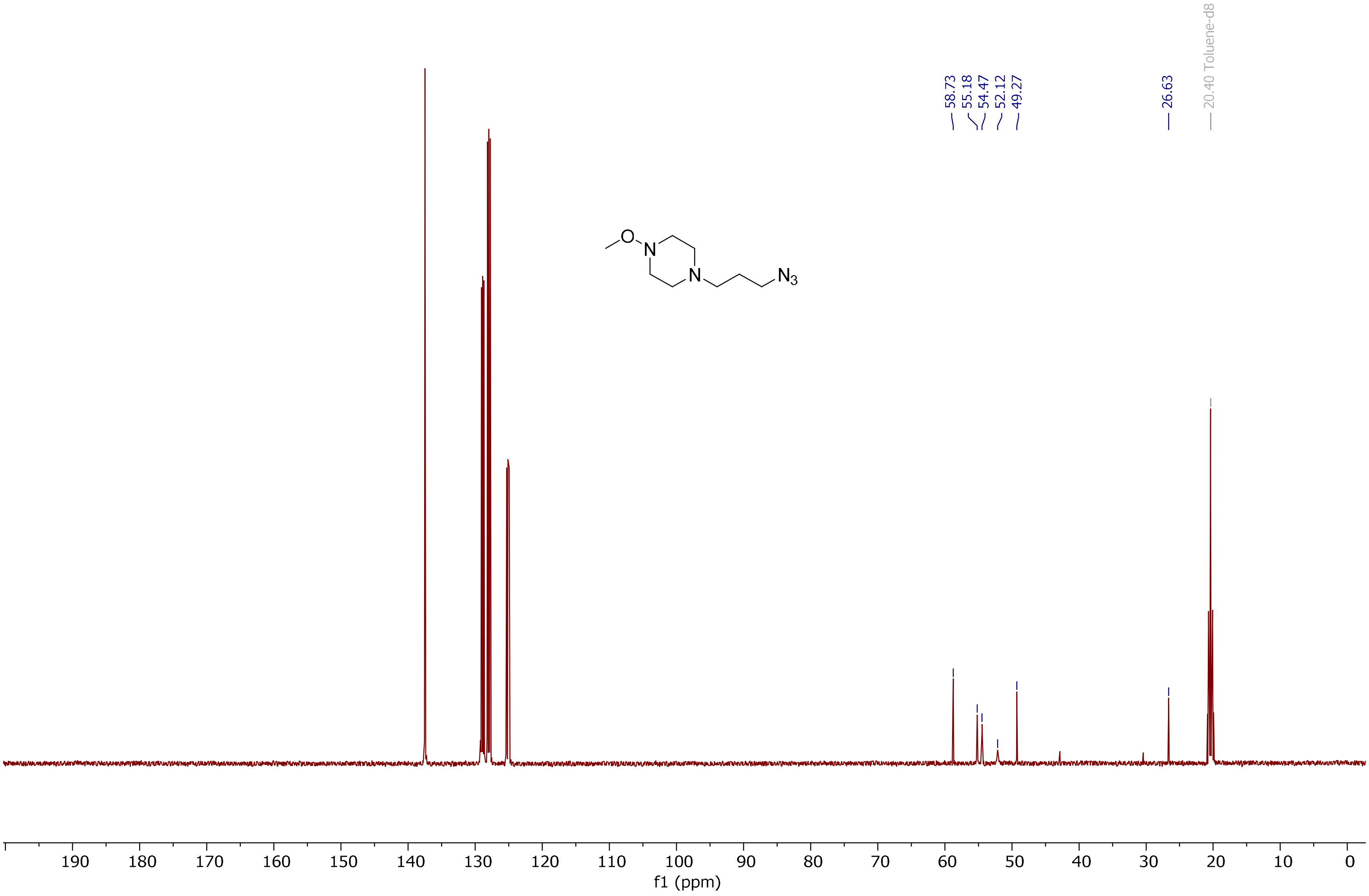

^{13}C NMR (151 MHz, Toluene-D$_8$) spectrum of 1-(3-azidopropyl)-4-methoxypiperazine **(31)** at 348 K

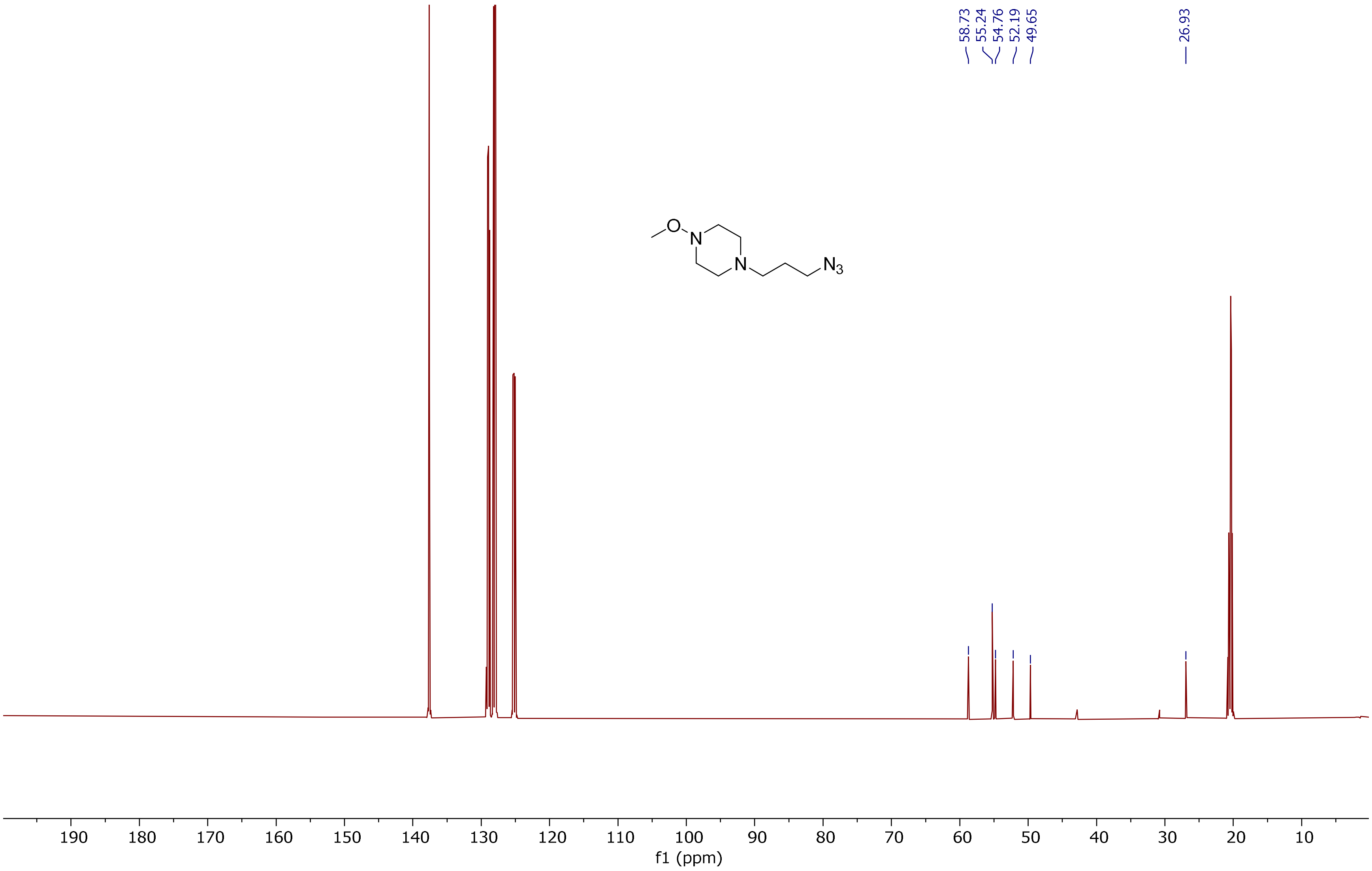

HSQC (Toluene-D8) spectrum of 1-(3-azidopropyl)-4-methoxypiperazine **(31)**

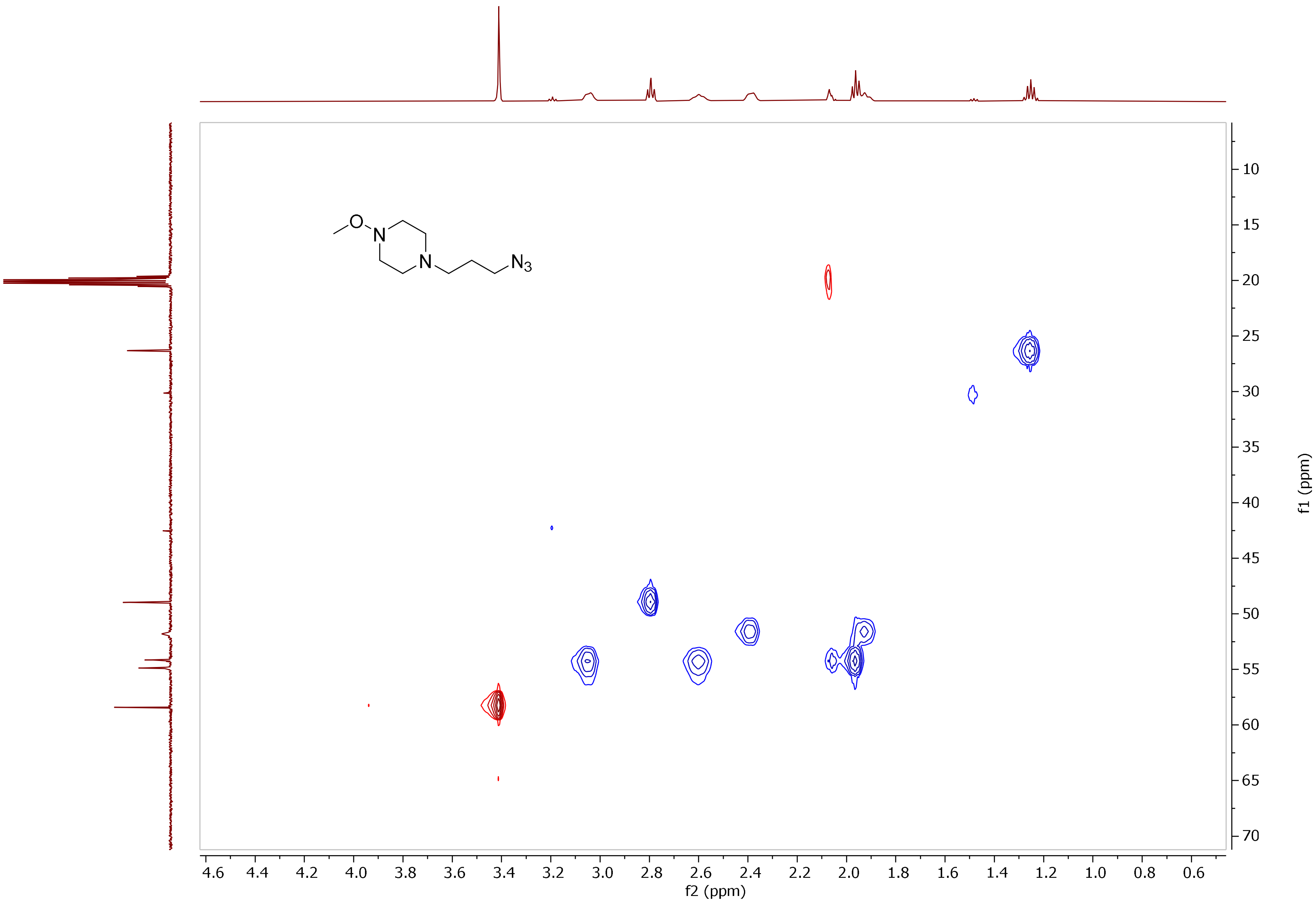

COSY (Toluene-D8) spectrum of 1-(3-azidopropyl)-4-methoxypiperazine **(31)**

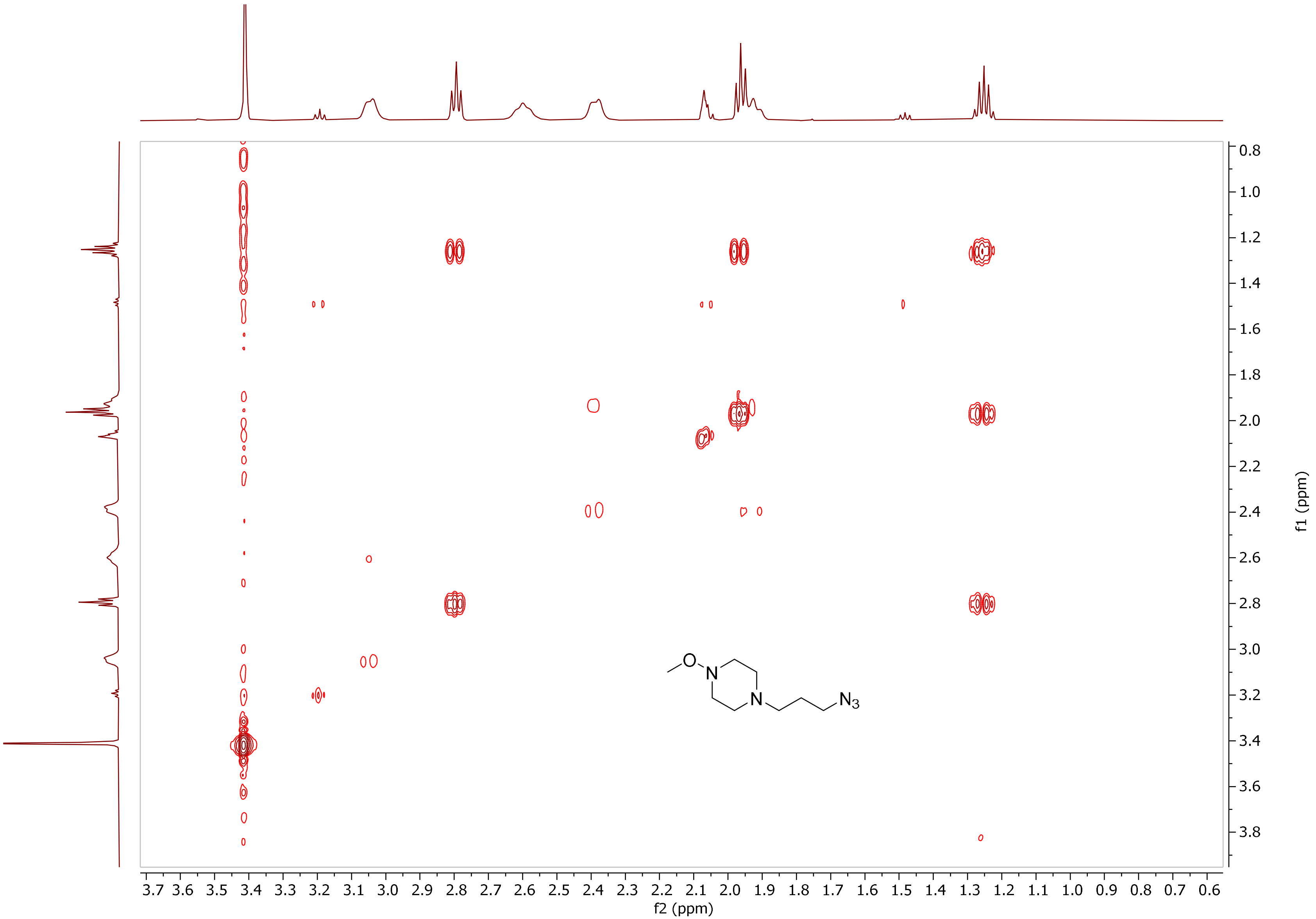

^{1}H NMR (500 MHz, $CDCl_3$) spectrum of 3-(4-methoxypiperazin-1-yl)propan-1-amine **(32)**

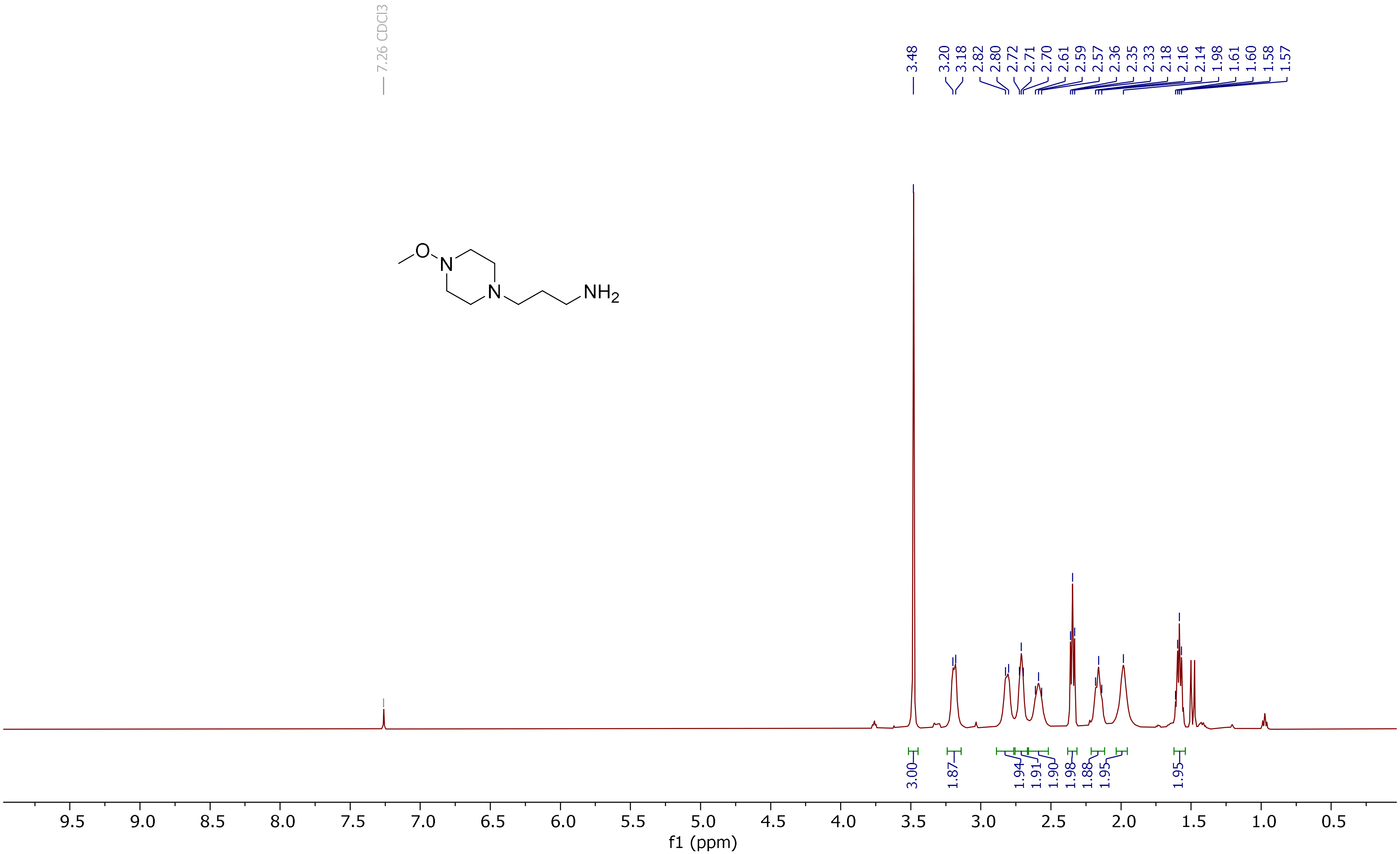

^{13}C NMR (126 MHz, $CDCl_3$) spectrum of 3-(4-methoxypiperazin-1-yl)propan-1-amine **(32)** at 298 K

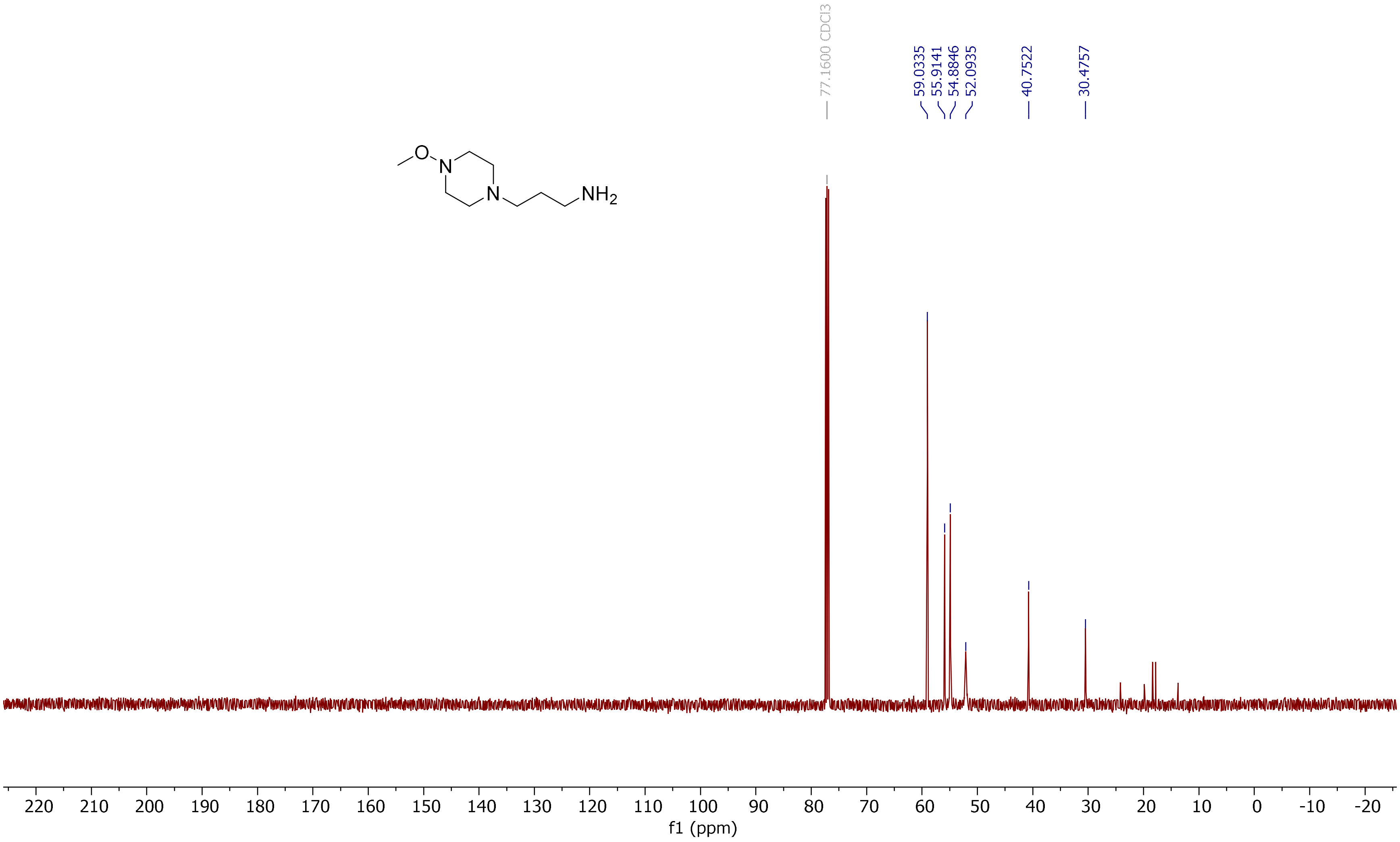

^{13}C NMR (151 MHz, Toluene-D_8) spectrum of 3-(4-methoxypiperazin-1-yl)propan-1-amine **(32)** at 348 K

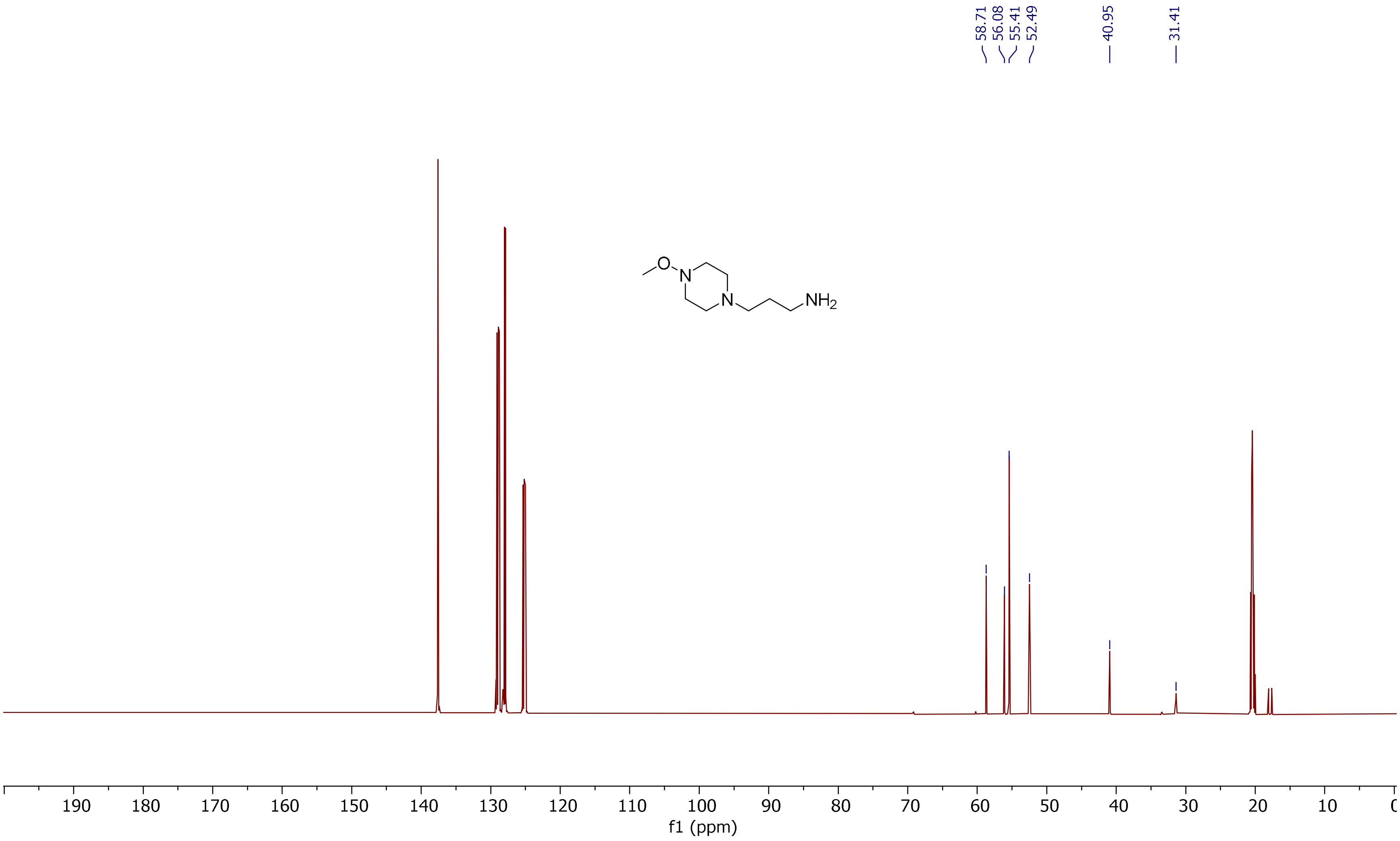

HSQC ($CDCl_3$) spectrum of 3-(4-methoxypiperazin-1-yl)propan-1-amine **(32)**

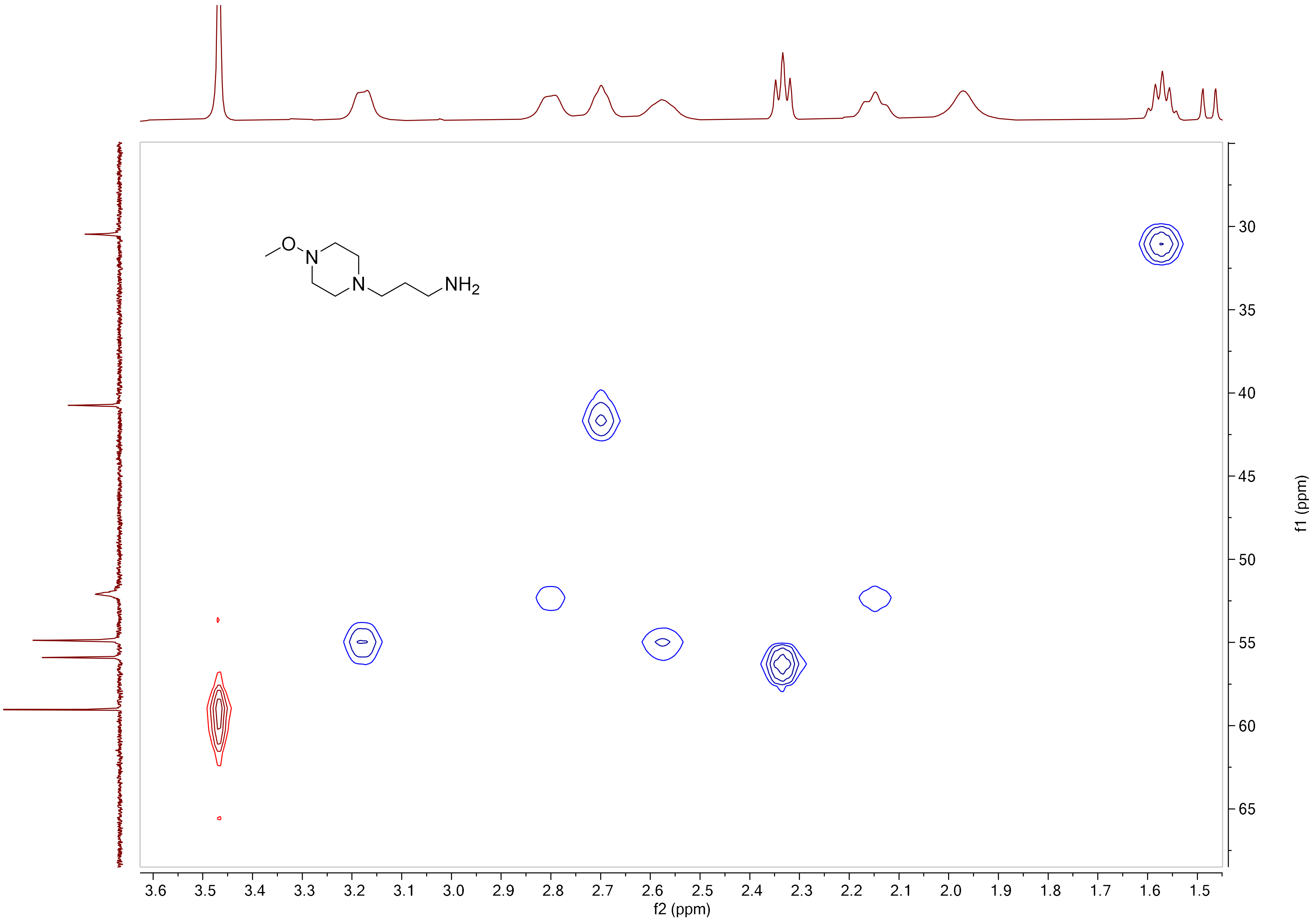

COSY ($CDCl_3$) spectrum of 3-(4-methoxypiperazin-1-yl)propan-1-amine **(32)**

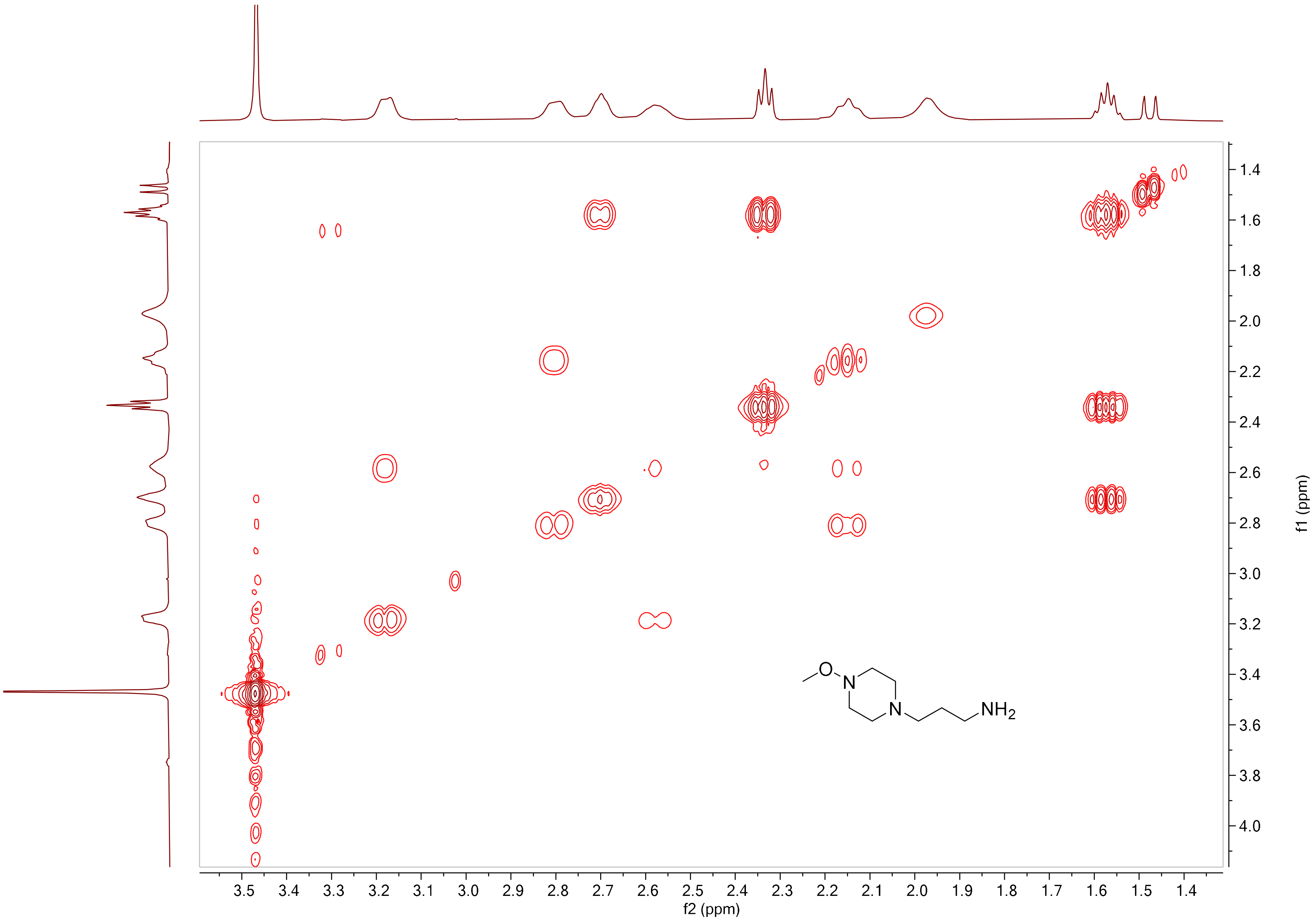

^{1}H NMR (500 MHz, $CDCl_3$) spectrum of 6-(2-chloro-4-(6-methylpyrazin-2-yl)phenyl)-8-ethyl-2-((3-(4-methoxypiperazin-1-yl)propyl)amino)pyrido[2,3-*d*]pyrimidin-7(8*H*)-one **(10)**

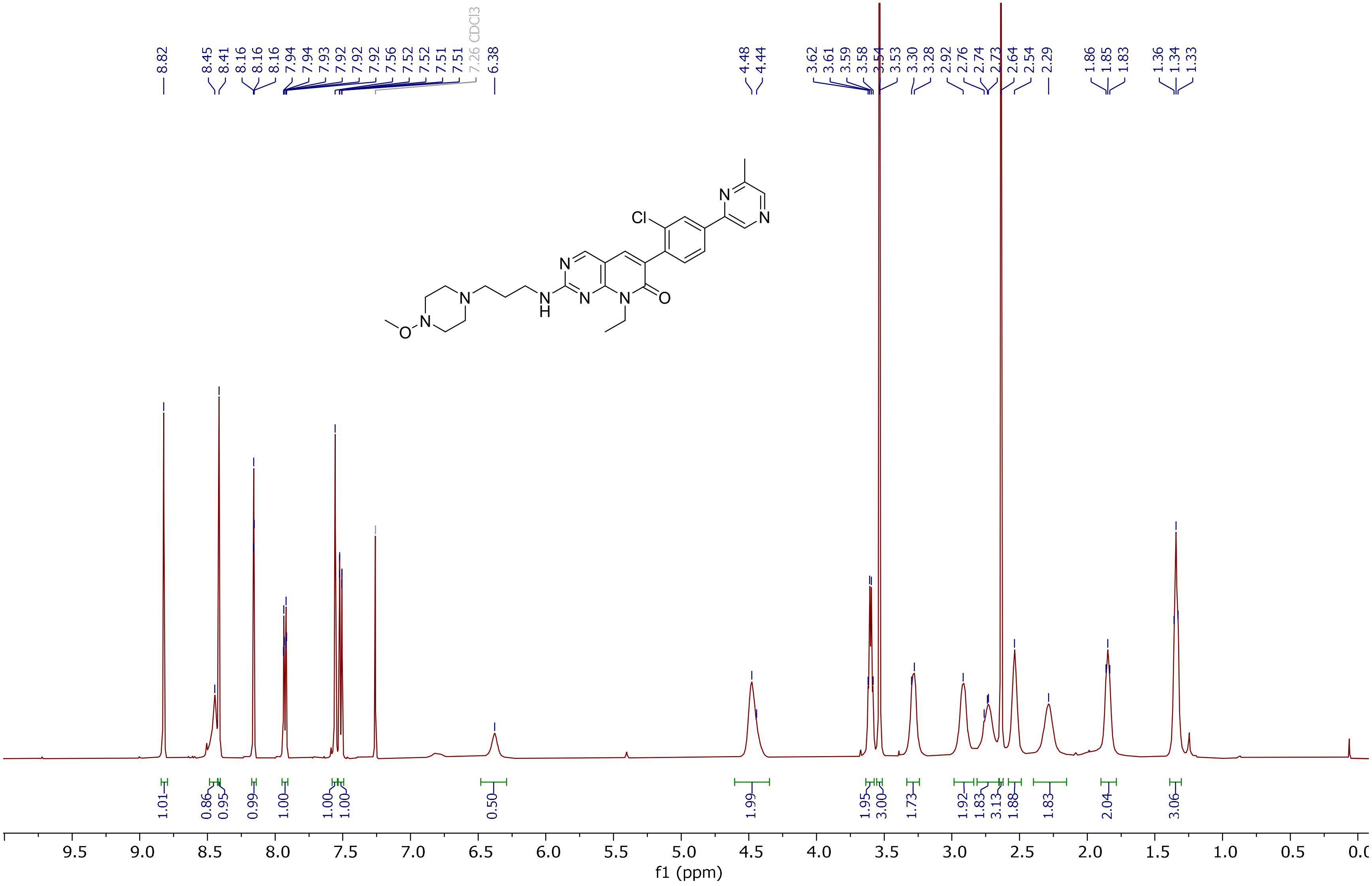

^{13}C NMR (126 MHz, $CDCl_3$) spectrum of 6-(2-chloro-4-(6-methylpyrazin-2-yl)phenyl)-8-ethyl-2-((3-(4-methoxypiperazin-1-yl)propyl)amino)pyrido[2,3-*d*]pyrimidin-7(8*H*)-one **(10)**

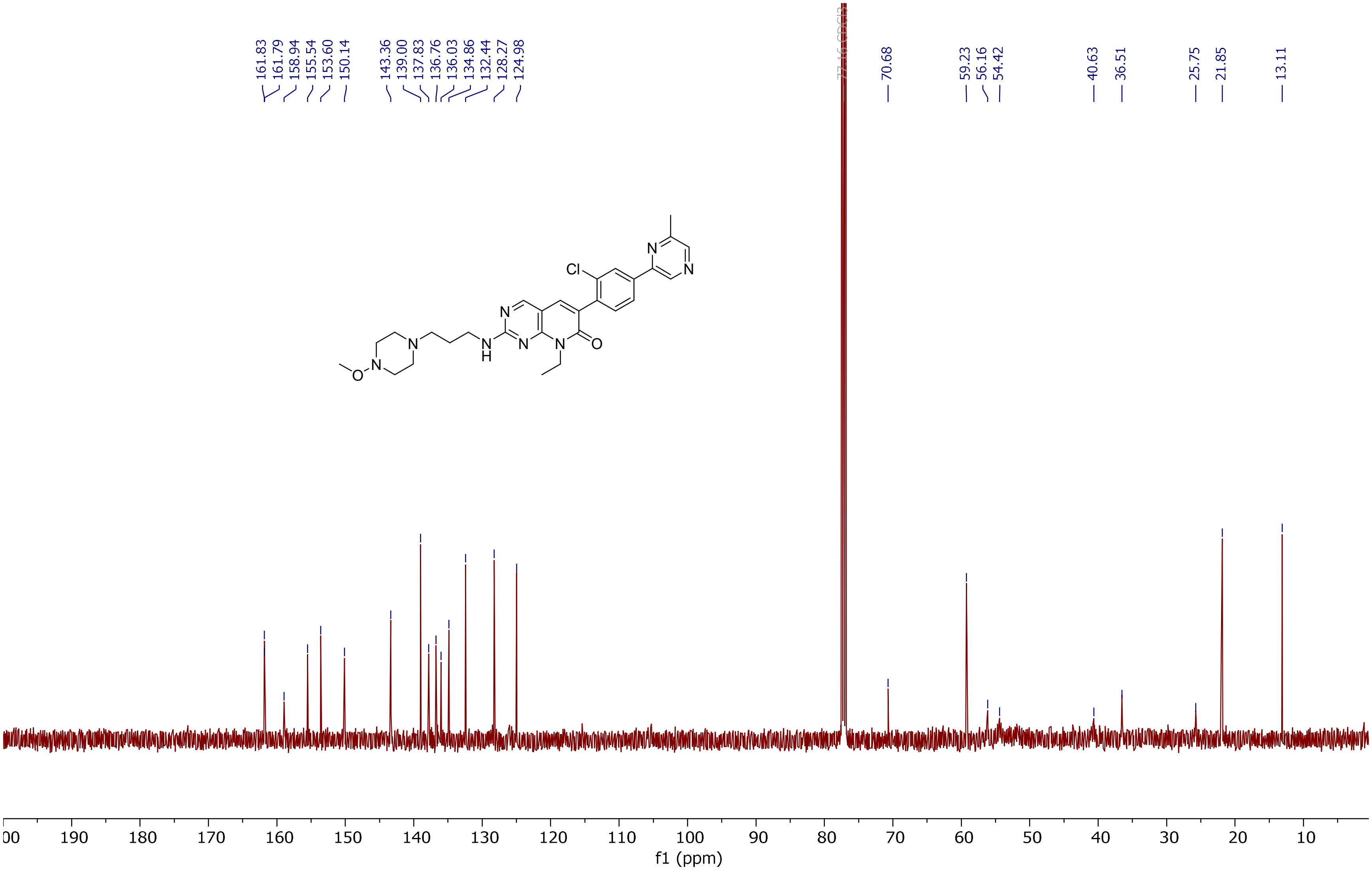

HSQC ($CDCl_3$) spectrum of 6-(2-chloro-4-(6-methylpyrazin-2-yl)phenyl)-8-ethyl-2-((3-(4-methoxypiperazin-1-yl)propyl)amino)pyrido[2,3-*d*]pyrimidin-7(8*H*)-one **(10)**

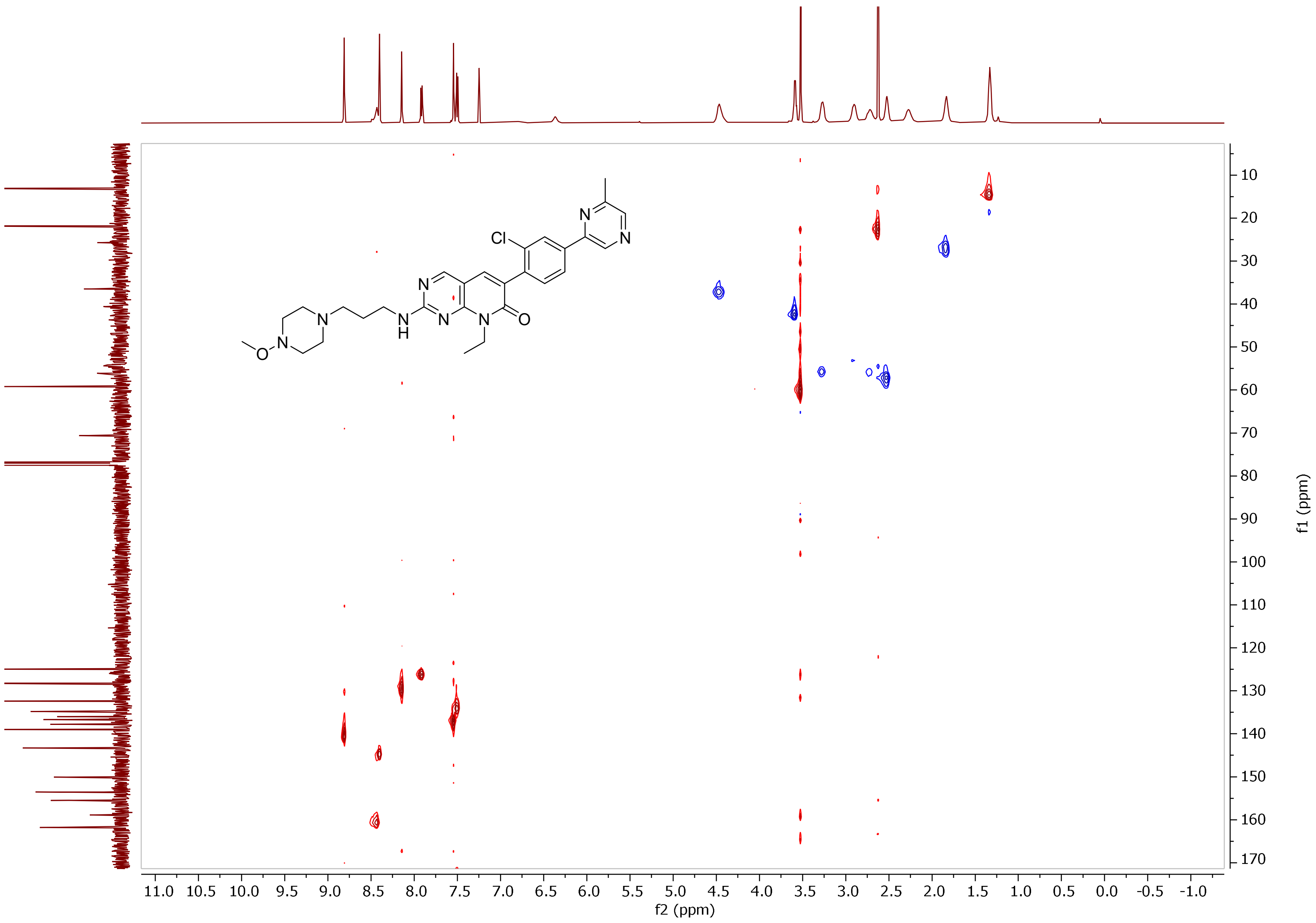

COSY ($CDCl_3$) spectrum of 6-(2-chloro-4-(6-methylpyrazin-2-yl)phenyl)-8-ethyl-2-((3-(4-methoxypiperazin-1-yl)propyl)amino)pyrido[2,3-*d*]pyrimidin-7(8*H*)-one **(10)**

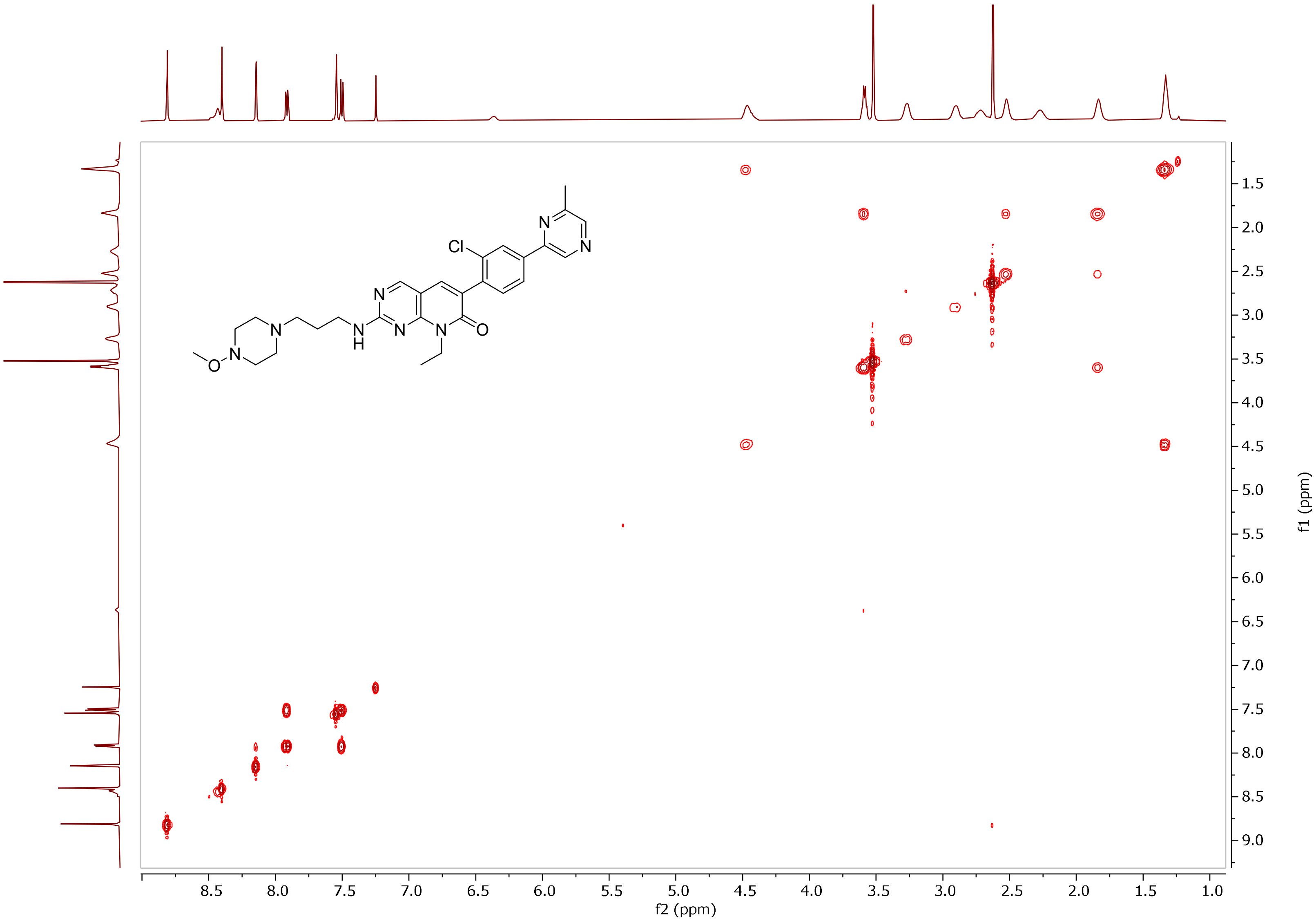

^{1}H NMR (500 MHz, $CDCl_3$) spectrum of 2-(2-(4-methoxypiperazin-1-yl)ethyl)isoindoline-1,3-dione **(34)**

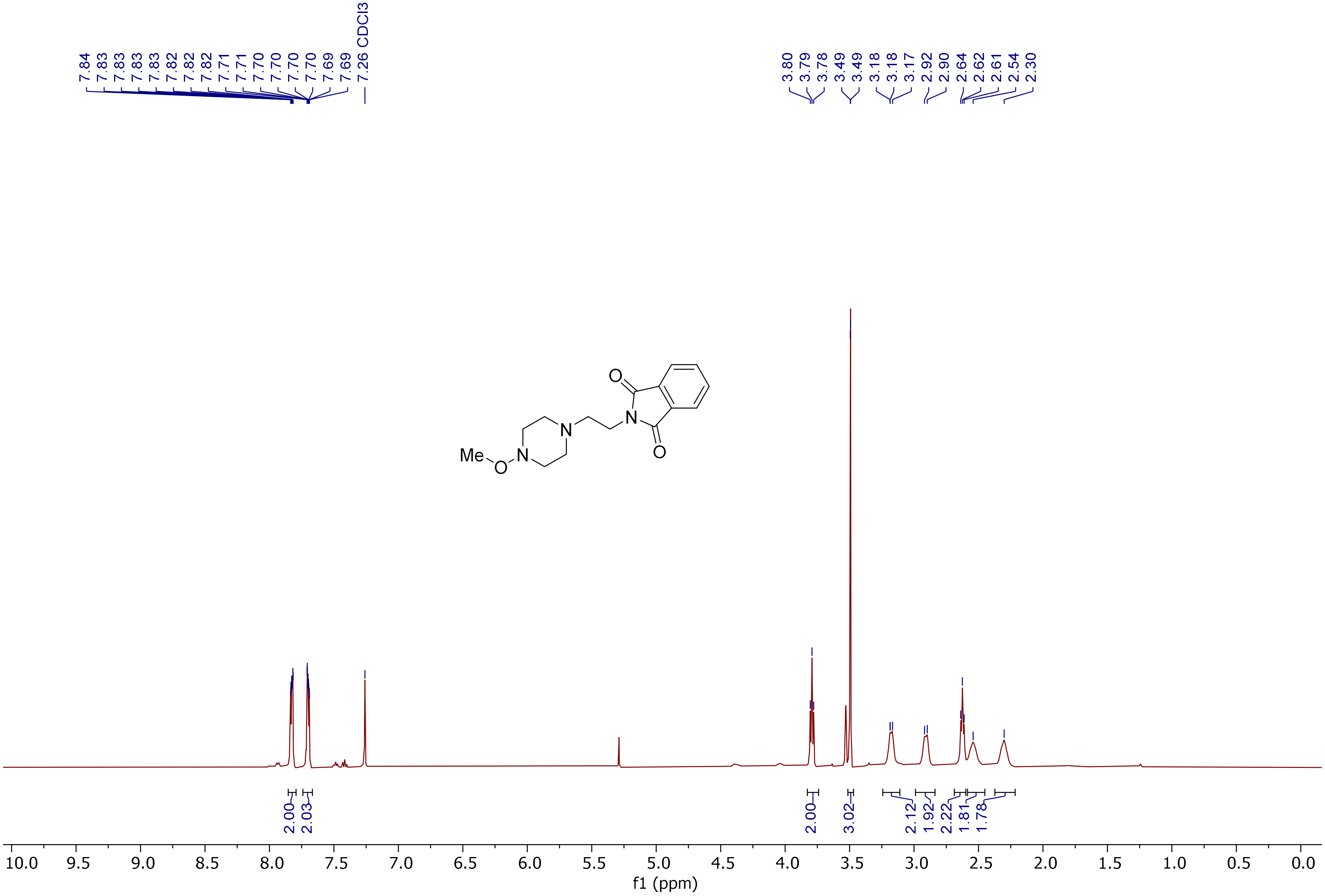

^{13}C NMR (126 MHz, $CDCl_3$) spectrum of 2-(2-(4-methoxypiperazin-1-yl)ethyl)isoindoline-1,3-dione **(34)**

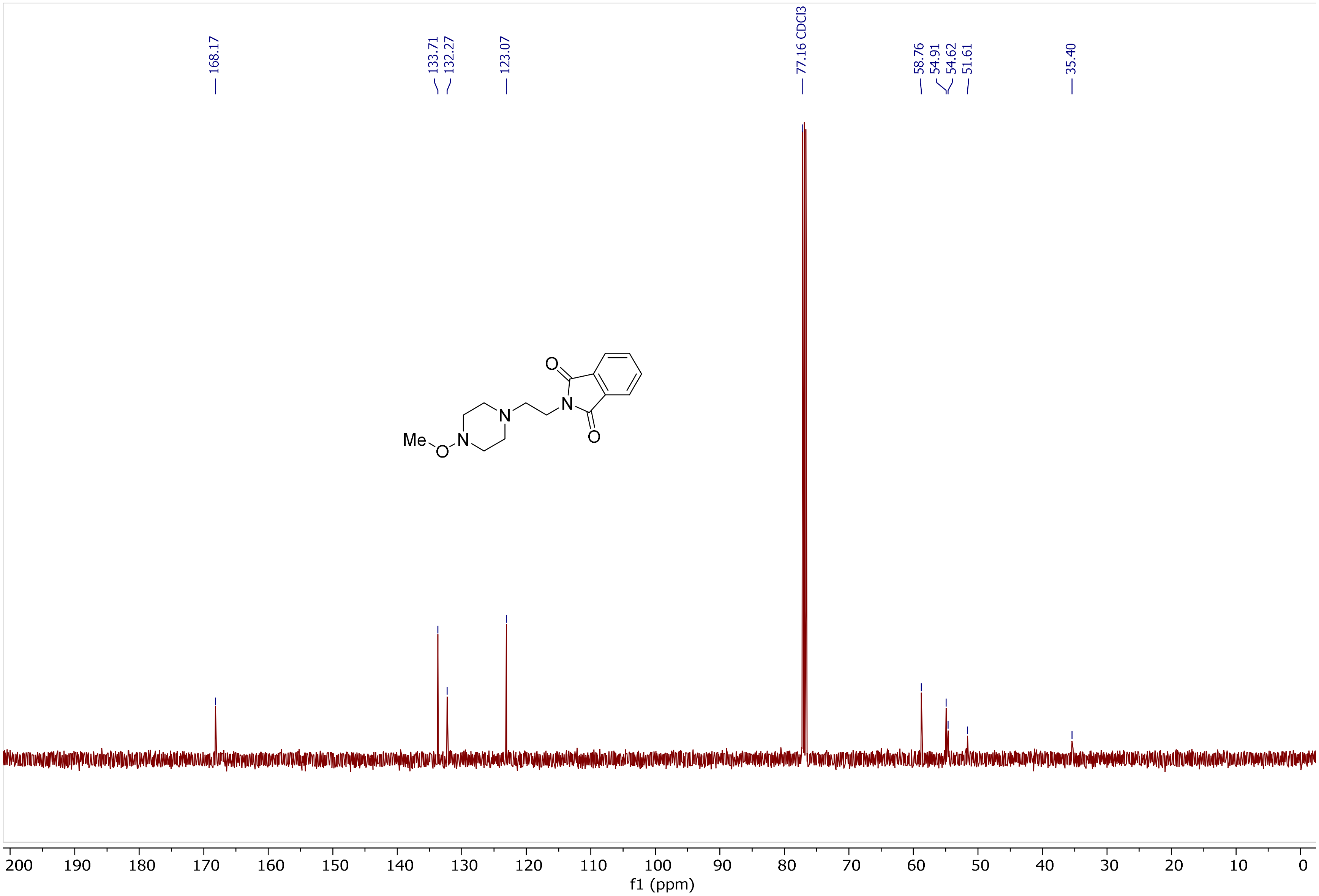

HSQC ($CDCl_3$) spectrum of 2-(2-(4-methoxypiperazin-1-yl)ethyl)isoindoline-1,3-dione **(34)**

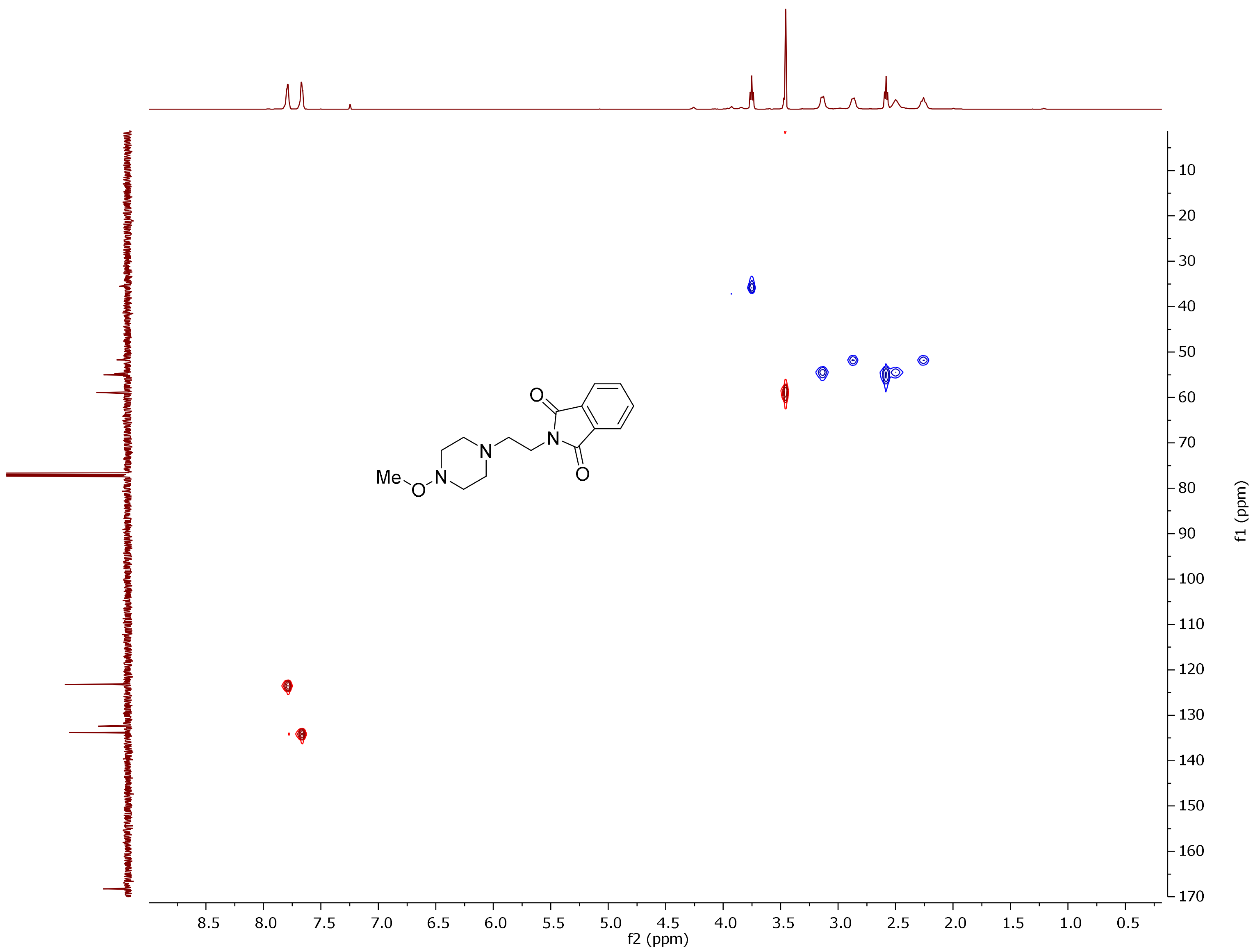

COSY ($CDCl_3$) spectrum of 2-(2-(4-methoxypiperazin-1-yl)ethyl)isoindoline-1,3-dione **(34)**

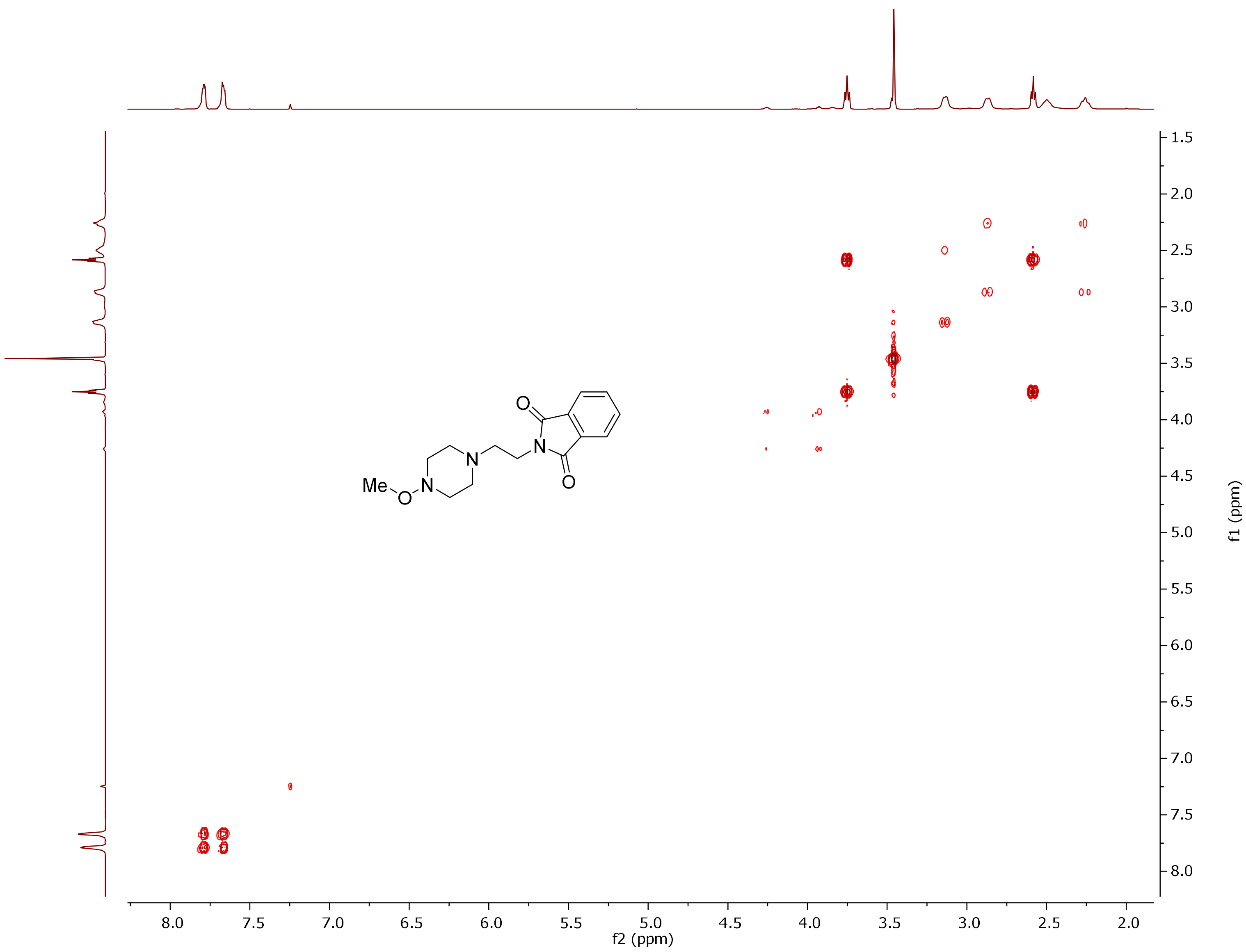

^{1}H NMR (500 MHz, $CDCl_3$) spectrum of 6-(2-chloro-4-(6-methylpyrazin-2-yl)phenyl)-8-ethyl-2-((2-(4-methoxypiperazin-1-yl)ethyl)amino)pyrido[2,3-*d*]pyrimidin-7(8*H*)-one **(11)**

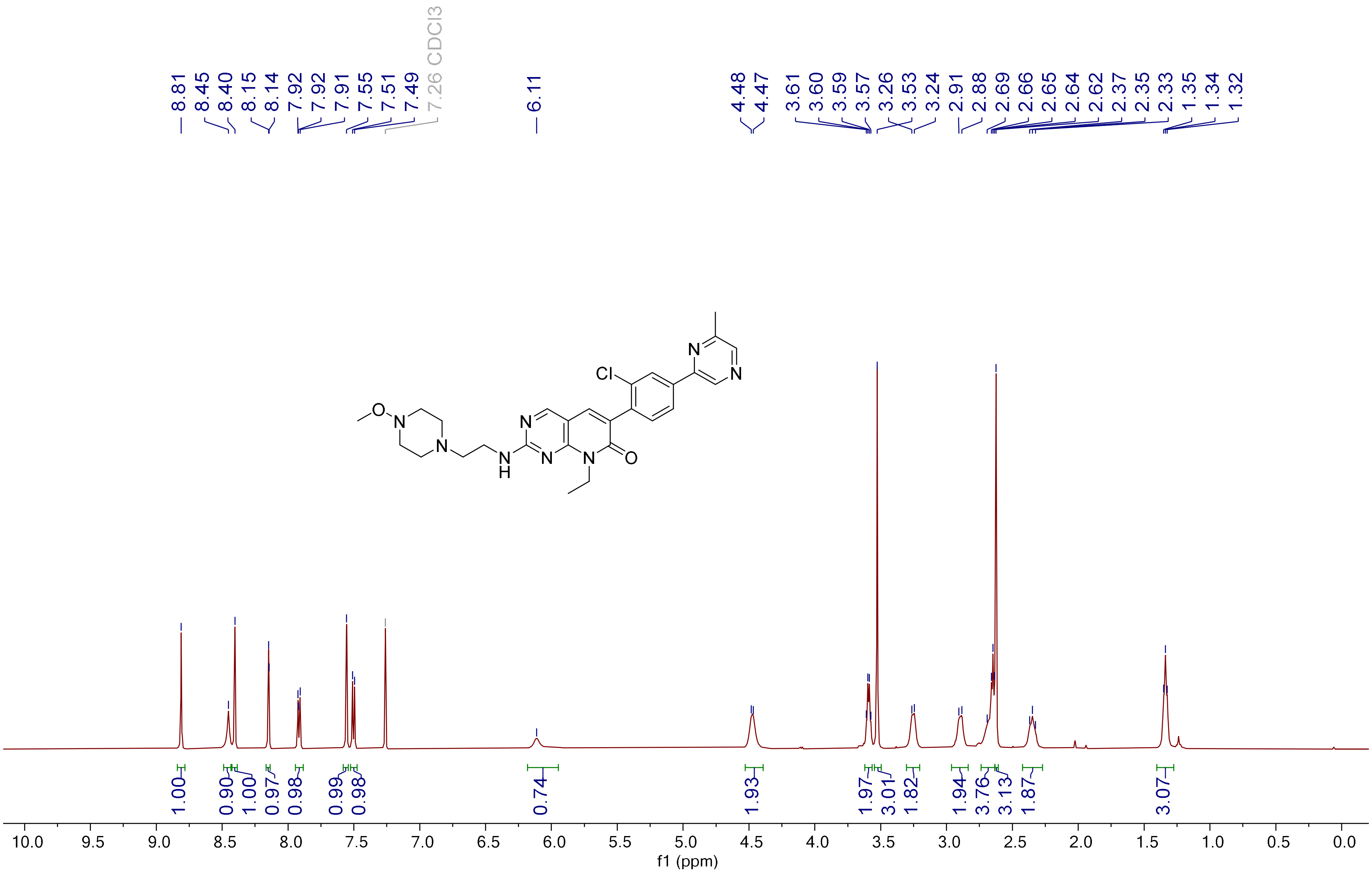

^{13}C NMR (126 MHz, $CDCl_3$) spectrum of 6-(2-chloro-4-(6-methylpyrazin-2-yl)phenyl)-8-ethyl-2-((2-(4-methoxypiperazin-1-yl)ethyl)amino)pyrido[2,3-*d*]pyrimidin-7(8*H*)-one **(11)**

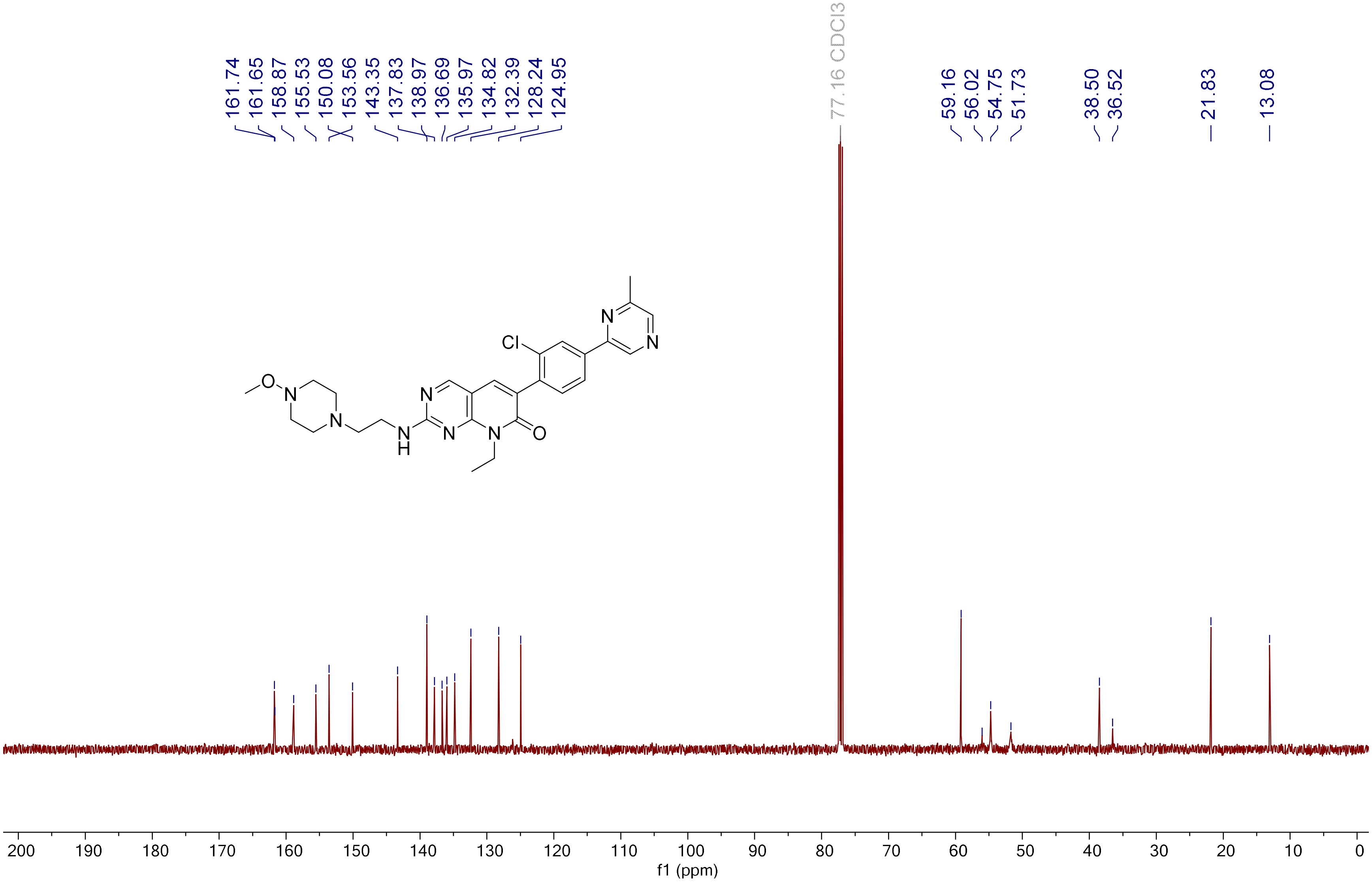

HSQC ($CDCl_3$) spectrum of 6-(2-chloro-4-(6-methylpyrazin-2-yl)phenyl)-8-ethyl-2-((2-(4-methoxypiperazin-1-yl)ethyl)amino)pyrido[2,3-*d*]pyrimidin-7(8*H*)-one **(11)**

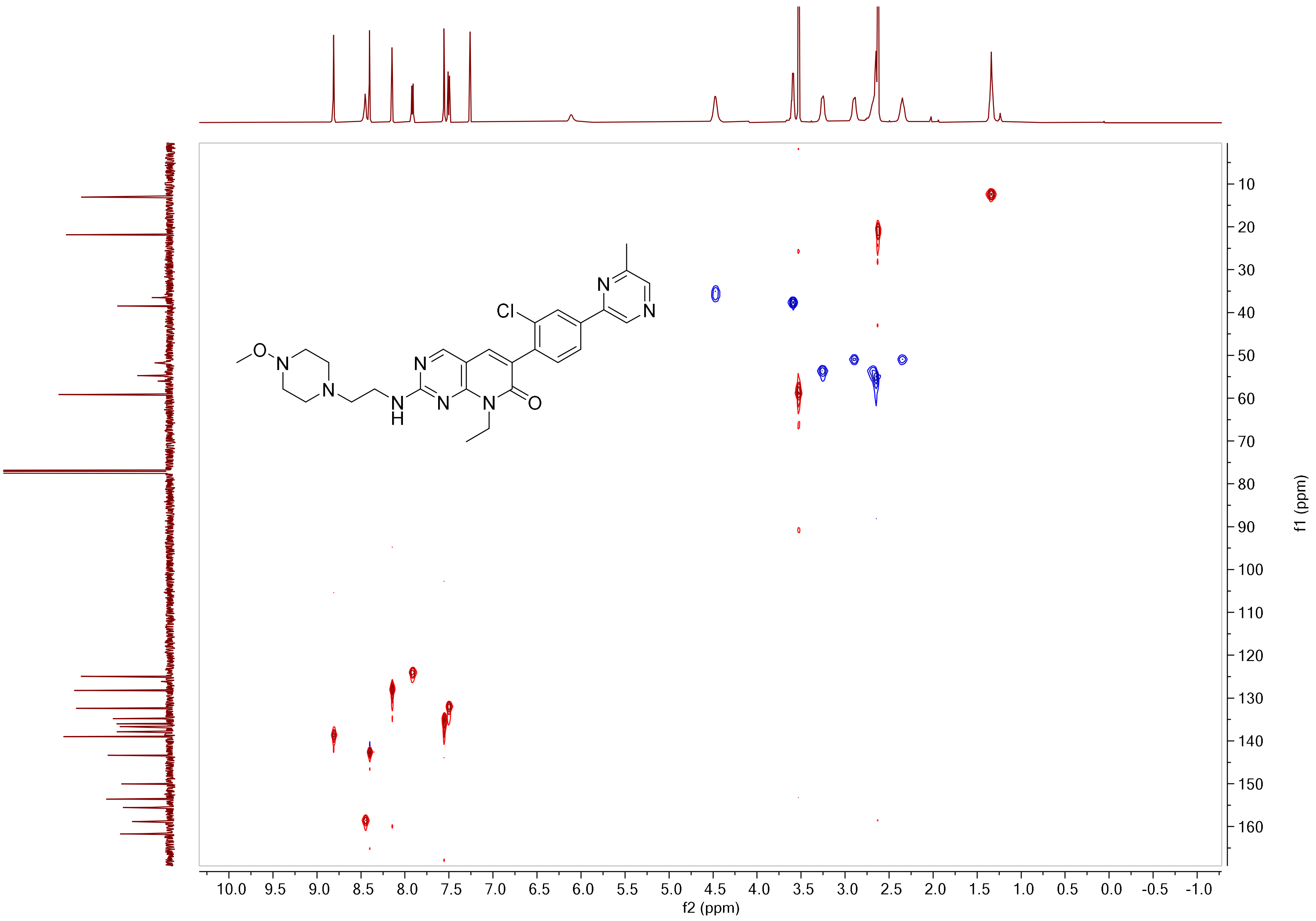

COSY ($CDCl_3$) spectrum of 6-(2-chloro-4-(6-methylpyrazin-2-yl)phenyl)-8-ethyl-2-((2-(4-methoxypiperazin-1-yl)ethyl)amino)pyrido[2,3-*d*]pyrimidin-7(8*H*)-one **(11)**

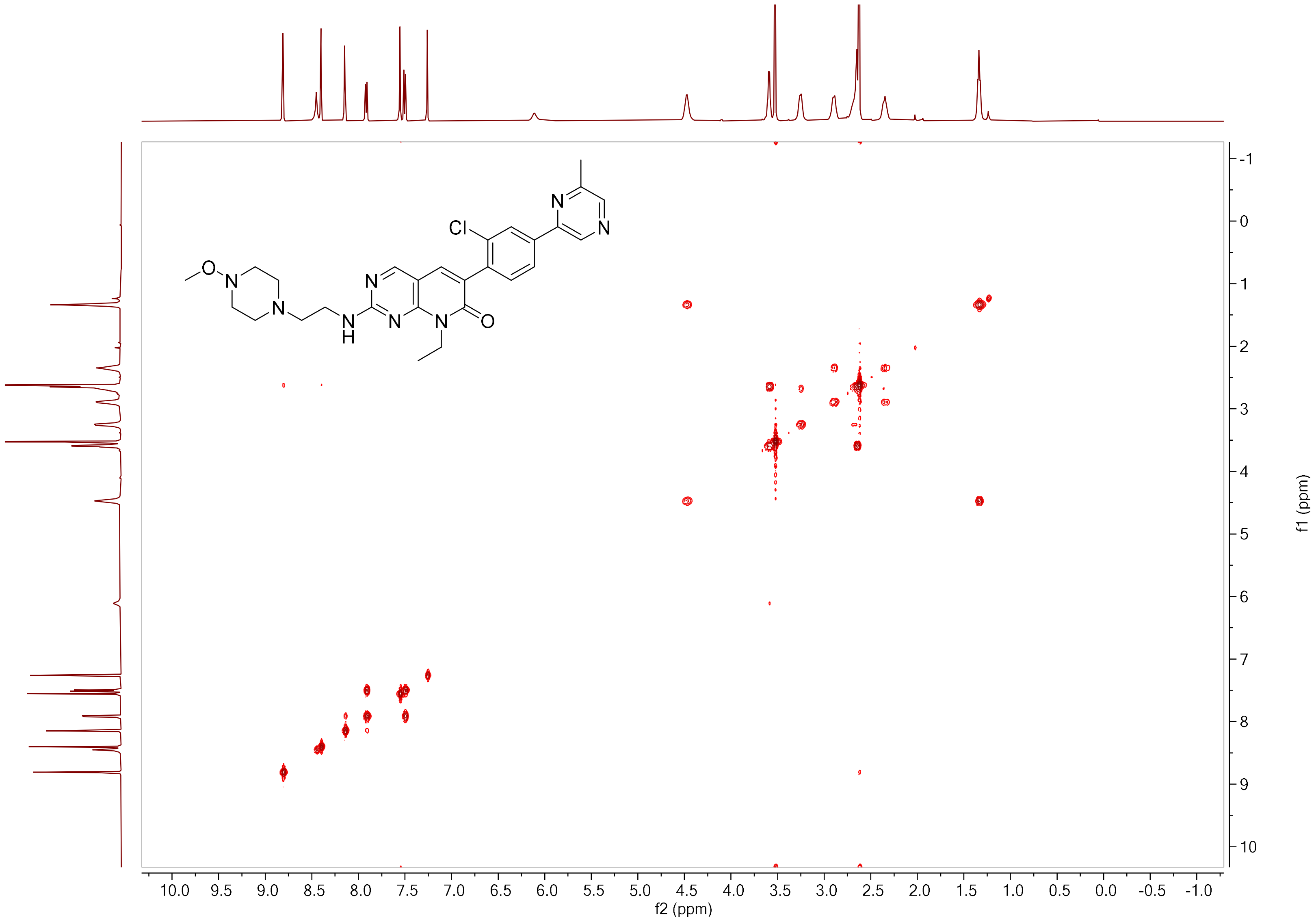

^{1}H NMR (500 MHz, $CDCl_3$) spectrum of Allyl hydroxycarbamate

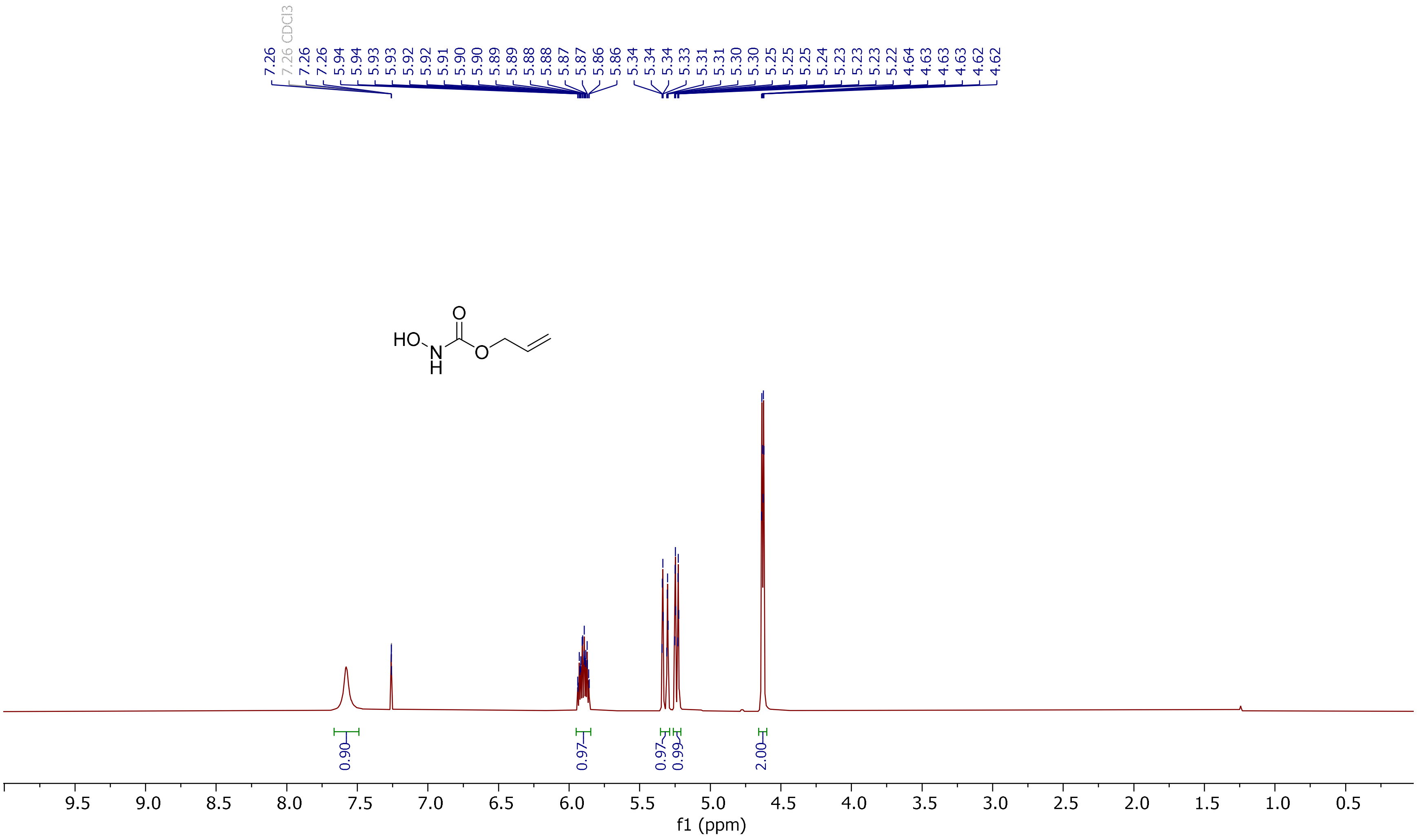

^{13}C NMR (126 MHz, $CDCl_3$) spectrum of Allyl hydroxycarbamate

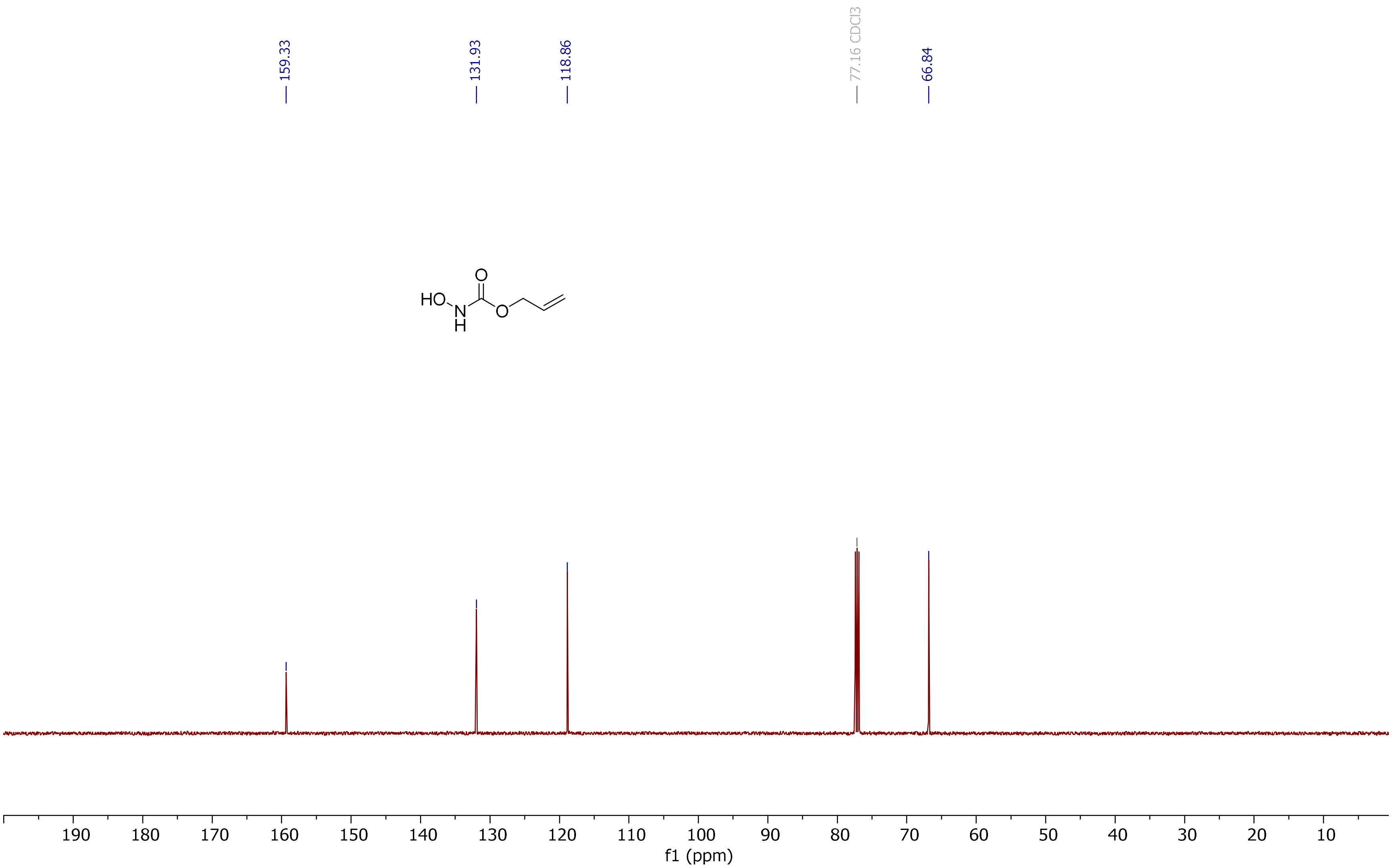

S112

HSQC ($CDCl_3$) spectrum of Allyl hydroxycarbamate

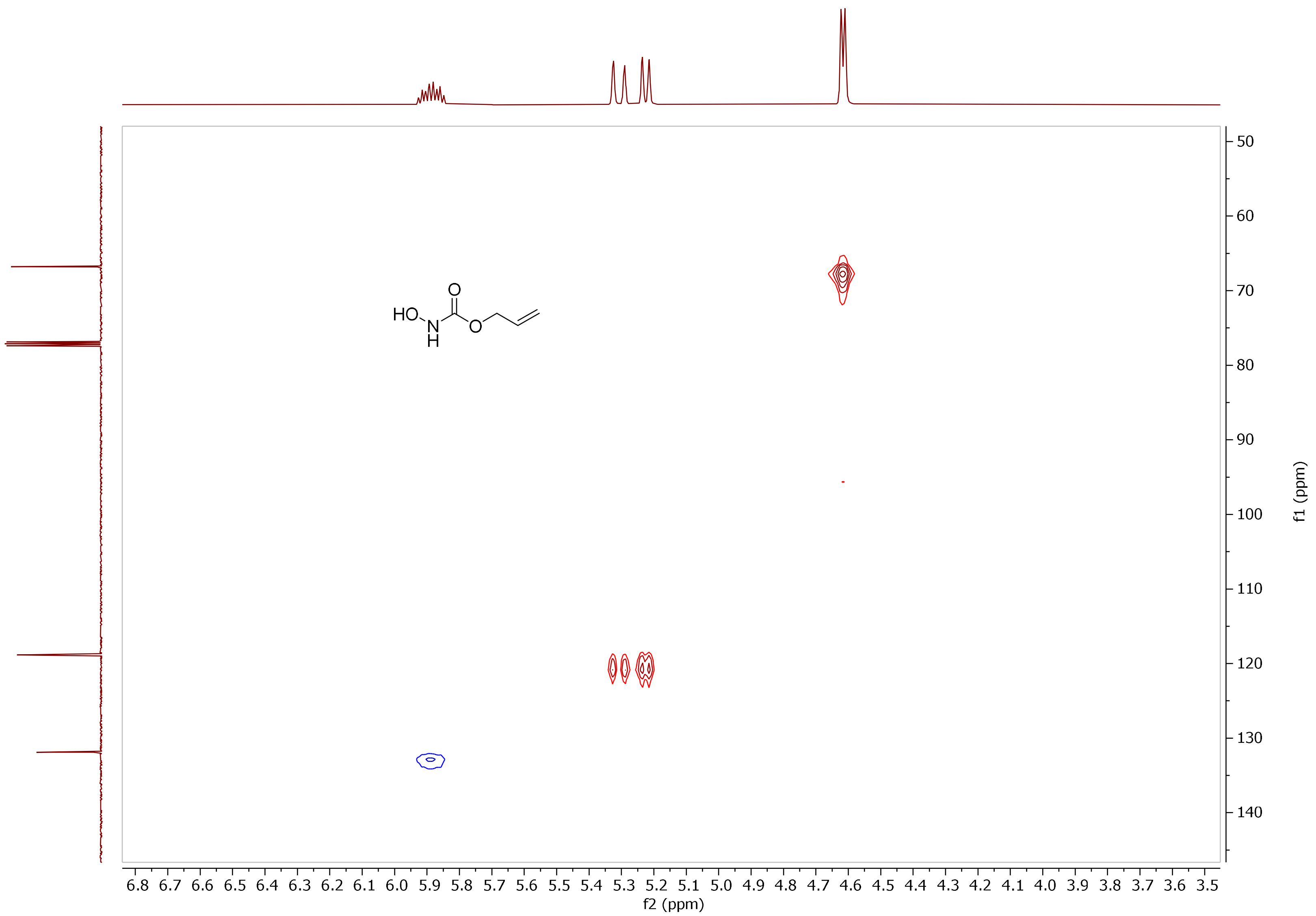

S113

COSY (CDCl$_3$) spectrum of Allyl hydroxycarbamate

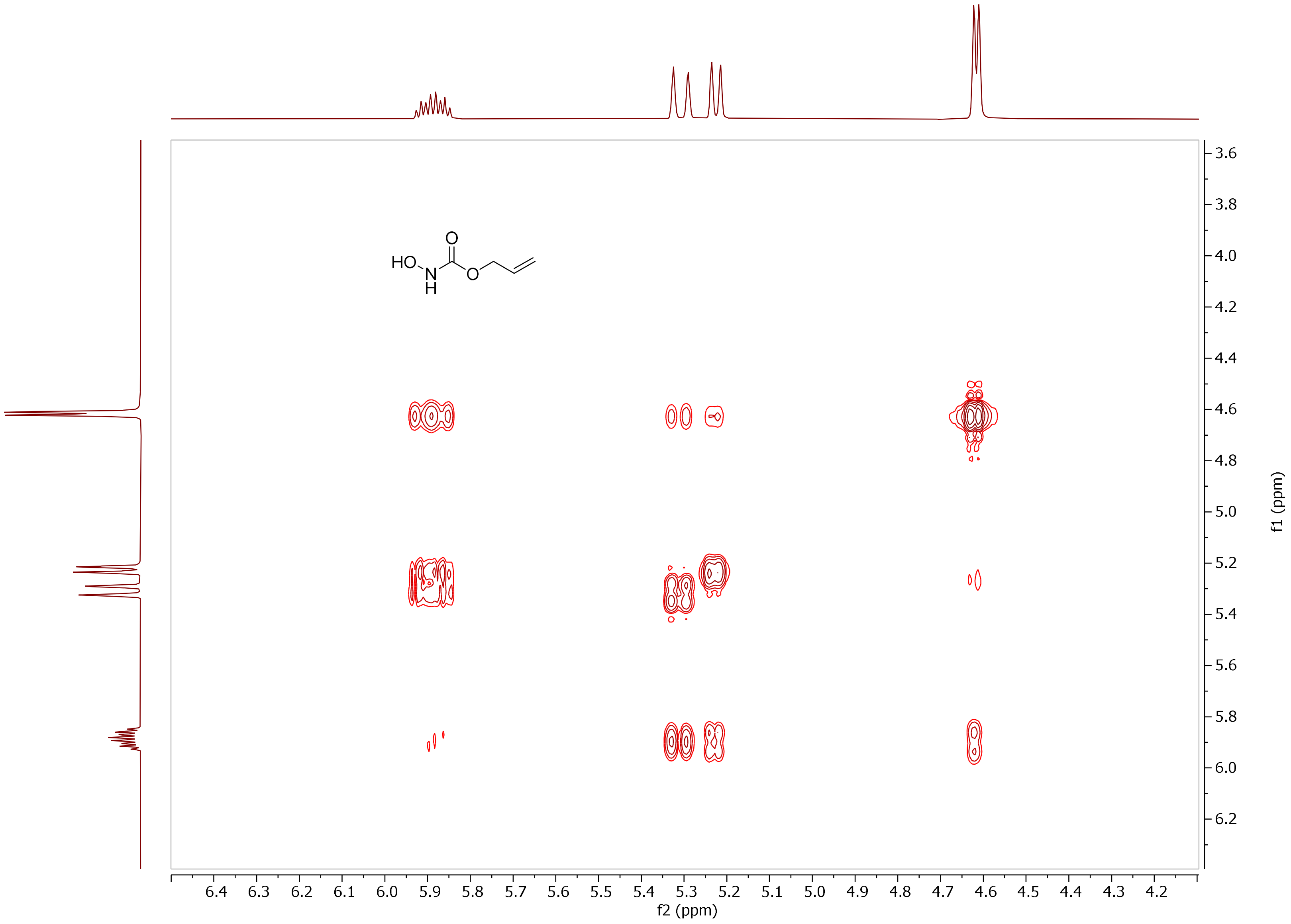

^{1}H NMR (500 MHz, $CDCl_3$) spectrum of 2-allyl 5-(*tert*-butyl) 1,2,5-oxadiazepane-2,5-dicarboxylate **(37)**

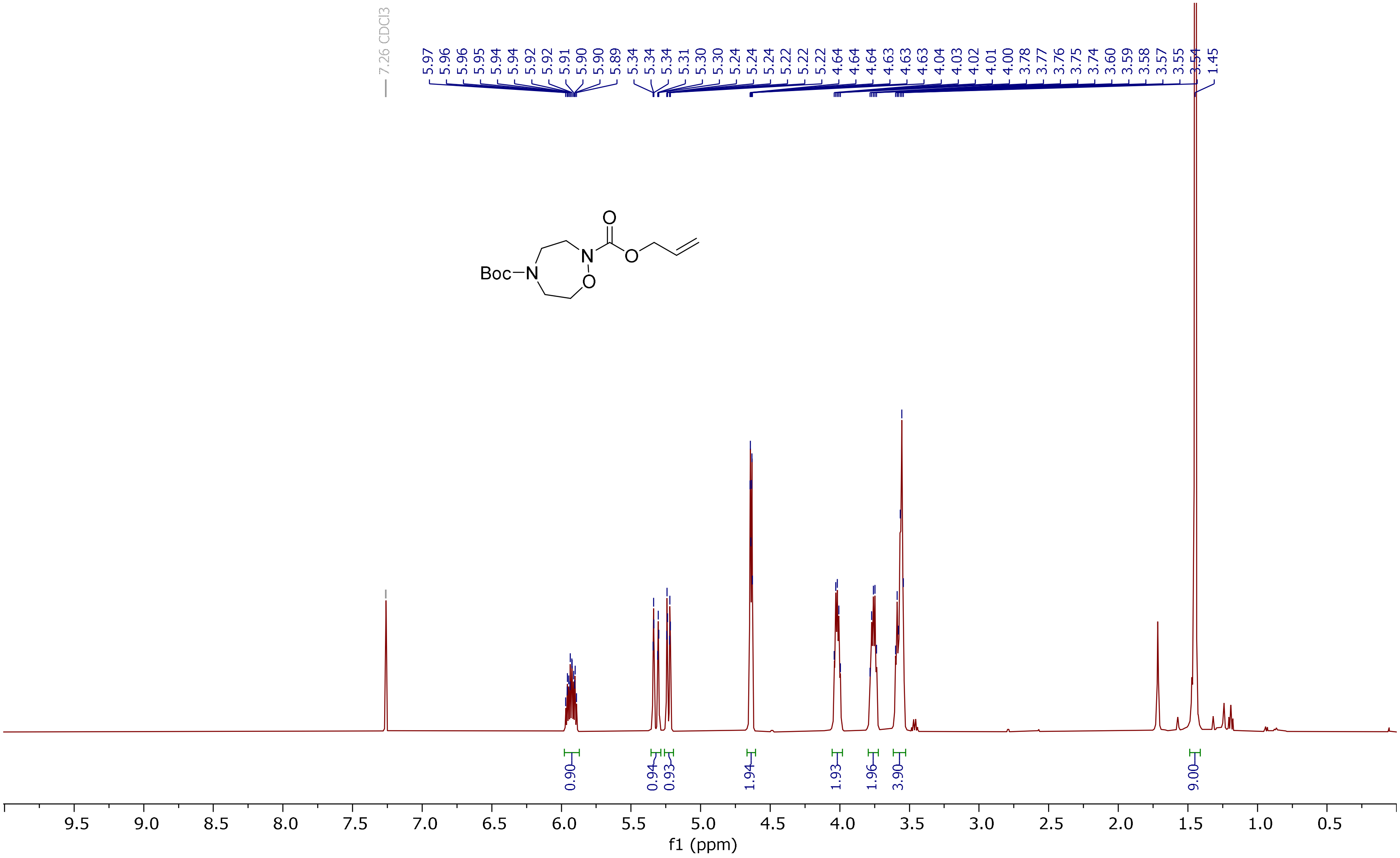

^{13}C NMR (126 MHz, $CDCl_3$) spectrum of 2-allyl 5-(*tert*-butyl) 1,2,5-oxadiazepane-2,5-dicarboxylate **(37)**

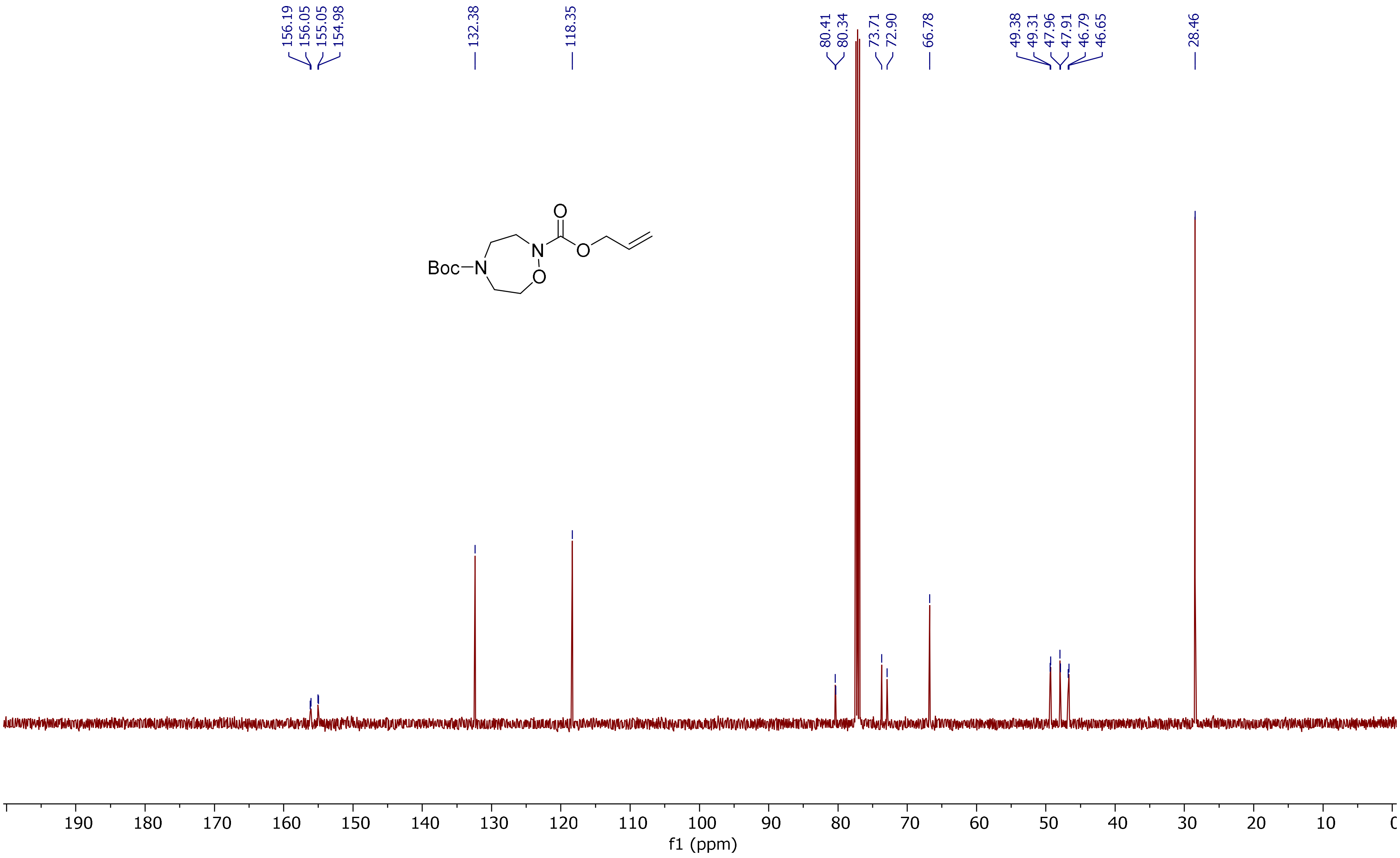

HSQC ($CDCl_3$) spectrum of 2-allyl 5-(*tert*-butyl) 1,2,5-oxadiazepane-2,5-dicarboxylate **(37)**

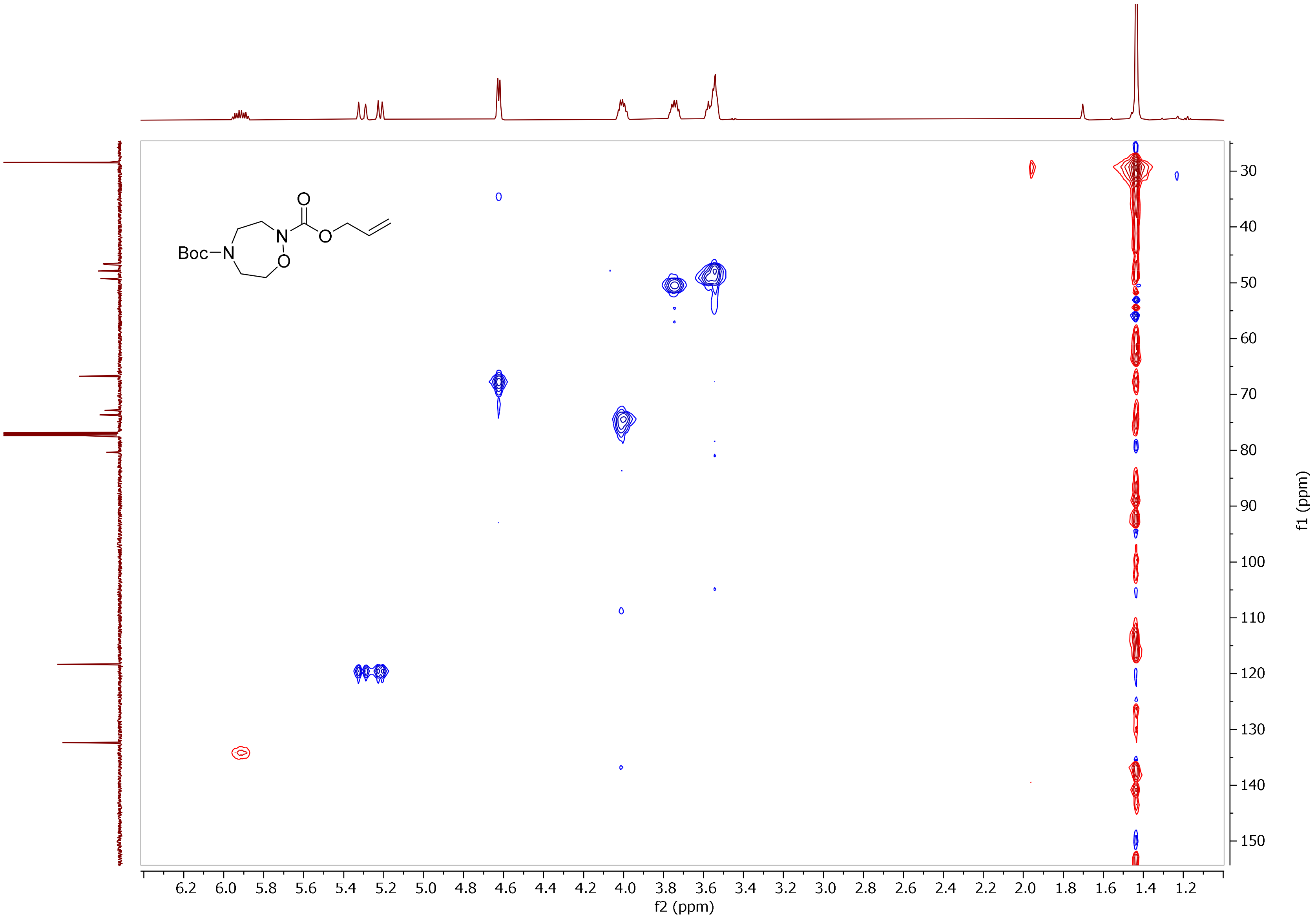

COSY ($CDCl_3$) spectrum of 2-allyl 5-(*tert*-butyl) 1,2,5-oxadiazepane-2,5-dicarboxylate **(37)**

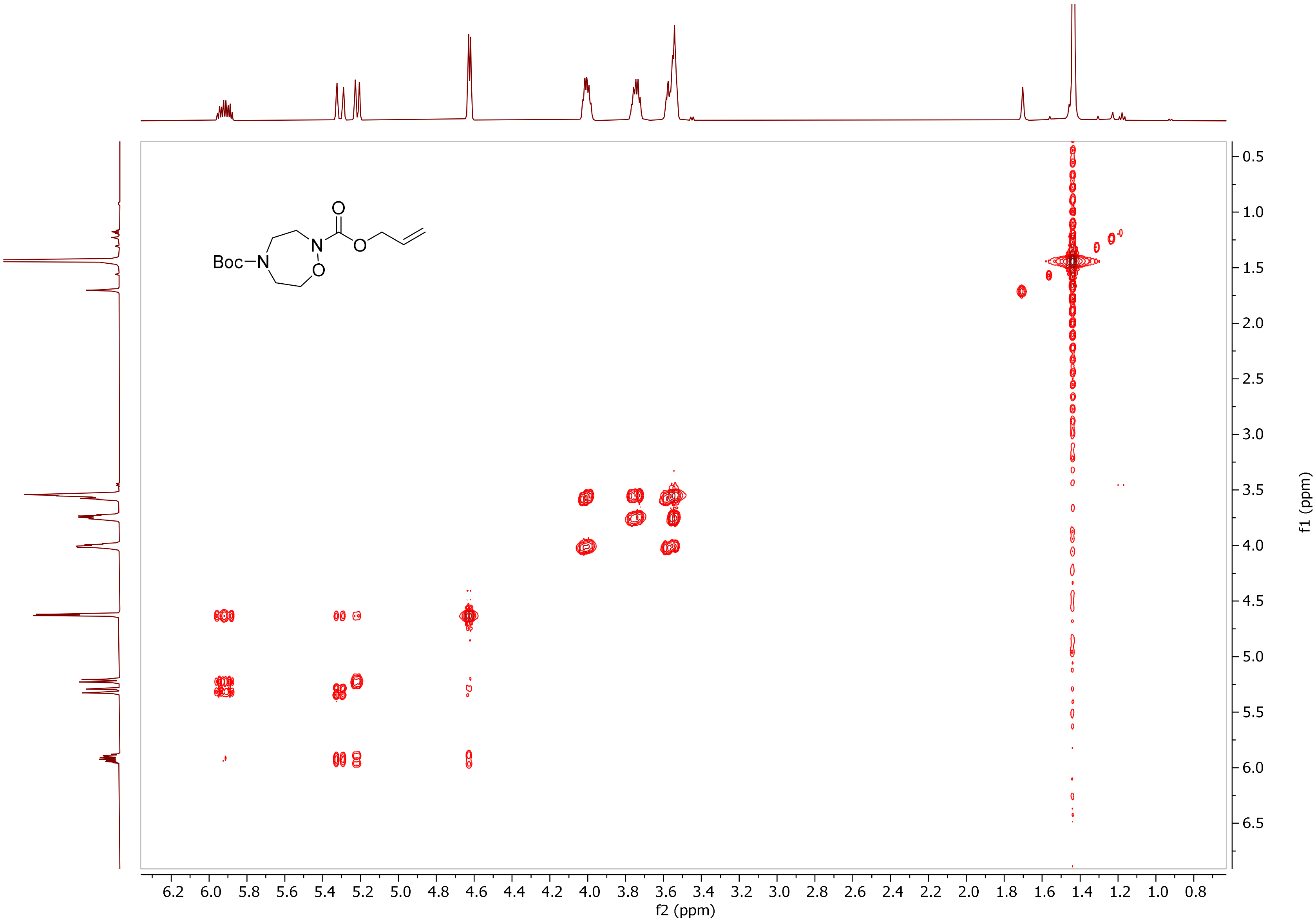

^{1}H NMR (500 MHz, $CDCl_3$) spectrum of *tert*-butyl 2-allyl-1,2,5-oxadiazepane-5-carboxylate **(38)**

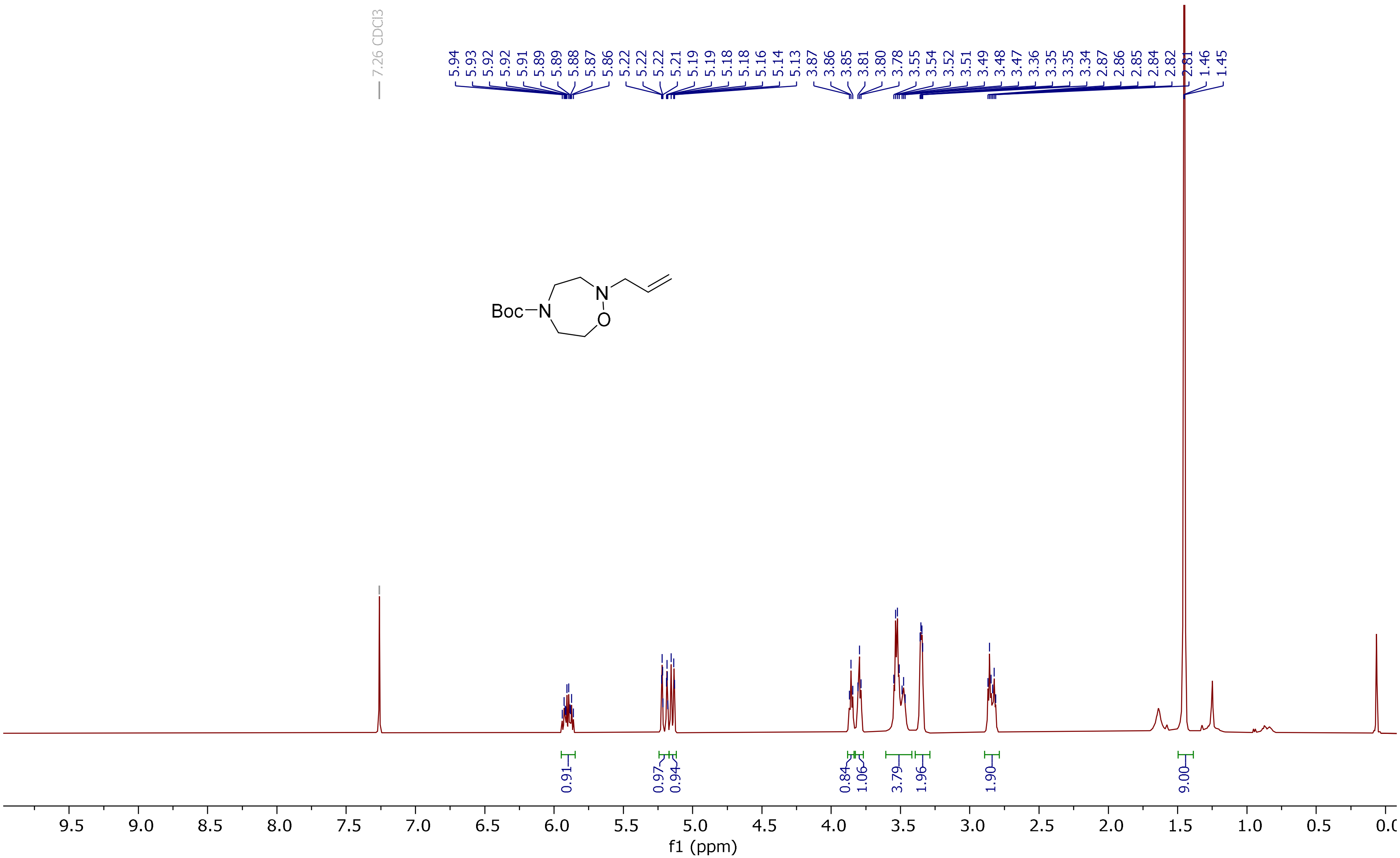

^{13}C NMR (126 MHz, $CDCl_3$) spectrum of *tert*-butyl 2-allyl-1,2,5-oxadiazepane-5-carboxylate **(38)**

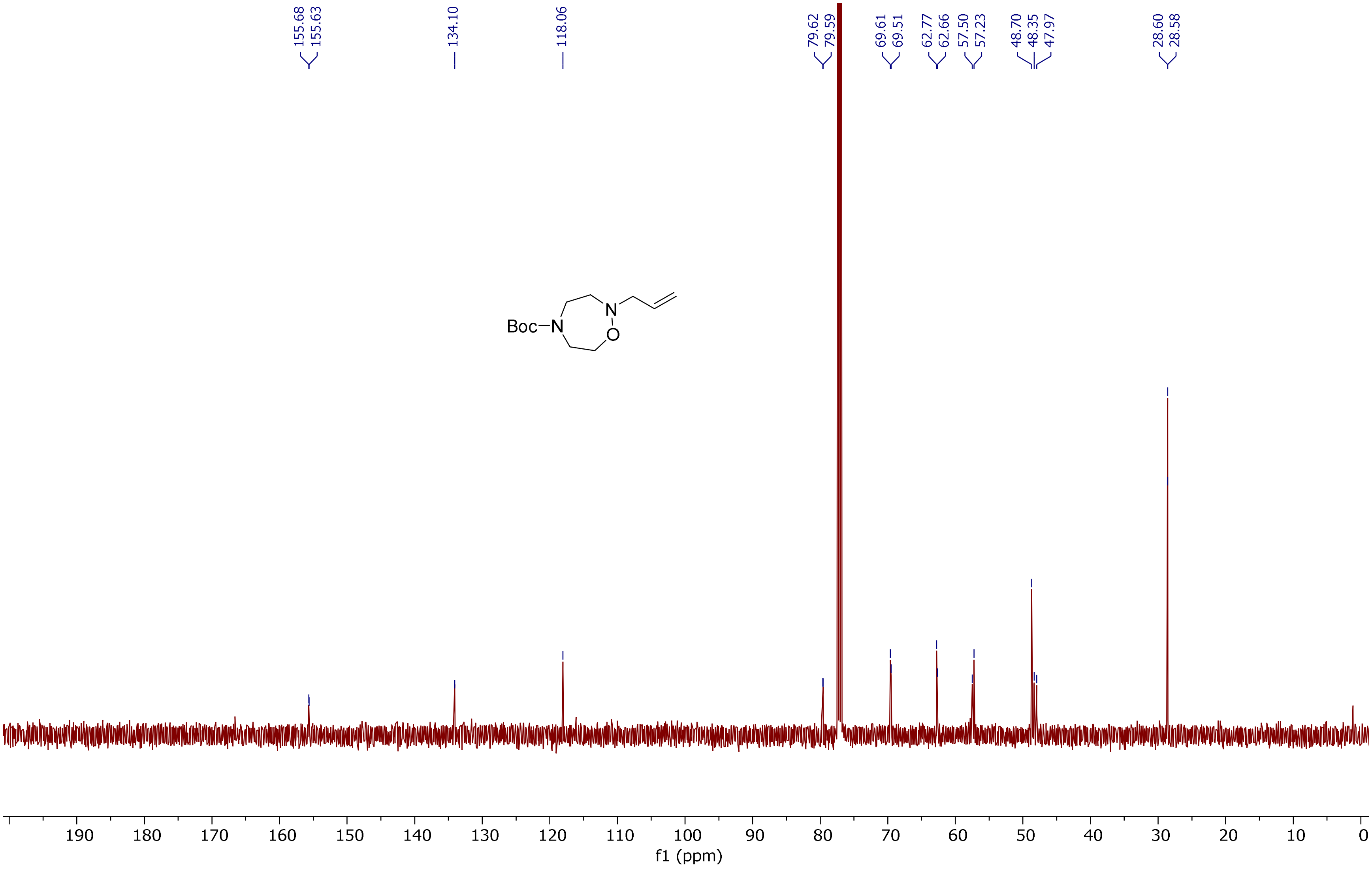

HSQC ($CDCl_3$) spectrum of *tert*-butyl 2-allyl-1,2,5-oxadiazepane-5-carboxylate **(38)**

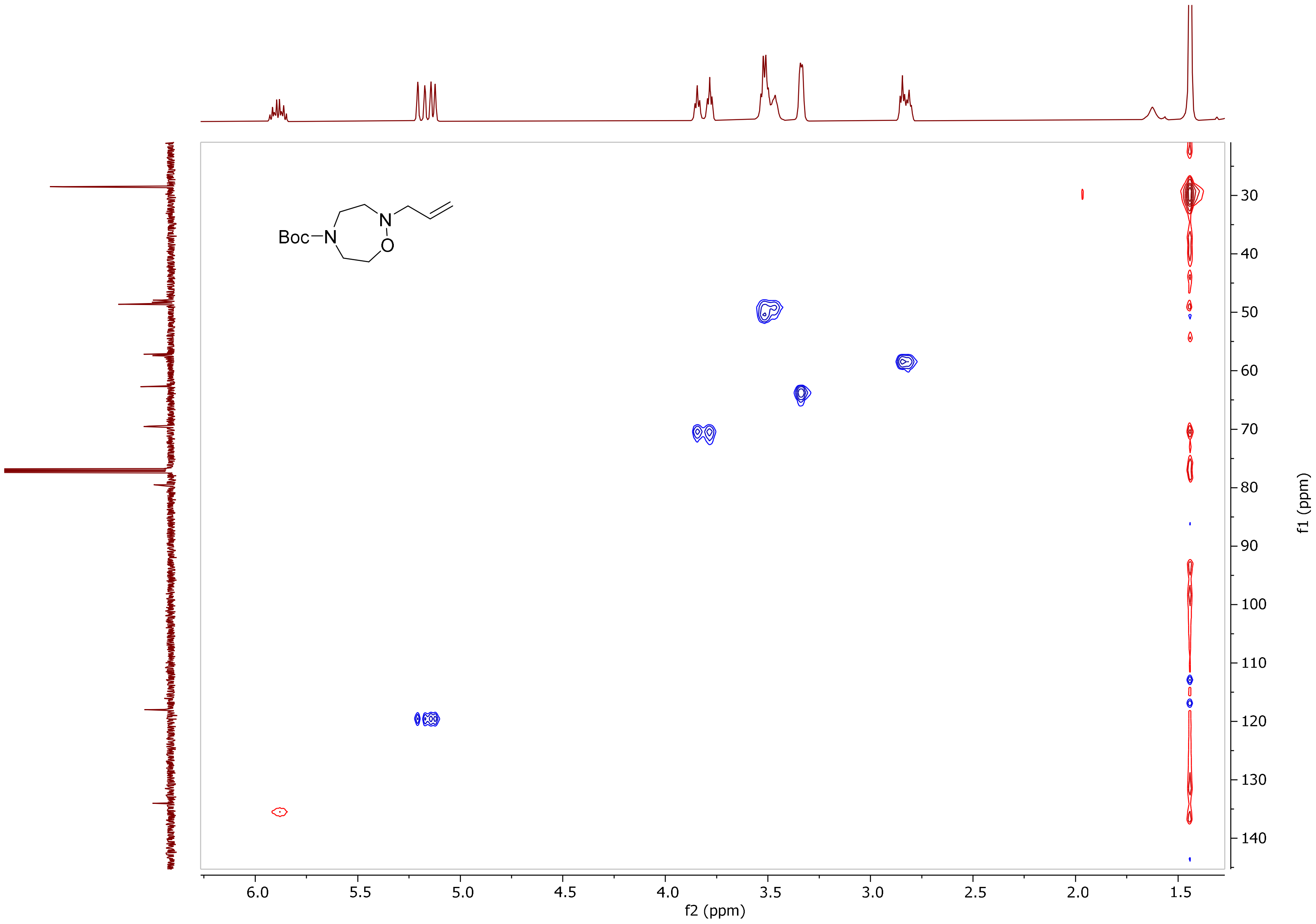

COSY ($CDCl_3$) spectrum of *tert*-butyl 2-allyl-1,2,5-oxadiazepane-5-carboxylate **(38)**

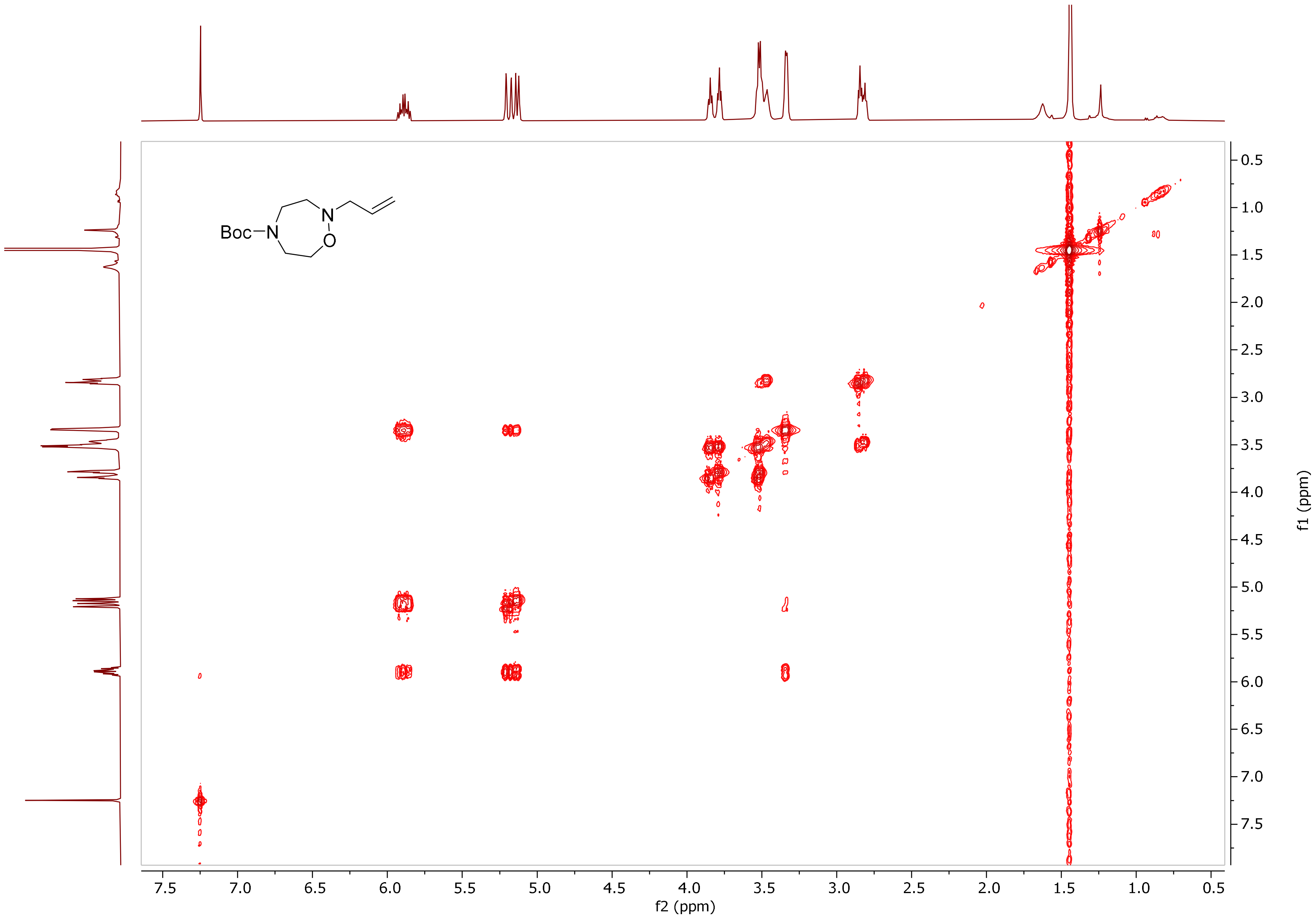

^{1}H NMR (500 MHz, $CDCl_3$) spectrum of *tert*-butyl 2-(2-hydroxyethyl)-1,2,5-oxadiazepane-5-carboxylate **(39)**

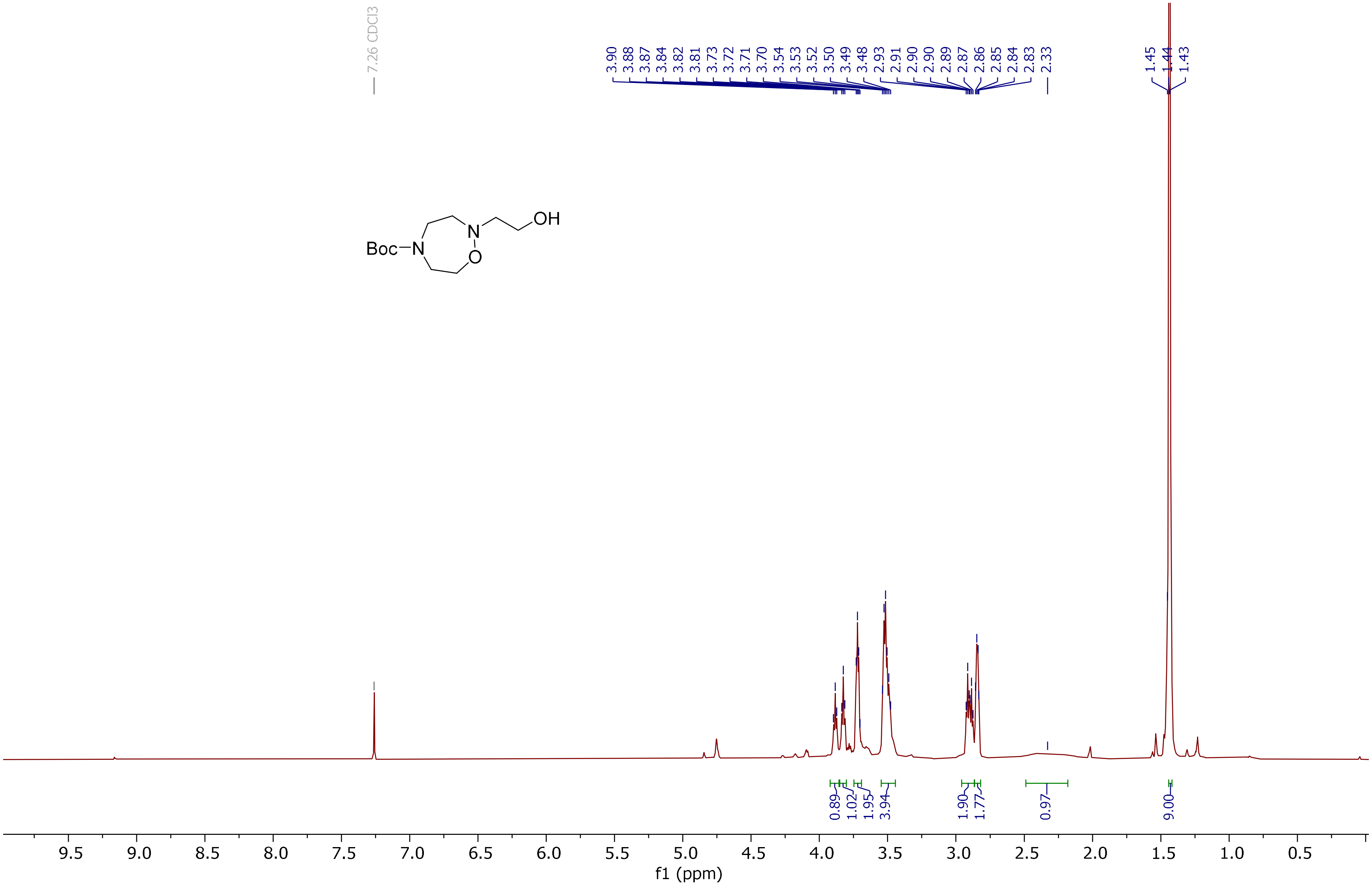

^{13}C NMR (126 MHz, $CDCl_3$) spectrum of *tert*-butyl 2-(2-hydroxyethyl)-1,2,5-oxadiazepane-5-carboxylate **(39)**

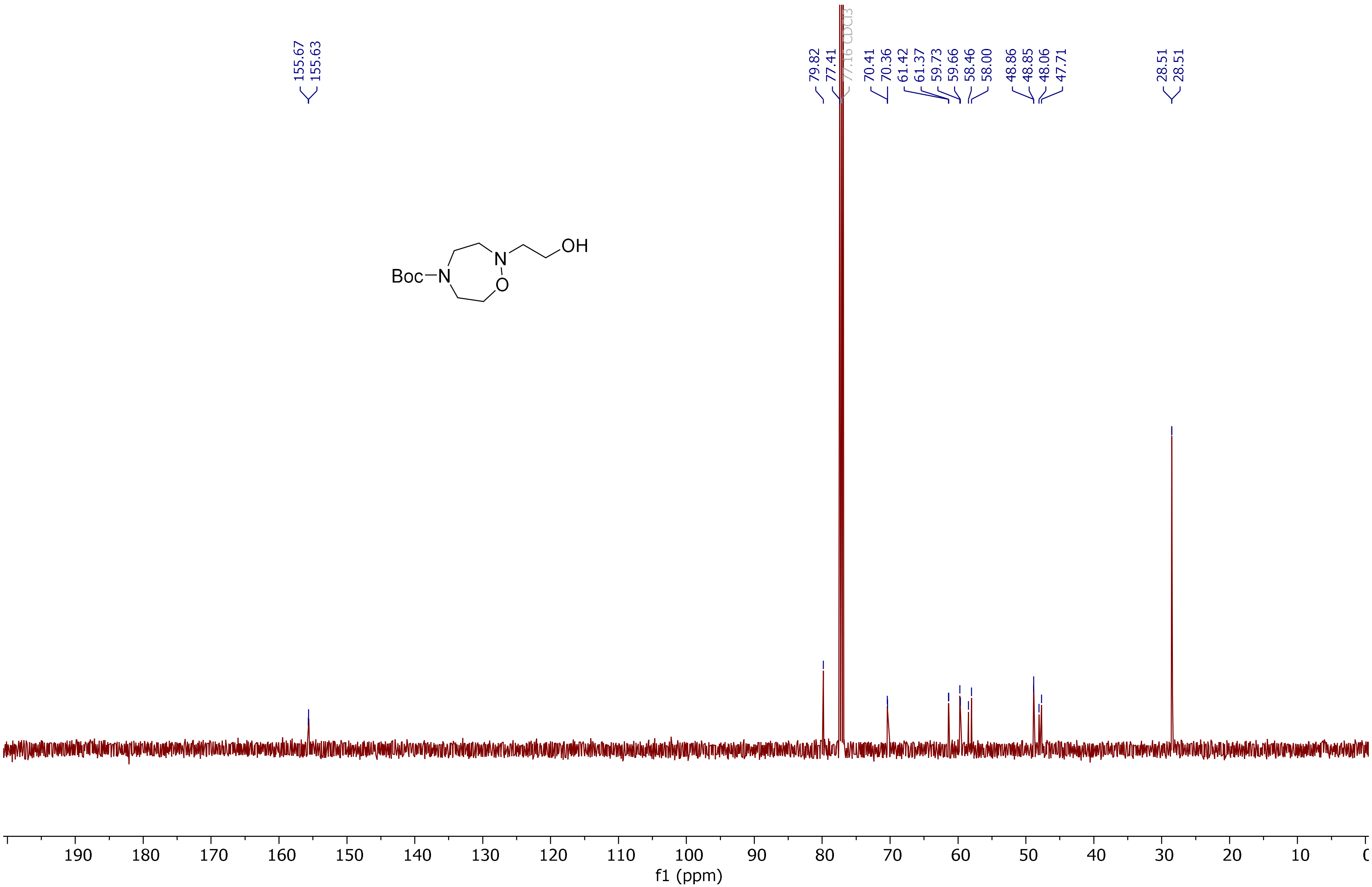

HSQC ($CDCl_3$) spectrum of *tert*-butyl 2-(2-hydroxyethyl)-1,2,5-oxadiazepane-5-carboxylate **(39)**

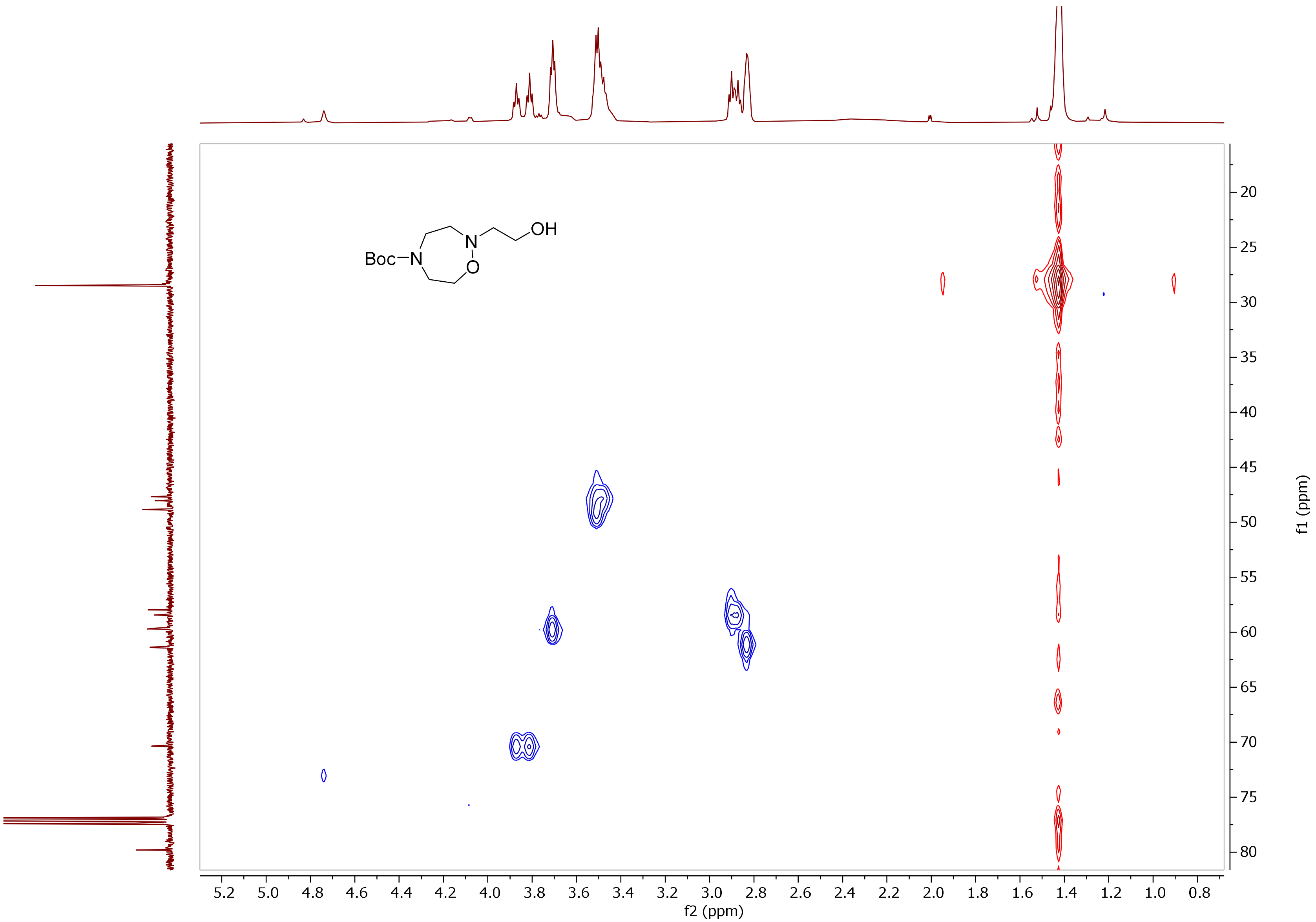

COSY ($CDCl_3$) spectrum of *tert*-butyl 2-(2-hydroxyethyl)-1,2,5-oxadiazepane-5-carboxylate **(39)**

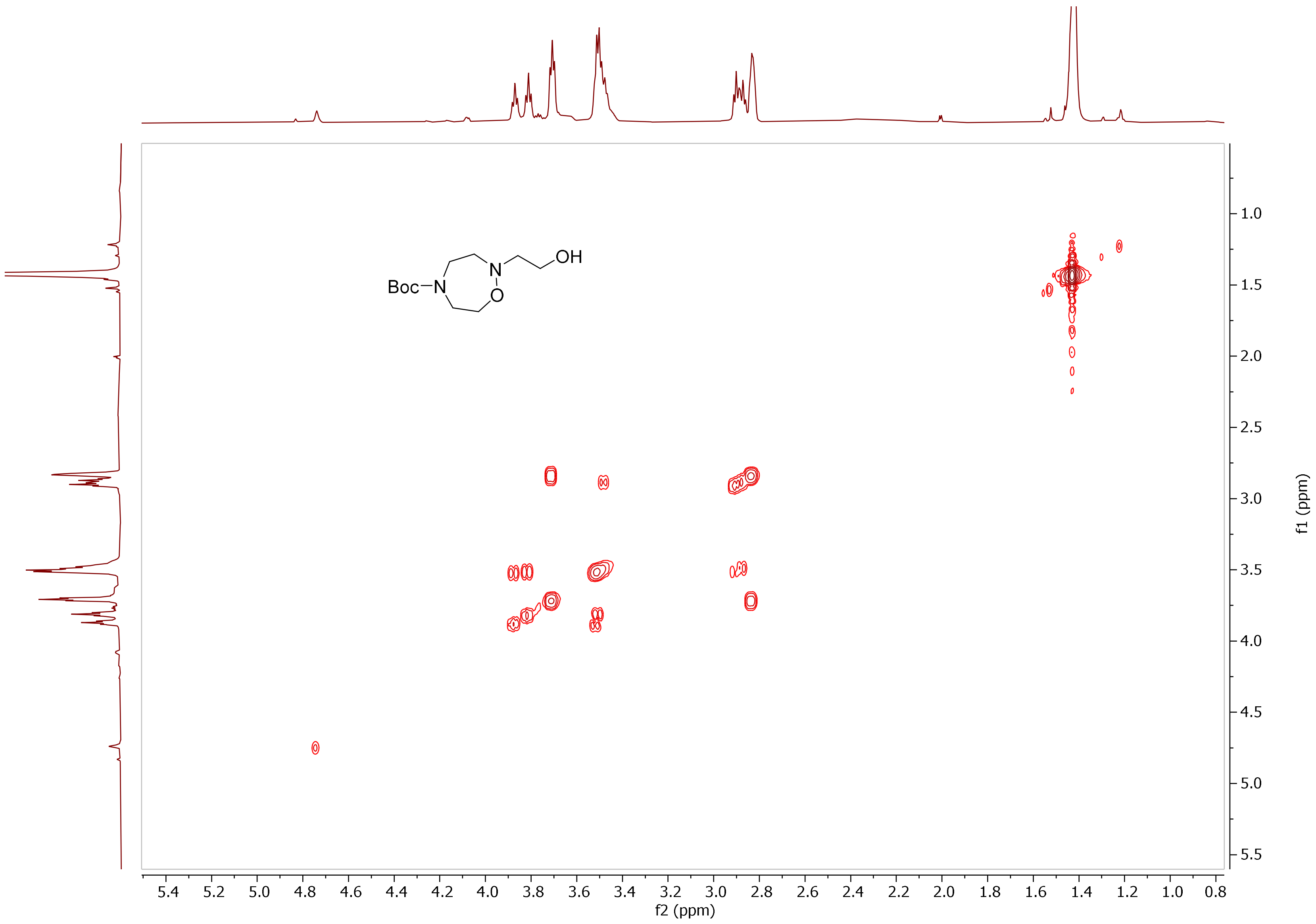

^1H NMR (500 MHz, $CDCl_3$) spectrum of *tert*-butyl 2-(2-((methylsulfonyl)oxy)ethyl)-1,2,5-oxadiazepane-5-carboxylate **(40)**

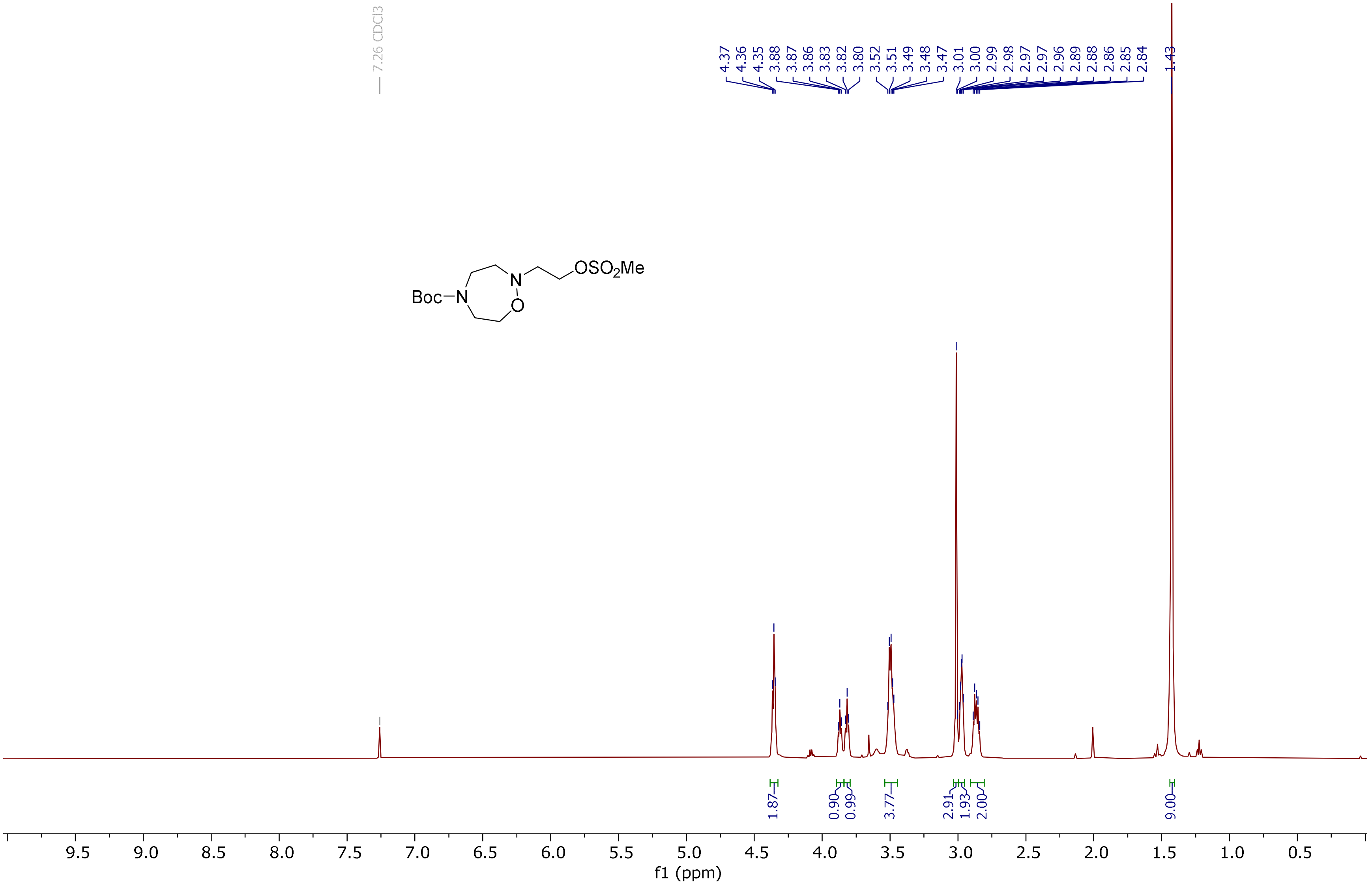

^{13}C NMR (126 MHz, $CDCl_3$) spectrum of *tert*-butyl 2-(2-((methylsulfonyl)oxy)ethyl)-1,2,5-oxadiazepane-5-carboxylate **(40)**

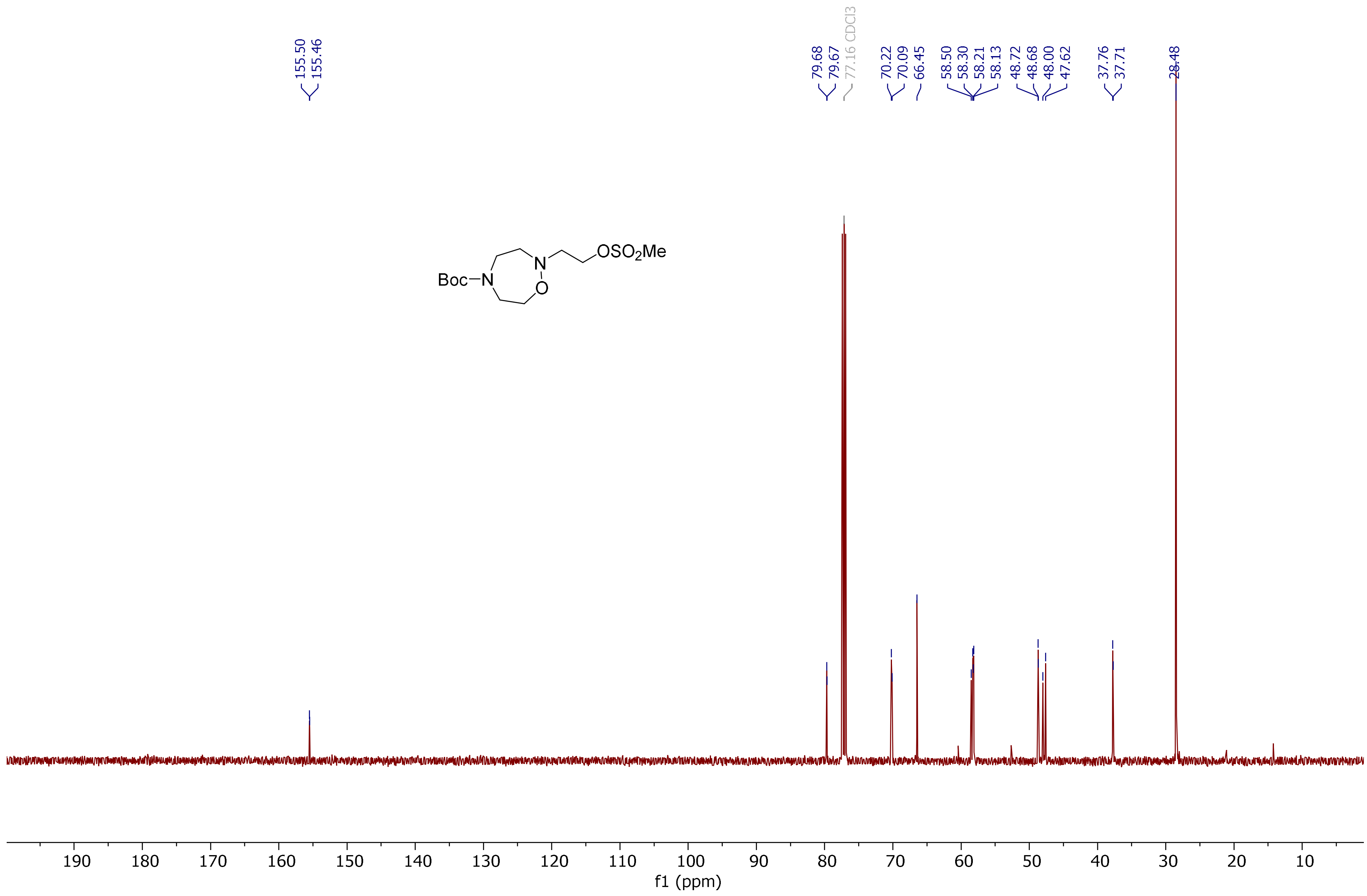

HSQC ($CDCl_3$) spectrum of *tert*-butyl 2-(2-((methylsulfonyl)oxy)ethyl)-1,2,5-oxadiazepane-5-carboxylate **(40)**

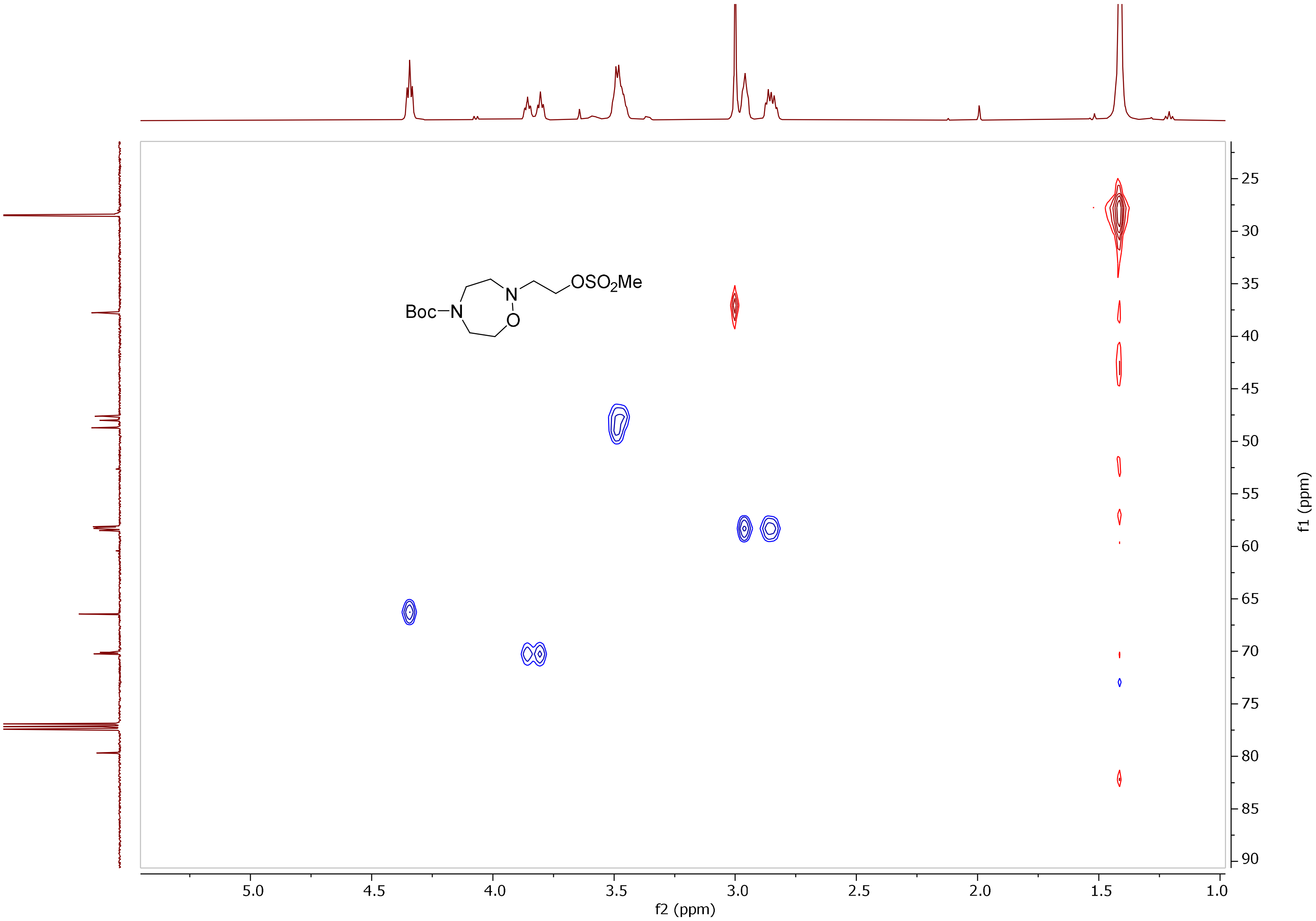

COSY ($CDCl_3$) spectrum of *tert*-butyl 2-(2-((methylsulfonyl)oxy)ethyl)-1,2,5-oxadiazepane-5-carboxylate **(40)**

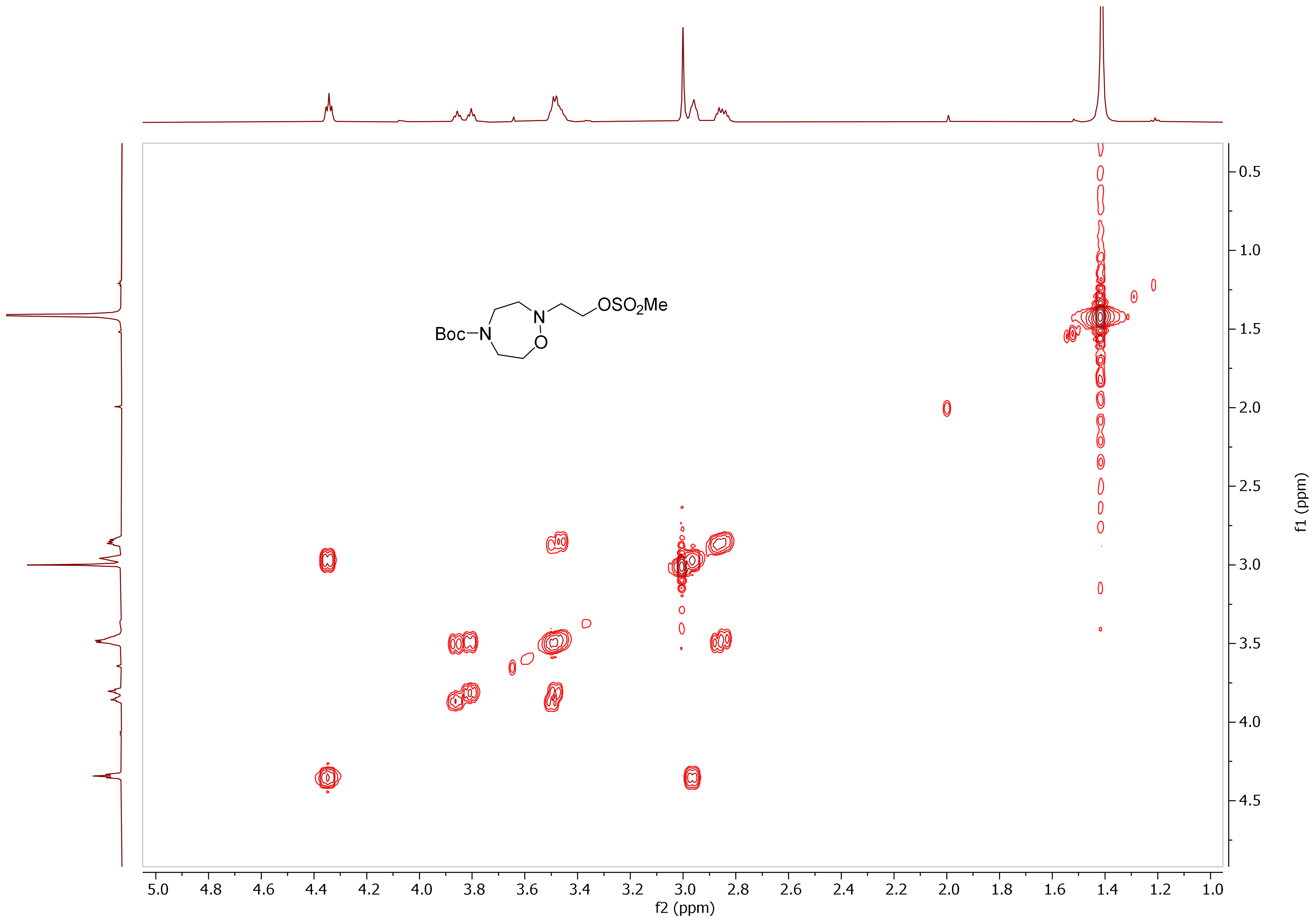

^{1}H NMR (500 MHz, $CDCl_3$) spectrum of *tert*-butyl 2-(2-azidoethyl)-1,2,5-oxadiazepane-5-carboxylate **(41)**

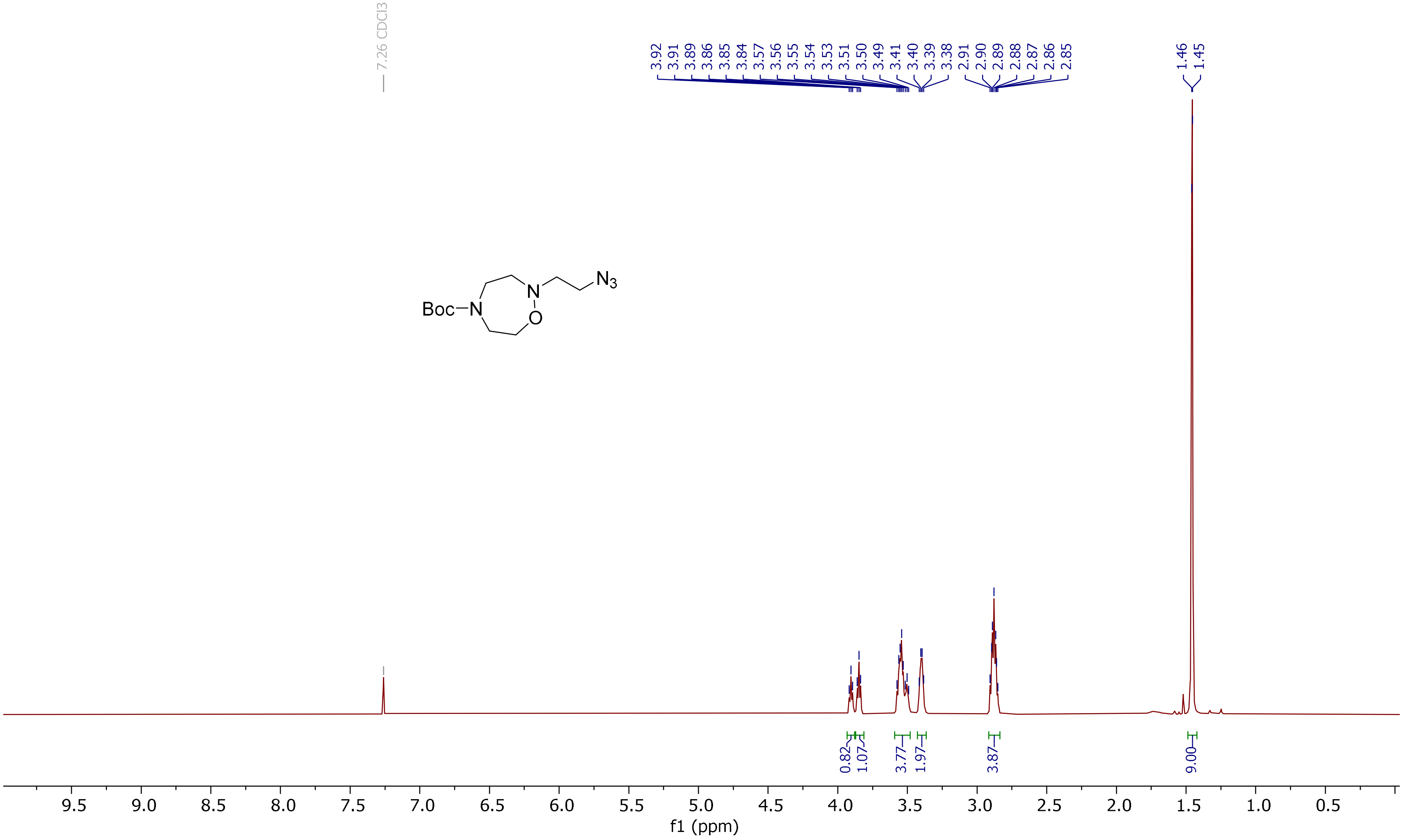

^{13}C NMR (126 MHz, $CDCl_3$) spectrum of *tert*-butyl 2-(2-azidoethyl)-1,2,5-oxadiazepane-5-carboxylate **(41)**

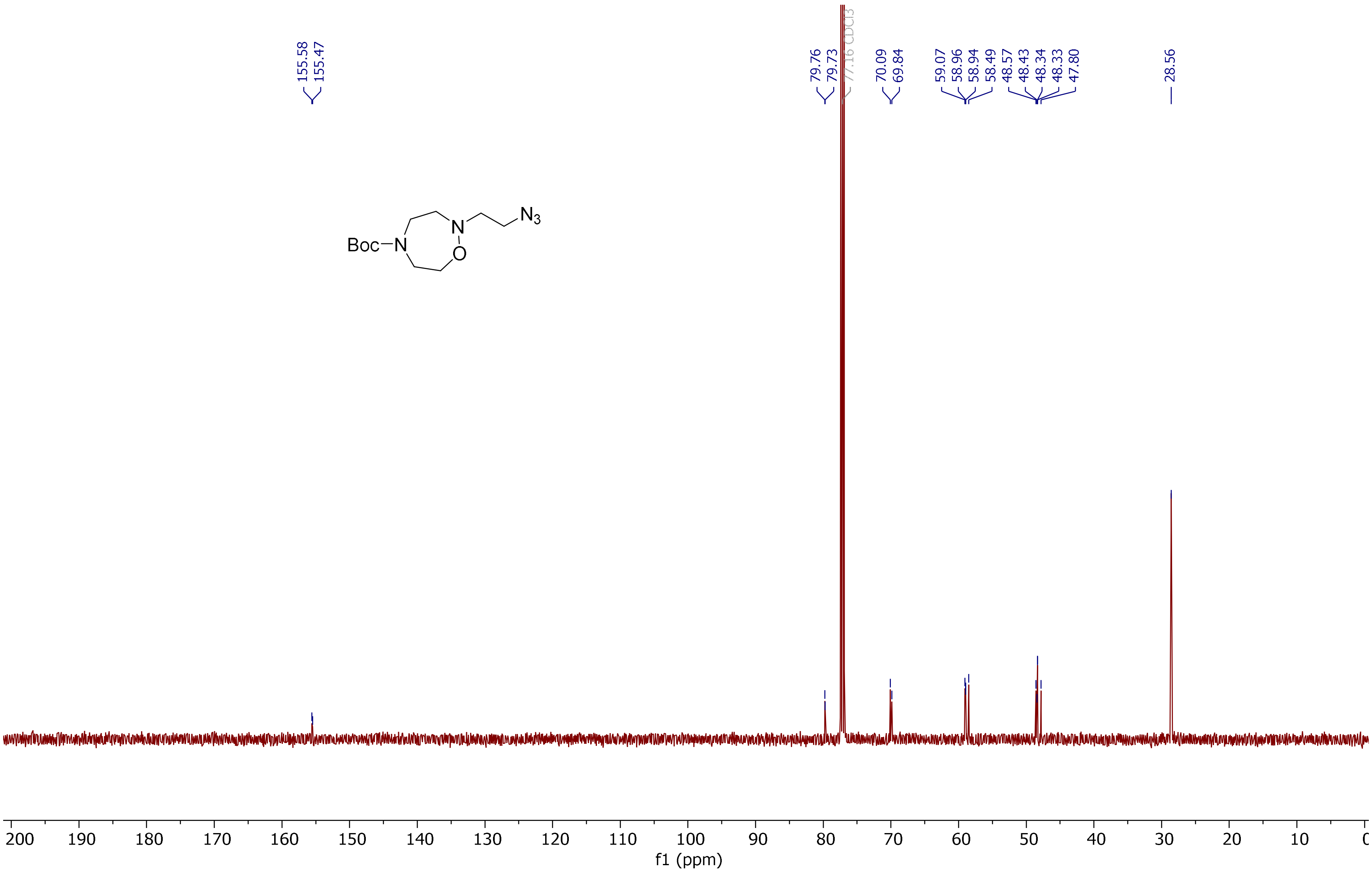

HSQC ($CDCl_3$) spectrum of *tert*-butyl 2-(2-azidoethyl)-1,2,5-oxadiazepane-5-carboxylate **(41)**

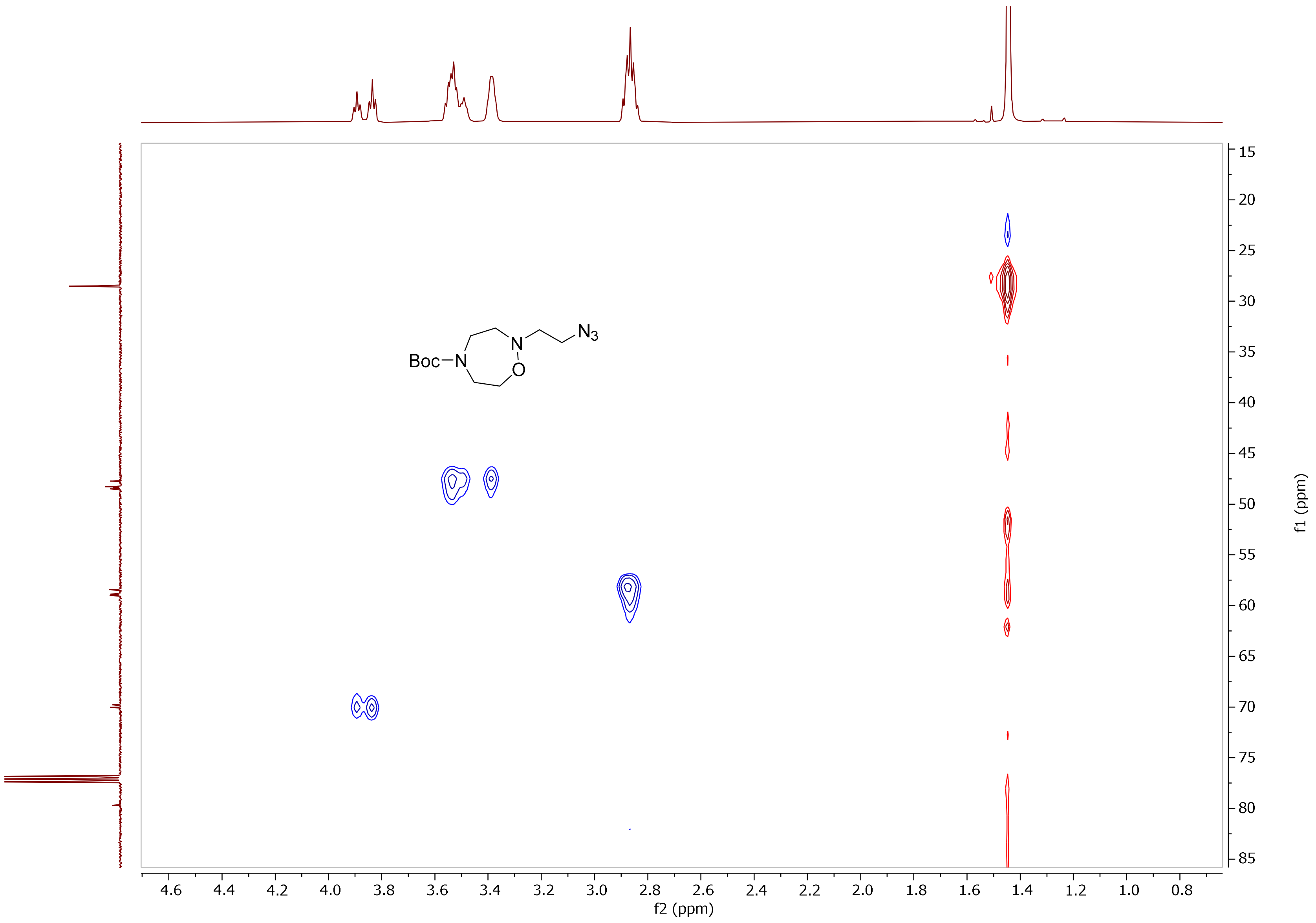

COSY ($CDCl_3$) spectrum of *tert*-butyl 2-(2-azidoethyl)-1,2,5-oxadiazepane-5-carboxylate **(41)**

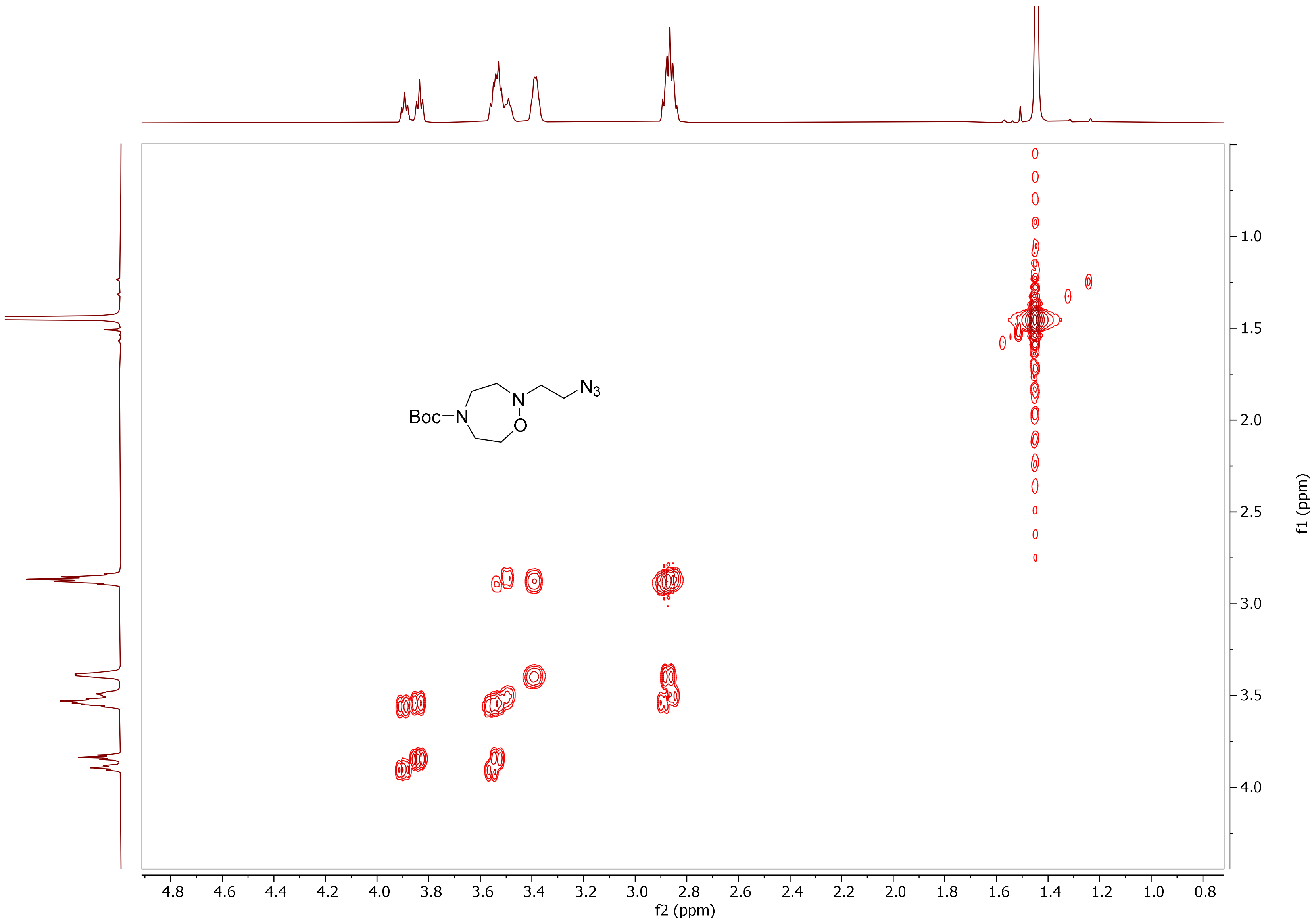

^{1}H NMR (500 MHz, $CDCl_3$) spectrum of 2-(2-azidoethyl)-5-methyl-1,2,5-oxadiazepane **(42)**

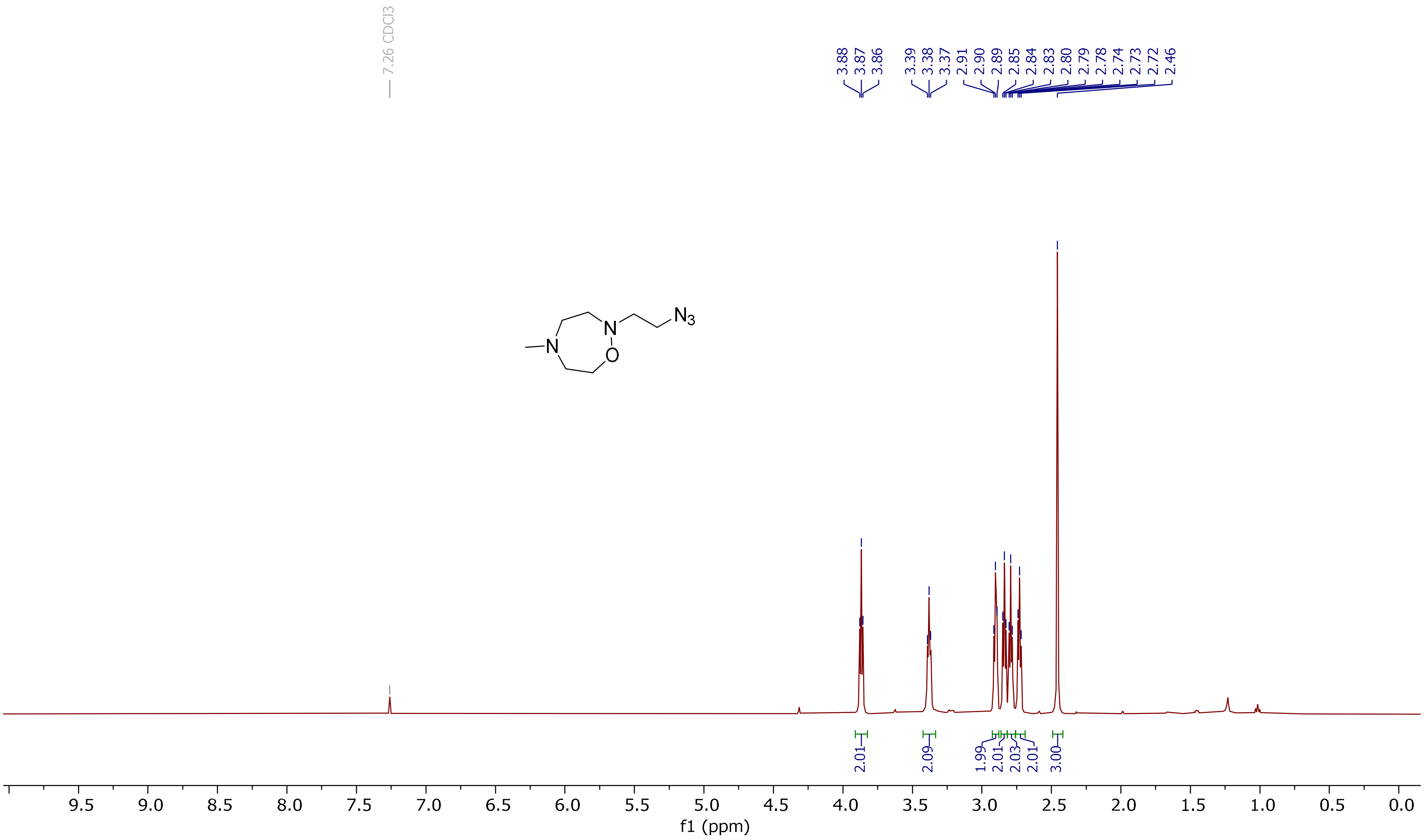

^{13}C NMR (126 MHz, $CDCl_3$) spectrum of 2-(2-azidoethyl)-5-methyl-1,2,5-oxadiazepane **(42)**

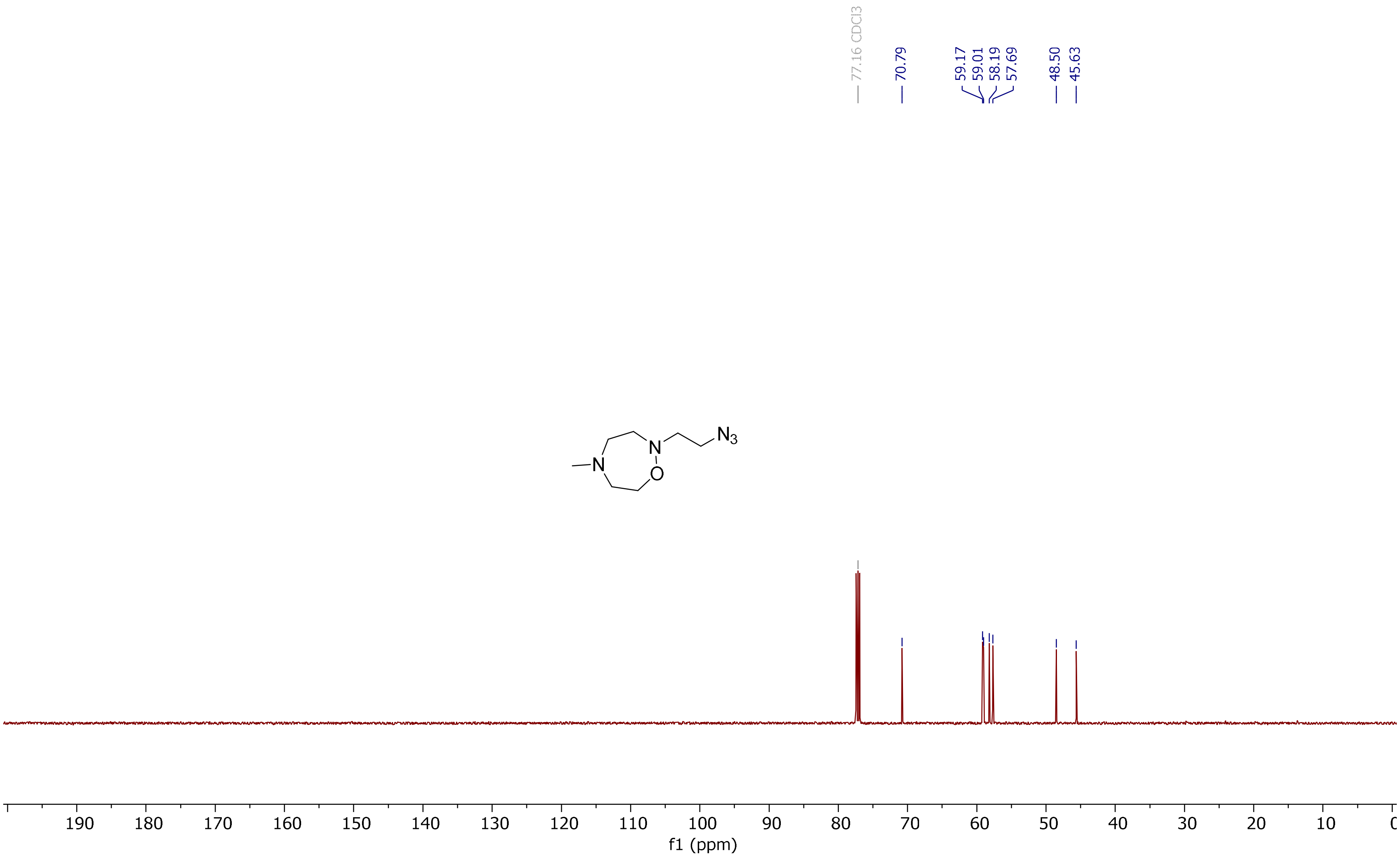

HSQC ($CDCl_3$) spectrum of 2-(2-azidoethyl)-5-methyl-1,2,5-oxadiazepane **(42)**

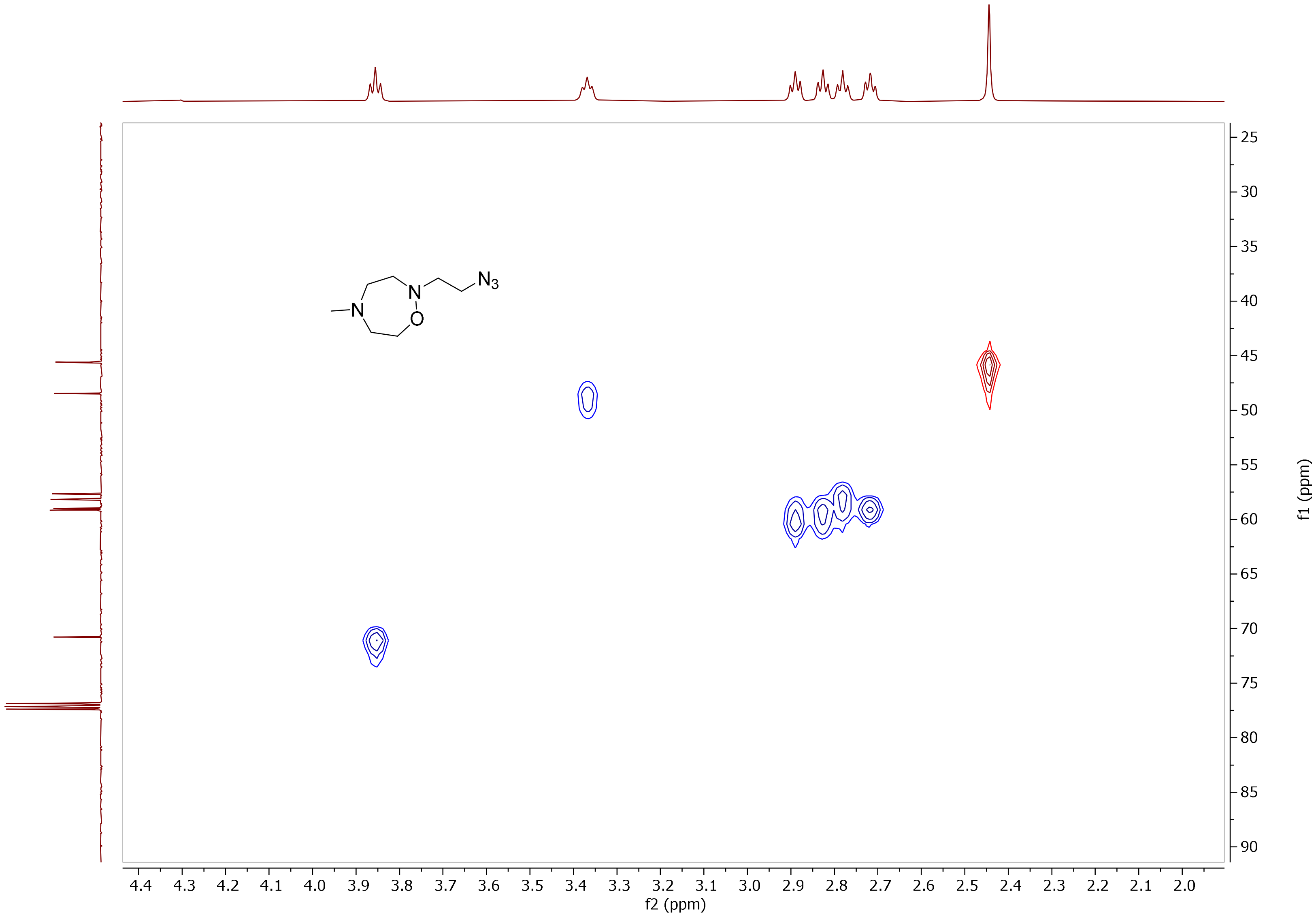

COSY ($CDCl_3$) spectrum of 2-(2-azidoethyl)-5-methyl-1,2,5-oxadiazepane **(42)**

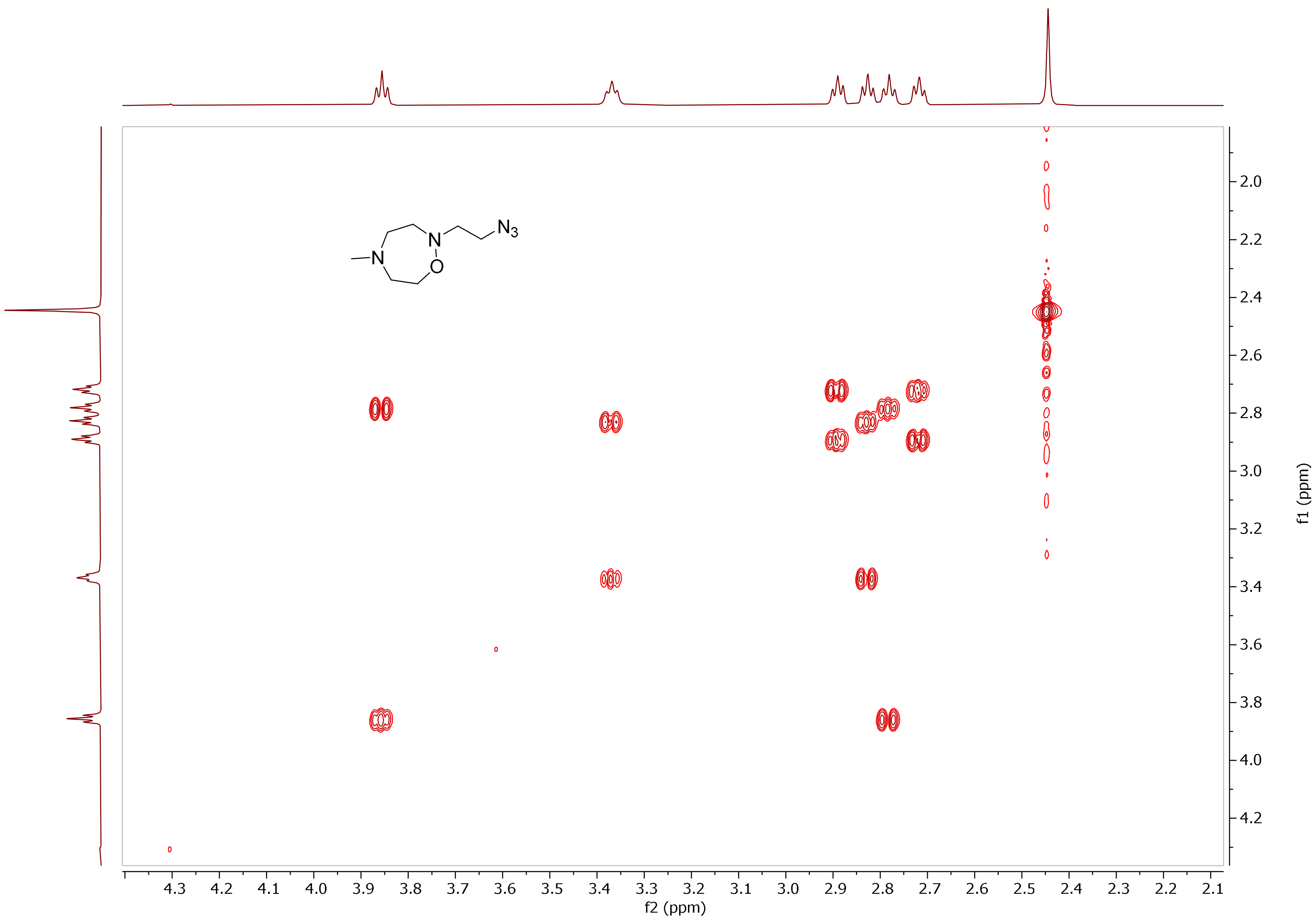

^{1}H NMR (500 MHz, $CDCl_3$) spectrum of 2-(5-methyl-1,2,5-oxadiazepan-2-yl)ethan-1-amine **(43)**

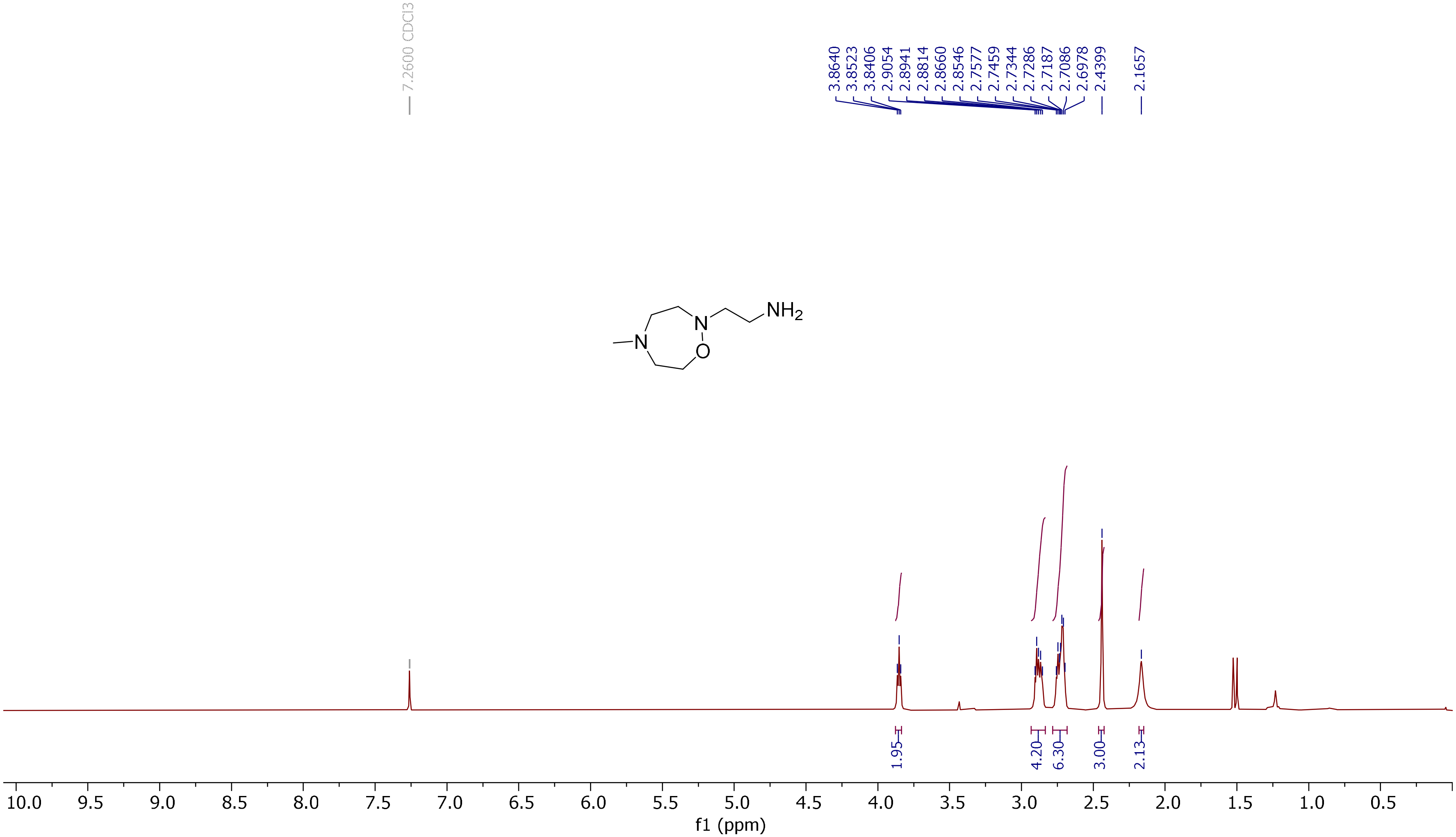

^{13}C NMR (126 MHz, $CDCl_3$) spectrum of 2-(5-methyl-1,2,5-oxadiazepan-2-yl)ethan-1-amine **(43)**

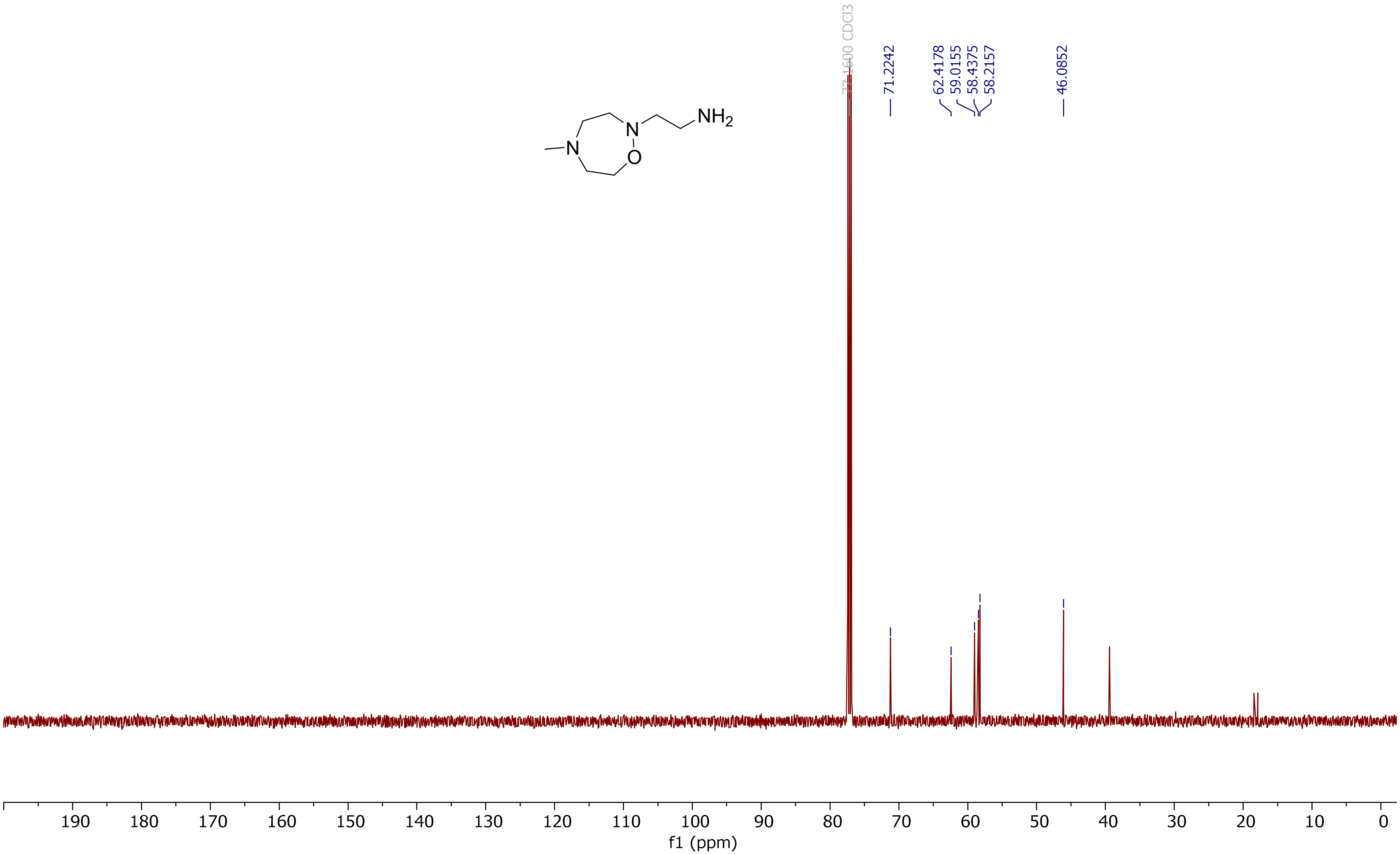

HSQC ($CDCl_3$) spectrum of 2-(5-methyl-1,2,5-oxadiazepan-2-yl)ethan-1-amine **(43)**

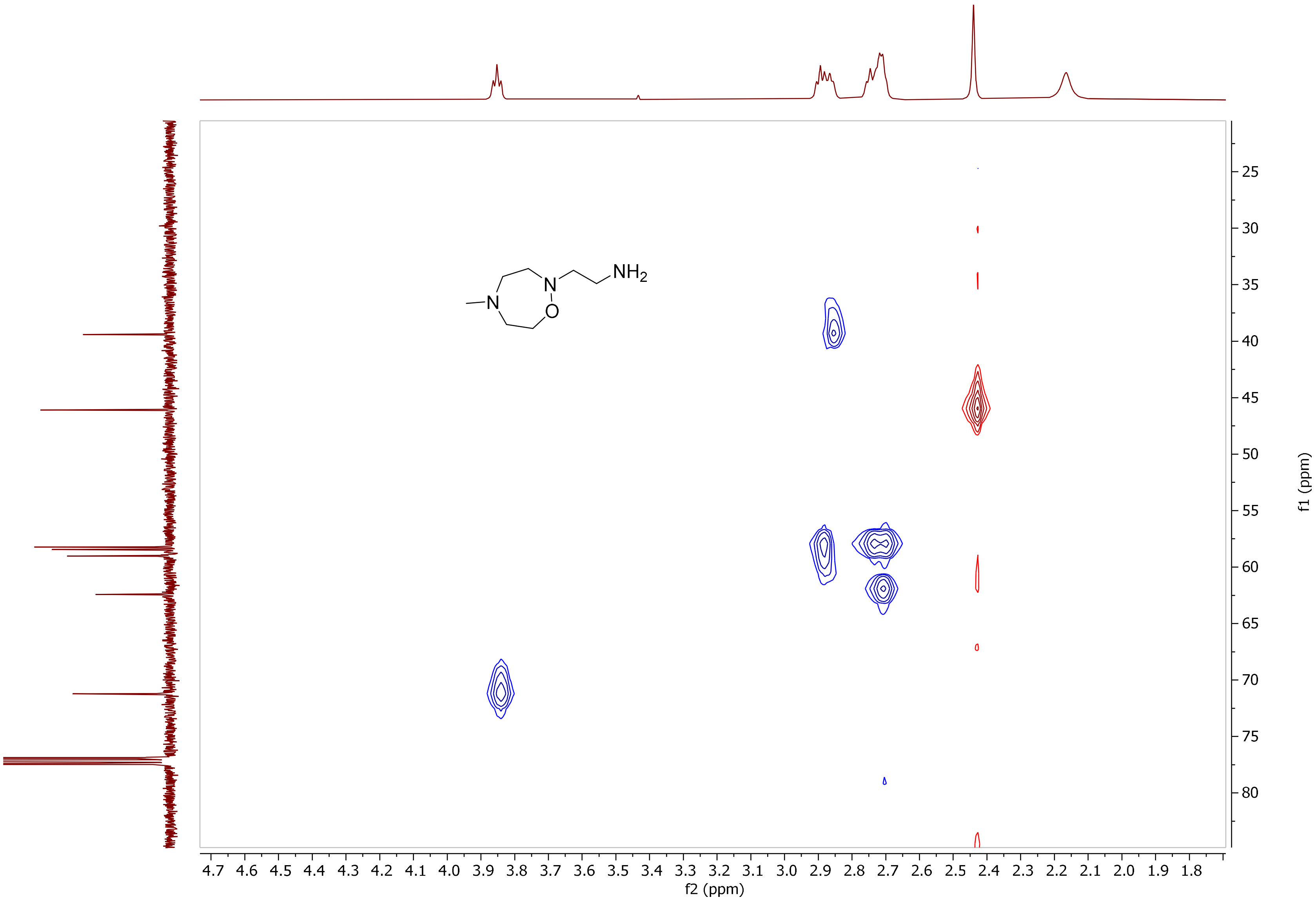

COSY ($CDCl_3$) spectrum of 2-(5-methyl-1,2,5-oxadiazepan-2-yl)ethan-1-amine **(43)**

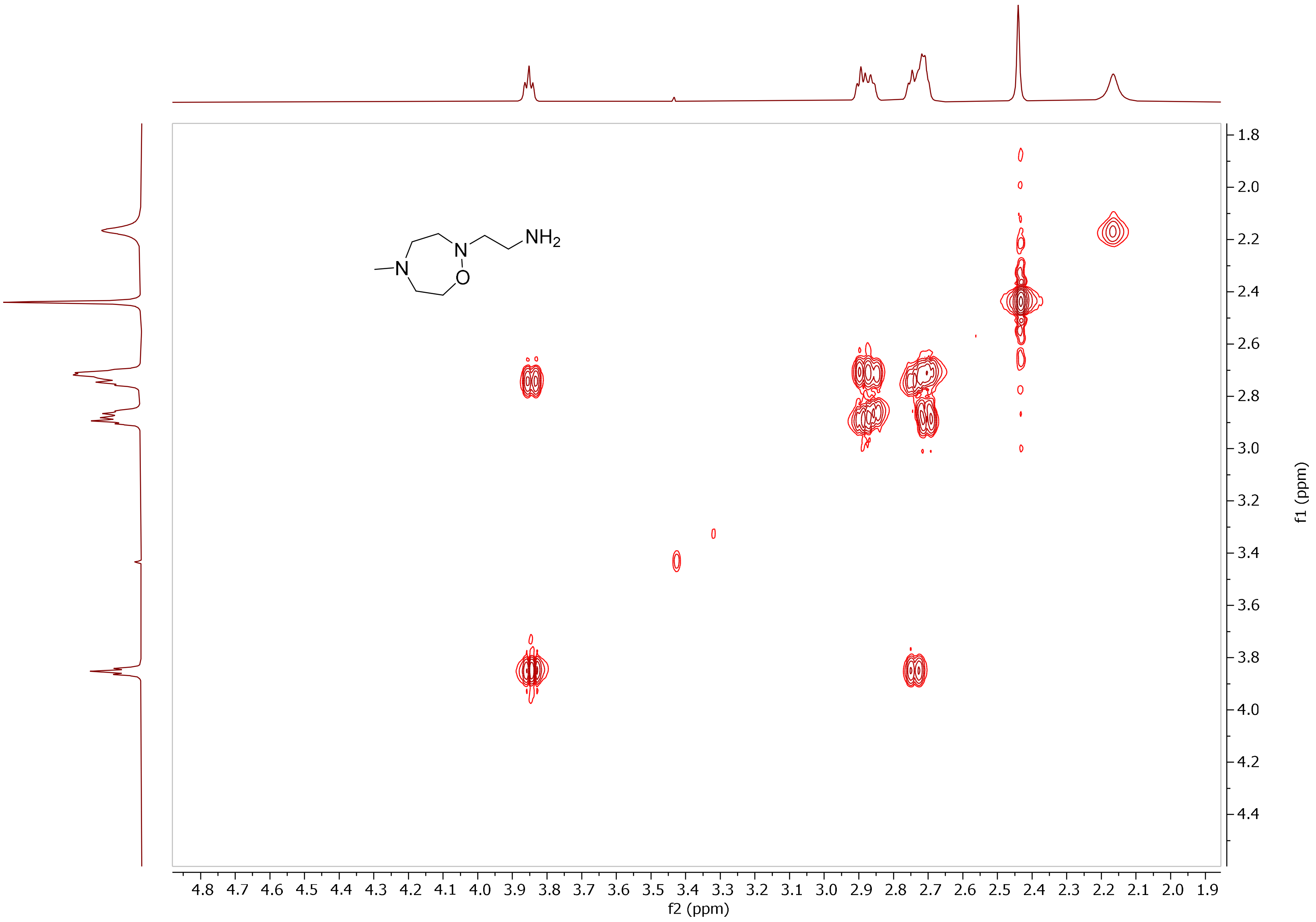

^{1}H NMR (500 MHz, $CDCl_3$) spectrum of 6-(2-chloro-4-(6-methylpyrazin-2-yl)phenyl)-8-ethyl-2-((2-(5-methyl-1,2,5-oxadiazepan-2-yl)ethyl)amino)pyrido[2,3-*d*]pyrimidin-7(8*H*)-one **(12)**

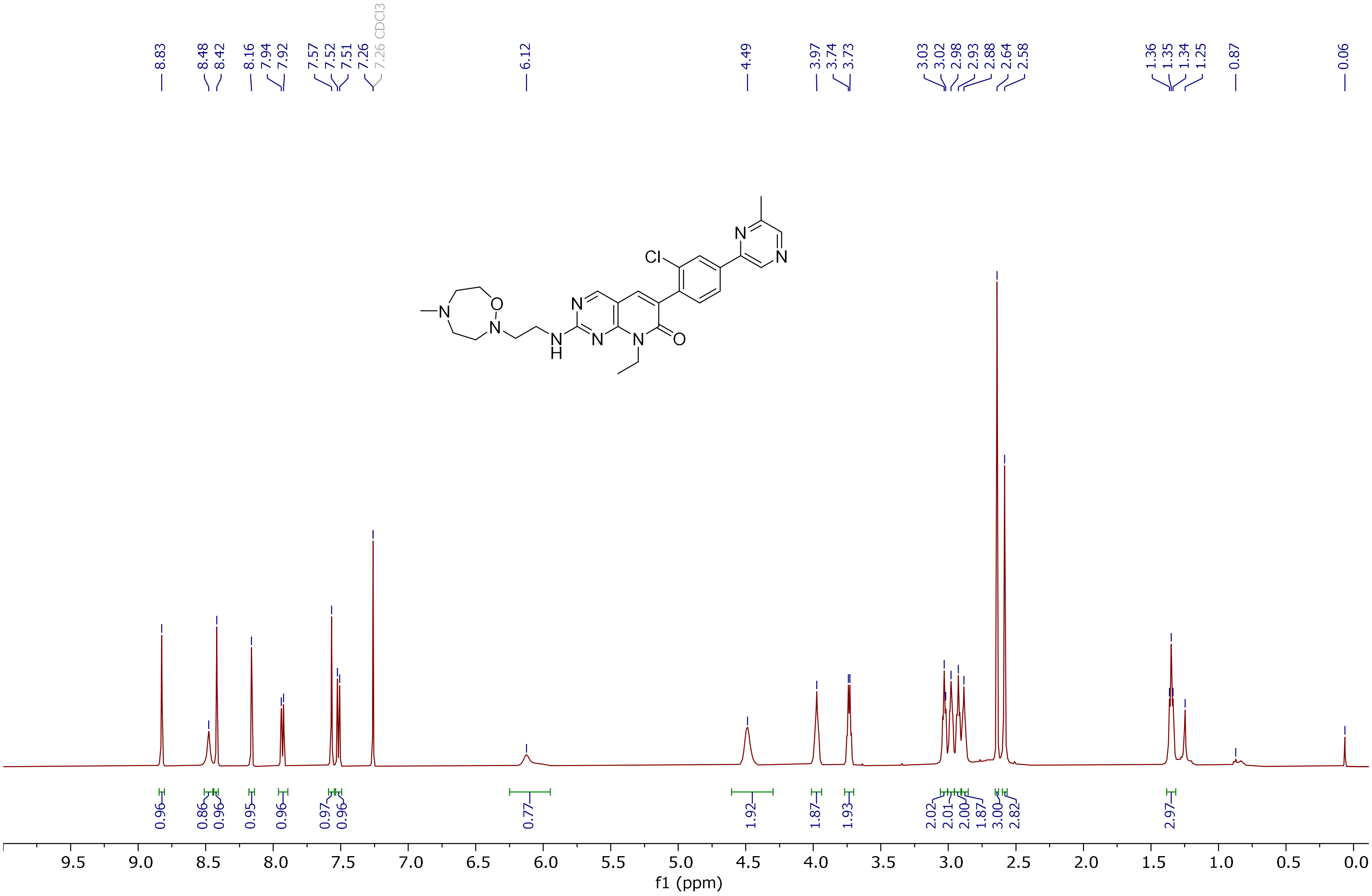

^{13}C NMR (226 MHz, $CDCl_3$) spectrum of 6-(2-chloro-4-(6-methylpyrazin-2-yl)phenyl)-8-ethyl-2-((2-(5-methyl-1,2,5-oxadiazepan-2-yl)ethyl)amino)pyrido[2,3-*d*]pyrimidin-7(8*H*)-one **(12)**

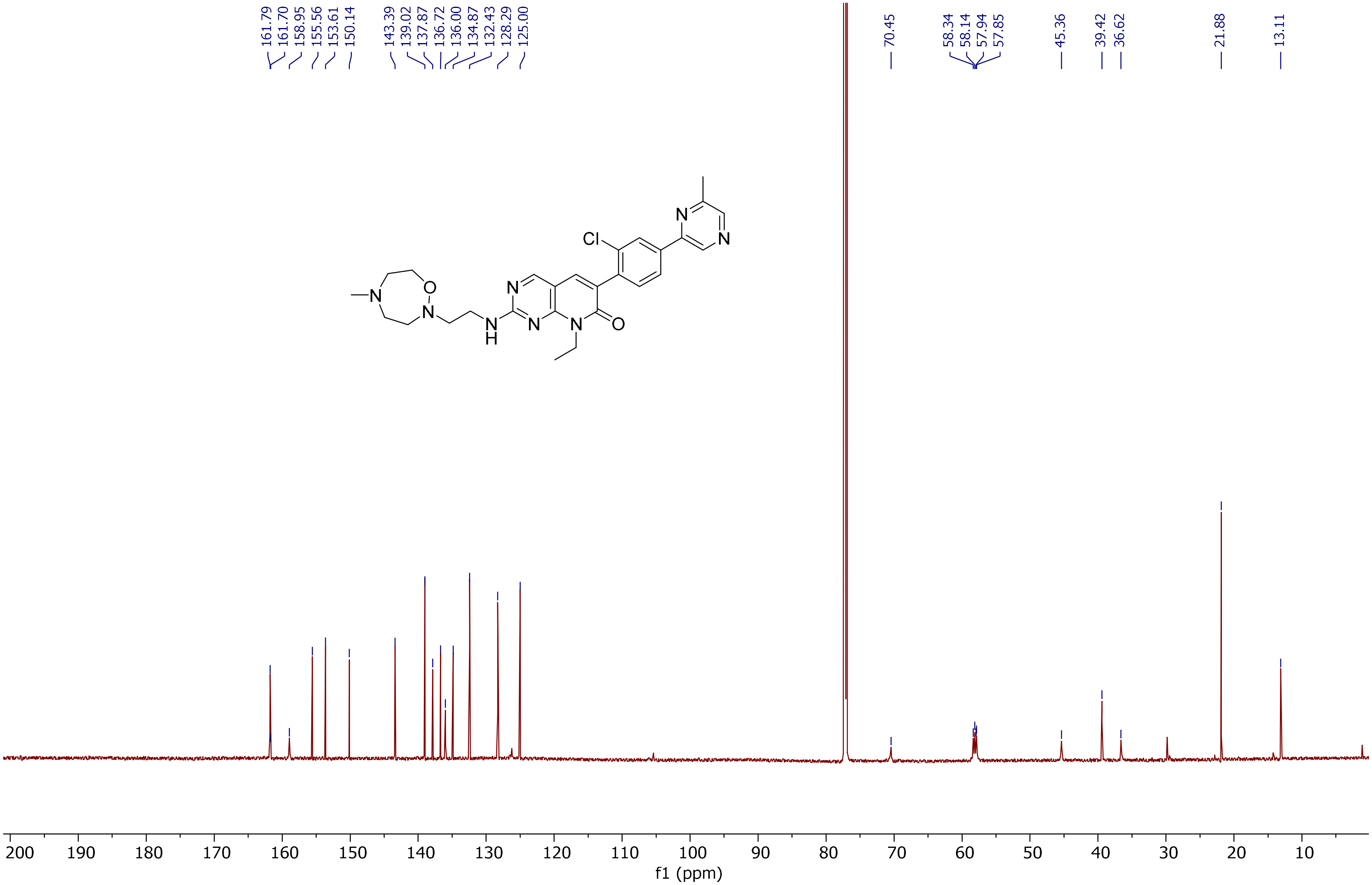

HSQC ($CDCl_3$) spectrum of 6-(2-chloro-4-(6-methylpyrazin-2-yl)phenyl)-8-ethyl-2-((2-(5-methyl-1,2,5-oxadiazepan-2-yl)ethyl)amino)pyrido[2,3-*d*]pyrimidin-7(8*H*)-one **(12)**

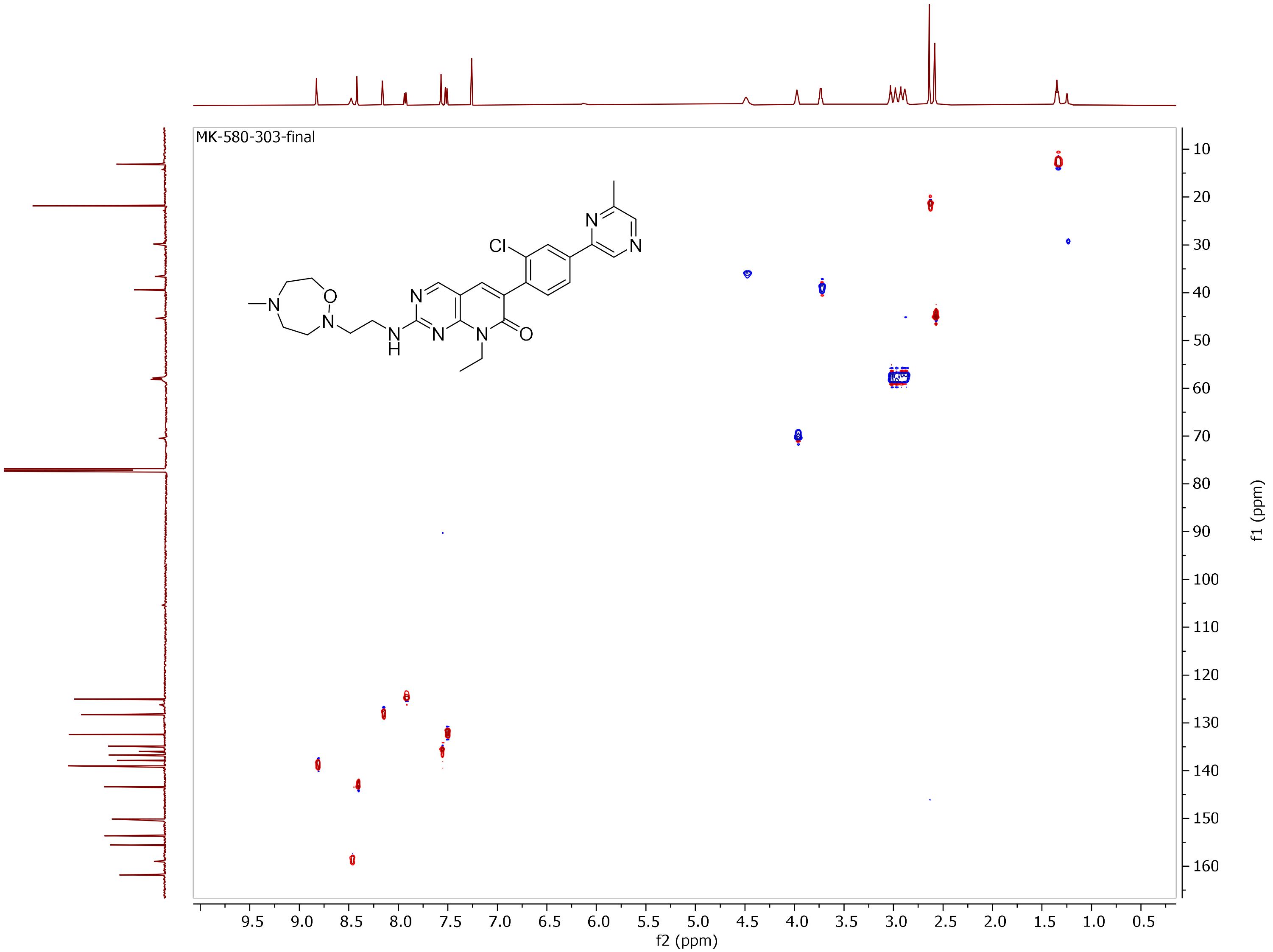

COSY ($CDCl_3$) spectrum of 6-(2-chloro-4-(6-methylpyrazin-2-yl)phenyl)-8-ethyl-2-((2-(5-methyl-1,2,5-oxadiazepan-2-yl)ethyl)amino)pyrido[2,3-*d*]pyrimidin-7(8*H*)-one **(12)**

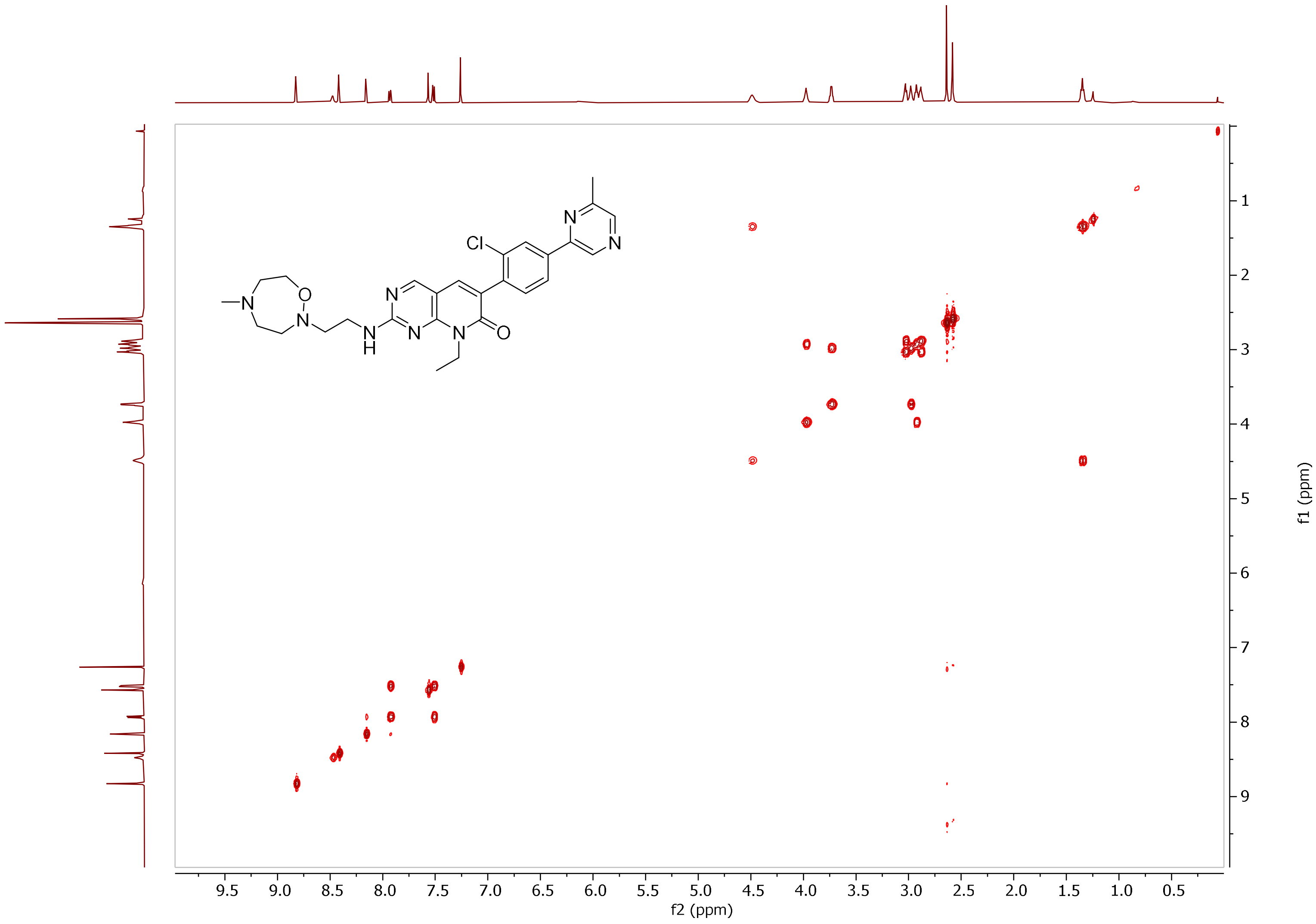

^{1}H NMR Spectrum (500 MHz, C_6D_6) of 2-benzyl 5-(*tert*-butyl) 1,2,5-oxadiazepane-2,5-dicarboxylate **(44)**

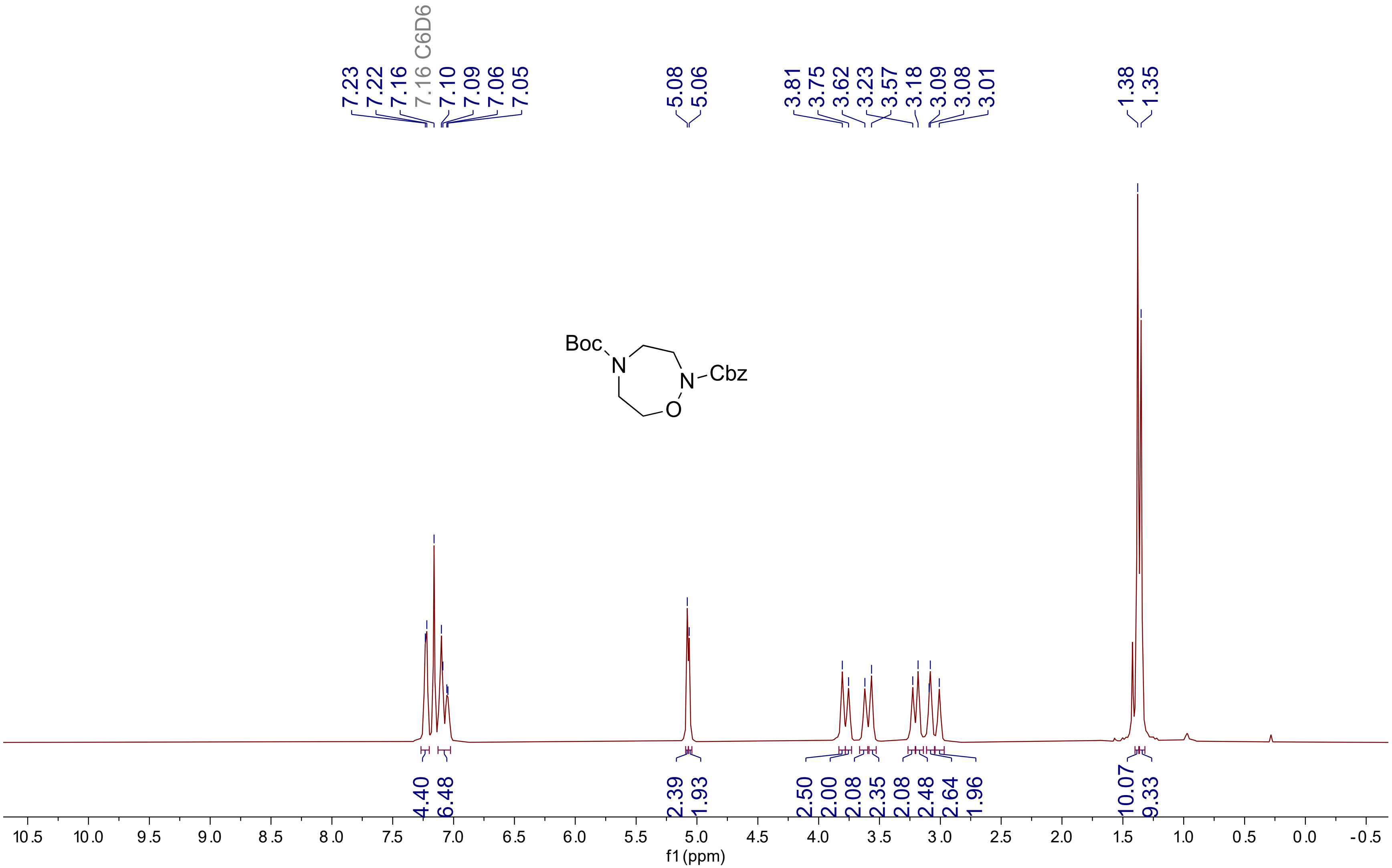

^{13}C NMR spectrum (126 MHz, C_6D_6) of 2-benzyl 5-(*tert*-butyl) 1,2,5-oxadiazepane-2,5-dicarboxylate **(44)**

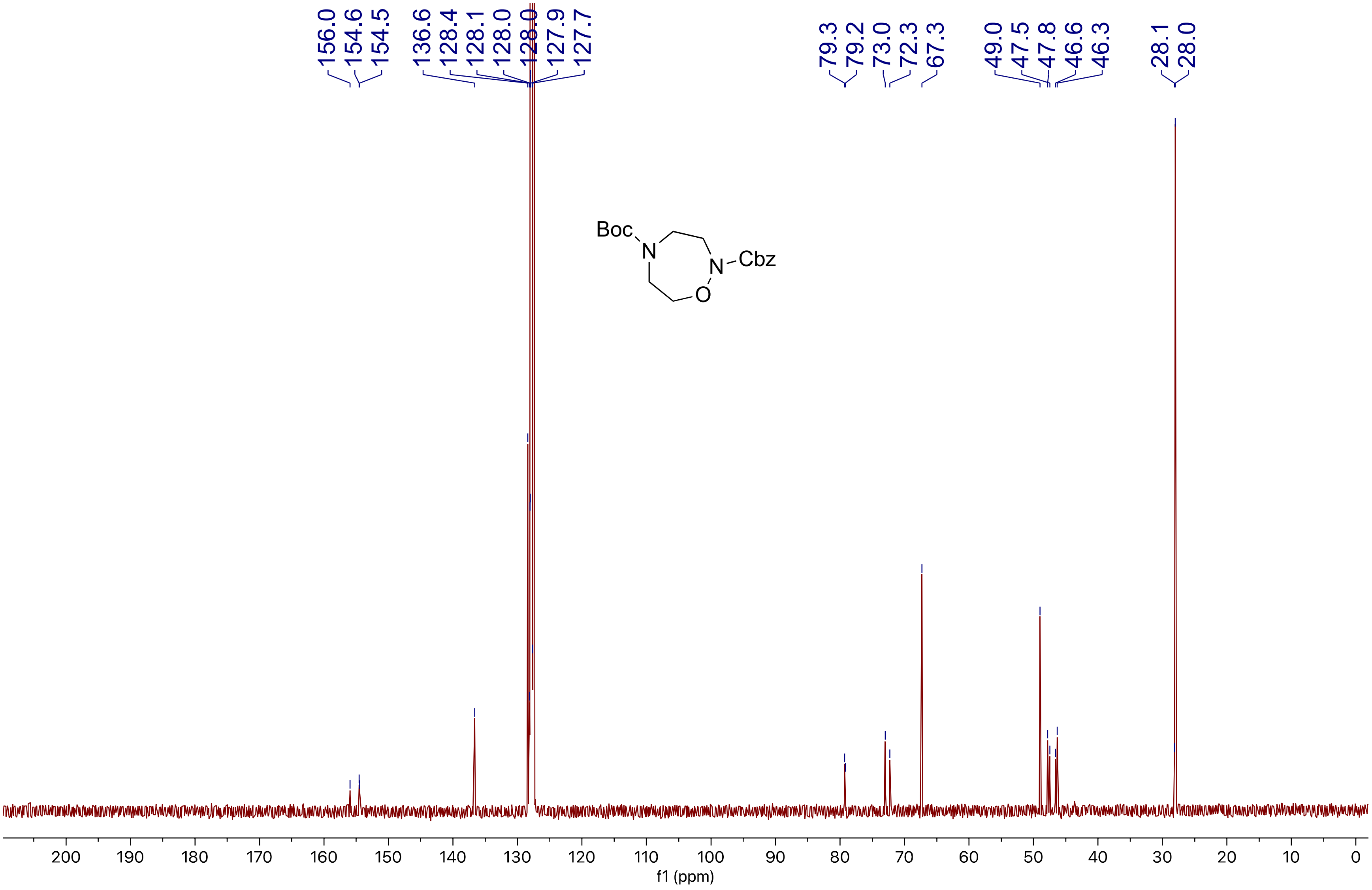

HSQC (C_6D_6) spectrum of 2-benzyl 5-(*tert*-butyl) 1,2,5-oxadiazepane-2,5-dicarboxylate **(44)**

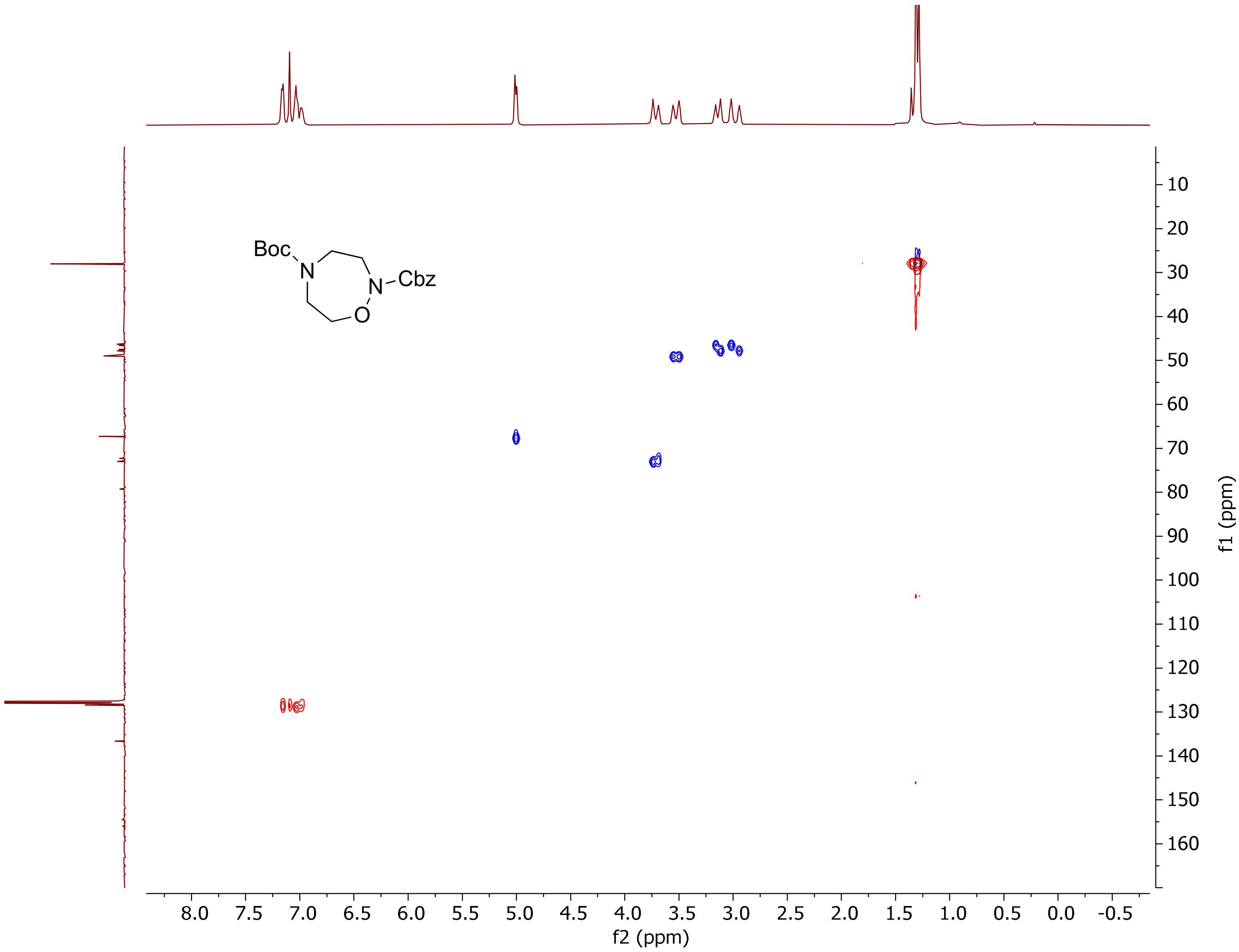

COSY(C_6D_6) spectrum of 2-benzyl 5-(*tert*-butyl) 1,2,5-oxadiazepane-2,5-dicarboxylate **(44)**

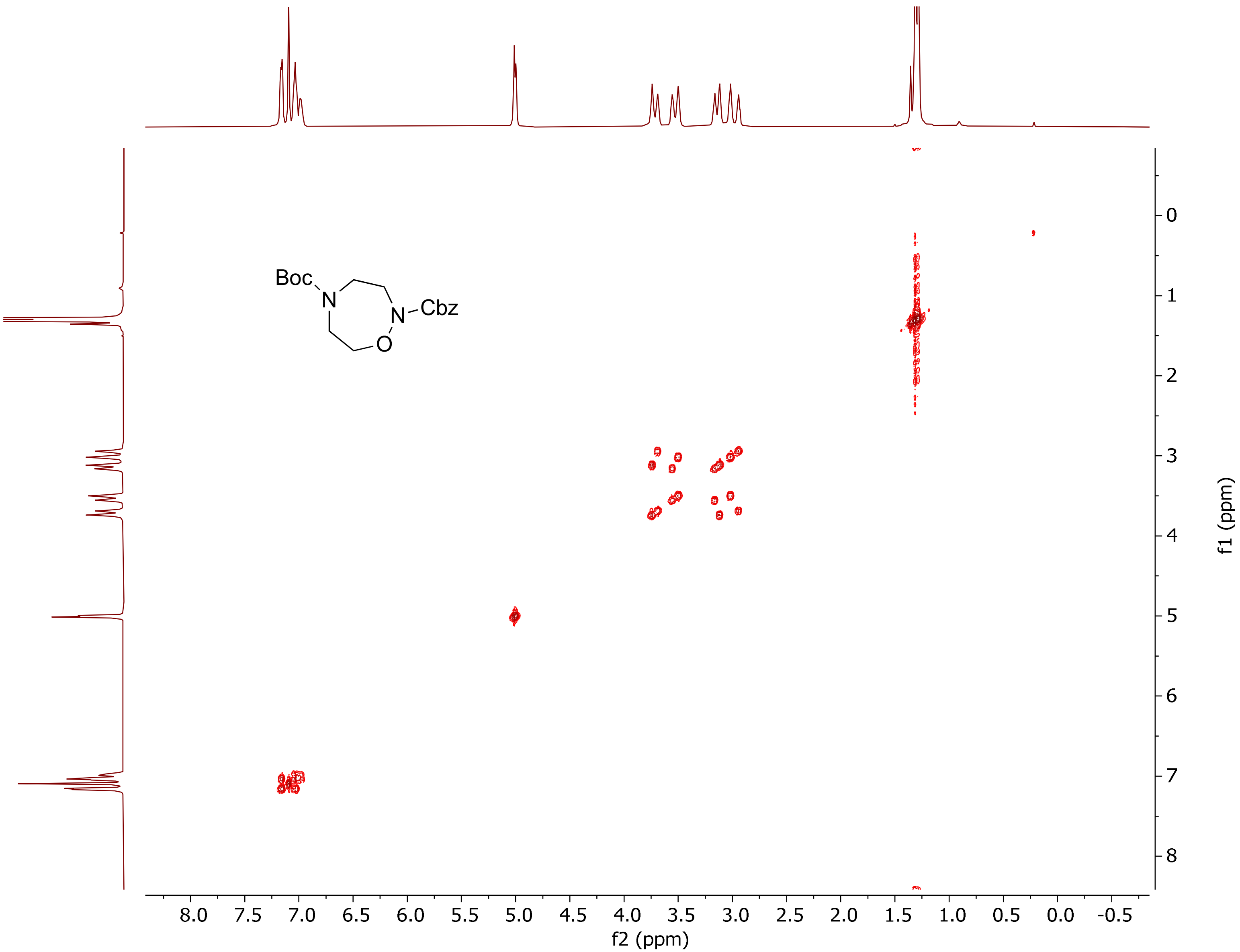

^{1}H NMR Spectrum (500 MHz, $CDCl_3$) of *tert*-butyl 1,2,5-oxadiazepane-5-carboxylate **(45)**

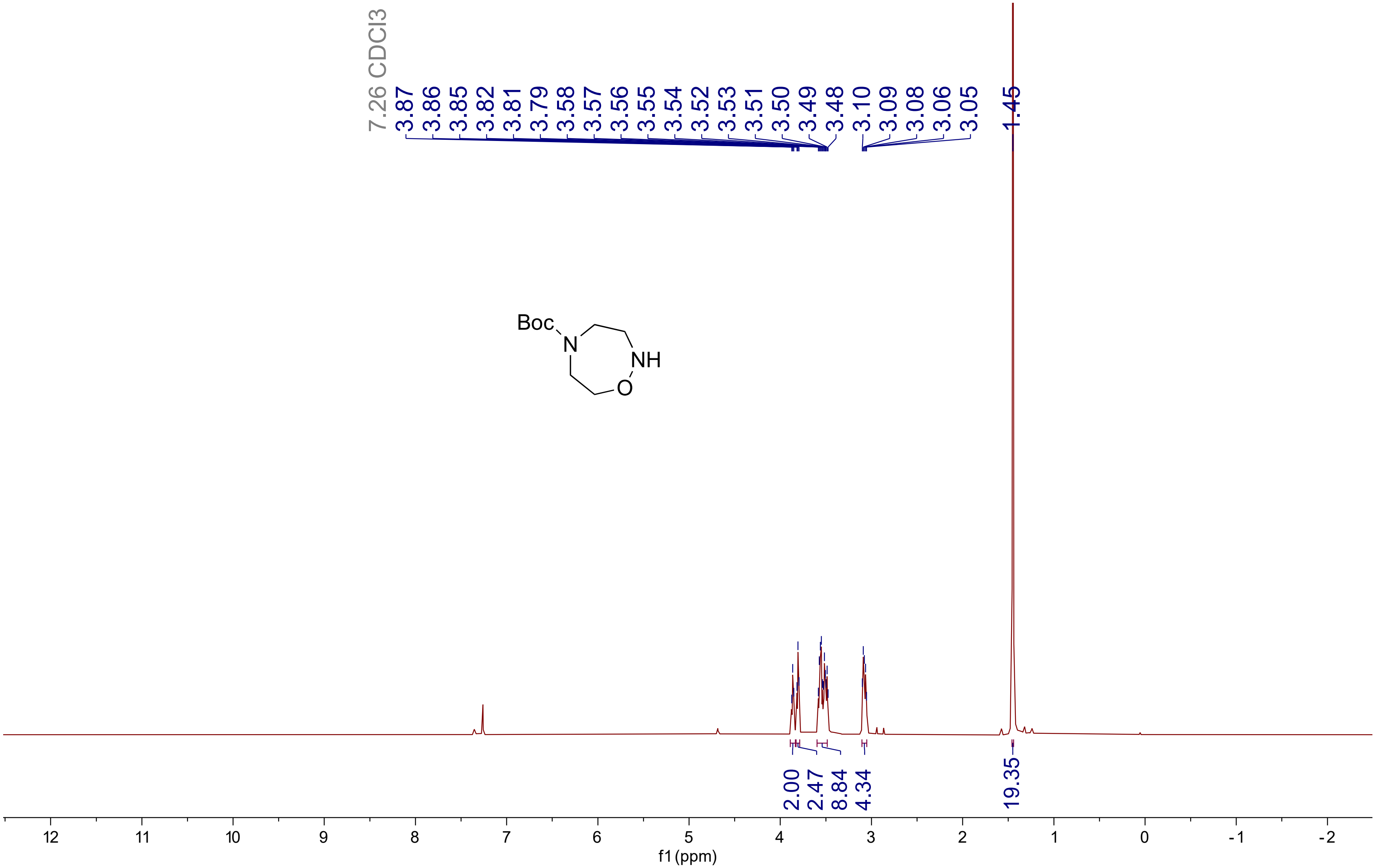

^{13}C NMR spectrum (126 MHz, $CDCl_3$) of *tert*-butyl 1,2,5-oxadiazepane-5-carboxylate **(45)**

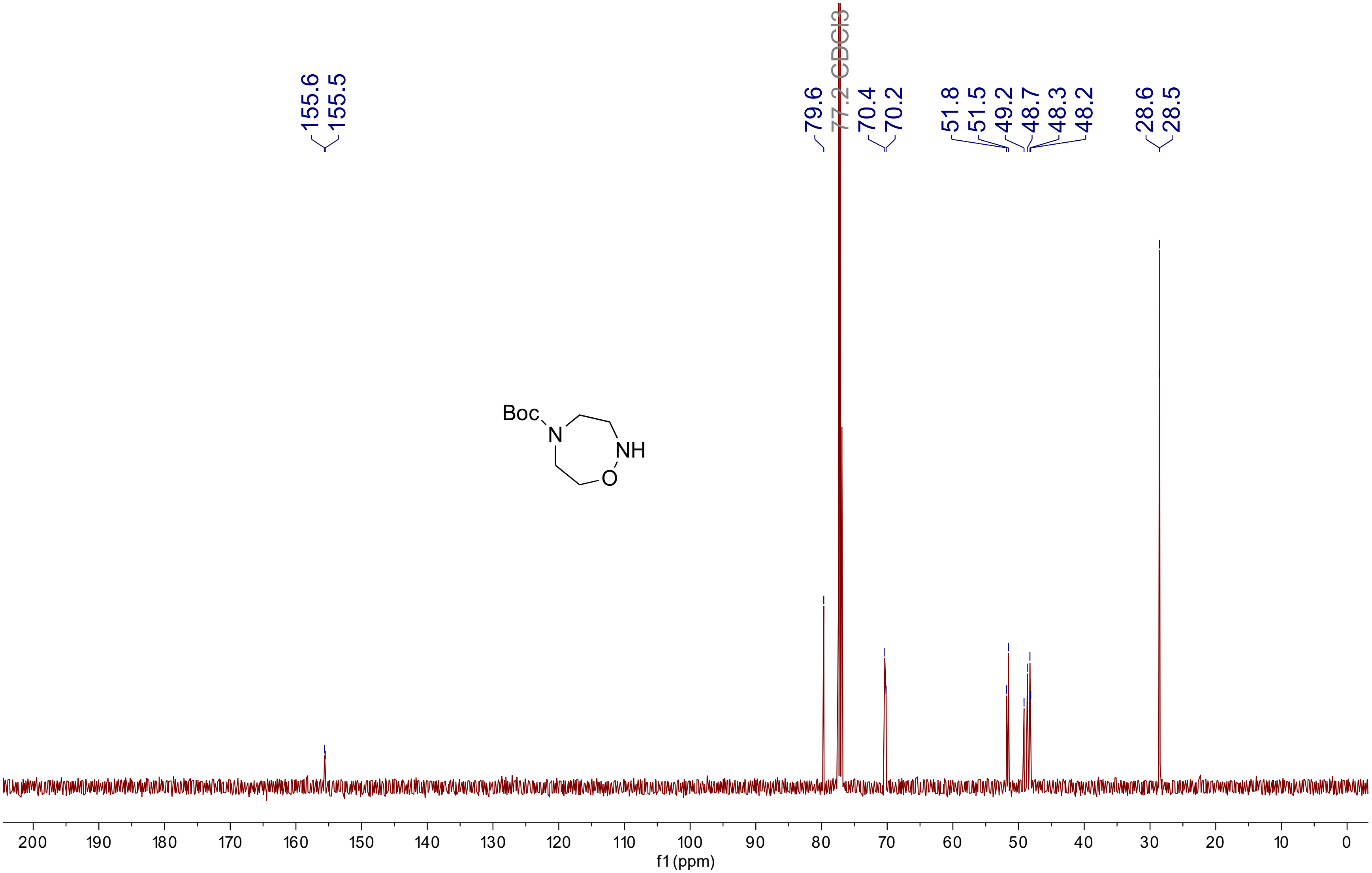

HSQC ($CDCl_3$) spectrum of *tert*-butyl 1,2,5-oxadiazepane-5-carboxylate **(45)**

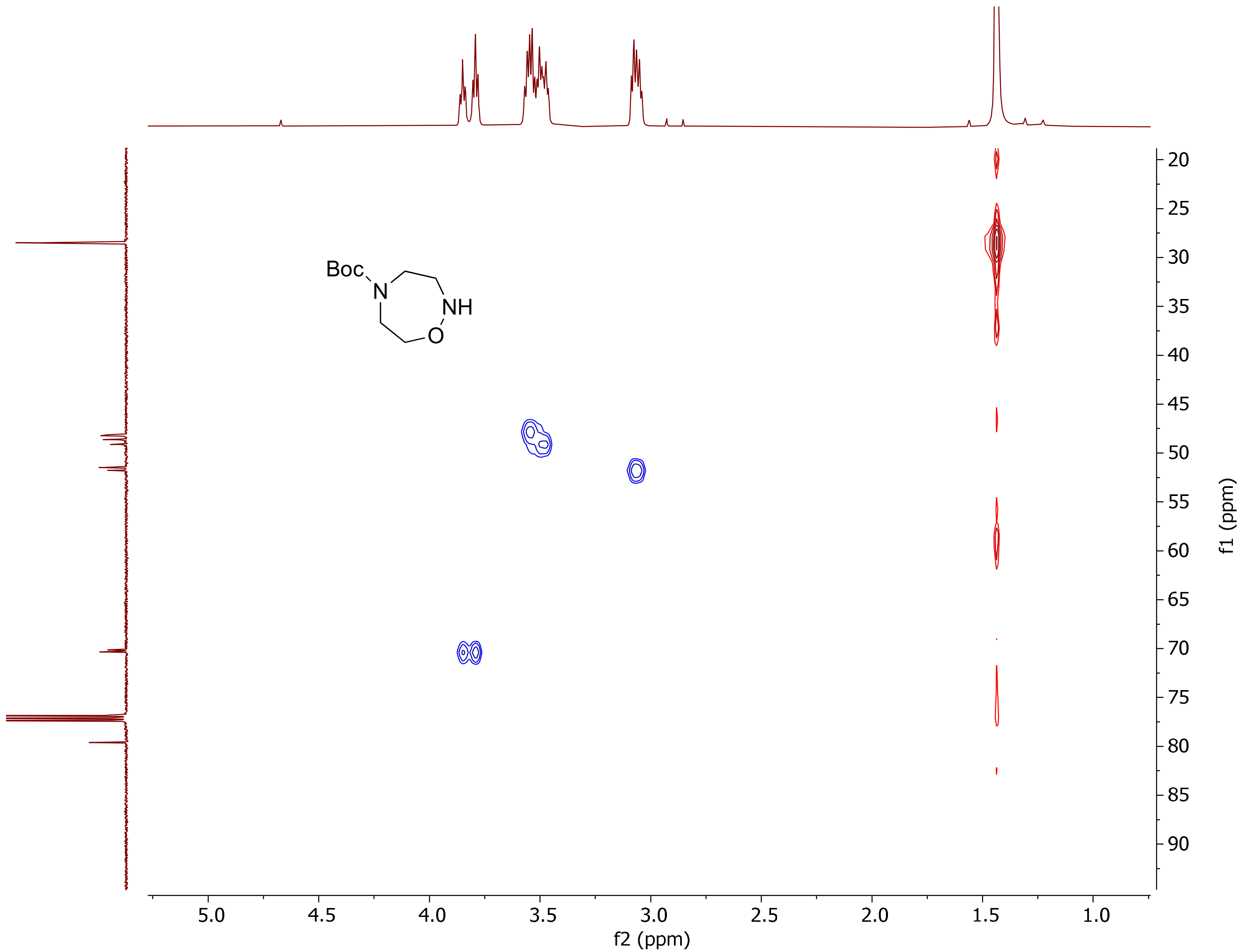

COSY ($CDCl_3$) spectrum of *tert*-butyl 1,2,5-oxadiazepane-5-carboxylate **(45)**

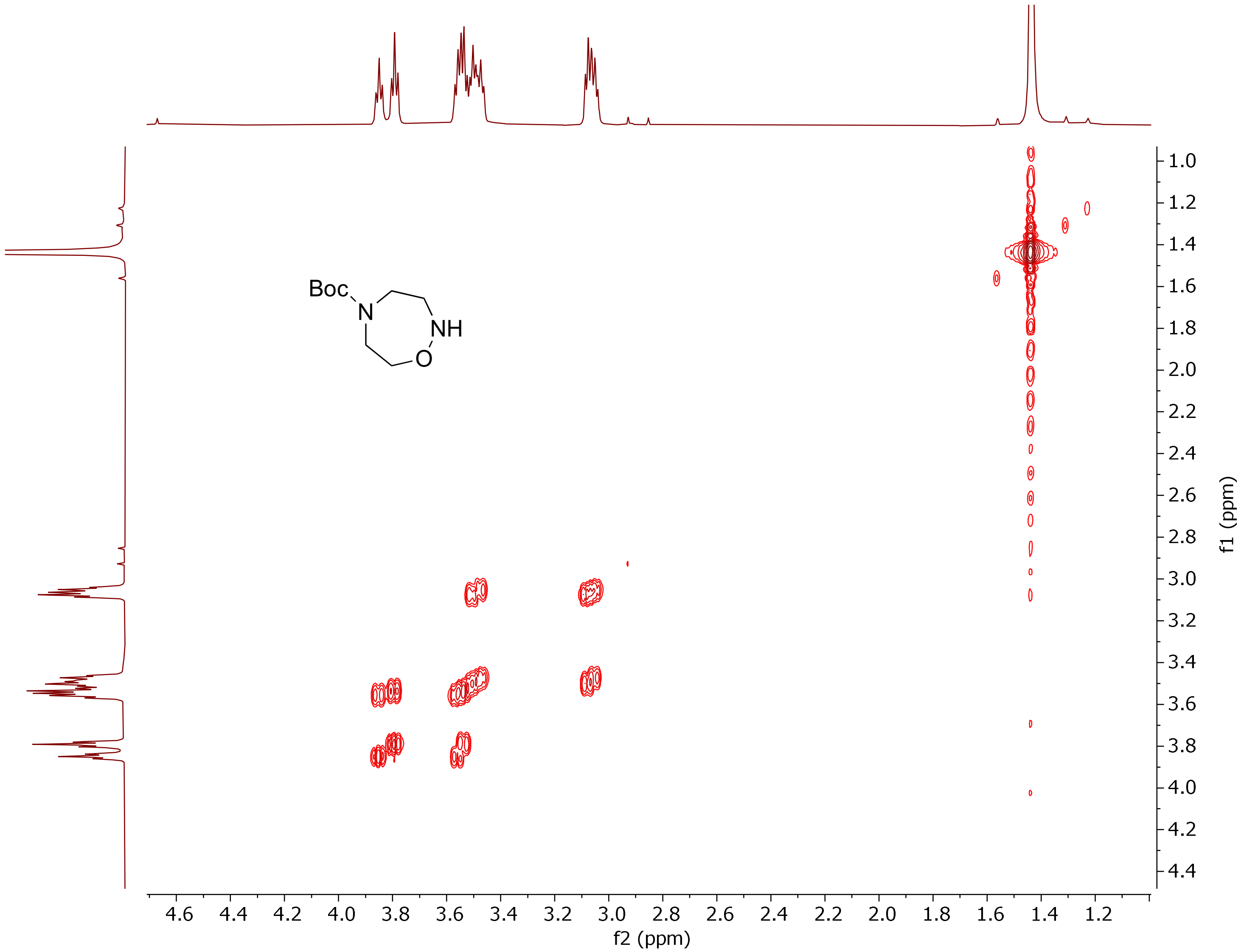

^{1}H NMR Spectrum (500 MHz, $CDCl_3$) of *tert*-butyl 2-methyl-1,2,5-oxadiazepane-5-carboxylate **(46)**

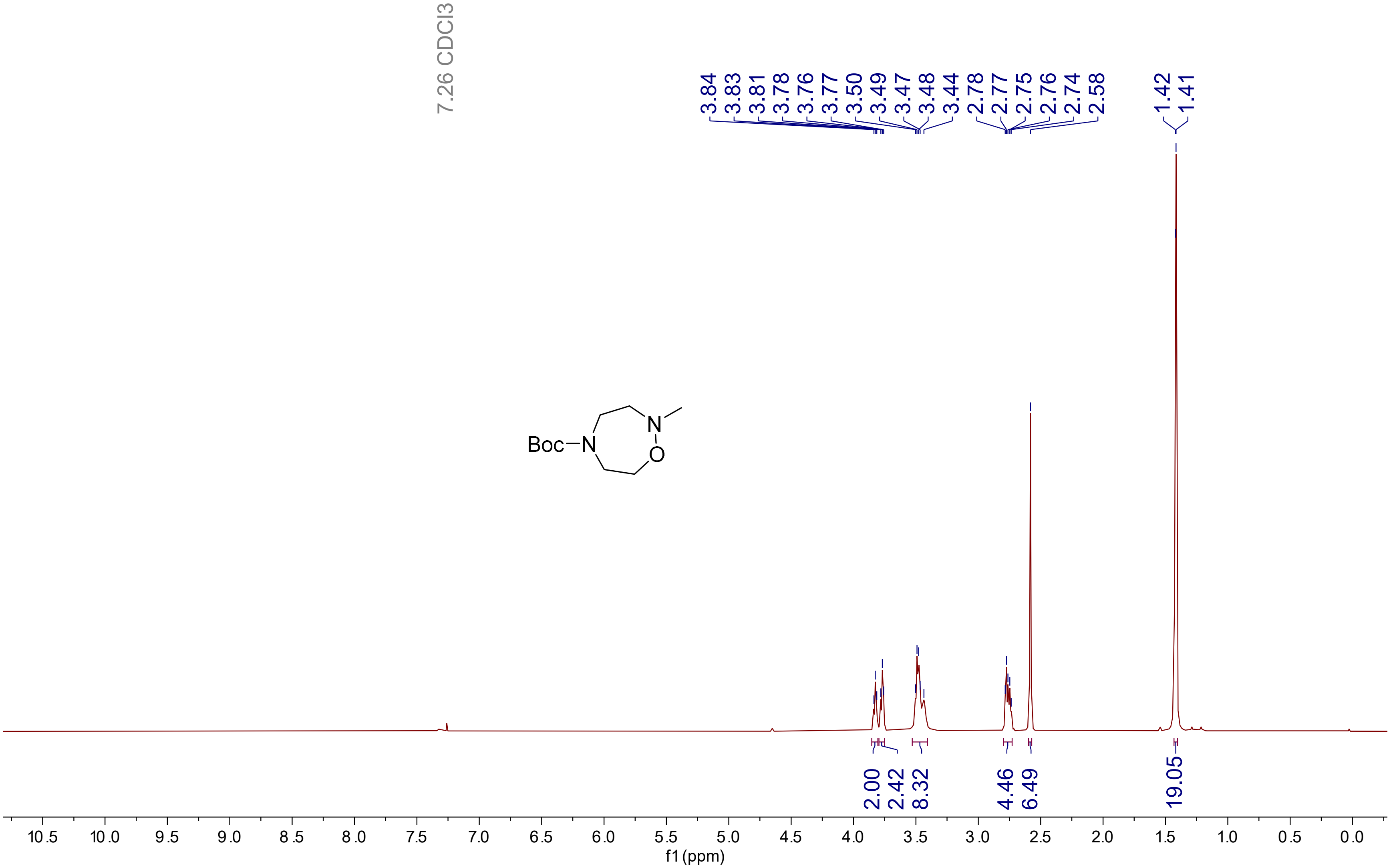

^{13}C NMR spectrum (126 MHz, $CDCl_3$) of *tert*-butyl 2-methyl-1,2,5-oxadiazepane-5-carboxylate **(46)**

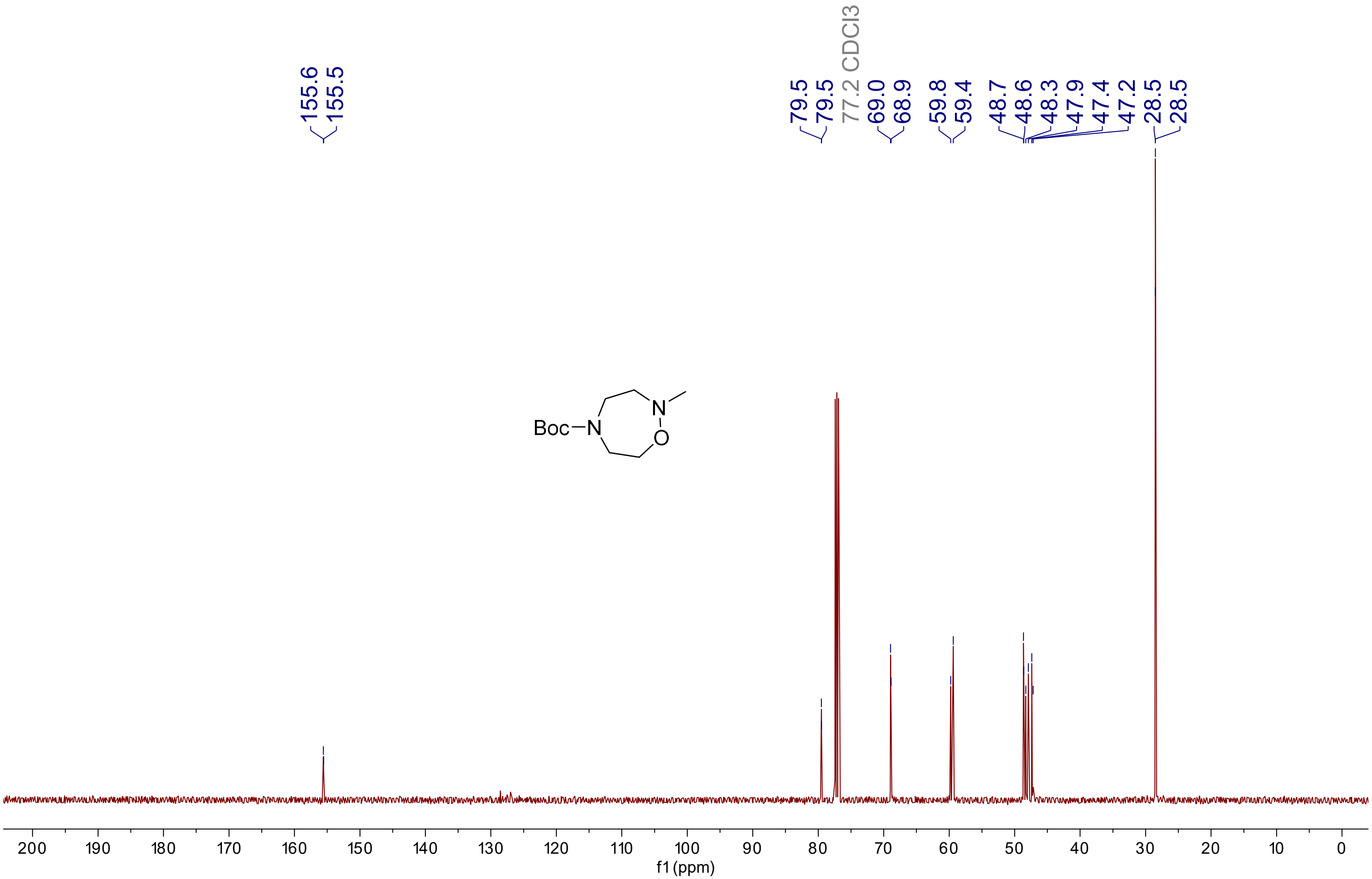

HSQC spectrum ($CDCl_3$) of *tert*-butyl 2-methyl-1,2,5-oxadiazepane-5-carboxylate **(46)**

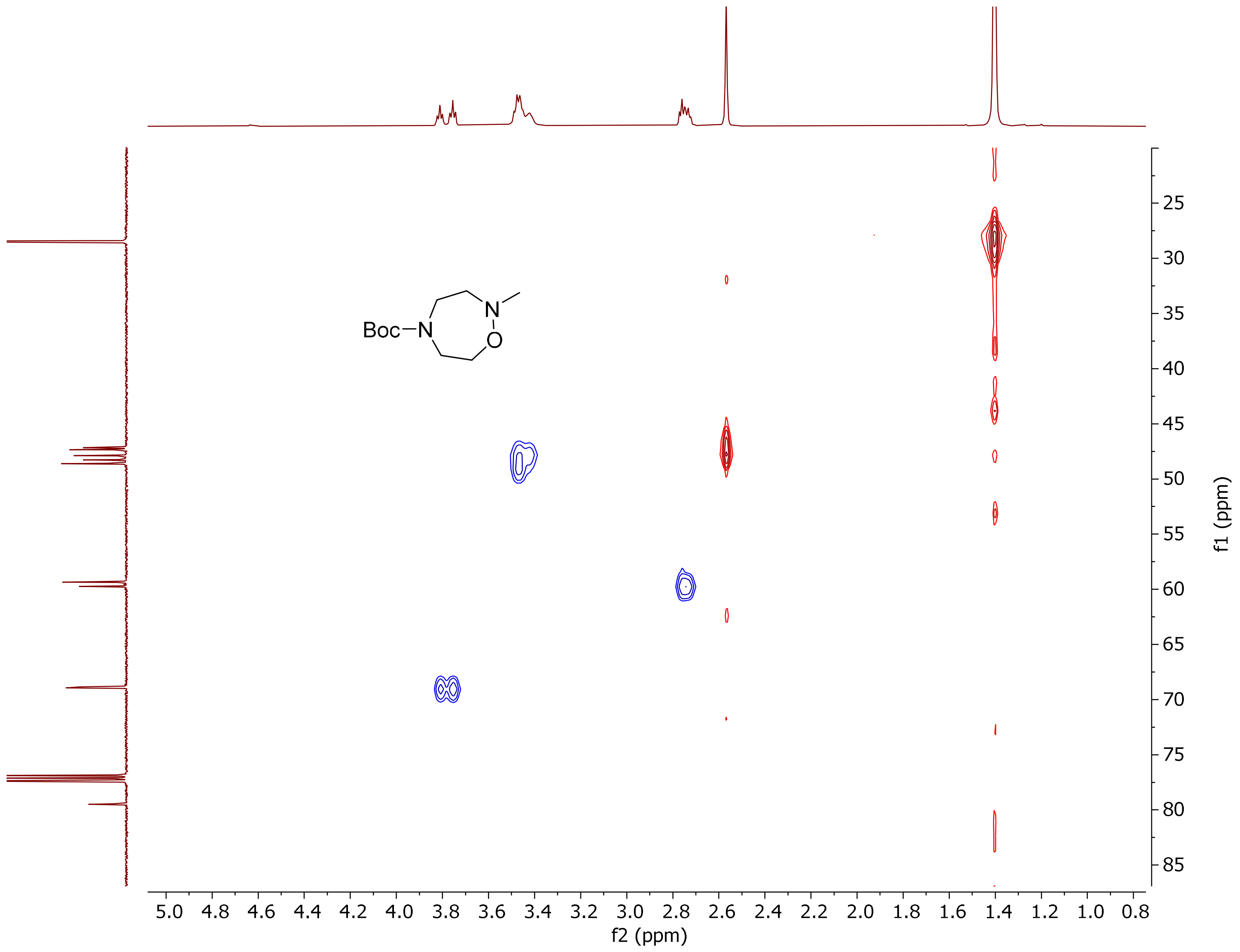

COSY spectrum ($CDCl_3$) of *tert*-butyl 2-methyl-1,2,5-oxadiazepane-5-carboxylate **(46)**

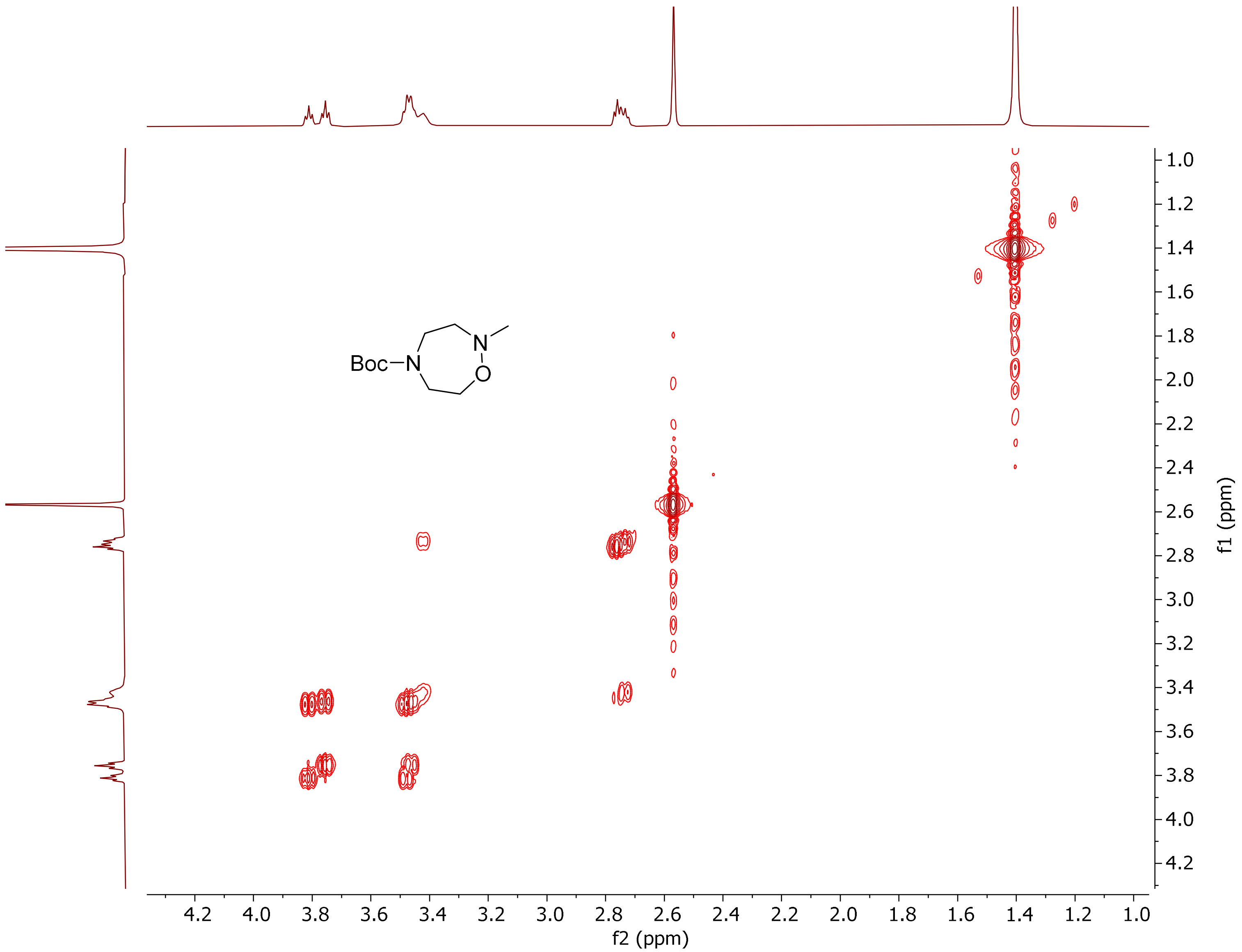

^{1}H NMR Spectrum (500 MHz, $CDCl_3$) of 2-(2-(2-methyl-1,2,5-oxadiazepan-5-yl)ethyl)isoindoline-1,3-dione **(48)**

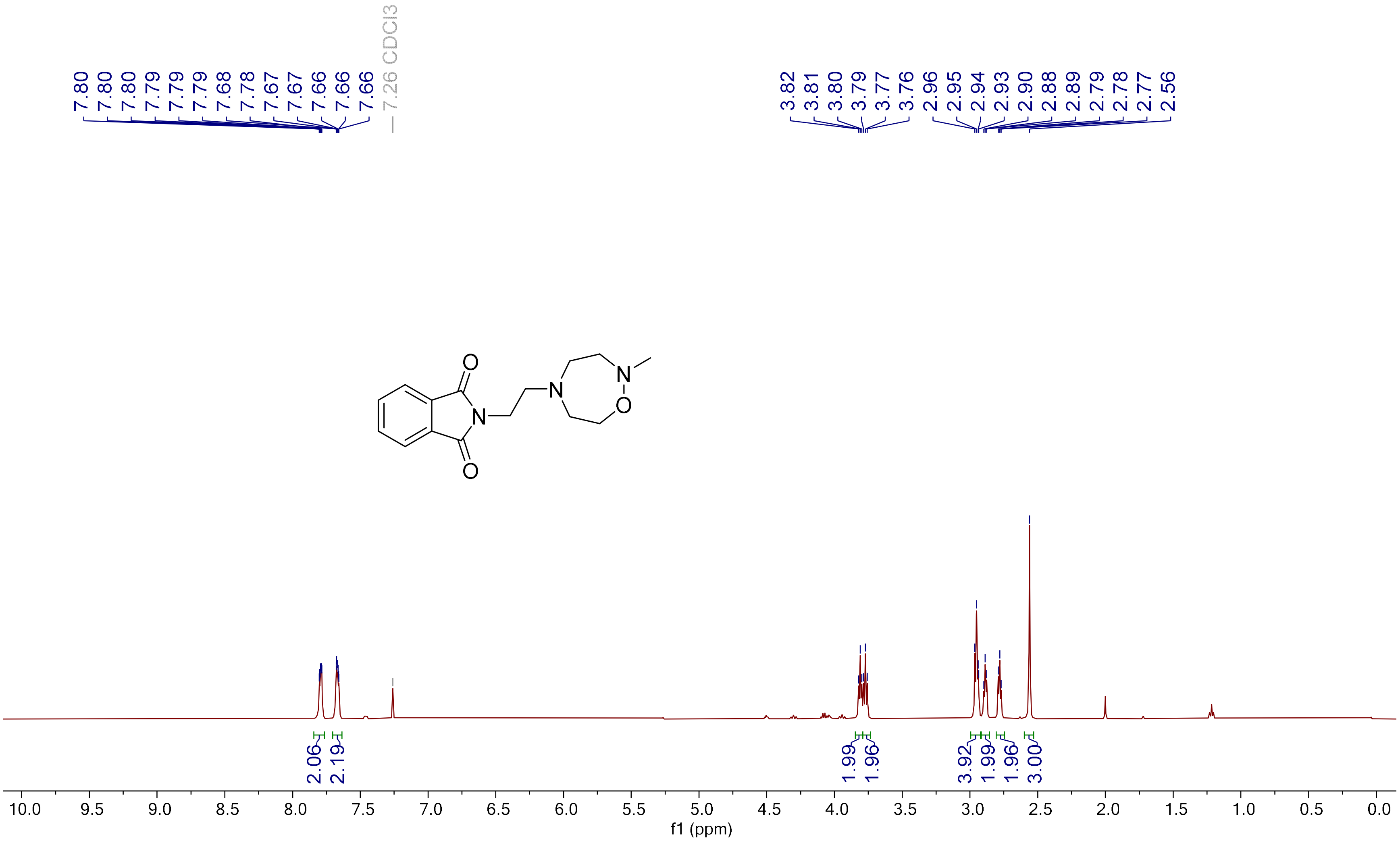

^{13}C NMR spectrum (126 MHz, $CDCl_3$) of 2-(2-(2-methyl-1,2,5-oxadiazepan-5-yl)ethyl)isoindoline-1,3-dione **(48)**

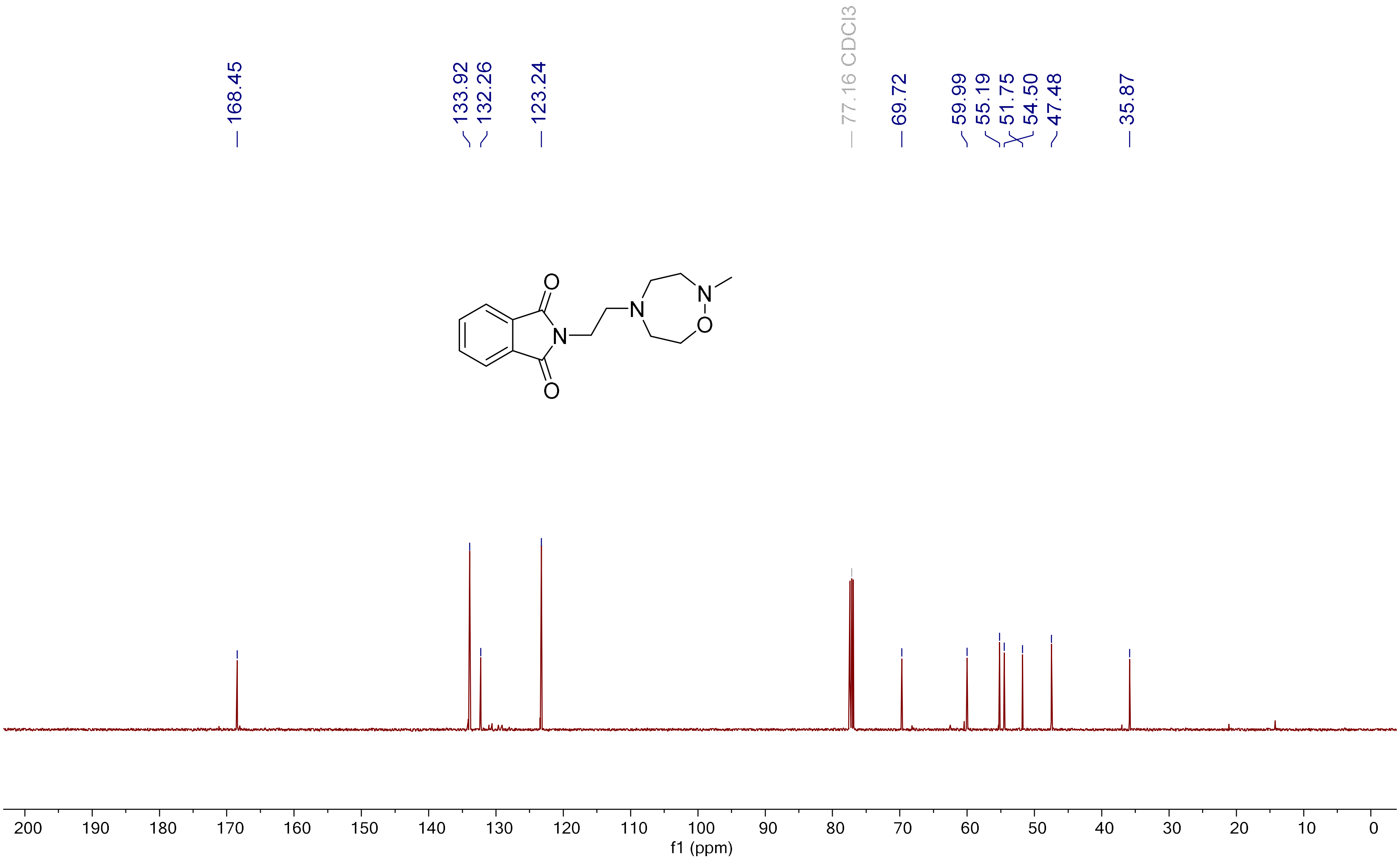

HSQC spectrum ($CDCl_3$) of 2-(2-(2-methyl-1,2,5-oxadiazepan-5-yl)ethyl)isoindoline-1,3-dione **(48)**

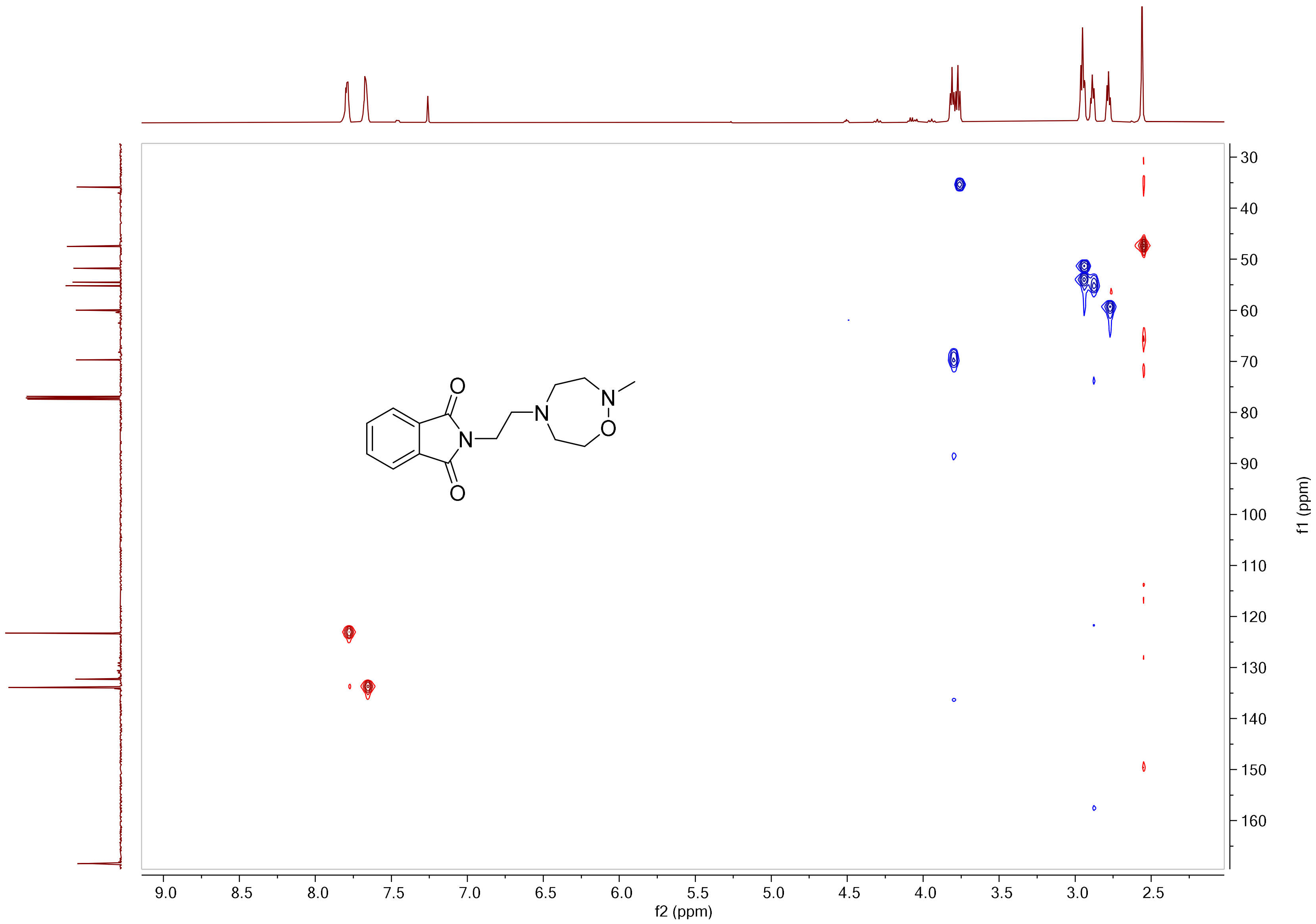

COSY spectrum ($CDCl_3$) of 2-(2-(2-methyl-1,2,5-oxadiazepan-5-yl)ethyl)isoindoline-1,3-dione **(48)**

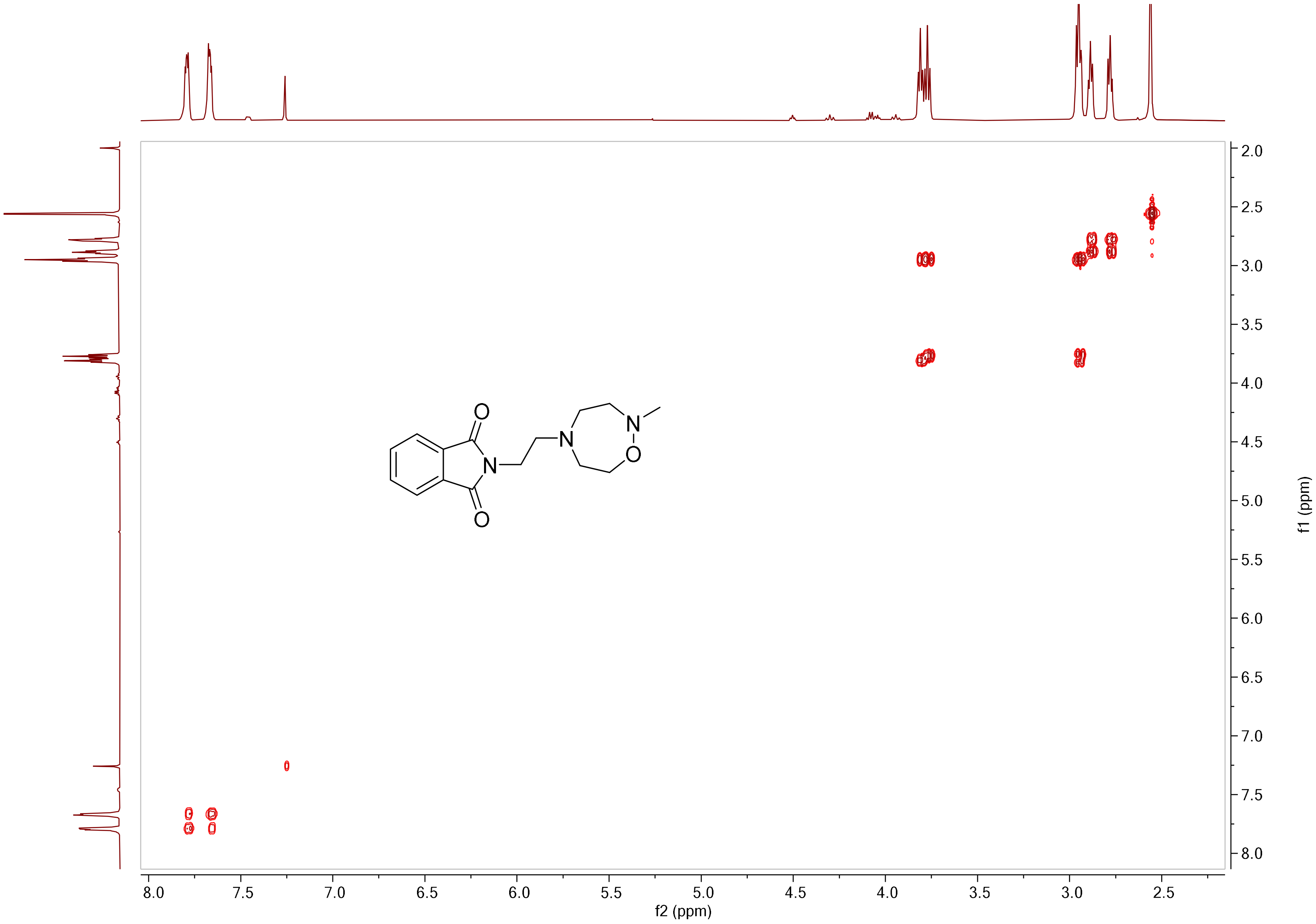

^{1}H NMR Spectrum (500 MHz, C_6D_6) of 2-(2-methyl-1,2,5-oxadiazepan-5-yl)ethan-1-amine **(49)**

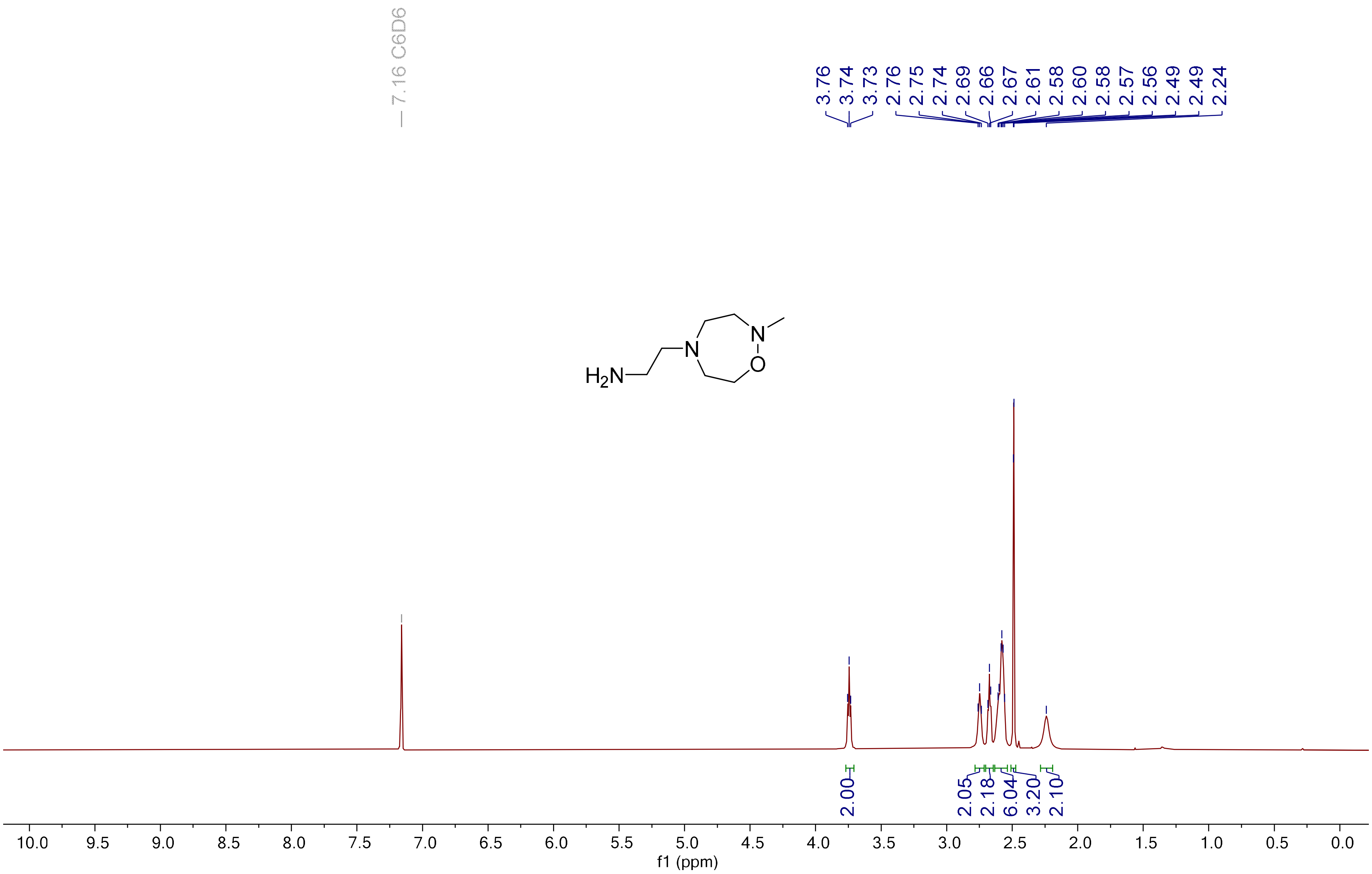

^{13}C NMR spectrum (126 MHz, C_6D_6) of 2-(2-methyl-1,2,5-oxadiazepan-5-yl)ethan-1-amine **(49)**

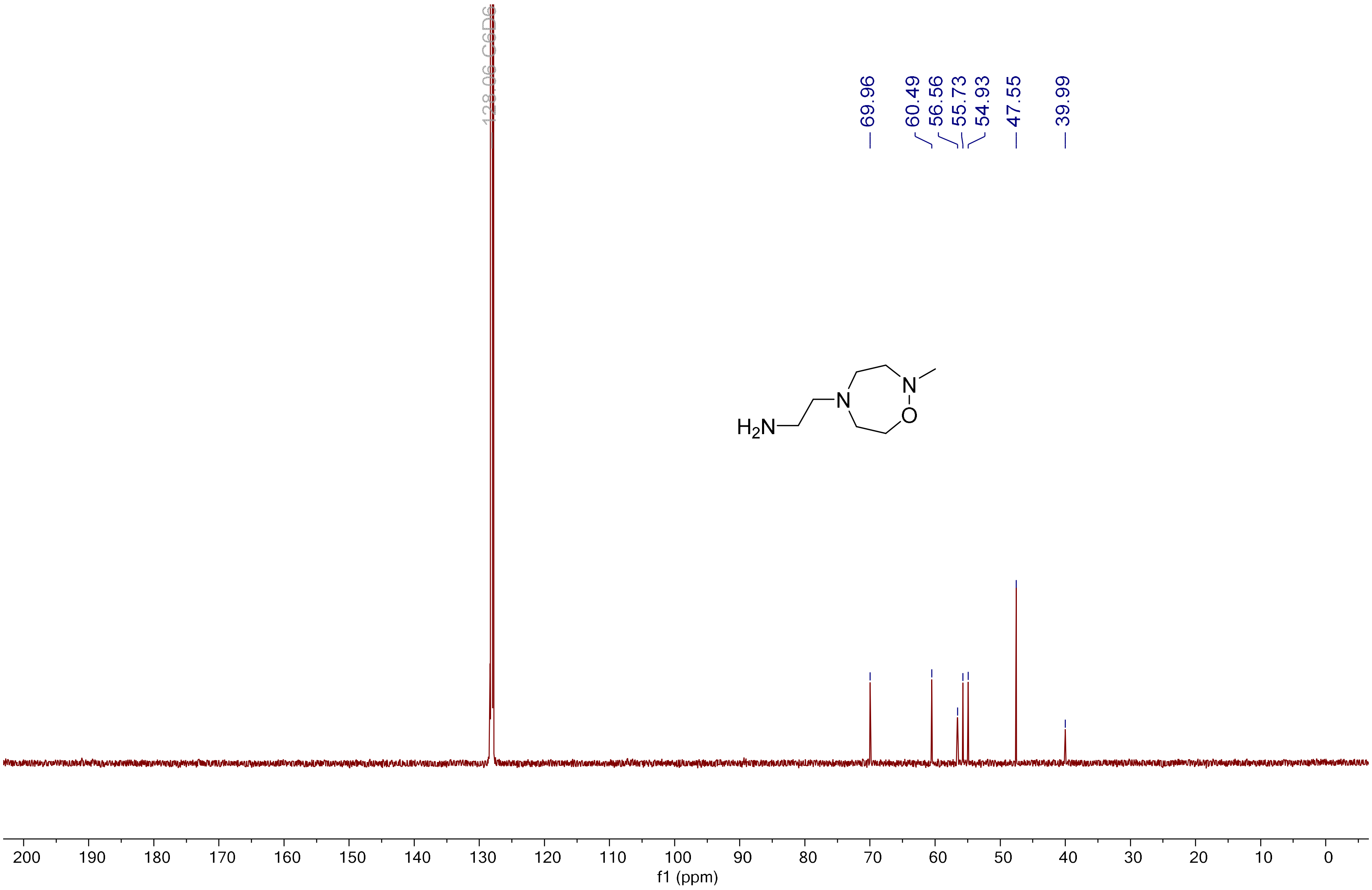

HSQC spectrum (C_6D_6) of 2-(2-methyl-1,2,5-oxadiazepan-5-yl)ethan-1-amine **(49)**

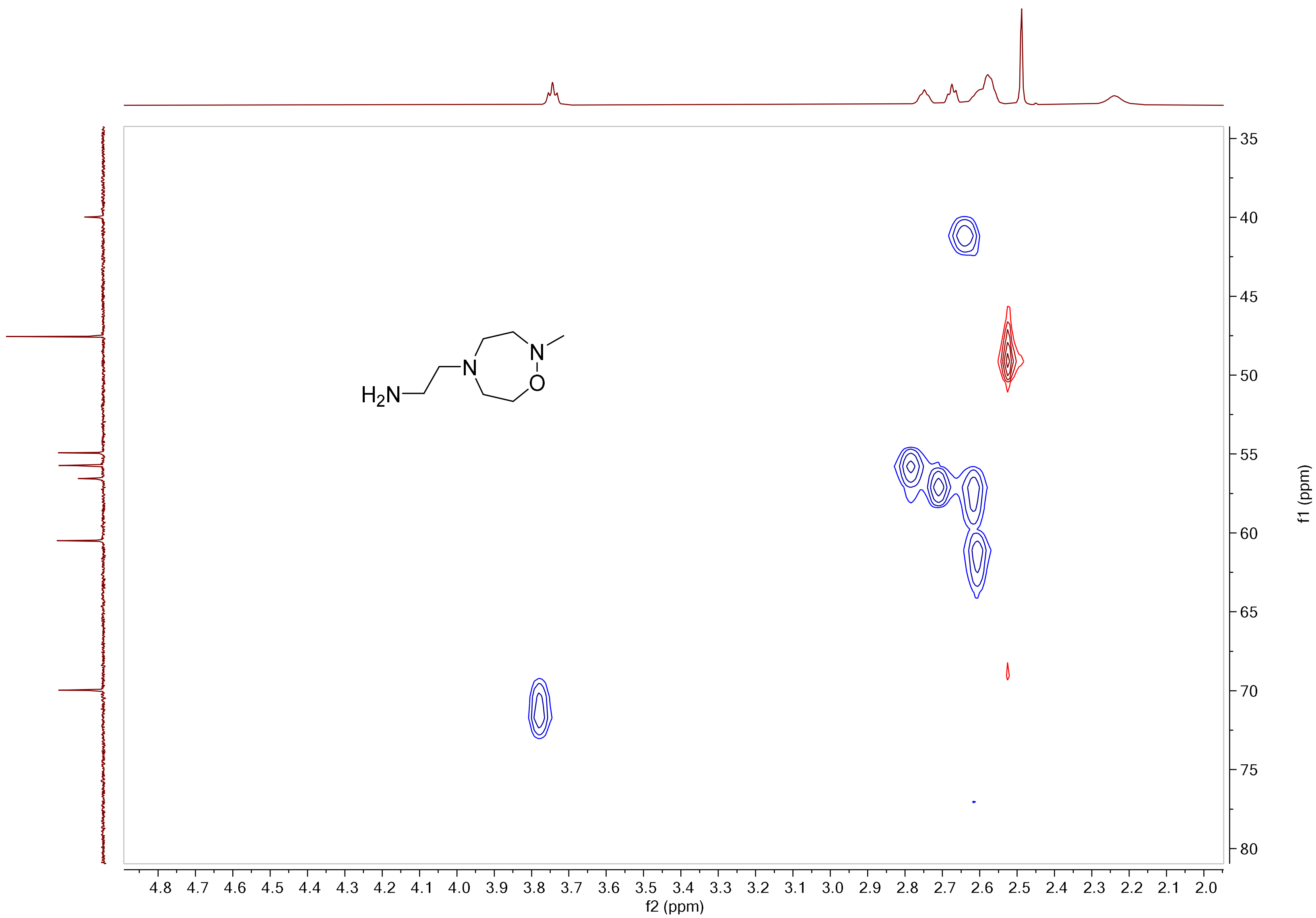

COSY spectrum (C_6D_6) of 2-(2-methyl-1,2,5-oxadiazepan-5-yl)ethan-1-amine **(49)**

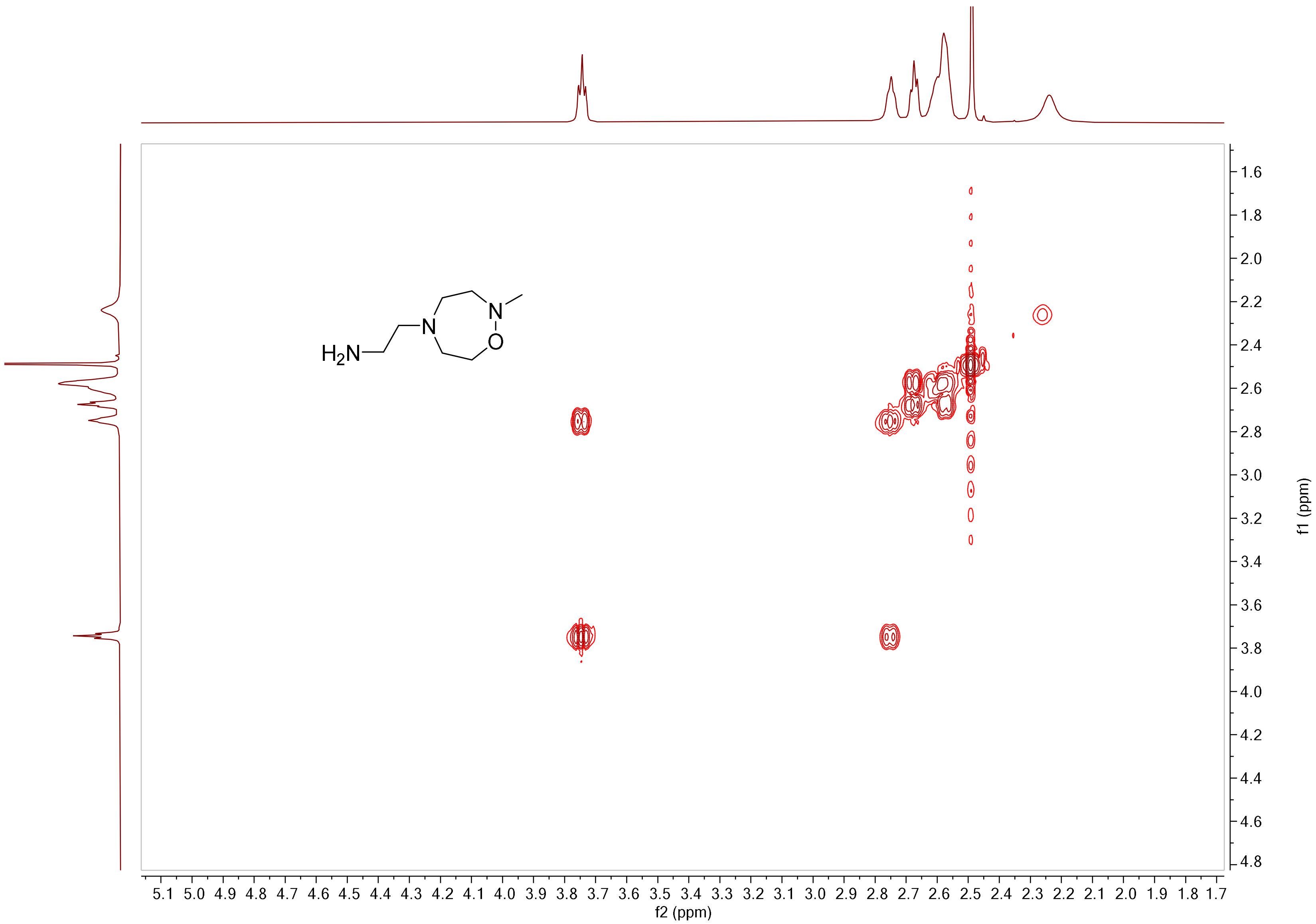

^{1}H NMR Spectrum (500 MHz, $CDCl_3$) of 6-(2-chloro-4-(6-methylpyrazin-2-yl)phenyl)-8-ethyl-2-((2-(2-methyl-1,2,5-oxadiazepan-5-yl)ethyl)amino)pyrido[2,3-*d*]pyrimidin-7(8*H*)-one **(13)**

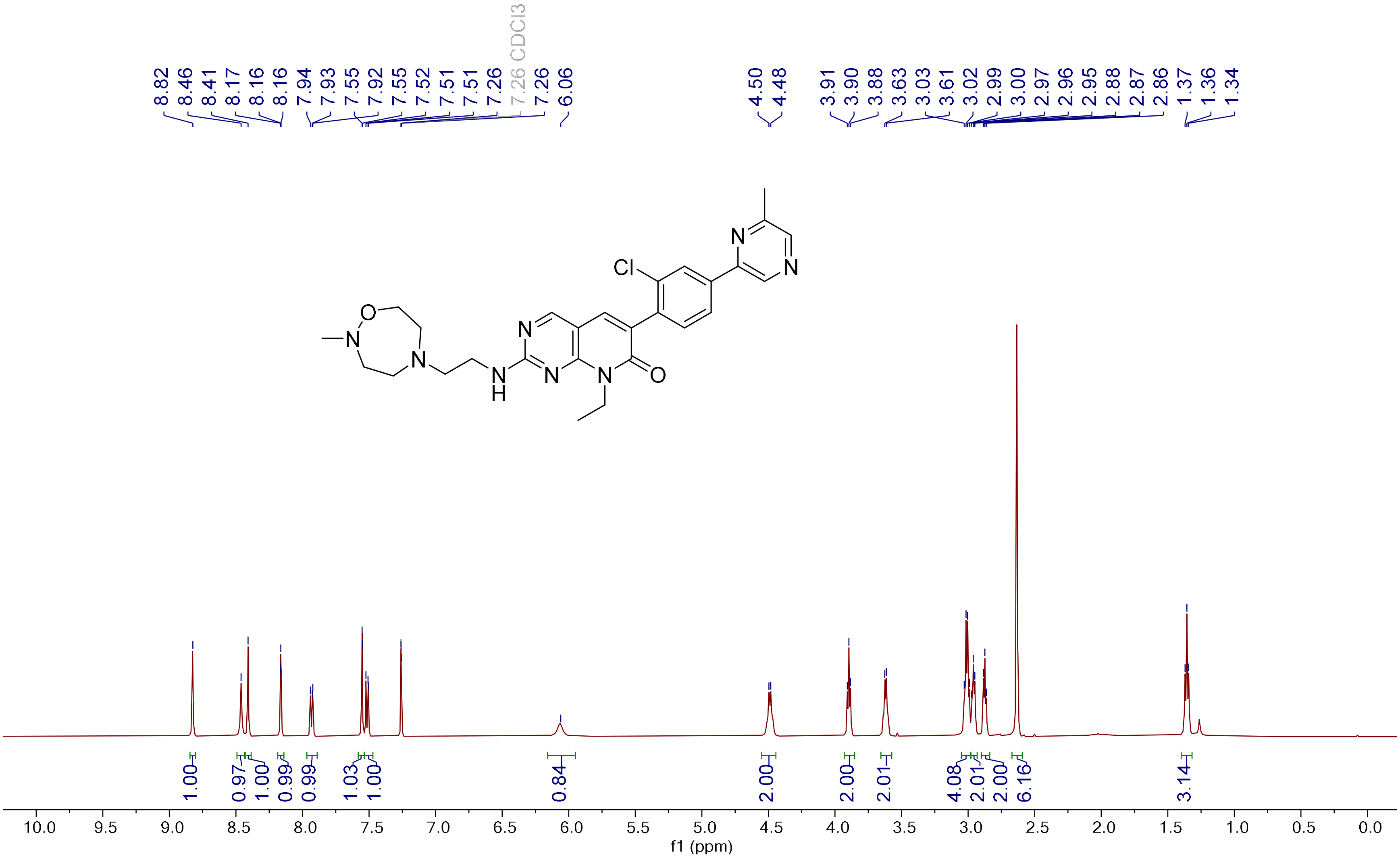

^{13}C NMR spectrum (126 MHz, $CDCl_3$) of 6-(2-chloro-4-(6-methylpyrazin-2-yl)phenyl)-8-ethyl-2-((2-(2-methyl-1,2,5-oxadiazepan-5-yl)ethyl)amino)pyrido[2,3-*d*]pyrimidin-7(8*H*)-one **(13)**

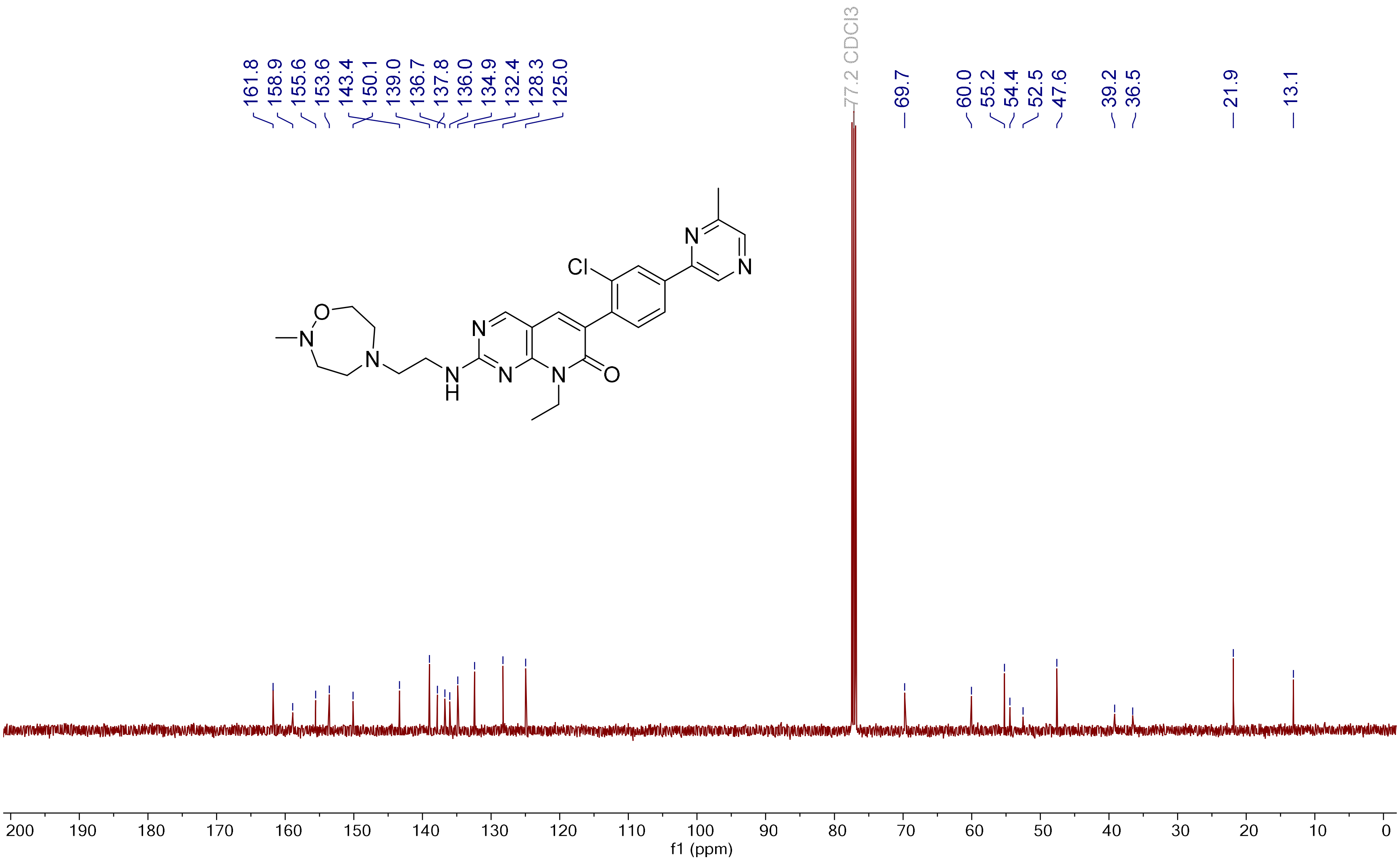

HSQC spectrum ($CDCl_3$) of 6-(2-chloro-4-(6-methylpyrazin-2-yl)phenyl)-8-ethyl-2-((2-(2-methyl-1,2,5-oxadiazepan-5-yl)ethyl)amino)pyrido[2,3-*d*]pyrimidin-7(8*H*)-one **(13)**

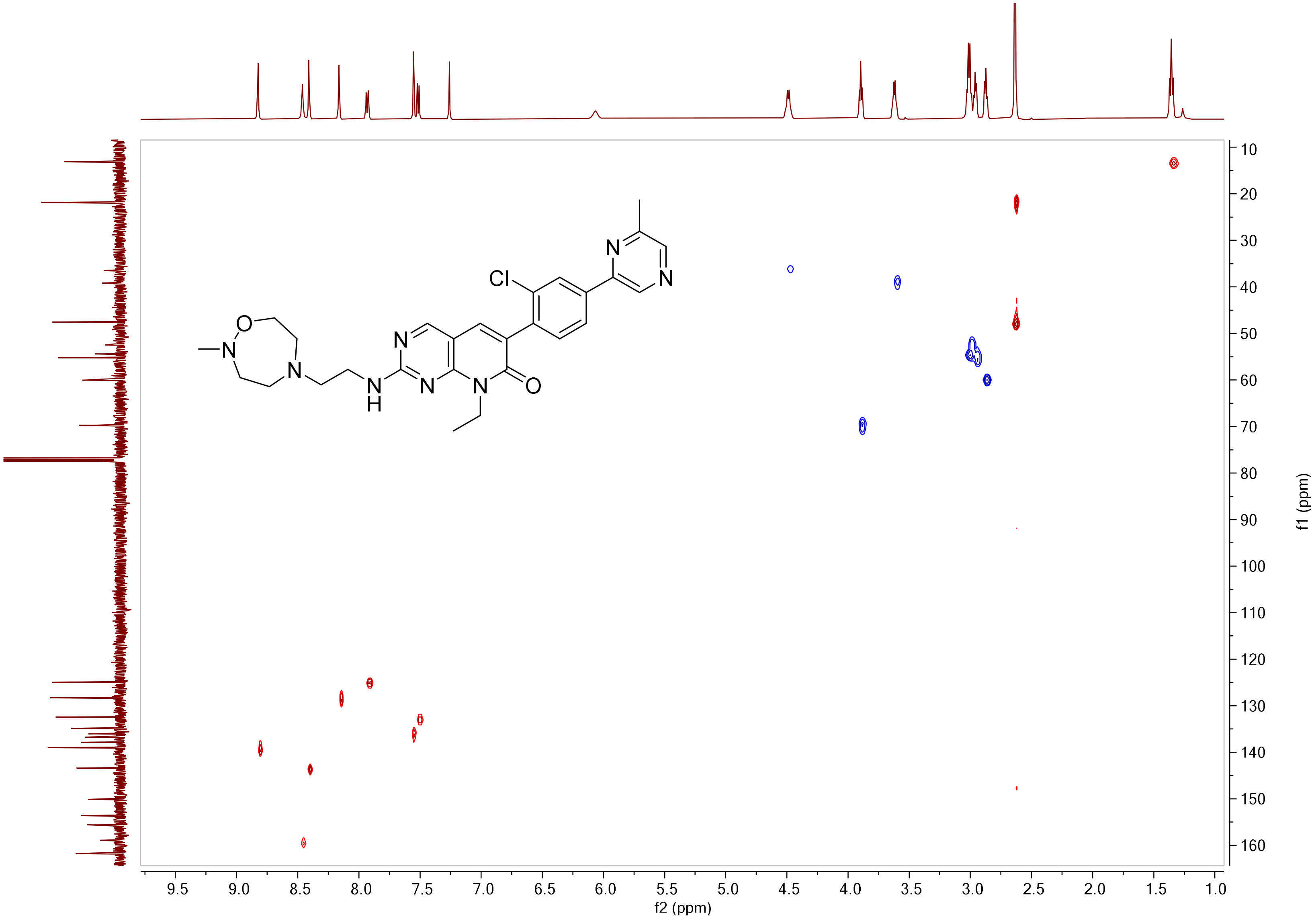

COSY spectrum ($CDCl_3$) of 6-(2-chloro-4-(6-methylpyrazin-2-yl)phenyl)-8-ethyl-2-((2-(2-methyl-1,2,5-oxadiazepan-5-yl)ethyl)amino)pyrido[2,3-*d*]pyrimidin-7(8*H*)-one **(13)**

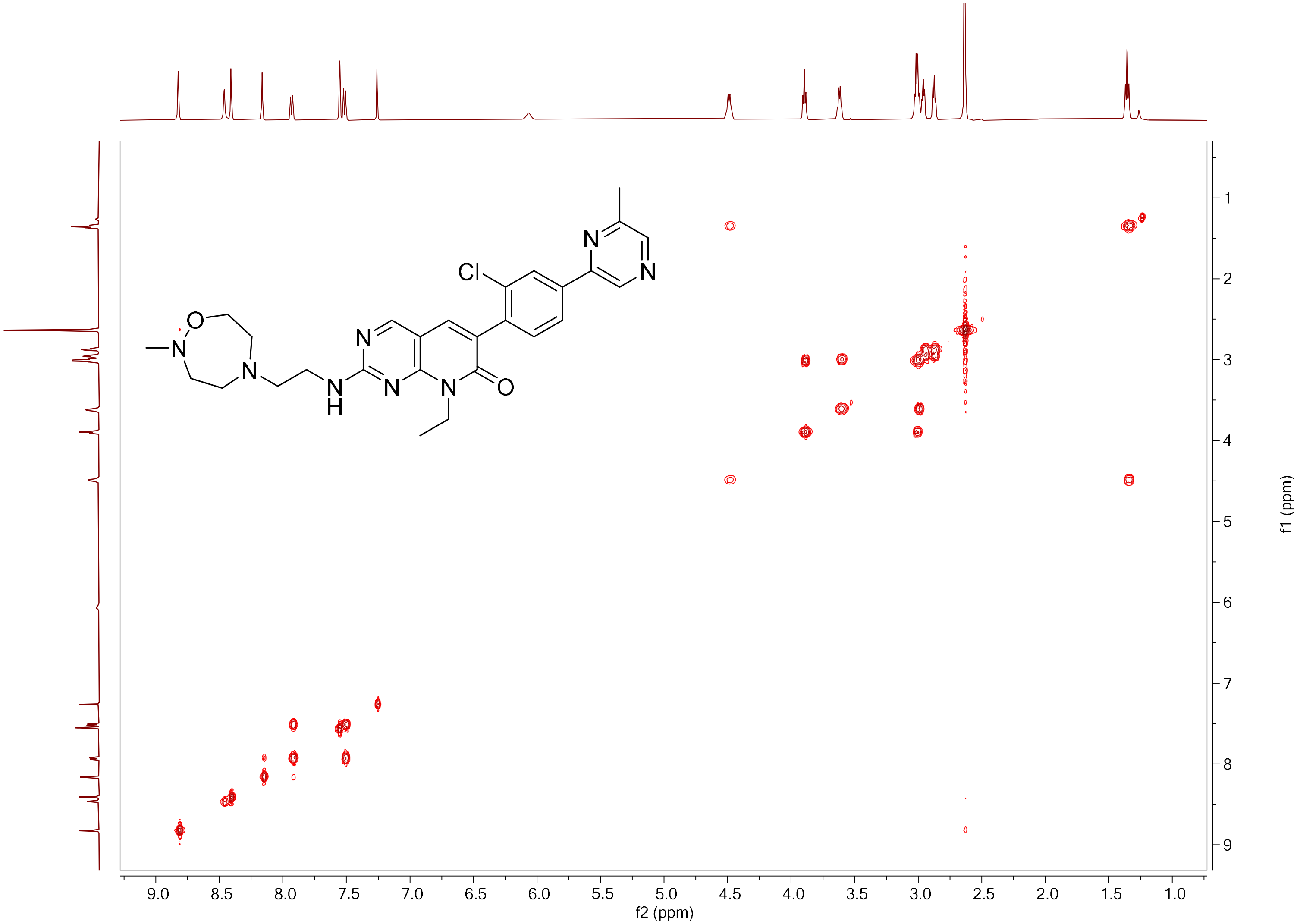

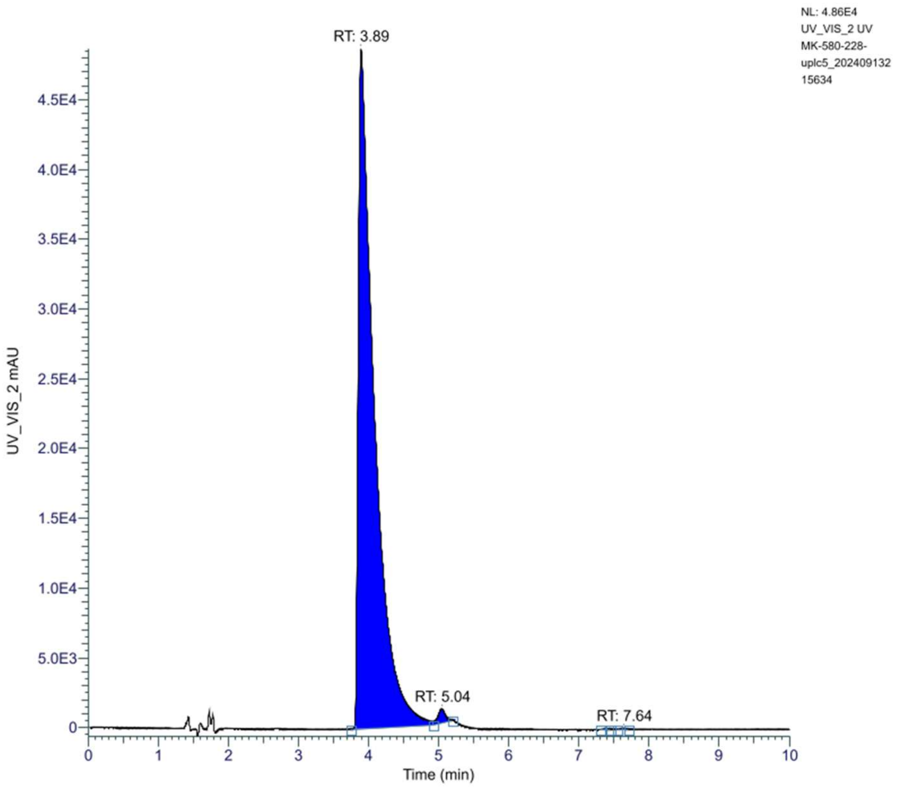

Figure S8. UHPLC trace of **6**. UHPLC/UV area percent purity of compound **6** at UV = 254 nm was determined to be 98.9% using automated Avalon peak area algorithm (peak list below).

Table S12. Peak List from Avalon peak area algorithm of compound **6** at UV = 254 nm.

**Retention Time (rt) (min)**	**Start rt (min)**	**End rt (min)**	**Peak Area**	**Area (%)**
3.89	3.74	4.91	7.793E+5	98.9
5.04	4.91	5.19	8.099E+3	1.03
7.40	7.30	7.44	2.819E+2	0.04
7.64	7.55	7.70	3.204E+2	0.04

Table S13. HPLC method. Mobile Phase A: CH_3CN. Mobile phase B: 0.1 % (v/v) formic acid in ultrapure H_2O. Flow rate: 1.000 [mL·min^{-1}], injection volume: 1.5 μL. Sample spiked with 0.1 mL formic acid.

**Time (min)**	**Mobile Phase A (%)**	**Mobile Phase B (%)**
0	38.5	61.5
10	55	45

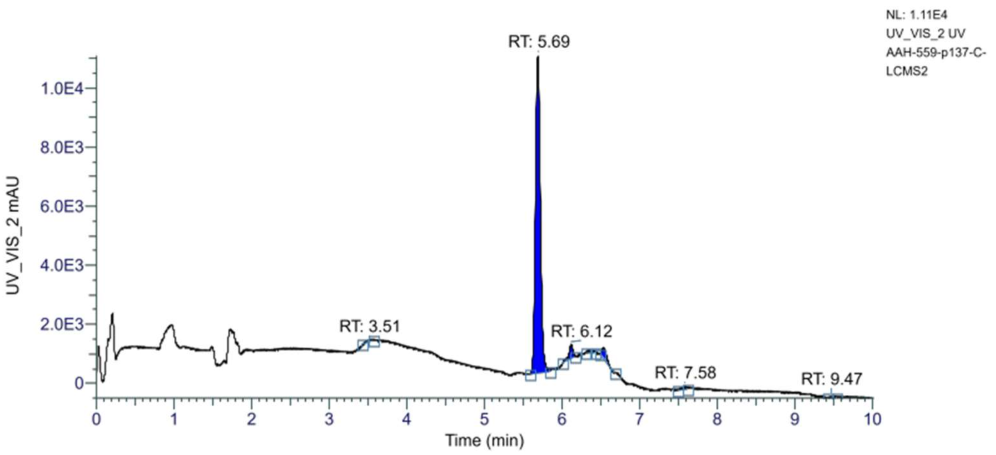

Figure S9. UHPLC trace of **8**. UHPLC/UV area percent purity of compound **8** at UV = 254 nm was determined to be 92.18% using automated Avalon peak area algorithm (peak list below).

Table S14. Peak List from Avalon peak area algorithm of compound **8** at UV = 254 nm.

Retention Time (rt) (min)	Start rt (min)	End rt (min)	Peak Area	Area (%)
3.51	3.42	3.58	2.499E+2	0.48
5.69	5.58	5.83	4.758E+4	92.18
6.12	6	6.16	1.611E+3	3.12
6.37	6.3	6.42	3.22E+2	0.62
6.54	6.49	6.68	1.418E+3	2.75
7.58	7.48	7.61	2.358E+2	0.46
9.47	9.41	9.53	1.988E+2	0.39

Table S15. HPLC method. Mobile Phase A: CH_3CN. Mobile phase B: 0.1 % (v/v) formic acid in ultrapure H_2O. Flow rate: 1.000 [$mL\cdot min^{-1}$], injection volume: 1.5 μL. Sample spiked with 0.1 mL formic acid.

Time (min)	Mobile Phase A (%)	Mobile Phase B (%)
0	55	45
10	0	100

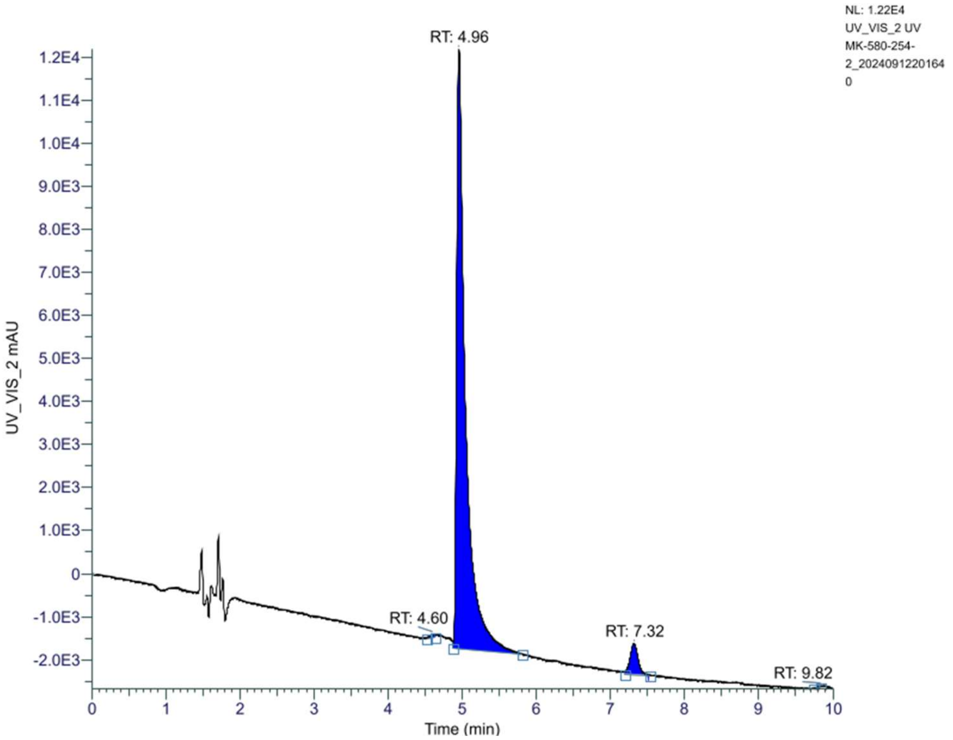

Figure S10. UHPLC trace of **9**. UHPLC/UV area percent purity of compound **9** at UV = 254 nm was determined to be 95.4% using automated Avalon peak area algorithm (peak list below).

Table S16. Peak List from Avalon peak area algorithm of compound **9** at UV = 254 nm.

Retention Time (rt) (min)	Start rt (min)	End rt (min)	Peak Area	Area (%)
4.60	4.52	4.63	2.522E+2	0.21
4.96	4.8	5.81	1.162E+5	95.4
7.32	7.19	7.52	5.249E+3	4.31
9.82	9.73	9.83	9.758E+1	0.08

Table S17. HPLC method. Mobile Phase A: CH_3CN. Mobile phase B: 0.1 % (v/v) formic acid in ultrapure H_2O. Flow rate: 1.000 [$mL \cdot min^{-1}$], injection volume: 1.5 µL. Sample spiked with 0.1 mL formic acid.

Time (min)	Mobile Phase A (%)	Mobile Phase B (%)
0	35	65
10	42.5	57.5

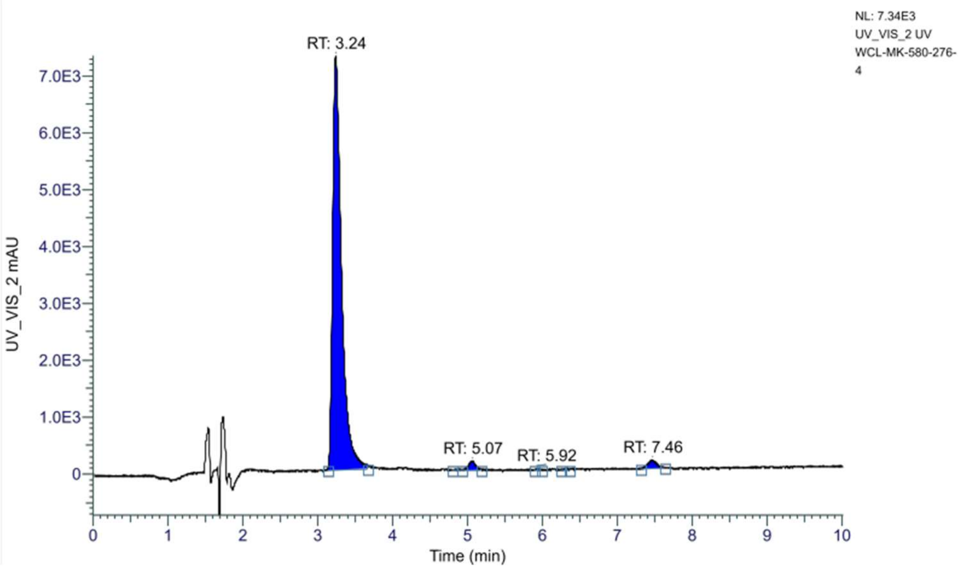

Figure S11. UHPLC trace of **10**. UHPLC/UV area percent purity of compound **10** at UV = 254 nm was determined to be 96.24% using automated Avalon peak area algorithm (peak list below).

Table S18. Peak List from Avalon peak area algorithm of compound **10** at UV = 254 nm.

Retention Time (rt) (min)	Start rt (min)	End rt (min)	Peak Area	Area (%)
3.24	3.13	3.44	6.042E+4	96.24
4.84	4.80	4.92	9.104E+1	0.15
5.07	4.92	5.18	1.03E+3	1.64
5.92	5.89	5.98	5.554E+1	0.09
6.29	6.24	6.35	6.392E+1	0.1
7.46	7.31	7.63	1.123E+3	1.79

Table S19. HPLC method. Mobile Phase A: CH_3CN. Mobile phase B: 0.1 % (v/v) formic acid in ultrapure H_2O. Flow rate: 1.000 [$mL \cdot min^{-1}$], injection volume: 1.5 µL. Sample spiked with 0.1 mL formic acid.

Time (min)	Mobile Phase A (%)	Mobile Phase B (%)
0	37.5	62.5
10	40	60

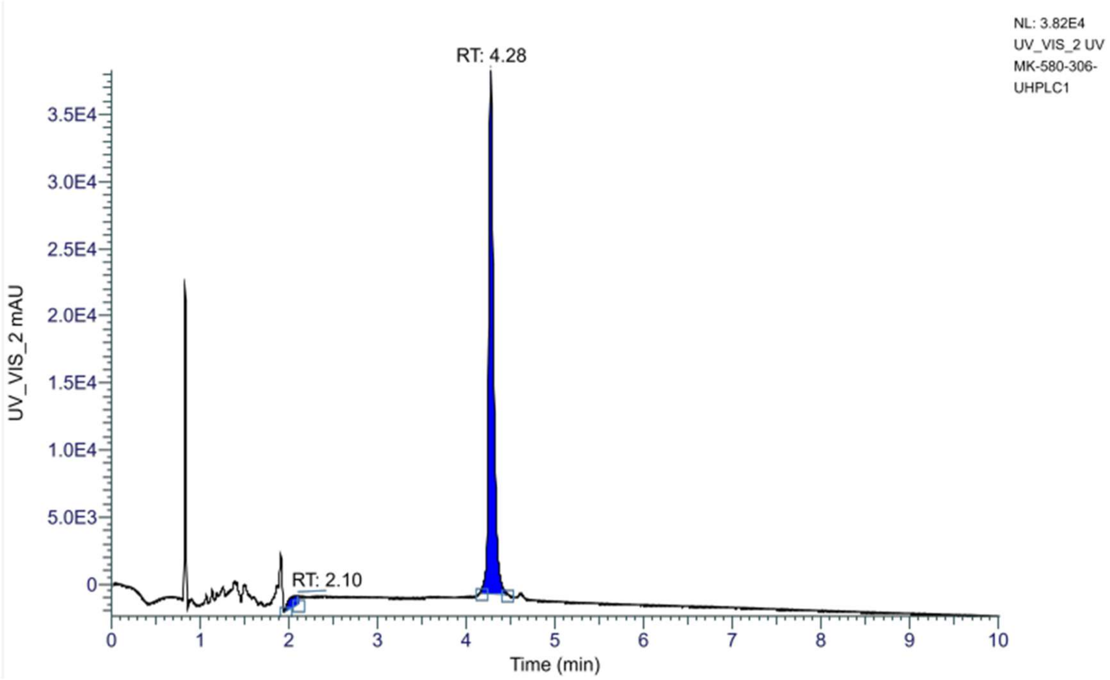

Figure S12. UHPLC trace of **11**. UHPLC/UV area percent purity of compound **11** at UV = 254 nm was determined to be 96.39% using automated Avalon peak area algorithm (peak list below).

Table S20. Peak List from Avalon peak area algorithm of compound **11** at UV = 254 nm.

Retention Time (rt) (min)	Start rt (min)	End rt (min)	Peak Area	Area (%)
2.1	1.95	2.11	5.778E+3	3.61
4.28	4.16	4.46	1.542E+5	96.39

Table S21. HPLC method. Mobile Phase A: CH_3CN. Mobile phase B: 0.1 % (v/v) formic acid in ultrapure H_2O. Flow rate: 1.000 [$mL\cdot min^{-1}$], injection volume: 1.5 µL. Sample spiked with 0.1 mL formic acid.

Time (min)	Mobile Phase A (%)	Mobile Phase B (%)
0	30	70
10	55	45

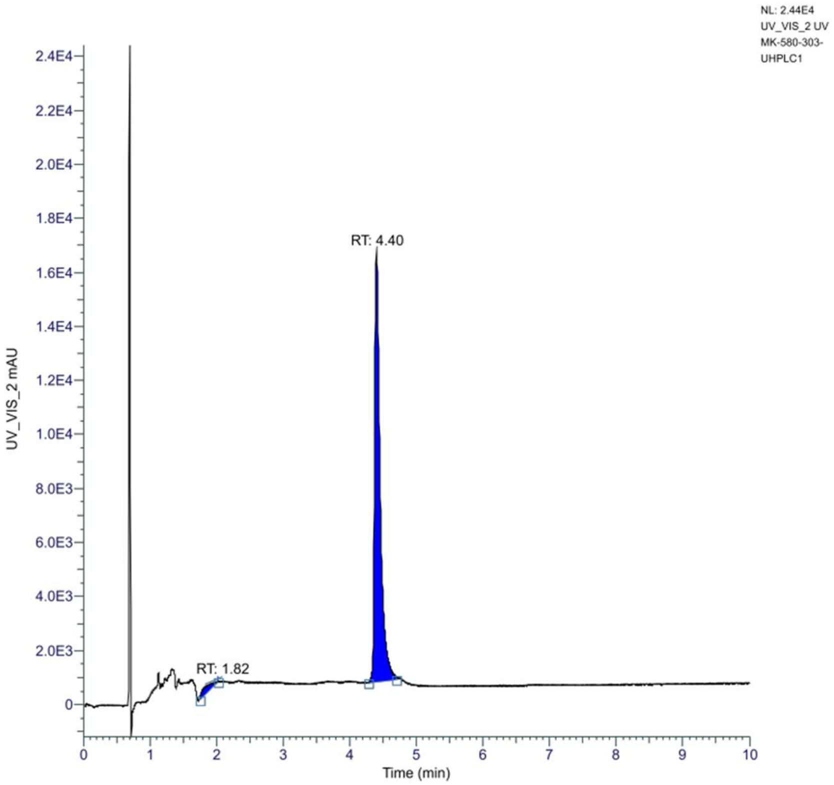

Figure S13. UHPLC trace of **12**. UHPLC/UV area percent purity of compound **12** at UV = 254 nm was determined to be 96.98% using automated Avalon peak area algorithm (peak list below).

Table S22. Peak List from Avalon peak area algorithm of compound **12** at UV = 254 nm.

Retention Time (rt) (min)	Start rt (min)	End rt (min)	Peak Area	Area (%)
1.82	1.75	2.02	2.934E+3	3.02
4.40	4.27	4.70	9.436E+4	96.98

Table S23. HPLC method. Mobile Phase A: CH_3CN. Mobile phase B: 0.1 % (v/v) formic acid in ultrapure H_2O. Flow rate: 1.000 [$mL\cdot min^{-1}$], injection volume: 1.5 µL. Sample spiked with 0.1 mL formic acid.

Time (min)	Mobile Phase A (%)	Mobile Phase B (%)
0	30	70
10	60	40

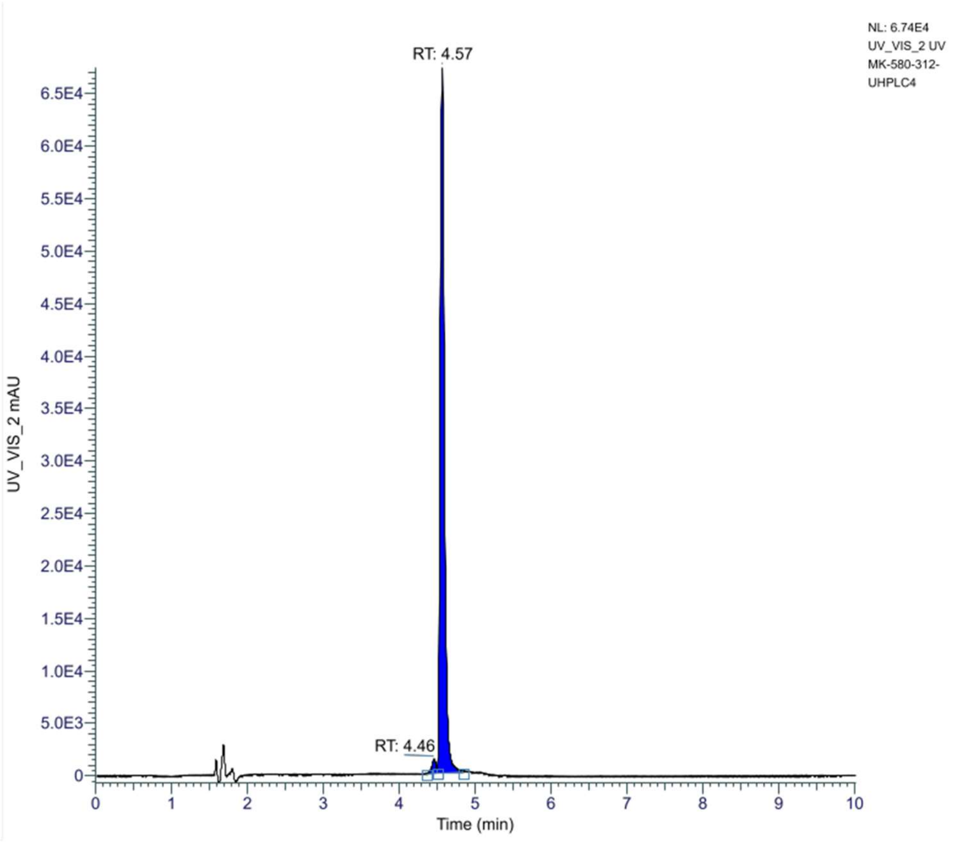

Figure S14. UHPLC trace of **13**. UHPLC/UV area percent purity of compound **13** at UV = 254 nm was determined to be 98.19% using automated Avalon peak area algorithm (peak list below).

Table S24. Peak List from Avalon peak area algorithm of compound **13** at UV = 254 nm.

Retention Time (rt) (min)	Start rt (min)	End rt (min)	Peak Area	Area (%)
4.46	4.36	4.50	5.091E+3	1.81
4.57	4.50	4.85	2.761E+5	98.19

Table S25. HPLC method. Mobile Phase A: CH_3CN. Mobile phase B: 0.1 % (v/v) formic acid in ultrapure H_2O. Flow rate: 1.000 [$mL\cdot min^{-1}$], injection volume: 1.5 µL. Sample spiked with 0.1 mL formic acid.

Time (min)	Mobile Phase A (%)	Mobile Phase B (%)
0	30	70
10	65	35
